# Excited‐State Antiaromaticity in Nonbenzenoid Aromatics: Examining the Dynamics of Intramolecular Proton Transfer With a Small Driving Force

**DOI:** 10.1002/anie.6799620

**Published:** 2026-06-12

**Authors:** Promeet K. Saha, Charlotte A. Bardsley, Hector G. Miranda‐Salinas, Daniel Crane, Rabia Ayub, Andrew P. Monkman, Paul R. McGonigal

**Affiliations:** ^1^ Department of Chemistry University of York York UK; ^2^ Department of Chemistry University of Oxford Oxford UK; ^3^ Department of Physics Durham University Durham UK

**Keywords:** acidity, Baird's rules, excited‐state proton transfer, photochemistry, tropylium

## Abstract

The relief of excited‐state antiaromaticity (ESAA) is a powerful driving force that underpins the photochemical behavior of photoexcited aromatic molecules. Photo‐induced isomerization, cycloaddition, or proton transfer pathways alleviate the large energetic destabilization caused by ESAA. However, the excited‐state dynamics of annulenes displaying only weak ESAA remain largely unexplored. Here, we fine‐tune the electronic character of a series of annulenes to minimize their ESAA. Excited‐state proton transfer (ESPT) converts hydroxytropyliums (HTs) and hydroxybenzotropyliums (HBTs) to their nonaromatic tautomers, which are only slightly lower in energy than the excited‐state antiaromatic forms. As a result of this near‐degeneracy, we find that HBTs exhibit weak photoacidity (pKa drop of 0.3–1.2 units) and unusually slow, reversible ESPT, proceeding on the ns timescale rather than the typical ps timescale. The fluorescence spectra of these HBTs display the hallmarks of ESPT emitters: (i) dual fluorescence; (ii) excitation‐dependent emission; and (iii) large Stokes shifts. The driving force for this ESPT is increased by the addition of electron‐donating groups, which promote intramolecular charge transfer. Our results demonstrate that judicious structural changes can moderate the ESAA of nonbenzenoid annulenes, minimizing the driving force for ESPT, which leads to slow proton transfer kinetics and weak photoacidity.

## Graphical abstract

The excited‐state antiaromaticity of hydroxybenzotropyliums (HBTs) can be tuned through substituent effects to bring the aromatic ‘enol’ (E) and keto (K) tautomers close in energy. This leads to abnormally slow excited‐state proton transfer (on the ns timescale), giving rise to dual fluorescence emission and weak photoacidity.

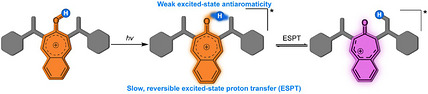

## 1 Introduction

Aromaticity arises as a result of the cyclic delocalization of π‐electrons in a ring [1]. Typically, aromatic molecules such as benzene exhibit exceptional stability and chemical inertness [2, 3]. However, the energetic benefits of aromatic stabilization are limited to the electronic ground states of such molecules [4, 5]. Baird first predicted on the basis of perturbation MO theory calculations that aromatic molecules become antiaromatic in their lowest triplet π–π* excited states [6, 7], leading to an altered electronic configuration [5, 8], structural changes [9, 10], and increased photoreactivity [11, 12]. Since this first prediction, there have been several experimental [9, 13, 14, 15] and theoretical [16, 17] investigations of the rich photochemistry arising from the ESAA of benzene and related benzenoid derivatives [18].

**FIGURE 1:**
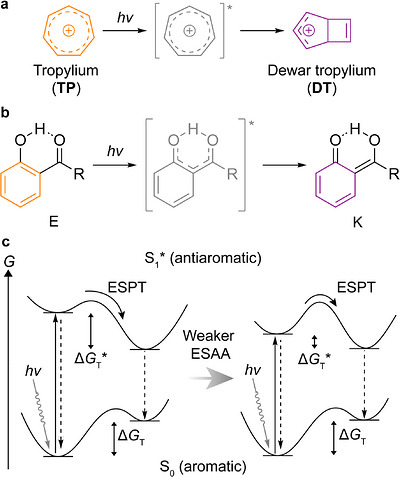
Effects of ESAA on ground‐state aromatic annulenes include (a) photoisomerization of **TP** to the nonaromatic, bicyclic **DT** isomer and (b) ESPT of *o*‐salicylic acid from an E tautomer to a quinoidal K tautomer. The schematic energy diagram (c) illustrates the impact of reducing ESAA on the energetics of ESPT.

Similarly to the chemistry of benzene, photoexcitation of the tropylium (**TP**) cation—a *C*_7_‐symmetric, aromatic ring—induces its isomerization to a bicyclic “Dewar tropylium” (**DT**) cation (Figure 1a) [19]. This property has been leveraged in the context of organocatalysis in recent years [20, 21]. Computational studies have tentatively attributed this photoisomerization to the relief of antiaromaticity in the excited state of nonbenzenoid annulenes [22], but quantification of this putative destabilization has remained elusive.

In addition to promoting photocyclization reactions, the relief of ESAA also provides a driving force for ESPT, that is, photo‐induced tautomerization by proton transfer from a donor to an acceptor moiety [23, 24]. An early observation of this phenomenon was reported for *o*‐salicylic acid [25, 26] (Figure 1b), which undergoes ESPT, converting from an aromatic ‘enol’ (E form) to a nonaromatic keto (K form) tautomer. This isomerization is identified by characteristic features in the electronic spectra, such as (i) a prominent Stokes shift, as well as (ii) a dual emission profile, arising from the presence of two emissive species in the excited state (Figure 1c) [27]. More recently, red‐light emitters [28] and dual white‐light emitters [29] based on single benzene fluorophores have been designed by exploiting ESPT driven by ESAA relief [30, 31].

As part of our ongoing investigations into the photophysics of seven‐membered ring compounds [32, 33, 34], we recently reported that counterbalancing strain arising from periphery overcrowding against the ground‐state aromatic stabilization energy of tropylium cations can cause them to rupture, giving rise to a thermal aromatic‐to‐nonaromatic equilibrium [35]. This transform‐ation is enabled by the relatively low aromatic stabilization energy of the **TP** cation, which is approximately half that of benzene [35]. As a general strategy, lowering or offsetting aromatic stabilization energy (by geometry control [36]) can “switch on” an otherwise energetically inaccessible process. One could imagine applying the same logic to the excited states of aromatic molecules to tune the dynamics of photoisomerization reactions. To date, however, it has been challenging to fine‐tune excited‐state antiaromatic destabilization energies. Therefore, the dynamics and kinetics of photoisomerization in molecules with weak ESAA, and hence, a small driving force for photoreactivity, remains largely unexplored.

**FIGURE 2:**
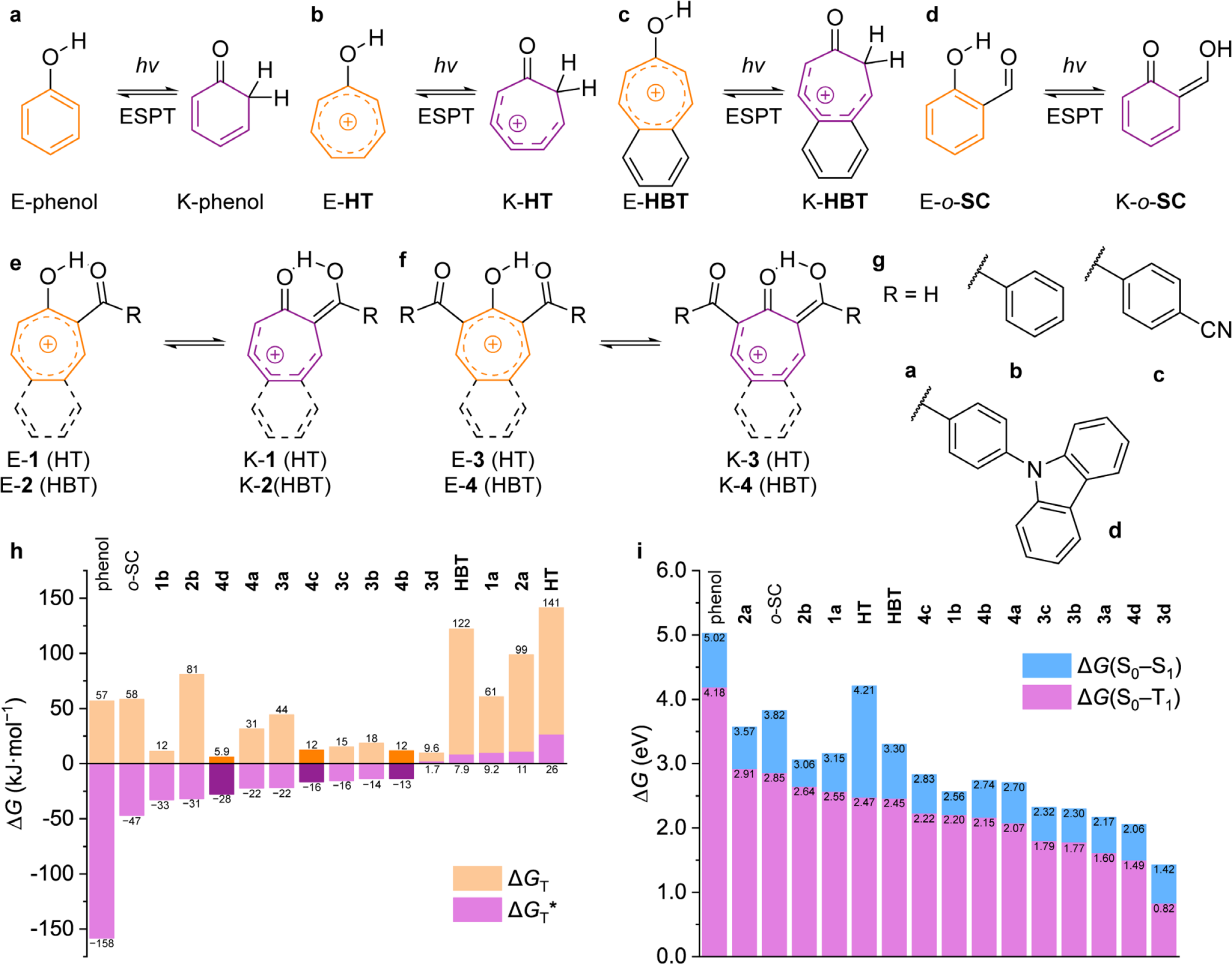
The E and K tautomers of model compounds (a) phenol; (b) **HT**; (c) **HBT**; and (d) *o*‐**SC**, as well as (e) mono‐ and (f) di‐functionalized HT‐ and HBT derivatives with (g) a series of substituents. (h) Calculated ground‐state (S_0_) and excited‐state (T_1_) Gibbs energy differences, Δ*G*_T_ and Δ*G*_T_*, respectively, between the E‐ and K‐tautomers. (i) Calculated S_0_–S_1_ and S_0_–T_1_ Gibbs energy differences (Δ*G*) of E tautomers. ((U)ωB97XD/6‐31G(d) for S_0_ and T_1_ state, TD‐ωB97XD/6‐31G(d) for S_1_ state).

Here, we report the reversal of the ground‐state aromatic stabilization of a series of tropylium derivatives, HTs and HBTs (Figure 2), upon excitation to the lowest π–π* excited state. The modest ESAA of these species manifests itself through their weak photoacidity, that is, an increase in acidity (*p*K_a_ drop of up to 1.2 units) upon absorption of light. Time‐resolved photolum‐inescence (TRPL) spectroscopic analysis of the excited states of the HBTs reveals that the smaller driving force for ESPT translates to abnormally slow rates of proton transfer [37, 38, 39] (on the ns timescale) from the hydroxy group of the benzotropylium core to the neighboring acetophenyl moieties, which act as proton acceptors to alleviate the ESAA. The slow kinetics of proton transfer give rise to a dual steady‐state fluorescence profile that is characteristic of many ESPT emitters [40]. We find that the addition of electron‐donating substituents conjugated with the proton‐accepting carbonyl groups produces a donor–acceptor structure, wherein intramolecular charge transfer (ICT) increases the driving force for ESPT. Our investigation demonstrates ESAA relief with a small driving force as a source of the distinct chemical and photophysical behavior of (weakly) aromatic annulenes.

## 2 Results and Discussion

Previous calculations have quantified the ground‐state aromatic stabilization of benzene [41, 42], as well as its antiaromatic destabilization in the singlet [43] and triplet excited states [22, 44]. Despite benefitting from lower ground‐state stabilization [35], the **TP** cation experiences equally high antiaromatic destabilization in its triplet excited state [22, 44]. With this in mind, we used density functional theory (DFT) and time‐dependent DFT (TD‐DFT) to assess the energetics of both ground‐state proton transfer (GSPT) and ESPT in HTs and HBTs. We modeled the S_0_ and T_1_ states by DFT and the S_1_ state by TD‐DFT. A screen of functionals revealed that the (U)ωB97XD functional most accurately predicted the (anti)aromatic characters of these species (Table S28) [45]. The 6‐31G(d) basis set was used to model the structural parameters of these systems, while the 6‐311+G(d,p) basis set was required to model the electronic and magnetic properties [46]. As a standard approach [8, 47, 48, 49], complete active space self‐consistent field (CASSCF) calculations with 6‐311++G(d,p) and def2‐svp single‐point basis sets were employed to model the magnetic and electronic characteristics, respectively in the S_1_ state [50].

### 2.1 Antiaromatic Destabilization of HTs and HBTs

Decorating the aromatic cores of phenol, **HT**, and **HBT** (Figure 2a–c) with a proton‐accepting carbonyl group gives *o*‐salicylaldehyde (*o*‐**SC**; a well‐known structural motif in ESPT luminogens) [51, 52, 53, 54], its homolog **1a**, and the benzannulated **2a** (Figure 2d,e), respectively. We began by calculating the ground‐state (S_0_) aromaticity indices (Table 1) of the E forms of **1a** and **2a** (Figure 2c), and comparing them to *o*‐**SC**. While all species exhibit ground‐state aromaticity, structural, magnetic, and electronic criteria (Tables 1, S12, and Figures S36–S45) are indicative of both **1a** and **2a** having somewhat decreased aromatic character compared to *o*‐**SC**. First, the harmonic oscillator model of aromaticity (HOMA) parameters [55, 56] of 0.644–0.868 reveal more bond‐length alternation in **1a** and **2a** compared to *o*‐**SC** (0.941), which has nearly equal C─C bond lengths in its structure as expected for an idealized aromatic ring. Second, the *zz* components of the nucleus‐independent chemical shifts (NICS*_zz_* (±1)) [57, 58] range from −32.6 to −16.1, confirming the presence of diatropic ring currents in a magnetic field [59]. We visualized these ring currents using anisotropy of the induced current density (ACID) plots (Figures S37–S44) [60]. The diatropic ring current in **2a** encompasses both the tropylium, as well as the appended benzene ring, indicating the benzenoid ring contributes stability through charge delocalization. Third, to quantify the number of electrons delocalized in the aromatic circuits, we performed electron density of delocalized bond (EDDB*^k^*) calculations (Figures S45–S47) [61]. The extent of electron delocalization in *o*‐**SC**, **1a** and **2a** are comparable. Together, these analyses corroborate the ground‐state aromaticity of the three compounds and the slightly diminished aromatic character of **1a** and **2a** relative to *o*‐**SC** (as determined by structural and magnetic criteria).

**TABLE 1:** Comparison of the ground‐state (S_0_) and excited‐state (S_1_ and T_1_) aromaticity indices of phenol derivative *o*‐**SC**, HT **1a**, and HBT **2a**.

	*o*‐SC			1a			2a^a^		
State	S_0_	S_1_	T_1_	S_0_	S_1_	T_1_	S_0_	S_1_	T_1_
HOMA^b^ (E)	0.941	0.904	0.392	0.802	0.783	0.764	0.644, 0.868, 0.780	0.721, 0.678, 0.781	0.694, 0.709, 0.779
HOMA^b^ (K)	0.305	0.617	0.494	0.420	0.656	0.704	−0.067, 0.864, 0.329	0.327, 0.768, 0.577	0.617, 0.649, 0.726
NICS*_zz_* (1)^c^ (E)	−23.9	56.4	27.3	−18.3	59.1	−2.4	−12.1, −31.7	57.6, −13.6	−1.4, −20.1
NICS*_zz_* (1)^c^ (K)	−9.9	11.3	1.9	−6.2	9.2	2.4	2.3, −23.2	12.1, −8.5	7.8, −15.2
Δ*G*_T_^(^*^)^ (kJ·mol^−1^)	58	−5.6	−47	61	5.1	9.1	99	−36	11

^a^ Values refer to the tropylium, benzene, and the HBT perimeter, respectively.

^b^ [(U)ωB97XD/6‐31G(d)].

^c^ NICS*_zz_* (1) values were calculated 1 Å above the averaged planes of the tropylium/benzene rings [S_0_,T_1_: (U)ωB97XD/6‐311+G(d,p)//ωB97XD/6‐31G(d); S_1_: CASSCF/6‐311++G(d,p))//TD‐ωB97XD/6‐31G(d)].

On the other hand, excitation to the lowest singlet (S_1_) excited state induces antiaromaticity, in accordance with Baird's rules [4, 6]. Large, positive NICS*_zz_* values (Table 1) ranging from +56.4 to +59.1 indicate the emergence of paratropic (antiaromatic) ring currents in all three compounds. This antiaromaticity is also associated with increased bond localization (decreases in HOMA values), concomitant with reduced electron delocalization (smaller EDDB*^k^* values). Interestingly, while *o*‐**SC** gains antiaromaticity in the lowest triplet (T_1_) excited state, the TP rings in **1a** and **2a** become non‐aromatic (NICS values ∼0) and the benzene ring in **2a** retains its aromaticity.

### 2.2 ESAA Relief Through Proton Transfer

Next, we examined the impact of intramolecular proton transfer (Figure 2) on the (anti)aromaticity of *o*‐**SC**, **1a**, and **2a**. In the S_0_ state, proton transfer converting the E form to the K tautomer is accompanied (Table 1) by a loss in aromaticity (increases in NICS*_zz_* values to ∼0 and marked decreases in HOMA values), arising from the disruption of the aromatic circuits (as visualized in the EDDB*^k^* plots shown in Figures S45–S47). In the S_1_ excited state, however, these ESPT reactions lead to large decreases in NICS*_zz_* in all three species. These data, in conjunction with the ACID and EDDB*^k^* plots for these structures, confirm the disappearance of antiaromatic ring currents, although the K forms retain some antiaromatic character (based on their NICS*_zz_* values between +9.2 to +12.1). No significant changes in these aromaticity indices are observed in the T_1_ state.

To assess the energetic impact of GSPT and ESPT, we calculated tautomerization energies in the ground and excited states (Δ*G*_T_ and Δ*G*_T_*, respectively), that is, the Gibbs energy differences between the K‐ and E‐tautomers. As GSPT leads to the loss of aromaticity, it is endergonic in *o*‐**SC** (Δ*G*_T_ = +58 kJ·mol^−1^). Tautomerization is even more disfavored in **1a** and **2a** (Δ*G*_T_ = +61 and +99 kJ·mol^−1^, respectively), as it decreases conjugative stabilization of the carbocation in addition to quashing aromaticity. Owing to the ESAA of *o*‐**SC** in its S_1_ and T_1_ excited states, ESPT becomes favorable (Δ*G*_T_* = −5.6 and −47 kJ·mol^−1^, respectively). Similarly, the ESAA in the S_1_ excited states of **1a** and **2a** reduces the energetic penalty of proton transfer. We find a Δ*G*_T_* value of +5.1 and −36 kJ·mol^−1^ for **1a** and **2a**, respectively. As the TP ring is non‐aromatic in the T_1_ state, ESPT remains disfavored (Δ*G*_T_* = 9.1 and 11 kJ·mol^−1^, respectively). Overall, these aromaticity indices allow us to (i) contrast the aromatic ground states of annulenes with their antiaromatic excited states and (ii) explore the impact of proton transfer in a qualitative manner. We estimate the magnitude of the antiaromatic destabilization through their Δ*G*_T_* values.

### 2.3 Tuning Excited‐State Dynamics

To assess the impact of substituents, we modeled Δ*G*_T_* of the S_1_ and T_1_ states for an extended series of annulenes (Table S27 and Figure 2c–h, respectively). While the S_1_ states of several of these species have n–π* or charge‐transfer characters, the T_1_ states are purely π–π* and serve as a more suitable platform to analyze the energetics of ESAA‐driven proton transfer. Compared to **1a**, the energetic preference can be increased in favor of K* further by (i) addition of another acyl moiety, as in **3a** (Δ*G*_T_* = −22 kJ·mol^−1^); or (ii) by extending the conjugation of the proton‐accepting moiety by a phenyl ring, as in **1b** and **3b** (Δ*G*_T_* = −33 and −13 kJ·mol^−1^, respectively).

Modifying the electronic character of the appended phenyl rings with electron‐withdrawing *p*‐cyano groups (**3c**) is predicted to have minimal effect on the Δ*G*_T_ and Δ*G*_T_* values of HTs. However, the addition of electron‐donating carbazole moieties (**3d**) increases the proton‐accepting ability of the acyl groups, leading to a small decrease in Δ*G*_T_. In contrast, Δ*G*_T_* rises slightly, bringing the E* and K* tautomers near degeneracy.

In general, the underlying principles that dictate the energetics of proton transfer in HTs can be extended to HBTs. Specifically, we find that the tautomerization energies (Table 2) of some bis(acetophenyl) derivates of HT (**3b**–**c**) are within a few kJ·mol^−1^ of their HBT analogs (**4b**–**c**). A notable exception is **4d** (Δ*G*_T_* = −28 kJ·mol^−1^), where ESPT is significantly more favored than in **3d**, presumably owing to the increased conjugation of HBTs. From an energetic standpoint, we find that for this series of annulenes, a lower excited‐state energy (Figure 2i and S69) generally correlates with a smaller driving force for ESPT (i.e., a lower Δ*G*_T_*).

**TABLE 2:** Comparison of the ground‐state (S_0_) and excited‐state (S_1_ and T_1_) aromaticity indices of HBT‐derivatives.

	4b			4c			4d		
State	S_0_	S_1_	T_1_	S_0_	S_1_	T_1_	S_0_	S_1_	T_1_
HOMA^a^ (E)	0.615, 0.880, 0.833	0.671, 0.631, 0.722	0.621, 0.655, 0.714	0.624, 0.879, 0.758	0.669, 0.630, 0.725	0.624, 0.658, 0.721	0.611, 0.880, 0.749	0.655, 0.608, 0.647	0.572, 0.769, 0.707
HOMA^a^ (K)	0.223, 0.903, 0.753	0.459, 0.626, 0.581	0.456, 0.608, 0.599	0.252, 0.899, 0.532	0.458, 0.632, 0.587	0.449, 0.615, 0.604	0.177, 0.907, 0.482	0.312, 0.634, 0.496	0.457, 0.605, 0.598
NICS_zz_(1)^a^, ^b^, ^c^ (E)	−12.6, −28.4	50.3, −7.0	16.0, −9.6	−12.1, −25.8	49.4, −8.3	14.3, −11.5	−9.6, −21.3	47.2, −12.3	15.7, −20.6
NICS_zz_(1) ^a^, ^b^, ^c^ (K)	−5.6, −26.1	10.9, −8.6	13.3, −10.1	−5.8, −24.4	9.7, −8.4	12.4, −10.7	1.9, −21.9	9.3, −14.3	13.3, −24.7
Δ*G*_T_^(^*^)^ (kJ·mol^−1^)	12	−6.7	−13	12	−9.9	−16	5.9	−23	−28

^a^ Values refer to tropylium, benzene, and the HBT perimeter, respectively [(U)ωB97XD/6‐31G(d)].

^b^ NICS_zz_(1) values were calculated 1 Å above the averaged plane of the tropylium rings.

^c^ [S_0_,T_1_: (U)ωB97XD/6‐311+G(d,p)//(U)ωB97XD/6‐31G(d); S_1_: CASSCF/6‐311++G(d,p)//TD‐ωB97XD/6‐31G(d)].

A closer analysis of the NICS*_zz_* (1) values of HBT **4b** reveals that the ground‐state aromatic tropylium ring (NICS*_zz_* (1) = −12.6) develops ESAA in the S_1_ and T_1_ excited states (NICS*_zz_* (1) = +50.3, +16.0, respectively). The slightly exergonic ESPT reaction to convert E‐**4b** to K‐**4b** (Δ*G*_T_* = −6.7 and −13 kJ·mol^−1^ in the S_1_ and T_1_ states, respectively) is accompanied by a decrease (Table 2) in this NICS*_zz_* (1) value (from +50.3 to +10.9 in the S_1_ state and +16.0 to +13.3 in the T_1_ state), indicating moderate relief of ESAA in the tropylium ring. Unlike the tropylium ring, the annulated benzene ring of the HBTs retains its local aromaticity upon excitation and following ESPT, consistent with the frontier molecular orbitals being localized on the tropylium in the excited state (NICS*_zz_* (1) = −28.4 for the E‐form in the S_0_ state, *c.f*. −7.0 and −9.6 in the S_1_ and T_1_ states, respectively). Similar conclusions can be drawn for both **4c** (*p*‐cyanophenyl) and **4d** (*p*‐(9‐carbazolyl)phenyl). ACID plots (Figures S49–S54) are consistent with ESPT causing the disappearance of the antiaromatic ring current in the T_1_ state, even though EDDB*^k^* values (Figures S55–S68) indicate that the overall electron delocalization in the molecule does not change significantly. This apparent discrepancy arises, due to magnetic aromaticity descriptors overstating the magnitude of ESAA relative to electronic criteria [31].

Overall, this analysis suggests that in HBTs decorated with two acetophenyl groups (**4b**), ESPT has a small driving force, as it relieves the weak ESAA of the E* tautomer. Tuning the electronic character of the appended phenyl rings with electron‐withdrawing substituents (**4c**) has a minimal effect on the energetics of proton transfer, while electron‐donating groups (**4d**) increase the driving force for ESPT.

### 2.4 Synthesis of Hydroxybenzotropyliums

Guided by our DFT calculations, we identified **4b**–**d** as suitable synthetic targets whose ESPT behavior could be controlled by varying the substituents on the acetophenyl moieties. An aromatic amine (carbazole) was chosen as the electron‐donating group over an aliphatic amine with higher basicity, as it is not prone to protonation under the acidic conditions used to prepare HBTs. We restricted our studies to HBTs only, as HTs are known to undergo constitutional isomerization upon photoexcitation [19, 20], which would further complicate our analysis.

**SCHEME 1:**
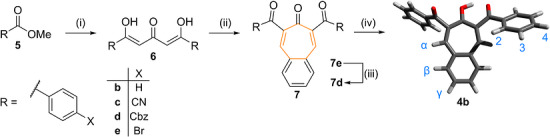
Synthesis of HBTs. Reagents and conditions: (i) acetone, NaH, rt→reflux, 24 h; (ii) *o*‐phthalaldehyde, piperidine, EtOH, reflux, 15 min, 68% (**7b**), 66% (**7c**), 22% (**7e**); (iii) **7e**, Pd_2_(dba)_3_, XPhos, Cs_2_CO_3_, PhMe, reflux, 24 h, 74% (**7d**); (iv) MsOH, CDCl_3_, rt, 10 min, quant. The structure shown of **4b** is a DFT‐optimized ground‐state model (ωB97XD/6‐31G(d)). Cbz = 9‐carbazolyl.

First, Claisen condensation reactions between methyl benzoate esters **5** and acetone afforded a series of 1,3,5‐diaryl‐pentanetriones **6** (Scheme 1) [62]. Subsequent Knoevenagel condensations with *o*‐phthalaldehyde formed benzotropones **7** [63]. While these conditions afforded pure **7b** and **7c** in high yields, **7e** was obtained in a lower yield (22%), as it required a complex purification protocol (Section S2). A Buchwald–Hartwig coupling of 9*H*‐carbazole [64] converted **7e** to **7d** in high yields. Treating benzotropones **7b**–**d** with a large excess of methanesulfonic acid (MsOH) gives the corresponding HBT mesylate salts **4**·OMs in quantitative yields [65]. Despite several attempts, we were unable to grow single crystals of **4b**–**d**·OMs suitable for x‐ray diffraction, presumably on account of decomposition caused by concentrating solutions in the presence of the large excess of acid required to generate the cation. A geometry‐optimized DFT model of **4b** (Scheme 1) shows the presence of the acetophenyl groups does not disrupt the planarity of the aromatic HBT ring system.

**FIGURE 3:**
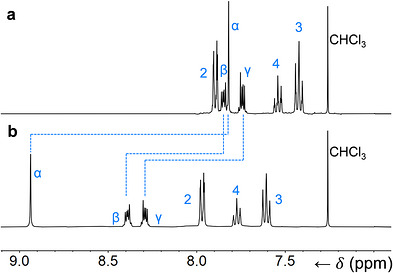
Partial ^1^H NMR (400 MHz, CDCl_3_, 298 K) spectra showing the protonation of (a) **7b** with MsOH to form (b) **4b**·OMs. The peak labels correspond to those shown in Scheme 1.

One of the two carbonyl groups is coplanar with the aromatic core, enabling the formation of an intramolecular hydrogen bond, while the other twists out of the plane of the HBT by a torsion angle of 62.5°. In addition to this *syn* arrangement of the carbonyl groups, our calculations indicate the presence of a low‐lying *anti* rotamer (Figure S46) with an overall L‐shaped structure. ^1^H NMR spectroscopic analysis of **4b**·OMs (Figure 3) reveals a pronounced downfield shift of the ^1^H resonances of the HBT core compared to the neutral **7b** precursor, consistent with the formation of an aromatic cation. We determined the p*K*_a_ of **4b**·OMs by an NMR acid–base titration, where the increase in chemical shift (Δ*δ*) of the α‐protons of **7b** was monitored at increasing concentrations of MsOH in CDCl_3_ (Figures S16 and S18).

Fitting these changes to the Henderson–Hasselbach equation yields p*K*_a_ = 1.69 ± 0.02, which is similar to structurally related HBTs [65]. By comparison, we measured a p*K*_a_ of 1.22 ± 0.03 for **4d** (Figures S17 and S19), indicating higher acidity of this HBT [66].

### 2.5 Optical Properties and Photoacidity

**FIGURE 4:**
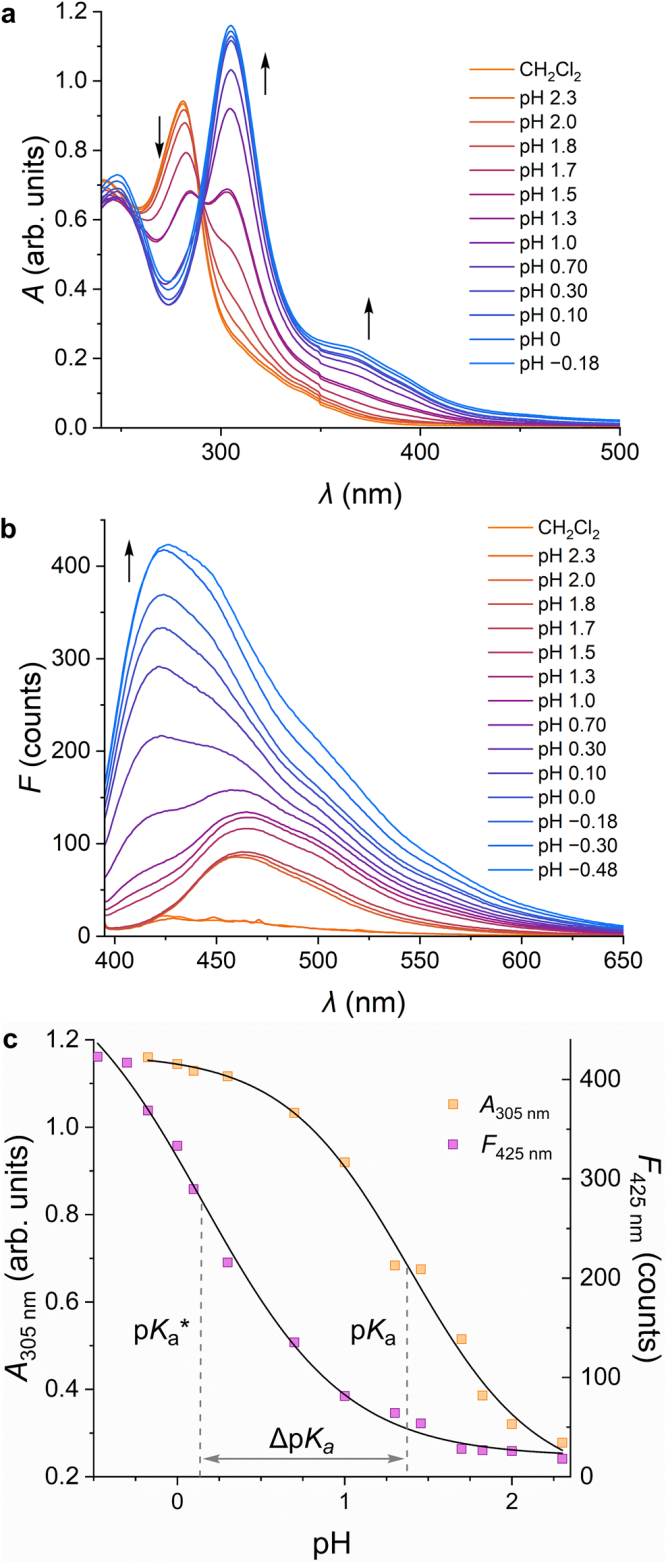
Photoacidity of **4b**. (a) UV–vis absorption and (b) fluorescence emission spectra of **7b** (*c* = 10 µM, *l* = 10 mm, *λ*_ex_ = 375 nm) in anhydrous CH_2_Cl_2_ at different pH values. (c) Variation of the absorbance at 305 nm (*A*_305 nm_) and emission at 425 nm (*F*_425 nm_) of **7b** with pH. The lines of best fit correspond to one‐step protonation equilibrium models, giving p*K*_a_ = 1.39 ± 0.06 and p*K*_a_* = 0.15 ± 0.08.

To understand the electronic character of the ground and excited states of HBT **4b**, we recorded UV–vis spectra (Figure 4a) and fluorescence emission spectra (Figure 4b) of **7b** in increasingly acidic media. In general, the protonation of **7b** to form **4b** causes a redshift in absorption on account of the increased conjugation present in the HBT.

Protonation also increases the photoluminescence response. While **7b** is only weakly emissive, giving a photoluminescence quantum yield, *Φ*_F_, of 0.011 (Table 3), **4b** emits with a maximum (*λ*_max_) centered at 425 nm with *Φ*_F_ = 0.15, and has a shoulder peak with *λ*_max_ = 511 nm. By following the increase in the absorption at *λ*_max_ = 305 nm and the emission maximum at *λ*_max_ = 425 nm, we were able to determine the ground‐state p*K*_a_ and the S_1_ excited‐state p*K*_a_* of **4b** (Figure 4c). The p*K*_a_ of **4b** derived by UV–vis spectroscopy (1.39 ± 0.06) is close to the value measured by ^1^H NMR spectroscopy (vide supra) [67].

**TABLE 3:** Photophysical properties of benzotropones **7b**–**d** and HBTs **4b**–**d**.

Species	Band (nm)	*Φ*_F_	a_1_ ^a^	τ_1_ (ns)	*τ*_2_ (ns)	S_1_ (eV)^b^	^3^LE (eV)^d^	Δ*E*_S–T_ (eV)
**7b**	400	0.011	0.8	1.68	0.15	3.74^b^	2.91	0.83
**4b**	425	0.119	1	2.31	—	3.25^b^	2.73	0.52
	510	0.005	0.24	6.29	1.49	2.81^c^		
**7c**	375	0.012	0.39	0.49	1.99	4.27^b^	2.60	1.67
**4c**	425	0.187	1	2.00	—	3.40^b^	2.70	0.70
	500	0.005	0.05	7.02	1.77	3.18^c^		
**7d**	400	0.011	0.69	1.52	0.066	3.83^b^	2.89	0.94
	500	—	1	15.3	—	—		
**4d**	420	0.128	1	1.94	—	3.42^b^	2.88	0.54
	530	0.003	0.17	8.18	1.55	2.83^c^		

^a^ The amplitude of the exponential decay.

^b^ All S_1_ energies were obtained using the steady state onset.

^c^ Energy onset obtained from the prompt emission TRPL spectrum after 10 ns.

^d^ Local triplet energies were obtained using the energy onset of late‐ms emission of the TRPL spectra.

By contrast, **4b** shows a modest, yet unambiguous increase in acidity in its S_1_ state, with p*K*_a_* = 0.15 ± 0.08 (i.e., a decrease of ∼1.2 units). This change indicates **4b** is a weak photoacid [68]—it undergoes more facile deprotonation upon photoexcitation.

Addition of the electron‐withdrawing nitrile groups in **4c** further weakens the photoacidity. Optical spectroscopy (Figures S21 and S22) shows a very small decrease in the p*K*_a_ of **4c** (Figure S24) of only ∼0.35 units from 0.26 ± 0.03 in the ground state to −0.09 ± 0.01 in the excited state [69]. These small observed decreases in p*K*_a_ are consistent with our DFT calculations, which predict that weak ESAA provides a small driving force for ESPT.

### 2.6 Excited‐State Kinetics

When comparing a family of closely related one‐step reactions with similar mechanisms, the one with the smaller thermodynamic driving force (Δ*G*) is also expected to proceed with a slower rate (Bell–Evans–Polanyi principle) [70, 71, 72]. The weak driving force for ESPT in **4b**, that is, its small Δ*G*_T_* and Δp*K*_a_ compared to benzenoid systems can give rise to (i) markedly slower kinetics of proton transfer [73]; and (ii) reversible ESPT [39, 74, 75]. While ESPT is typically known to occur on the fs‐to‐ps timescale [76], we expect ns‐timescale ESPT in **4b** [77].

**FIGURE 5:**
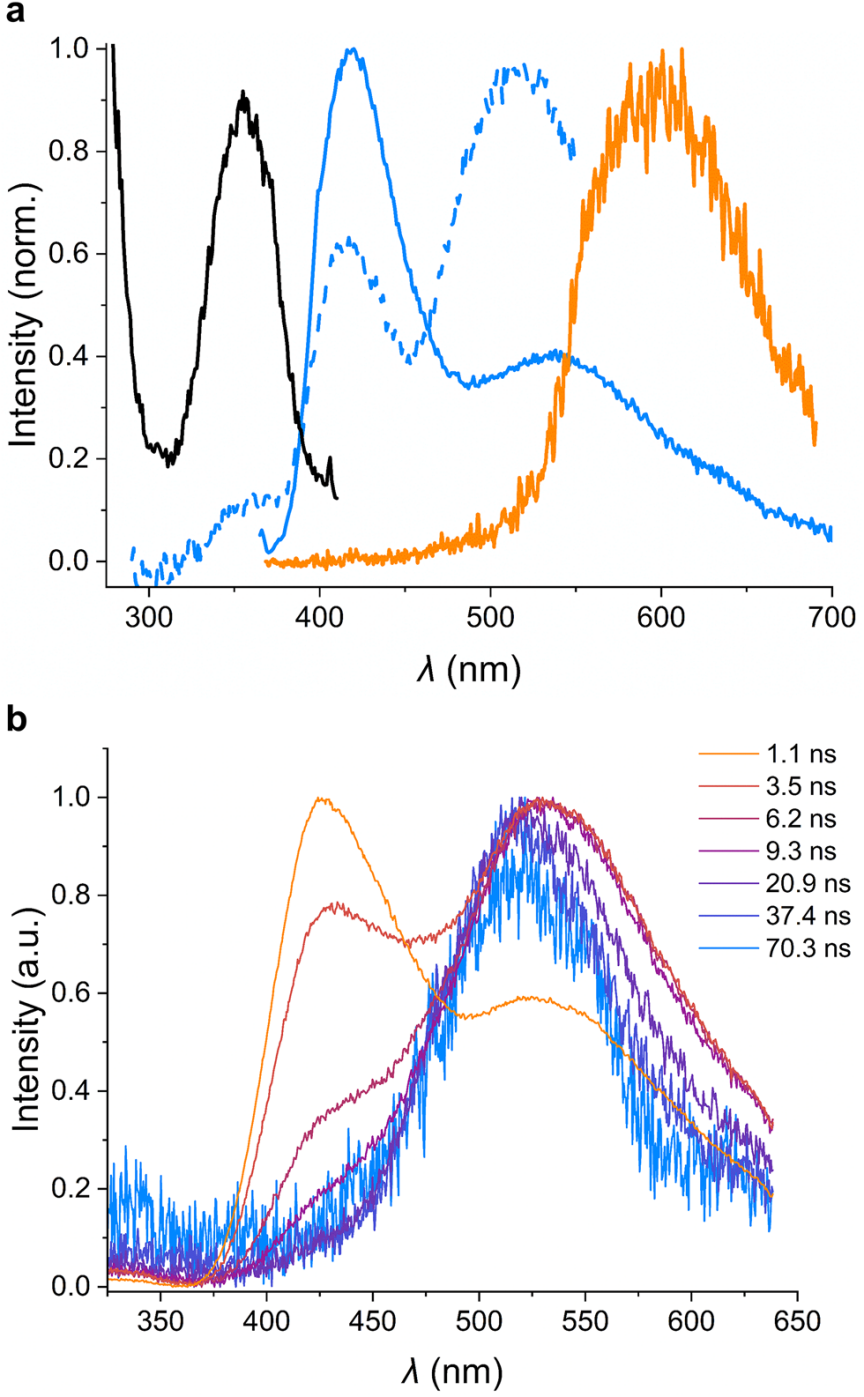
(a) Steady‐state excitation (black line, *λ*_em_ = 420 nm, 298 K), emission (solid blue line: *λ*_ex_ = 355 nm, dotted blue line: *λ*_ex_ = 280 nm, 298 K), and phosphorescence spectrum (orange line, *λ*_ex_ = 355 nm, 80 K) of **4b**; (b) TRPL spectra of **4b** (298 K). All spectra were recorded in 1 M solutions of MsOH in anhydrous CH_2_Cl_2_ (*c* = 2 µM, *l* = 10 mm).

ESPT on such timescales can compete with radiative decay, giving rise to dual fluorescence emission [78]. Indeed, the photoluminescence spectrum of **4b** exhibits two maxima centered at 425 and 510 nm with excitation wavelength‐dependent relative intensities (Figure 5a). We assign these peaks to emission from the (anti)aromatic E*‐**4b** and tautomerized K*‐**4b** singlet excited states, respectively. The emission maximum at 510 nm (2.43 eV), arising from an excitation peak at *λ*_ex_ = 355 nm (3.49 eV), corresponds to a large Stokes shift of (1.06 eV), which is characteristic of ESPT emitters. Upon cooling to 80 K, the emission of **4b** redshifts further to 600 nm in degassed solutions, which we attribute to phosphorescence emission from the T_1_ excited state.

To gain further insight into the kinetics of ESPT and explore the time evolution of these spectral features, we studied **4b** by TRPL spectroscopy (Figure 5b). The propensity of **4b**–**d** to aggregate (Figures S25–S35) precluded transient absorption spectroscopic studies, which typically require significantly higher concentrations. Moreover, the spectral overlap between the emission from K*‐**4b** and aggregates of E*‐**4b** introduces a competing decay pathway for E*‐**4b** in addition to ESPT and non‐radiative decay. As a result, we were unable to reliably determine a rate of ESPT (*k*_ESPT_) from steady‐state *Φ*_F_ values and time‐correlated single‐photon counting lifetime measurements alone. Since K* is predicted to be only a few kJ·mol^−1^ lower in energy than E*, we expect ESPT to be reversible in the S_1_ state.

Indeed, the notably low *Φ*_F_ of K*‐**4b** (*Φ_F_* = 0.005) compared to E*‐**4b** (*Φ_F_* = 0.119) may be explained by the rate of reverse proton transfer (from K*‐**4b** to E*‐**4b**) competing with emission from K*‐**4b**. After 20 ns, the relative intensities of the two emission maxima of **4b** shift in favor of the redshifted K* emission, indicating the increase in its relative population, following ESPT. Fluorescence lifetime measurements (Table 3) reveal a short lifetime of emission, *τ*, from the antiaromatic S_1_ (E*) state of 2.3 ns and a comparatively longer lifetime of 6.3 ns for emission from the tautomerized K* form, further confirming the enhanced stability of the latter. This observation of dual fluorescence emission from **4b** indicates that ESPT is a slow, weakly driven process that can only produce a modest population of K*‐**4b**. Additionally, the low *Φ*_F_ of K*‐**4b** suggests ESPT is reversible, such that reverse proton transfer (K*→E*) is an additional non‐radiative pathway that suppresses K* emission. In sum, the progress of ESPT in **4b** can be followed through (i) its dual fluorescence spectrum featuring both E*‐ and K*‐emission with a moderate and prominent Stokes shift respectively, (ii) an increase in relative population of K* over time, and (iii) the longer *τ* of K* relative to E*. The excited‐state kinetics of HBT **4c** closely follow those of **4b**. The larger difference in fluorescence lifetimes of E*‐**4c** (*τ* = 2.0 ns) compared to K*‐**4c** (*τ* = 7.0 ns) can be attributed to its slightly larger Δ*G*_T_*.

To rule out conformational relaxation in the excited state (Figure S48 and Table S13) as a source of this dual fluorescence emission, we recorded a series of TRPL spectra of **4b**–**d** at low temperatures (80 K) in frozen solutions (Figures S27–S29, S32, and S34 and S35). While aggregation effects gave rise to complex emission spectra of **4b** (Figures S27–S29), **4c** retained its dual emission profile (Figure S32d) and exhibited a redshifting of the emission maximum over the ns‐timescale. As conformational interconversions are suppressed in these conditions, we assign this prominent Stokes shift to the effects of ESPT.

Adding electron donating substituents to the aryl group in **4b** creates a donor–acceptor–donor‐like structure (**4d**), where ICT can promote ESPT. The precursor benzotropones **7b** and **7c** emit a single wavelength of light, independent of the excitation wavelength (Figures S25 and S31). No significant spectral changes are observed over the ns timescale, suggesting that no ICT occurs upon excitation. As such, the dual emission observed in **4b** and **4c** is unlikely to originate from a charge transfer event. By contrast, excitation of **7d**, bearing electron‐donating carbazole groups, leads to ICT from the carbazole donor moiety to the benzotropone acceptor (Figures S70 and S71). This ICT leads to a redshifting of the fluorescence emission maximum of **7d** over the ns‐timescale from *λ*_max_ = 425 nm to $\lambda_{\max}^{\prime }$ = 505 nm (Figure S33). TRPL decay analysis reveals a lifetime of *τ* = 15.3 ns for the intramolecular charge transfer state. Protonation of **7d** to form **4d** establishes a strong hydrogen bond between the hydroxy group and one of the neighboring acyl groups, which facilitates efficient ICT in the ground state, as evidenced by its broad absorption band centered at *λ*_max_ = 550 nm (Figure S21). On the ns‐timescale, **4d** undergoes a similar charge transfer interaction as in **7d**, leading to a gradual redshifting in absorption (Figure S34), concomitant with ESPT.

## 3 Conclusion

Photoexcitation reverses the aromatic stabilization of TPs, giving rise instead to ESAA. The ESAA of nonbenzenoid aromatics is of mechanistic interest in the design of organocatalysts [20, 21] and has potential uses in in ESPT‐active luminogens, exhibiting large Stokes shift and dual emission [27, 28, 29, 30, 31]. Our TD‐DFT calculations, in conjunction with our spectroscopic analysis confirm that the destabilization is modest in HBTs. We have observed this effect experimentally as a small driving force for ESPT to a nonaromatic, K tautomer in **4b** and **4c**. The near degeneracy of the excited‐state tautomers leads to weak photoacidity (p*K*_a_ drop of approximately 1 unit) and unusually slow rates of reversible proton transfer on the ns timescale, as confirmed by TRPL spectroscopy. Decorating the proton‐accepting acyl moiety with electron‐donating carbazole groups (**4d**) leads instead to ICT, which further promotes ESPT. Our investigation provides experimental evidence for ESAA in nonbenzenoid annulenes and analyzes structural modifications that control the degree of this destabilization, providing a handle to regulate the kinetics and thermodynamics of their excited‐state reactivity and photoluminescence.

Unlike the [4*n*+2] annulenes studied in this work, 4*n* annulenes are non‐aromatic in their ground‐states, but can develop excited‐state aromaticity upon photoirradiation [79, 80]. While ESPT driven by the gain of excited‐state aromaticity has been explored computationally [81], examples of such chromophores remain sparse, owing to the unstable nature of 4*n* annulenes. We envisage that our approach, that is, the use of ionic odd‐numbered ring systems, could be extended to weaken the ground‐state antiaromaticity of 4*n* annulenes, while modulating their excited‐state aromaticity to tune their emission profiles.

## Author Contributions

**Promeet K. Saha**: conceptualization, investigation, writing – original draft, formal analysis. **Charlotte A. Bardsley**: investigation, writing – original draft, formal analysis. **Hector G. Miranda–Salinas**: investigation, writing – original draft, formal analysis. **Daniel Crane**: investigation, writing ‐ review and editing. **Rabia Ayub**: investigation, writing – original draft, formal analysis. **Andrew P. Monkman**: conceptualization, funding acquisition, writing – review and editing, supervision. **Paul R. Mcgonigal**: conceptualization, funding acquisition, writing – original draft, supervision.

## Conflicts of Interest

The authors declare no conflicts of interest.

## Supporting information




**Supporting File 1**: anie73122‐sup‐0001‐SuppMat.pdf.

## Data Availability

The data that supports the findings of this study are available in the supplementary material of this article.
