# Supplementary material for: Excited‐State Antiaromaticity in Nonbenzenoid Aromatics: Examining the Dynamics of Intramolecular Proton Transfer With a Small Driving Force
Source: Angew Chem Int Ed Engl. 2026 Jun 12;65(33):e6799620. doi: 10.1002/anie.6799620 (PMC13452492; doi:10.1002/anie.6799620)
Supplement: Supplementary file 1 [file ANIE-65-e6799620-s001.pdf]

Excited-State Antiaromaticity in Nonbenzenoid Aromatics: Examining the Dynamics of Intramolecular Proton Transfer with a Small Driving Force

Promeet K. Saha,[†] Charlotte A. Bardsley,[†,‡] Hector G. Miranda-Salinas,[#] Daniel Crane,[#] Rabia Ayub,[†] Andrew P. Monkman,[#] Paul R. McGonigal[†,‡,*]

[†]Department of Chemistry, University of York, Heslington, York, YO10 5DD, UK

[‡]Department of Chemistry, University of Oxford, Oxford, OX1 3TA, UK

[#]Department of Physics, Durham University, Lower Mountjoy, Stockton Road, Durham, DH1 3LE, UK

Table of Contents

1. General Methods S2
2. Synthetic Procedures S4
3. ^{1}H and ^{13}C NMR Spectroscopic Characterization of Synthesized Compounds S11
    3.1. pK_a Determination by NMR Spectroscopy S25
4. X-Ray Crystallographic Analysis of **7d** S30
5. UV-Vis Absorption Spectroscopy S32
6. Fluorescence Spectroscopy S36
7. Phosphorescence Spectroscopy S43
8. *In Silico* Modelling S62
9. References S205

Supporting Information

General Methods

Materials: All reagents were purchased from commercial suppliers (Sigma-Aldrich, Acros Organics, Fluorochem or Alfa Aesar) and used without further purification.

Instrumentation and Analytical Techniques: Analytical thin-layer chromatography (TLC) was performed on neutral aluminum sheet silica gel plates and visualized under UV irradiation (254 nm). Nuclear magnetic resonance (NMR) spectra were recorded using a Jeol ECX-400 or a Jeol ECS400 (^{1}H 400.130 MHz and ^{13}C 100.613 MHz), Varian Inova-500 (^{1}H 500.130 MHz and ^{13}C 125.758 MHz), or a Varian VNMRS-600 (^{1}H 600.130 MHz and ^{13}C 150.903 MHz) spectrometer, at a constant temperature of 298 K unless otherwise stated. Chemical shifts (δ) are reported in parts per million (ppm) relative to the signals corresponding to residual non-deuterated solvents [$CDCl_3$: δ = 7.26 or 77.16. CD_2Cl_2: δ = 5.32 or 54.00. $(CD_3)_2SO$: δ = 2.05 or 29.84 and 206.26]. Coupling constants (J) are reported in Hertz (Hz). ^{13}C NMR Experiments were proton decoupled. Assignment of ^{1}H and ^{13}C NMR signals were accomplished by two-dimensional NMR spectroscopy (COSY, NOESY, HSQC, HMBC). NMR spectra were processed using MestReNova version 15.0. Data are reported as follows: chemical shift; multiplicity; coupling constants; integral and assignment. Low-resolution ASAP-MS were performed using a Waters Xevo QTOF equipped with an Atmospheric Solids Analysis Probe (ASAP). High-resolution electrospray (HRESI), APCI and ASAP (HRASAP) mass spectra were measured using a Waters LCT Premier XE high resolution or a Bruker Daltronics micrOTOF MS, accurate mass UPLC ES MS (also with ASAP ion source). Melting points were recorded using a Gallenkanp (Sanyo) apparatus and are uncorrected. The X-ray single crystal data have been collected at a temperature of 120.0(2) K using MoKα radiation (λ = 0.71073Å) on a Bruker D8Venture with a Photon III MM C7 or C14 CPAD detector, IµS-III-microsource, focusing mirrors diffractometer equipped with a Cryostream (Oxford Cryosystems 700+) open-flow nitrogen cryostat. The structure was solved using Olex2[1] with

the ShelXT[2] structure solution program using Intrinsic Phasing and refined with the ShelXL[3] or Olex2.refine[4] refinement package using Least Squares minimization on F^2. All non-hydrogen atoms were refined with anisotropic displacement parameters. Hydrogen atoms were located on the difference map and refined isotropically on a riding model unless otherwise specified. Crystal data and parameters of refinement are listed in Table S3. Crystallographic data for the structure have been deposited with the Cambridge Crystallographic Data Centre with deposition number CCDC-2474833.

2. Synthetic Procedures

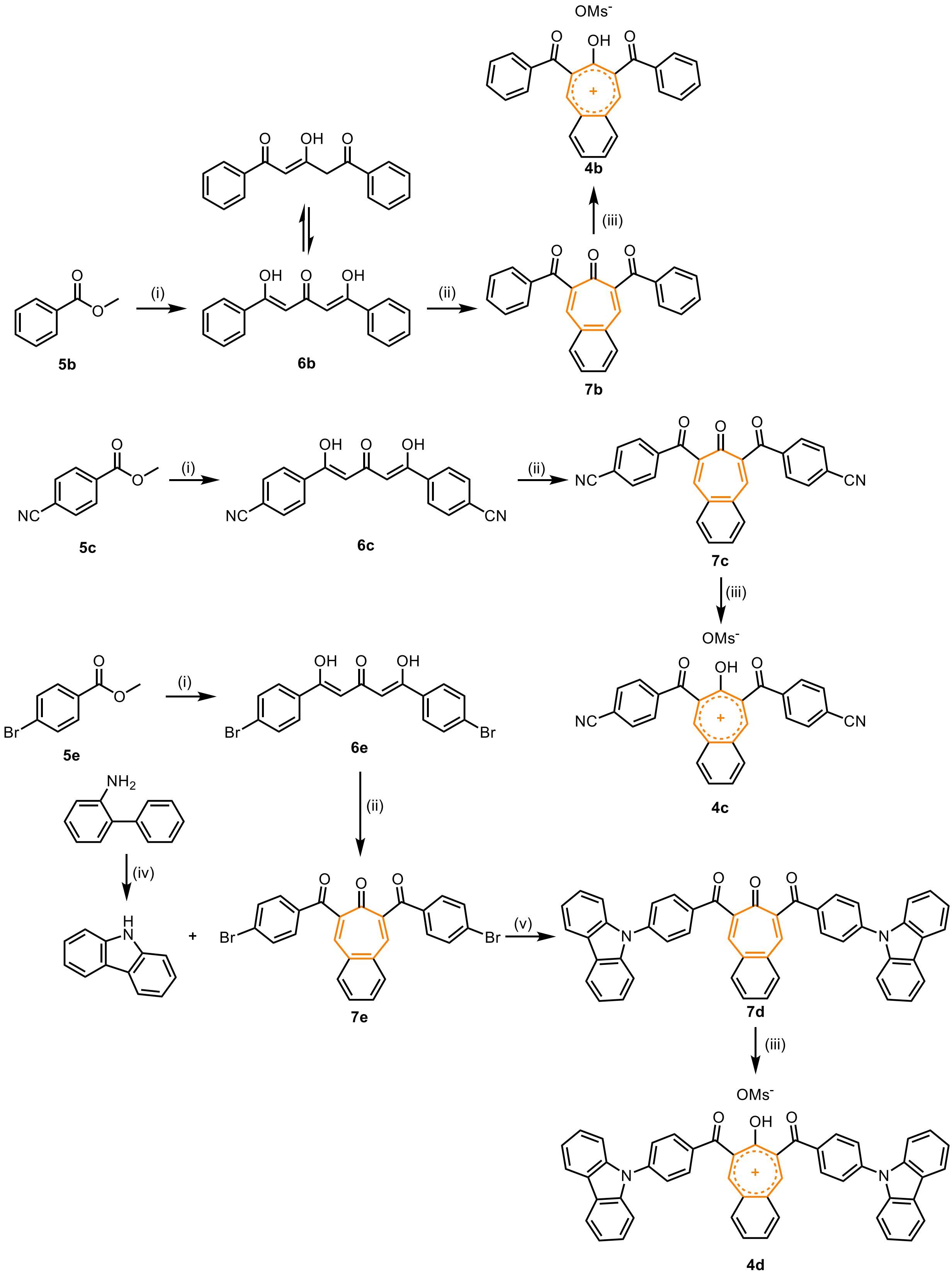

Fig. S1. Synthesis of tropylium cations **4b**–**4d**. Reagents and conditions: (i) Me_2CO, NaH, THF, 70 °C, 24 h; (ii) piperidine, phthalaldehyde, EtOH, 80 °C. 15 min, 68% (**7b**) over two steps, 66% (**7c**) over two steps, 22% (**7e**) over two steps; (iii) MsOH, $CDCl_3$, rt, 5 min, quant.; (iv) $HCl_{(aq)}$, $NaNO_2$, NaN_3, rt, 20 min, 69% (v) $Pd_2(dba)_3$, XPhos, Cs_2CO_3, PhMe, 100 °C, 24 h, 75%.

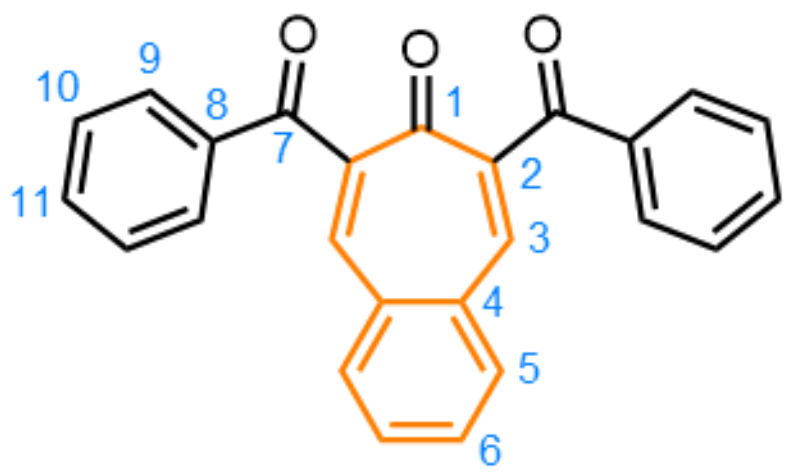

7b

7b: Anhydrous acetone (0.34 mL, 4.4 mmol, 1.0 eq.) was added dropwise to a suspension of NaH (60% in mineral oil, 1.07 g, 26.7 mmol, 6.0 eq.) in anhydrous THF (25 mL) in a flame-dried three-necked round bottomed flask fitted with a reflux condenser under an inert atmosphere at rt, followed by methyl benzoate (1.21 g, 9.0 mmol, 2.0 eq.). The mixture was heated to reflux for 24 h. Upon cooling to rt, the reaction mixture was poured onto a mixture of ice and water (300 mL) and acidified by careful addition of an aqueous solution of concentrated HCl (37% by wt.). The aqueous layer was extracted with $CHCl_3$ (3 × 75 mL). The combined organic extracts were washed with brine (150 mL) and dried over $MgSO_4$. The solvent was removed under reduced pressure, leaving a dark yellow residue, which was washed with MeOH (3 × 10 mL) to yield **6b** (1.0 g, 3.8 mmol, 0.9 eq.) as a pale-yellow solid that was taken on without further purification. Piperidine (0.12 g, 1.4 mmol, 0.3 eq.) was added dropwise to a refluxing solution of **6b** (1.0 g, 3.8 mmol, 0.9 eq.) and phthalaldehyde (0.37 g, 2.7 mmol, 0.6 eq.) in refluxing anhydrous EtOH (12 mL). The solution was refluxed at this temperature for a further 15 min. Upon cooling to rt, a precipitate formed, which was collected by filtration and washed with cold EtOH (2 × 5 mL) to afford the title compound as a colorless solid (0.81 g, 2.2 mmol, 68%). **M.P.** = 244–245 °C. **^{1}H NMR** (400 MHz, $CDCl_3$) δ 7.93–7.86 (m, 4H, H_9), 7.86–7.80 (m, 4H, $H_{3,5}$), 7.77–7.71 (m, 2H, H_6), 7.57–7.51 (m, 2H, H_{11}), 7.46–7.38 (m, 4H, H_{10}). **^{13}C NMR** (100 MHz, $CDCl_3$) δ 196.0 (C_7), 184.6 (C_1), 145.4 (C_2), 141.1 (C_3), 136.3 (C_8), 135.4 (C_5), 135.1 (C_4), 133.6 (C_{11}), 132.1 (C_6), 129.5 (C_9), 128.7 (C_{10}). **HR-APCI-MS** *m/z* = 365.1200 [M+H$^+$] (calculated for $C_{25}H_{17}O_3^+$ = 365.1135).

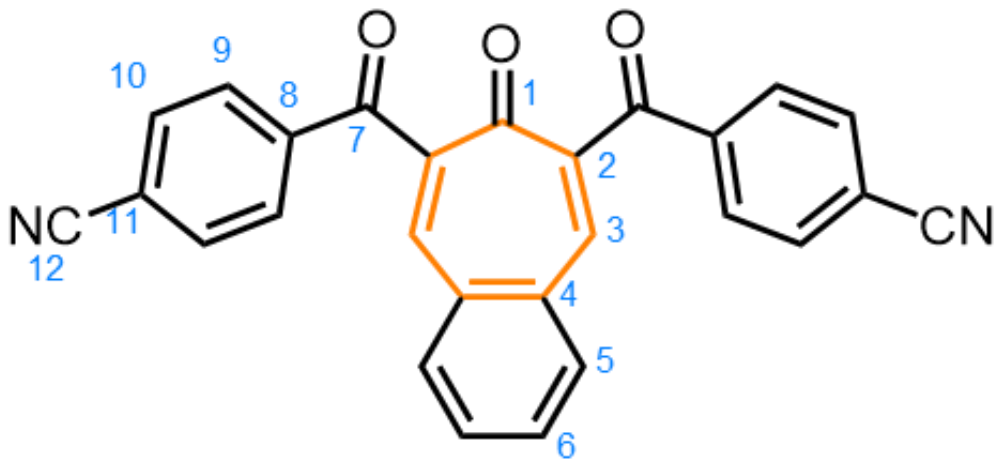

7c

7c: Anhydrous acetone (0.23 mL, 3.1 mmol, 1.0 eq.) was added dropwise to a suspension of NaH (60% in mineral oil, 0.74 g, 19 mmol, 6.0 eq.) in anhydrous THF (25 mL) in a flame-dried two-necked round bottomed flask fitted with a reflux condenser under an inert atmosphere at rt, followed by 4-cyanomethyl benzoate (1.00 g, 6.21 mmol, 2.0 eq.). The mixture was heated to reflux for 48 h. Upon cooling to rt, the reaction mixture was poured onto a mixture of ice and water (200 mL) and neutralized by careful addition of an aqueous solution of concentrated HCl (37% by wt.), leading to the formation of a yellow precipitate. The precipitate was collected by filtration under reduced pressure and washed with MeOH (3 × 20 mL), then dried under vacuum to yield the title compound **6c** (0.93 g, 2.9 mmol, 0.9 eq.). Piperidine (0.077 g, 0.90 mmol, 0.3 eq.) was added dropwise to a refluxing solution of **6c** (1.00 g, 2.9 mmol, 0.9 eq.) and phthalaldehyde (0.24 g, 1.81 mmol, 0.6 eq.) in refluxing anhydrous EtOH (12 mL). The solution was refluxed at this temperature for a further 15 min. Upon cooling to rt, a precipitate formed, which was collected by filtration and washed with EtOH to afford the title compound as a pale-yellow solid (0.66 g, 1.58 mmol, 66%). **M.P.** >350 °C. **^{1}H NMR** (400 MHz, $(CD_3)_2SO$) δ 8.23 (s, 2H, H_3), 8.19–8.14 (m, 2H, H_5), 8.03 (d, J = 8.6 Hz, 4H, H_9), 7.97 (d, J = 8.6 Hz, 4H, H_{10}), 7.90–7.85 (m, 2H, H_6). **^{13}C NMR** (100 MHz, $CDCl_3$) δ 195.4 (C_7), 185.0 (C_1), 143.9 (C_2), 143.5 (C_3), 139.9 (C_8), 136.4 (C_5), 135.6 (C_4), 133.4 (C_9), 133.2 (C_6), 129.9 (C_{10}), 118.6 (C_{11}), 115.9 (C_{12}). **HR-APCI-MS**: m/z = 415.1087 $[M+H]^+$ (calculated for $C_{27}H_{15}N_2O_3^+$ = 415.1077).

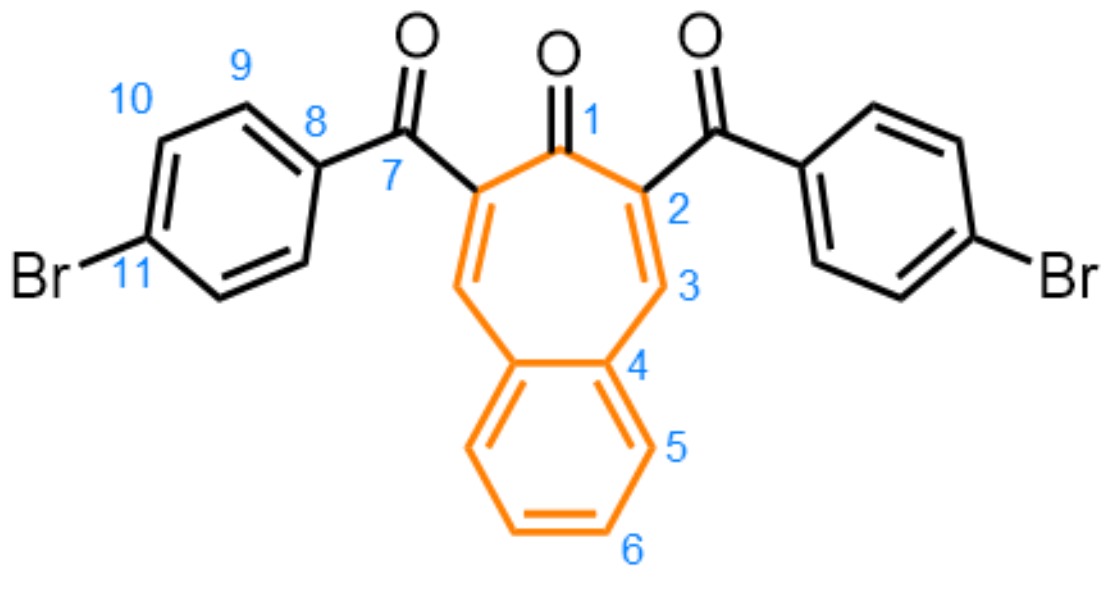

7e

7e: Anhydrous acetone (0.33 mL, 4.3 mmol, 1.0 eq.) was added dropwise to a suspension of NaH (60% in mineral oil, 1.0 g, 25 mmol, 6.5 eq.) in anhydrous THF (50 mL) in a flame-dried three-necked round bottomed flask fitted with a reflux condenser under an inert atmosphere at rt, followed by methyl 4-bromobenzoate (1.85 g, 8.6 mmol, 2.0 eq.). The mixture was heated to reflux for 24 h. Upon cooling to rt, the reaction mixture was poured onto a mixture of ice and water (100 mL) and neutralized by careful addition of an aqueous solution of concentrated HCl (37% by wt.). The aqueous layer was extracted with $CHCl_3$ (3 × 25 mL). The combined organic extracts were washed with brine (50 mL) and dried over $MgSO_4$. The solvent was removed under reduced pressure, leaving an orange residue, which was washed with MeOH (3 × 10 mL) to yield the title compound and **S1** (the mono-condensed intermediate species) as an inseparable mixture (**6e**:**S1** = 53:47, 1.0 g). Piperidine (0.10 g, 1.2 mmol, 0.3 eq.) was added dropwise to a refluxing solution of **6e** and **S1** (1.0 g, 0.6 eq.) and phthalaldehyde (0.32 g, 2.4 mmol, 0.6 eq.) in refluxing anhydrous EtOH (12 mL). The solution was refluxed at this temperature for a further 15 min. Upon cooling to rt, a precipitate formed, which was collected by filtration and washed with cold EtOH (2 × 5 mL) to afford the title compound as a colorless solid (0.49 g, 0.94 mmol, 22%). **M.P.** >350 °C. **^{1}H NMR** (500 MHz, $(CD_3)_2SO$, 353 K) δ 8.17–8.12 (m, 2H, H_5), 8.10 (s, 2H, H_3), 7.89–7.84 (m, 2H, H_6), 7.83–7.78 (m, 4H, H_9), 7.73–7.68 (m, 4H, H_{10}). **^{13}C NMR** (126 MHz, $(CD_3)_2SO$, 353 K), 194.0 (C_7), 184.0 (C_1), 143.5 (C_2), 141.2 (C_3), 135.1 (C_5), 134.9 (C_8), 134.5 (C_4), 131.9 (C_6), 131.4 (C_{10}), 130.4 (C_9), 127.2 (C_{11}). **HR-ASAP-MS** *m/z* = 520.9374 $[M+H]^+$ (calculated for $C_{25}H_{15}O_3Br^{2+}$ = 520.9388).

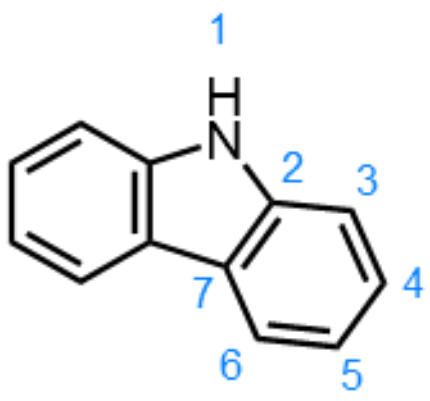

9*H*-Carbazole: An aqueous solution of HCl (2.0 M, 13.5 mL, 27 mmol) was added to 2-aminobiphenyl (2.0 g, 12 mmol), and the mixture was stirred for 10 min. An aqueous solution of $NaNO_2$ (3.0 M, 4.3 mL, 13 mmol) was added dropwise, and the mixture was stirred for 10 min. An aqueous solution of NaN_3 (6.0 M, 2.1 mL, 13 mmol) was added dropwise, and the mixture was stirred for 20 min. CH_2Cl_2 (20 mL) was added, and the layer was separated. The aqueous layer was extracted with CH_2Cl_2 (2 × 20 mL), and the combined organic extracts were washed with brine, then dried over $MgSO_4$. The solvent was removed *in vacuo*, leaving a brown residue. A solution of this residue in *o*-dichlorobenzene (60 mL) was heated to 180 °C for 1 h. Upon cooling to rt, a precipitate formed, which was collected by vacuum filtration and washed with hexanes (3 × 10 mL) to afford the title compound as an off-white solid (1.4 g, 8.1 mmol, 69%). **^{1}H NMR** (400 MHz, $CDCl_3$): 8.14–7.97 (m, 3H), 7.47–38 (m, 4H), 7.29–7.19 (m, 2H). The spectroscopic data was consistent with literature reports.[5]

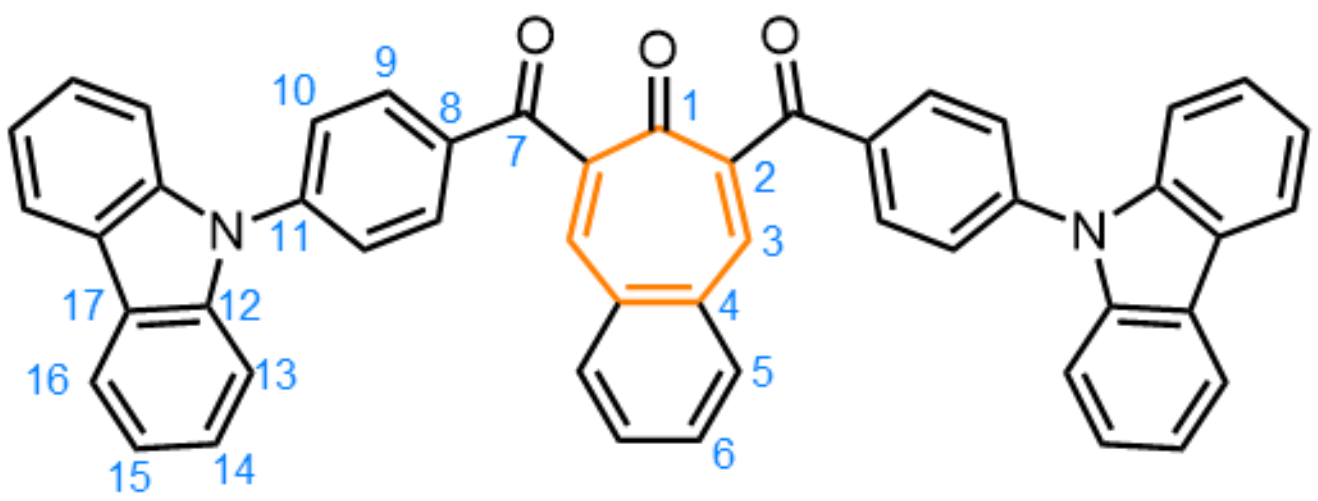

7d

7d: Anhydrous PhMe (5 mL), degassed through 3 × freeze-pump-thaw cycles was added to a flame-dried Schlenk flask containing **7e** (50 mg, 96 μmol, 1 eq.), carbazole (32 mg, 192 μmol, 2 eq.), $Pd_2(dba)_3$ (8.8 mg, 9.6 μmol, 0.1 eq.), XPhos (18 mg, 38 μmol, 0.4 eq.), and Cs_2CO_3 (69 mg, 210 μmol, 2.2 eq.) under an inert atmosphere. The solution was sparged with Ar for 15 min, and the reaction was heated to reflux for 24 h. Upon cooling to rt, the reaction mixture was passed through a pad of SiO_2, eluting in CH_2Cl_2. The solvent was removed under reduced pressure, leaving a brown residue, which was dissolved in CH_2Cl_2 (1 mL) and triturated with

Et_2O (4 mL) to yield the title compound as an off-white solid (50 mg, 72 µmol, 75%). **M.P.** = 203–204 °C. **^{1}H NMR** (600 MHz, $CDCl_3$) δ 8.22–8.17 (m, 4H, H_9), 8.14–8.10 (m, 4H, H_{16}), 7.96–7.90 (m, 4H, $H_{3,5}$), 7.82–7.78 (m, 2H, H_6), 7.73–7.69 (m, 4H, H_{10}), 7.50–7.45 (m, 4H, H_{13}), 7.38–7.32 (m, 4H, H_{14}), 7.32–7.27 (m, 4H, H_{15}). **^{13}C NMR** (151 MHz, $CDCl_3$) δ 194.8 (C_7), 184.8 (C_1), 145.4 (C_2), 142.8 (C_{11}), 141.8 (C_3), 140.2 (C_{12}), 135.6 (C_5), 135.2 (C_4), 134.6 (C_8), 132.3 (C_6), 131.5 (C_9), 126.6 (C_{10}), 126.4 (C_{14}), 124.0 (C_{17}), 120.8 (C_{15}), 120.5 (C_{16}), 110.0 (C_{13}). **HR-ASAP-MS** *m/z* = 695.2356 [M+H$^+$] (calculated for $C_{49}H_{31}N_2O_3^+$ = 695.2335).

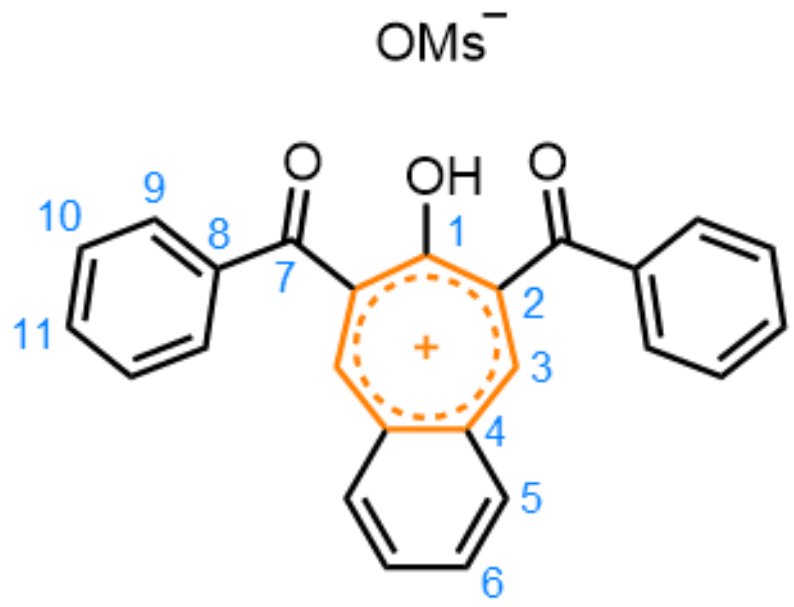

4b

4b: MsOH (32 µL) was added to a solution of **7b** (3.5 mg, 9.6 µmol) in anhydrous $CDCl_3$ (0.5 mL) in an oven-dried NMR tube under an inert atmosphere at rt. NMR spectroscopic analysis indicated quantitative conversion of the starting material to **4b**. **^{1}H NMR** (400 MHz, $CDCl_3$) δ 9.22 (s, 2H, H_3), 8.49–8.43 (m, 2H, H_5), 8.39–8.34 (m, 2H, H_6), 7.97–7.92 (m, 4H, H_9), 7.80–7.74 (m, 2H, H_{11}), 7.63–7.55 (m, 4H, H_{10}). **^{13}C NMR** (100 MHz, $CDCl_3$) δ198.5 (C_7), 186.2 (C_1), 155.9 (C_3), 138.9 (C_5), 137.4 (C_6), 136.9 ($C_{2/4}$), 135.9 ($C_{4/2}$), 135.8 (C_{11}), 134.6 (C_8), 130.9 (C_9), 129.6 (C_{10}).

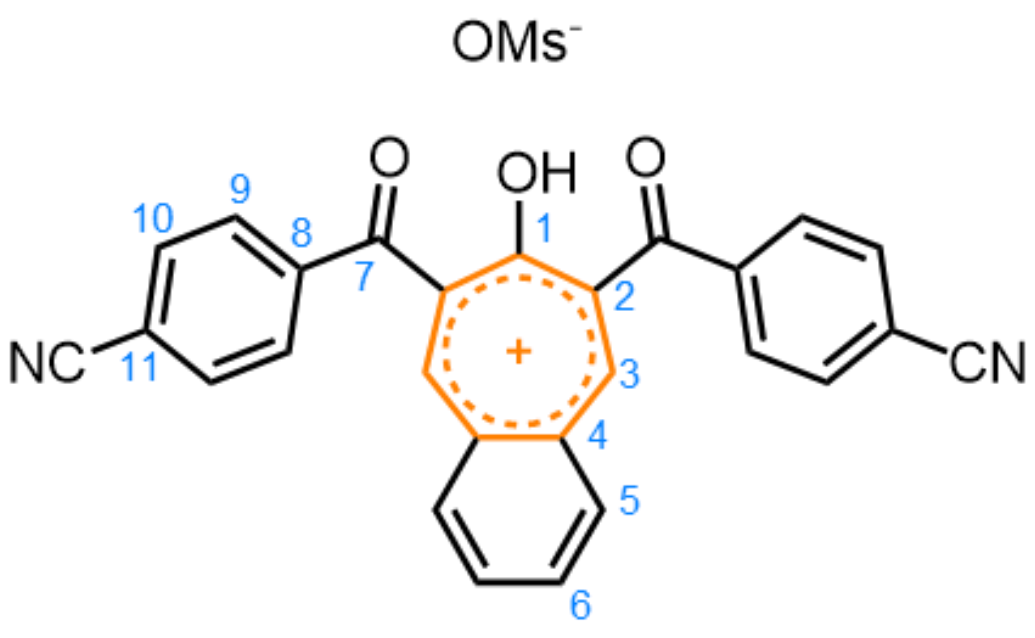

4c

4d: Due to its low solubility, we could not characterize **4d** by ^{1}H NMR spectroscopy. We limit our characterization of this species to optical spectroscopic studies (see section 5, pg. S32)

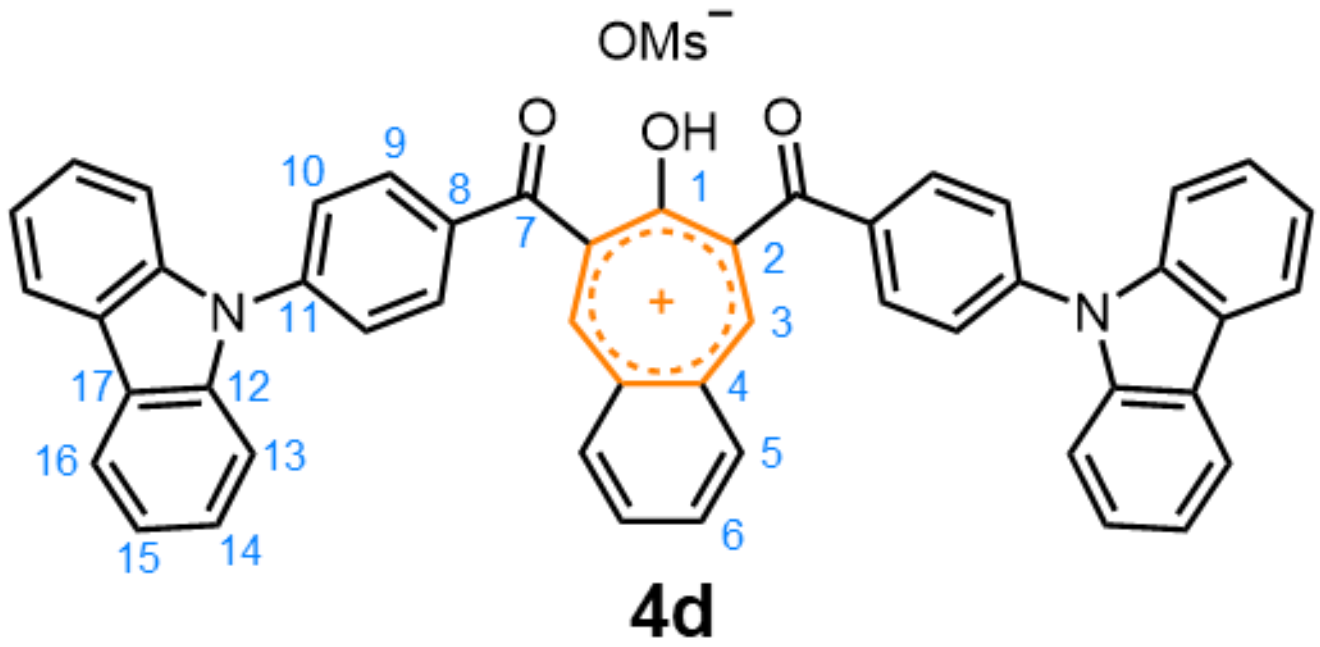

4d

4d: MsOH (32 µL) was added to a solution of **7d** (5.0 mg, 9.6 µmol) in anhydrous $CDCl_3$ (0.5 mL) in an oven-dried NMR tube under an inert atmosphere at rt. NMR spectroscopic analysis indicated quantitative conversion of the starting material to **4d**. **^{1}H NMR** (400 MHz, $CDCl_3$) δ 9.34 (s, 2H, H_3), 8.68–8.54 (m, 2H, H_5), 8.52–8.41 (m, 2H, H_6), 8.28 (d, *J* = 8.4 Hz, 4H, H_9), 8.15 (d, *J* = 7.7 Hz, 4H, H_{16}), 7.94 (d, *J* = 8.3 Hz, 4H, H_{10}), 7.65 (d, *J* = 8.2 Ha, 4H, H_{13}), 7.53–7.41 (m, 4H, H_{14}), 7.39–7.30 (m, 4H, H_{15}). **^{13}C NMR** (100 MHz, $CDCl_3$) δ 197.5 (C_7), 186.6 (C_1), 157.0 (C_3), 145.2 (C_2), 139.8 (C_{12}), 139.4 (C_{11}), 138.0 (C_5), 137.2 (C_6), 133.4 (C_9), 132.2 (C_8), 126.8 (C_{10}), 126.7 (C_{14}), 124.4 (C_{17}), 121.5 (C_{15}), 120.8 (C_{16}), 110.2 (C_{13}

3. ^{1}H and ^{13}C NMR Spectroscopic Characterization of Synthesized Compounds

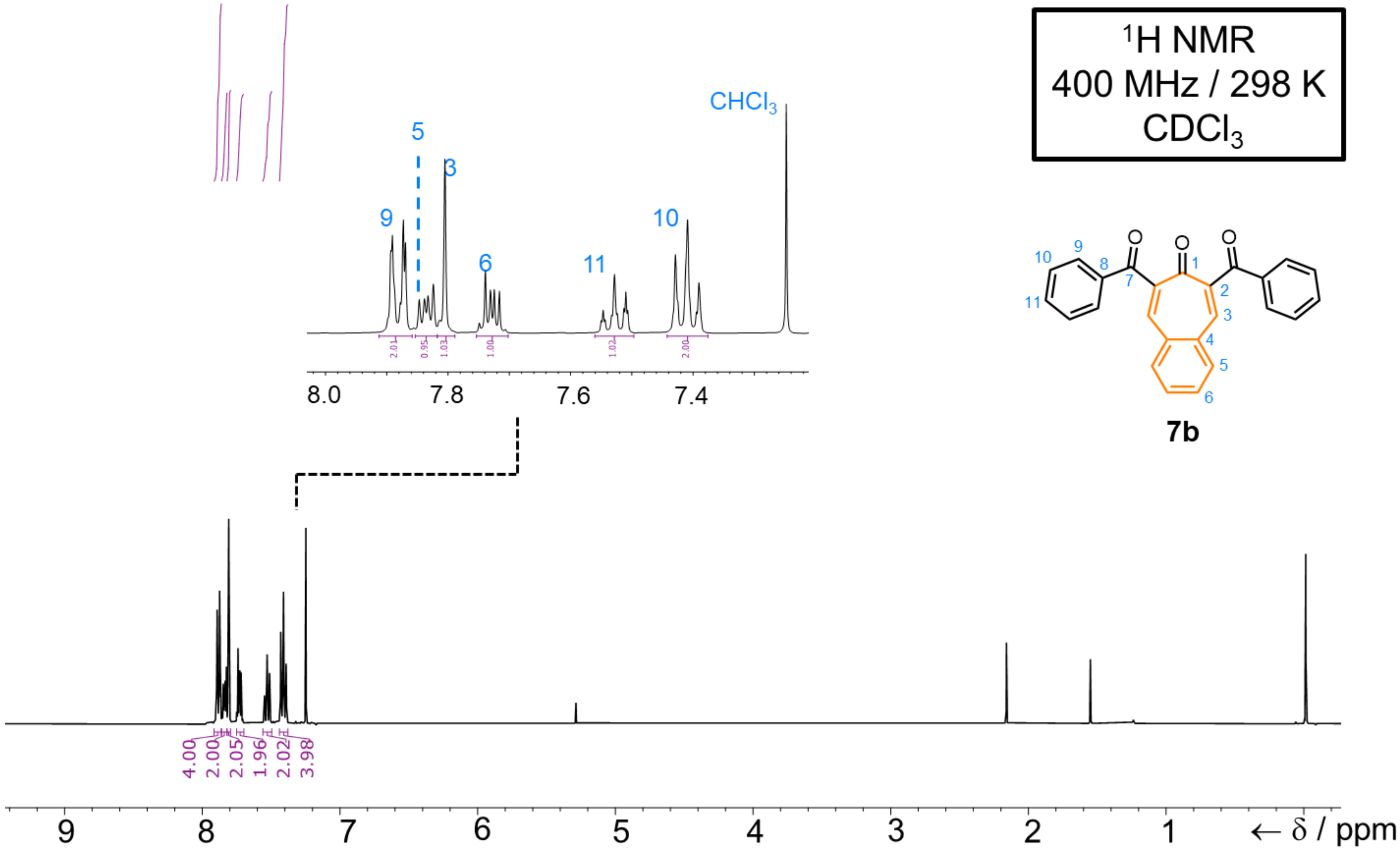

Fig. S2. ^{1}H NMR spectrum of **7b.**

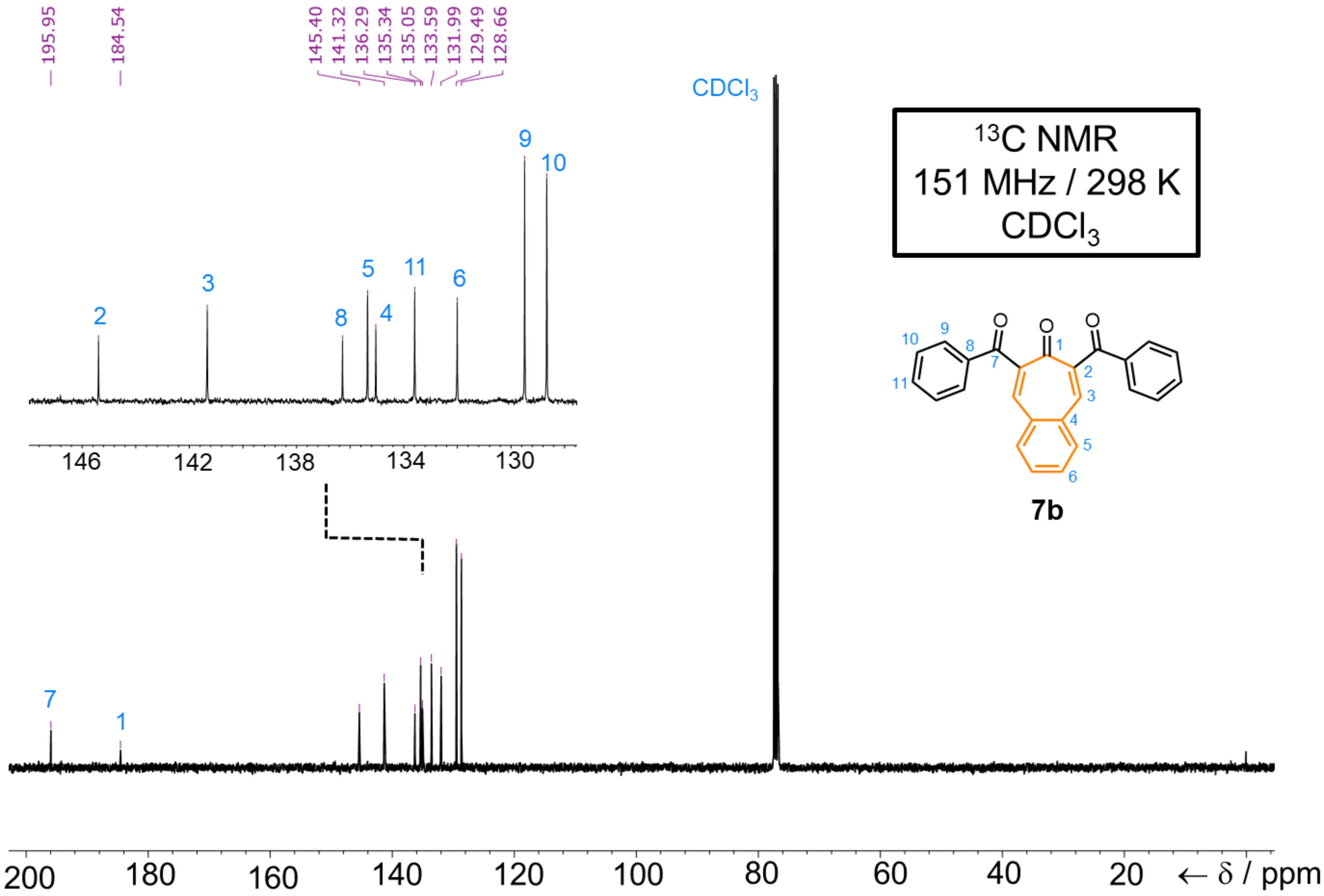

Fig. S3. ^{13}C NMR spectrum of **7b.**

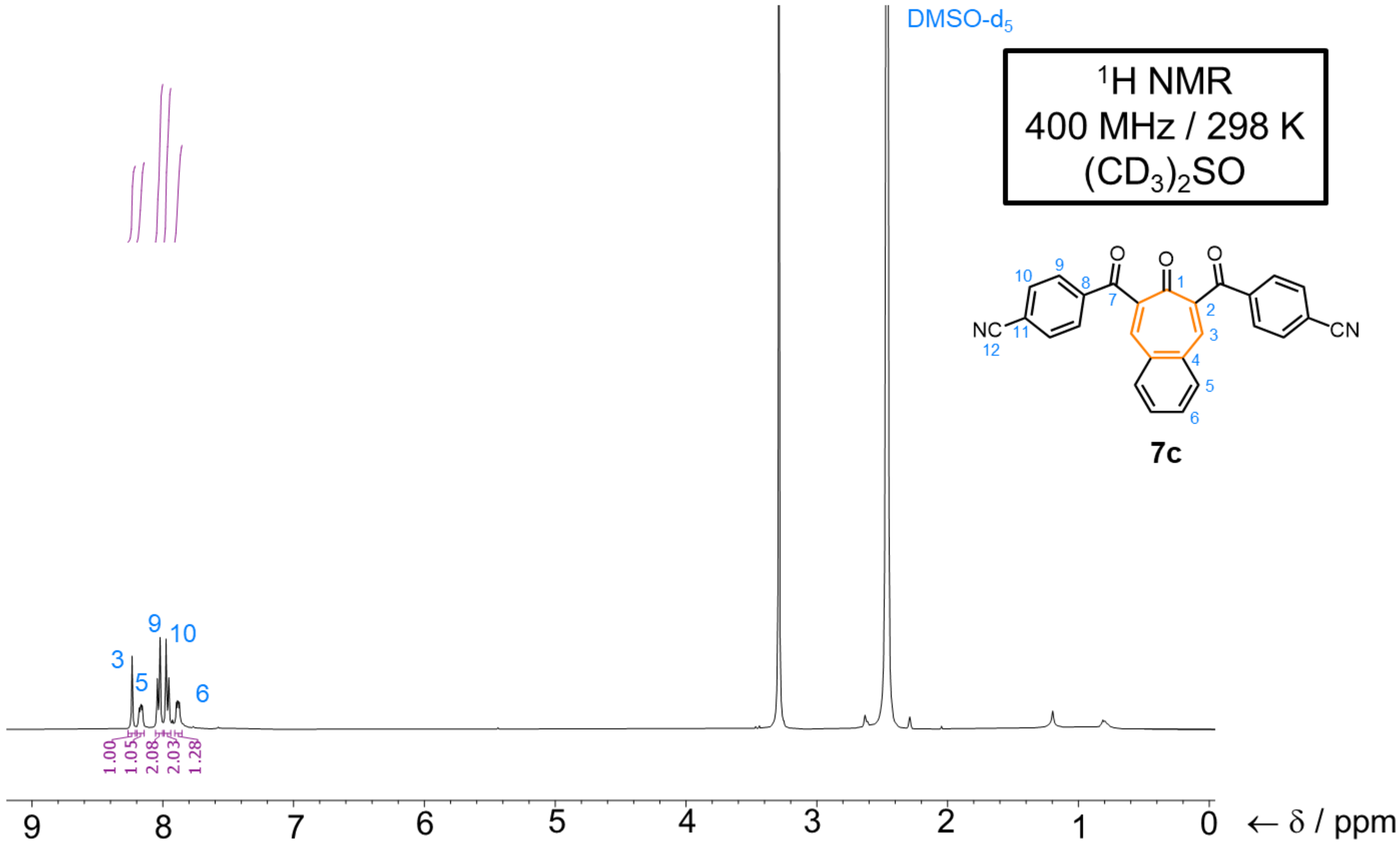

Fig. S4. ¹H NMR spectrum of **7c**.

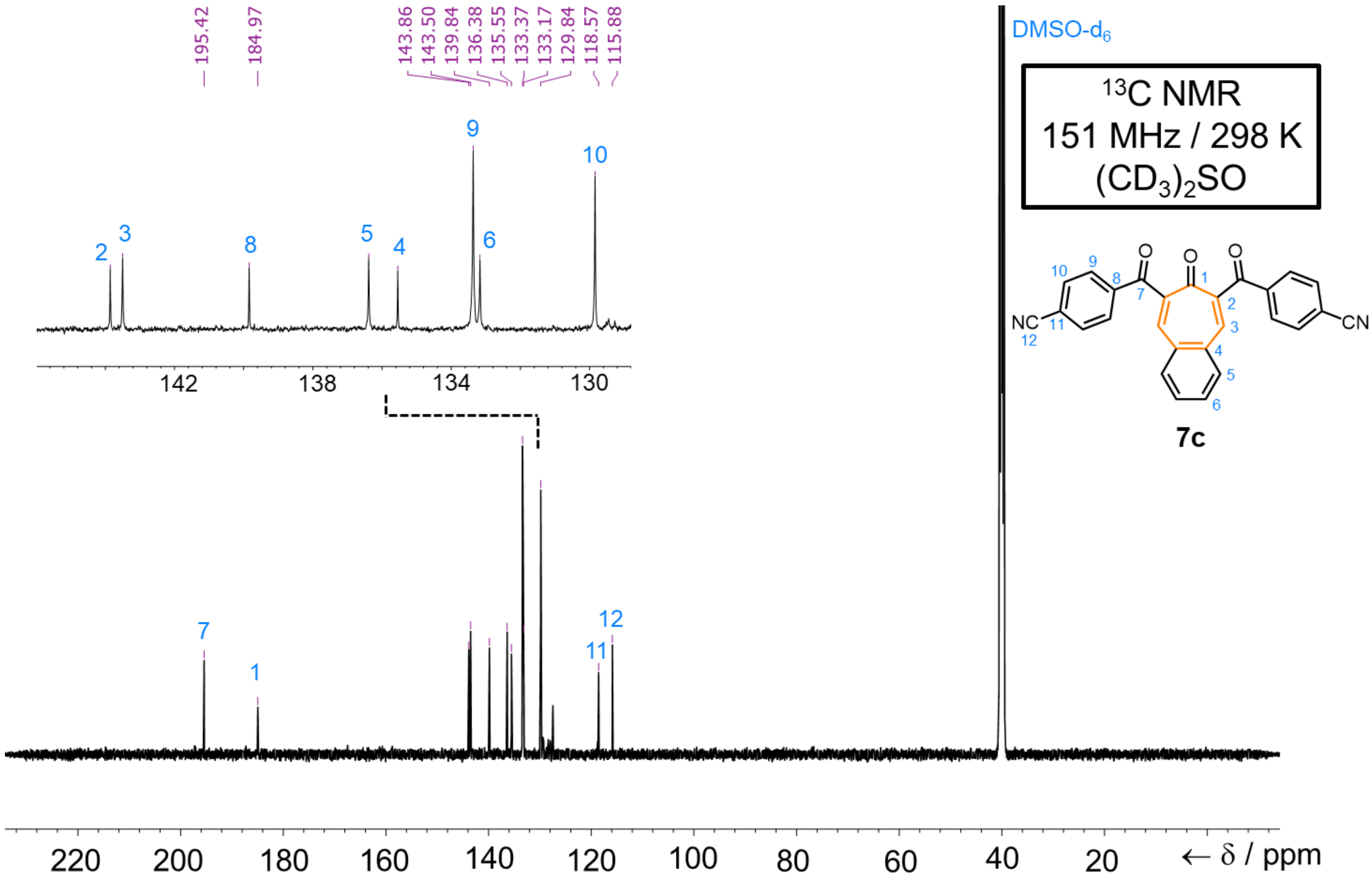

Fig. S5. ^{13}C NMR spectrum of **7c.**

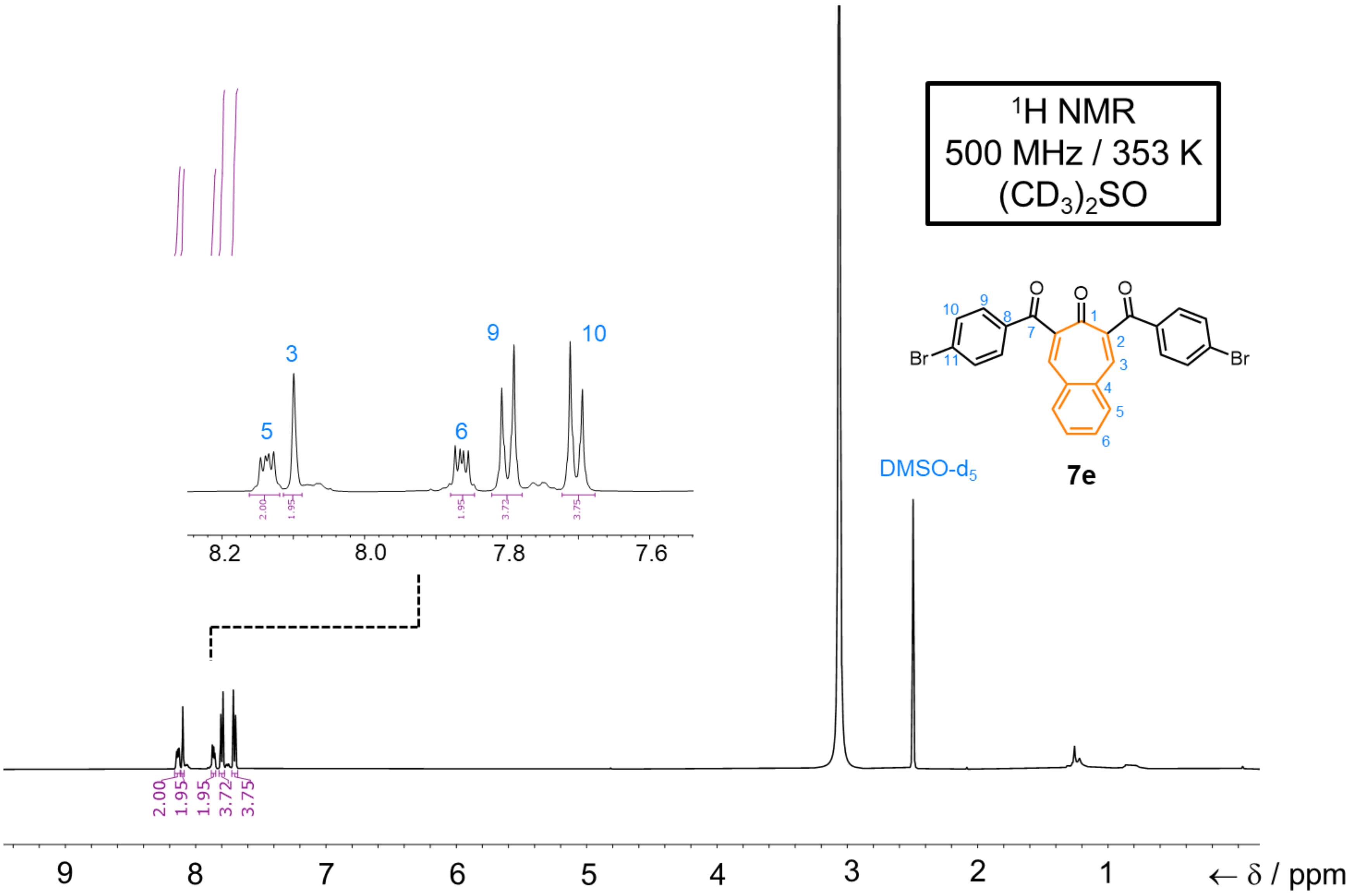

Fig. S6. ^{1}H NMR spectrum of **7e**.

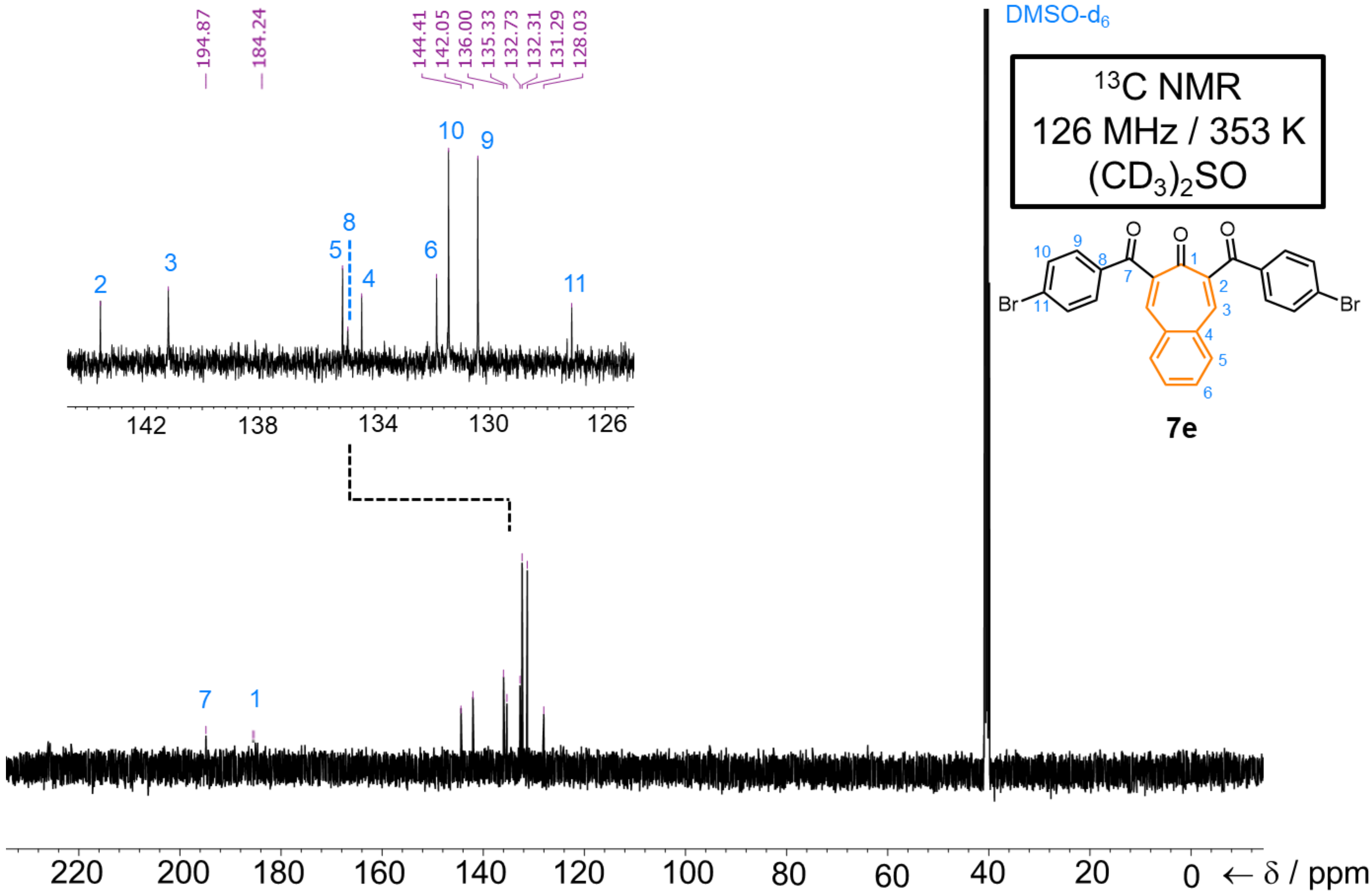

Fig. S7. ^{13}C NMR spectrum of **7e.**

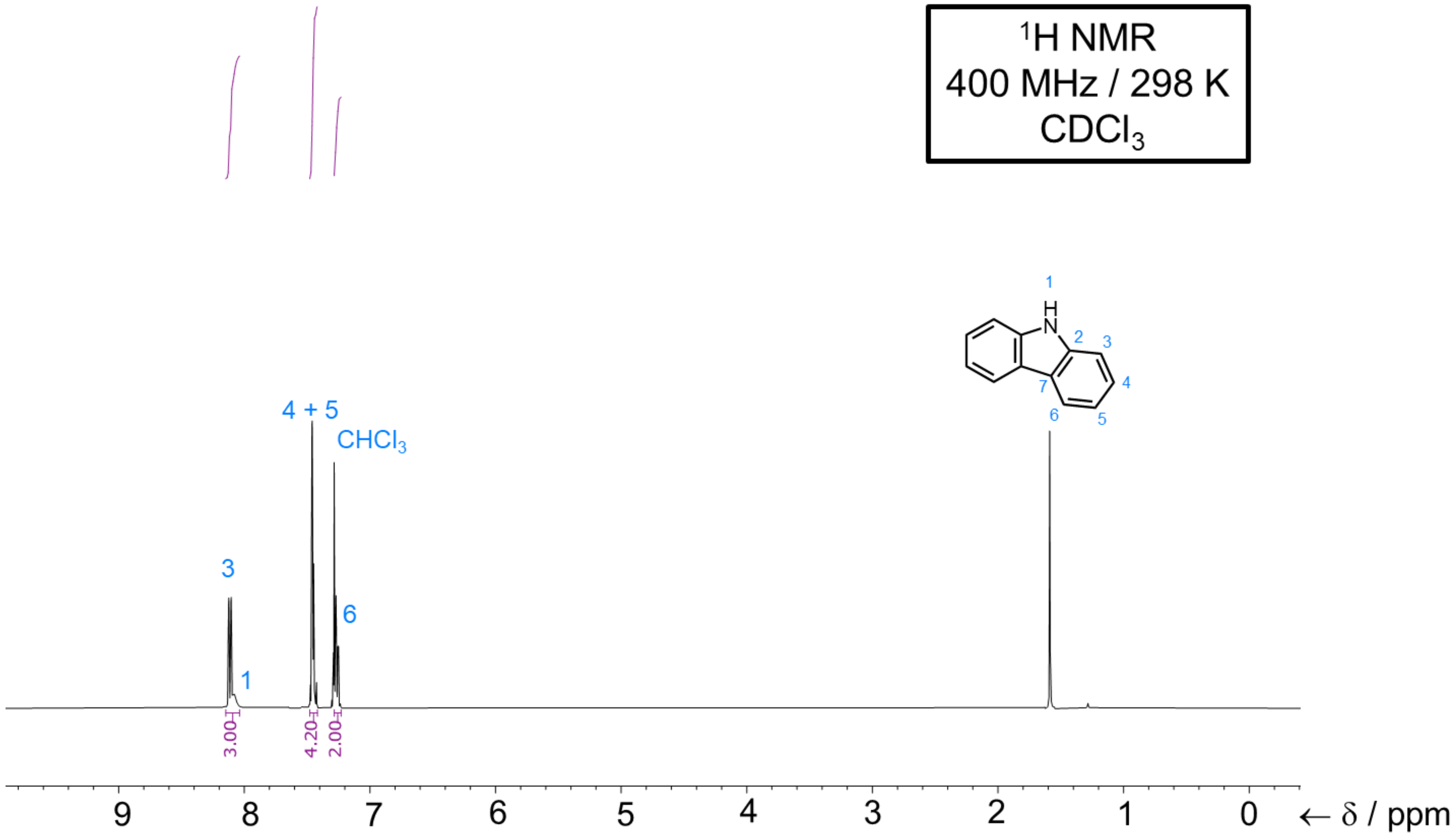

Fig. S8. ^{1}H NMR spectrum of **9*H*-Carbazole.**

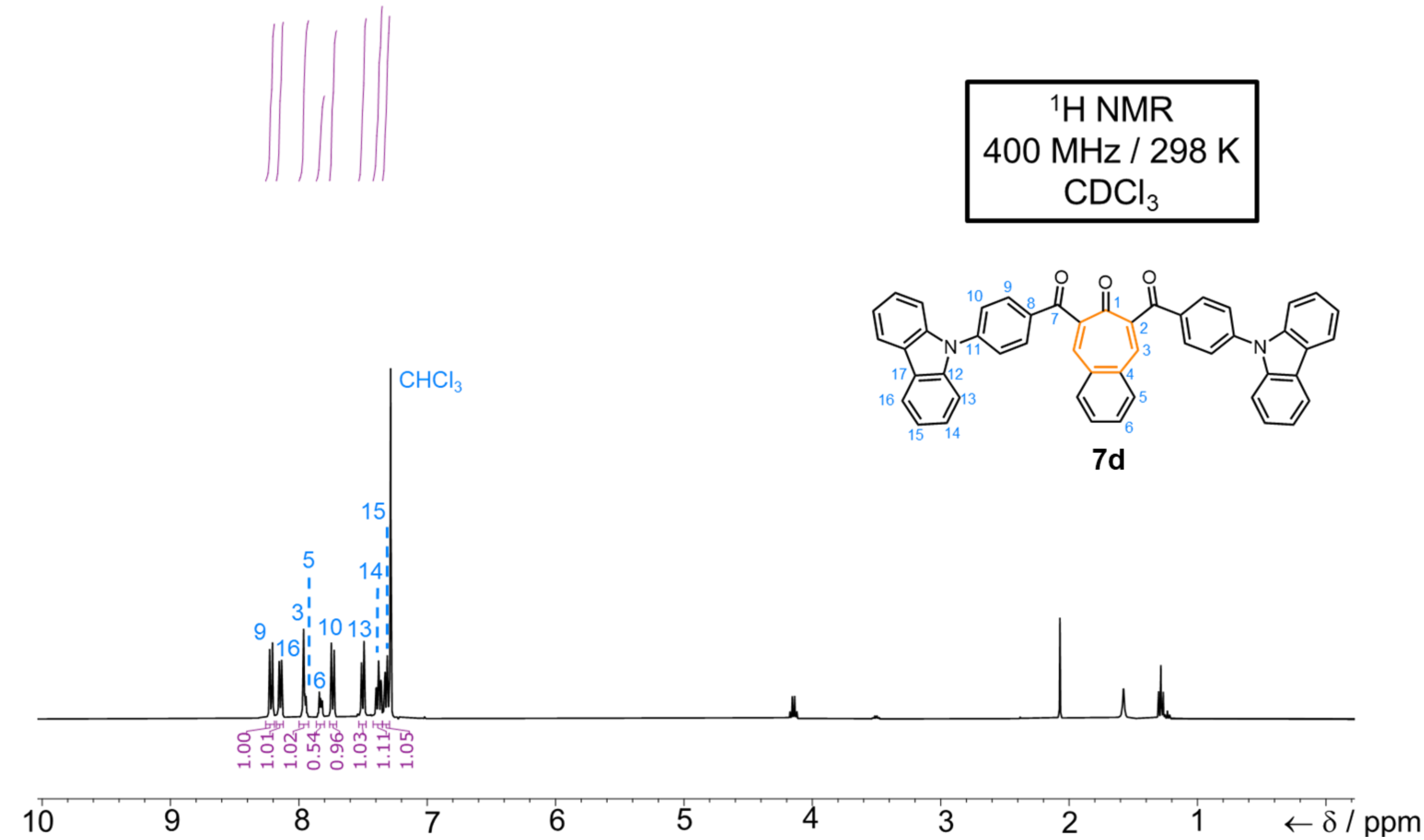

Fig. S9. ^{1}H NMR spectrum of **7d**.

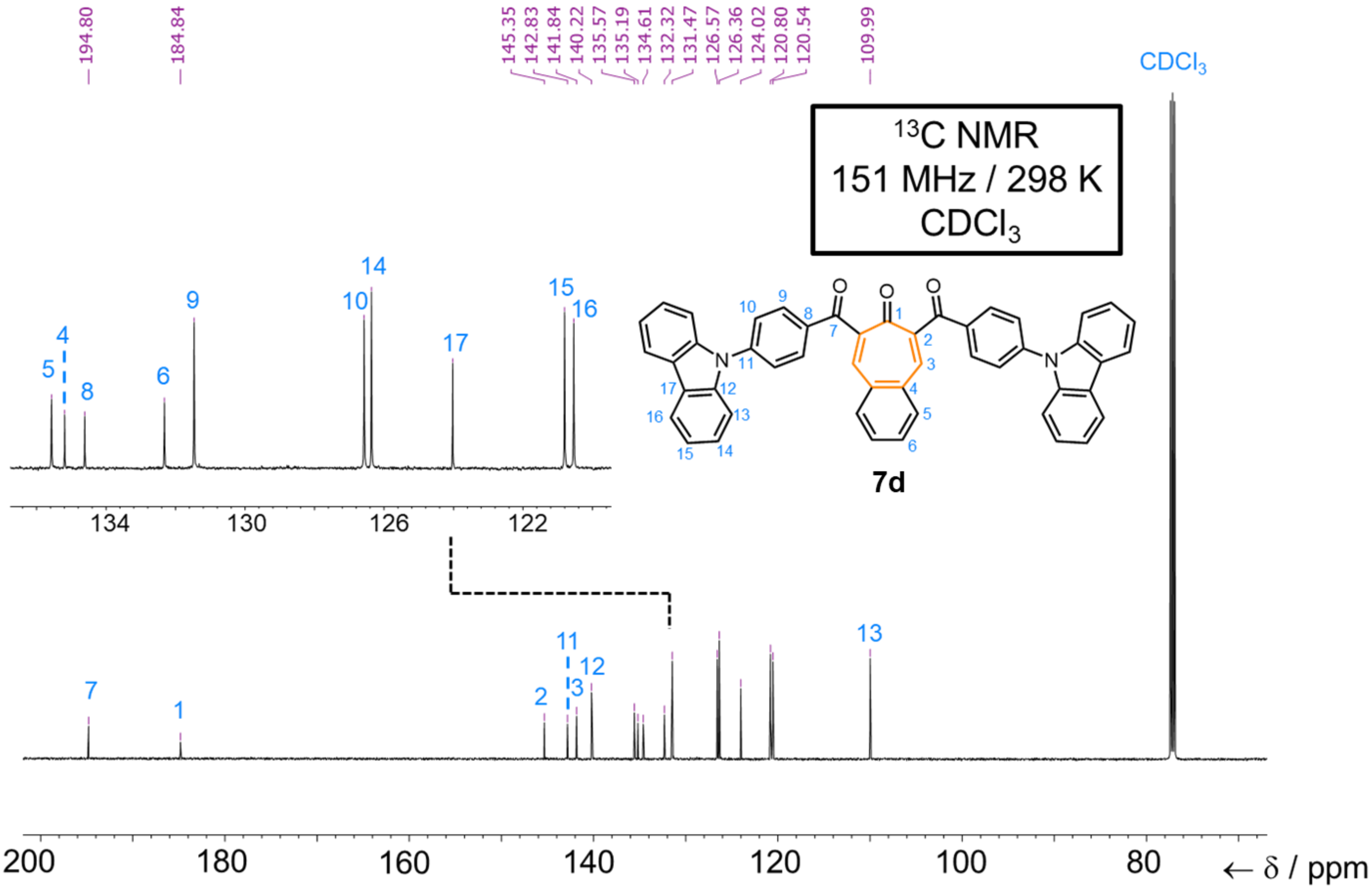

Fig. S10. ^{13}C NMR spectrum of **7d.**

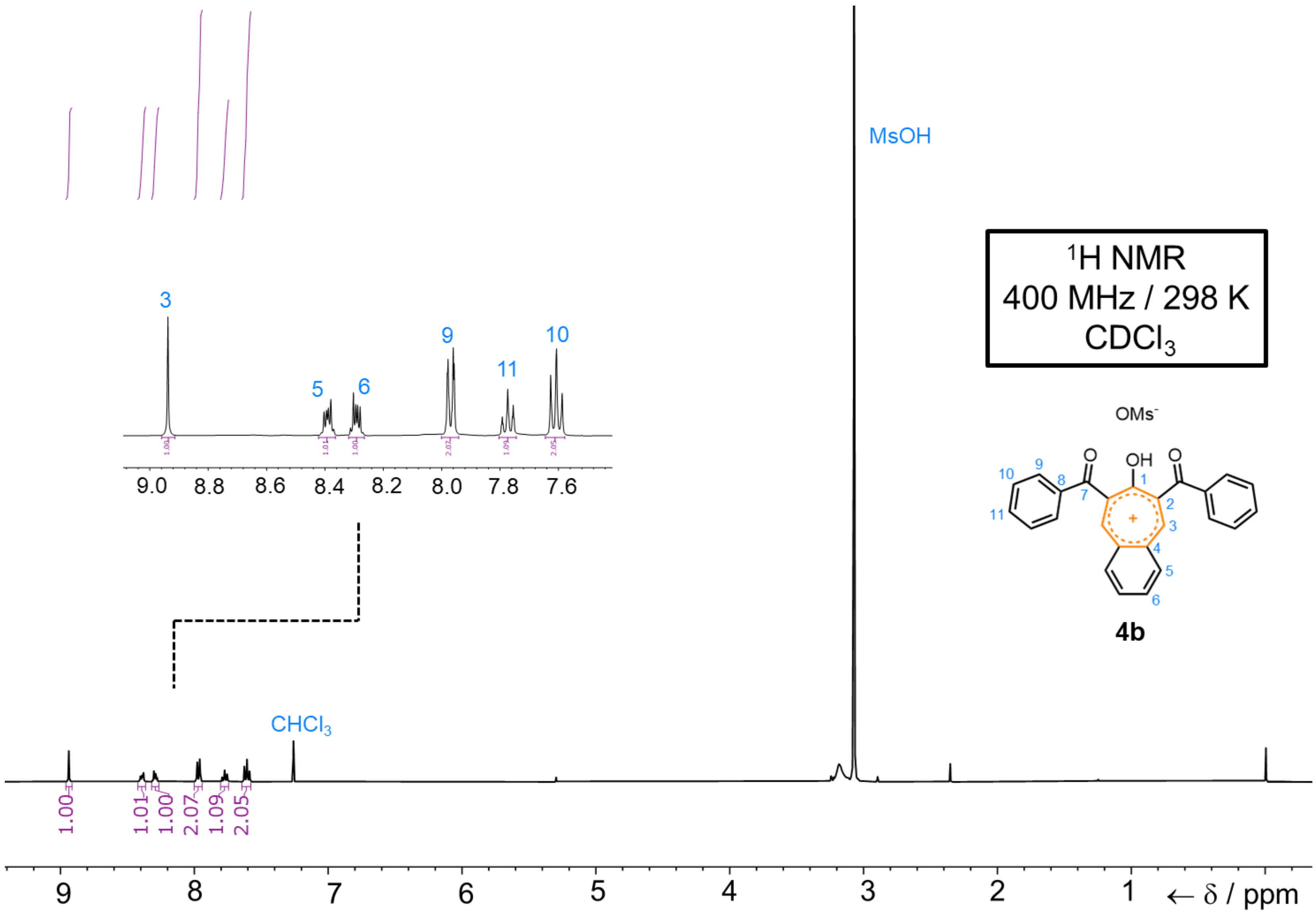

Fig. S11. ^{1}H NMR spectrum of **4b**·OMs**.**

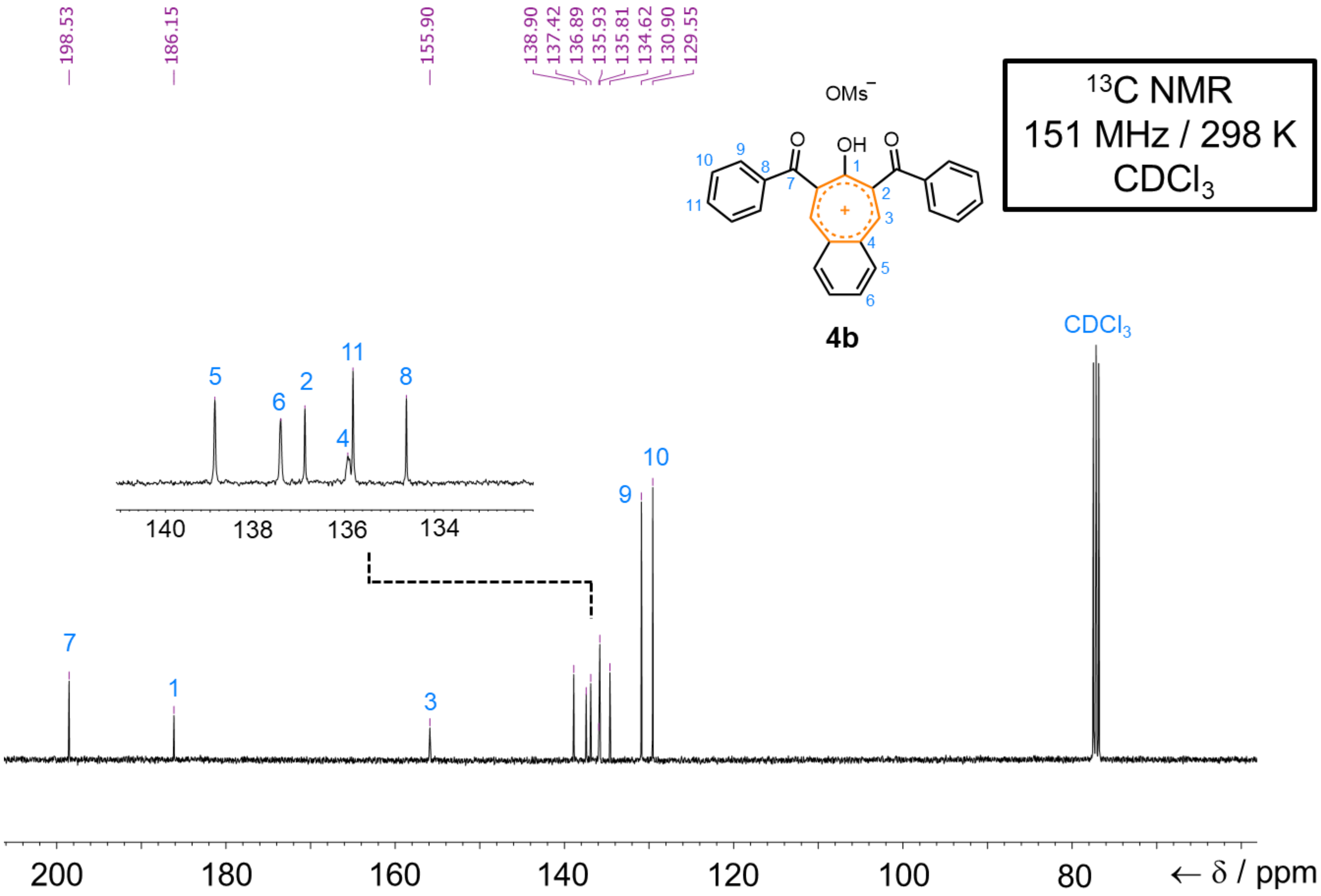

Fig. S12. ^{13}C NMR spectrum of **4b**·OMs**.**

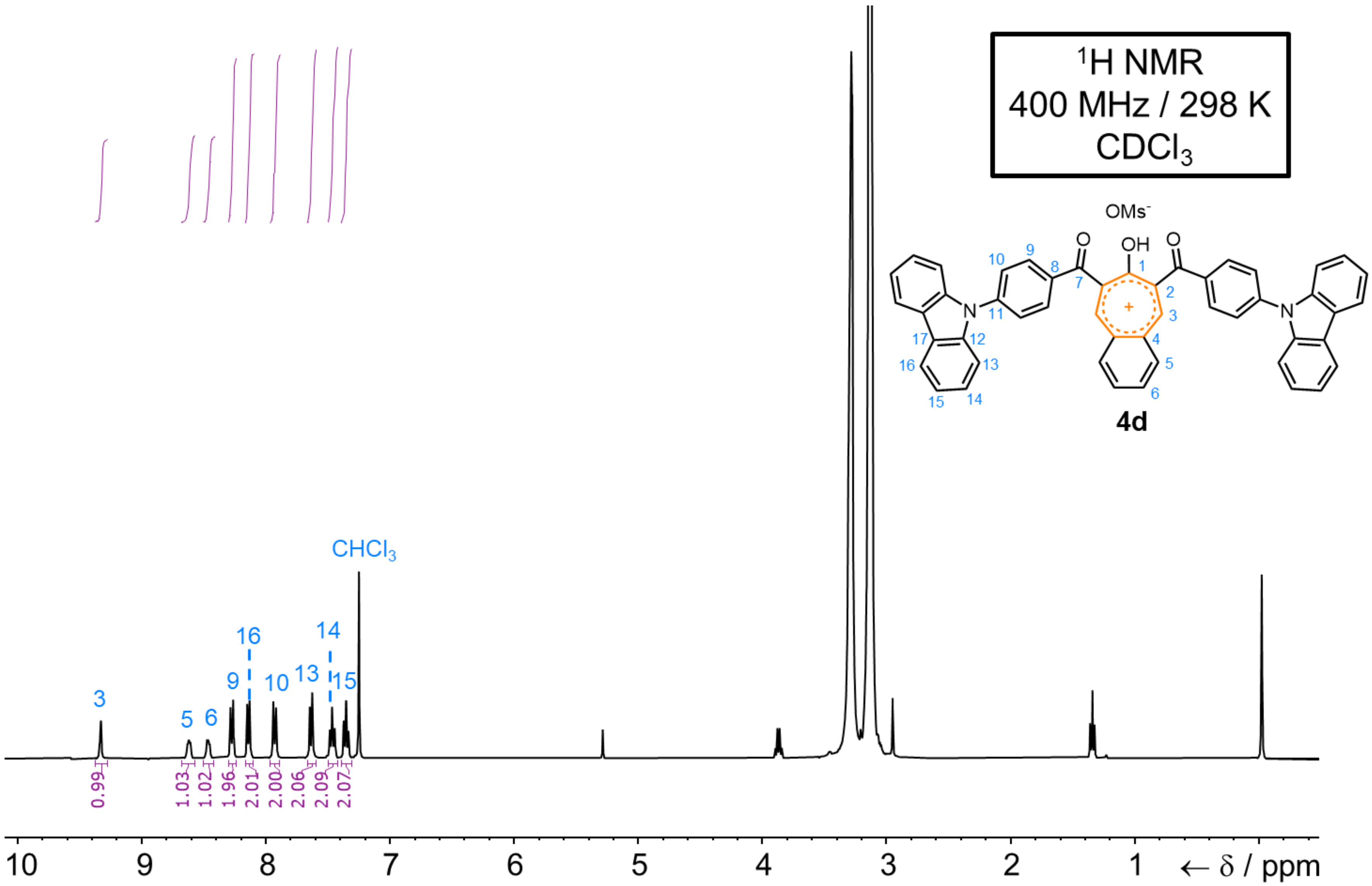

Fig. S13. ^{1}H NMR spectrum of **4d**·OMs**.**

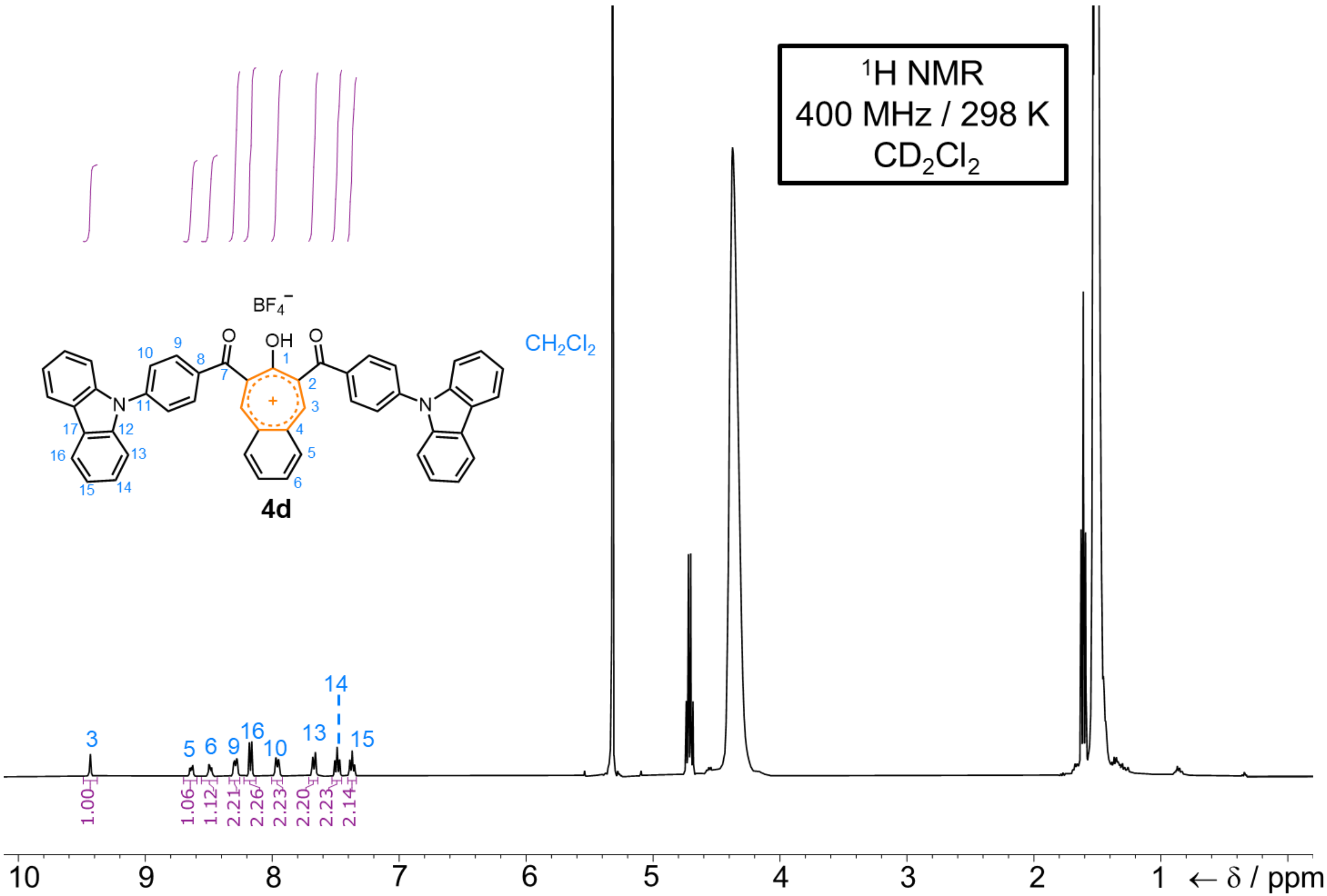

Fig. S14. ^{1}H NMR spectrum of **4d**·BF_4.

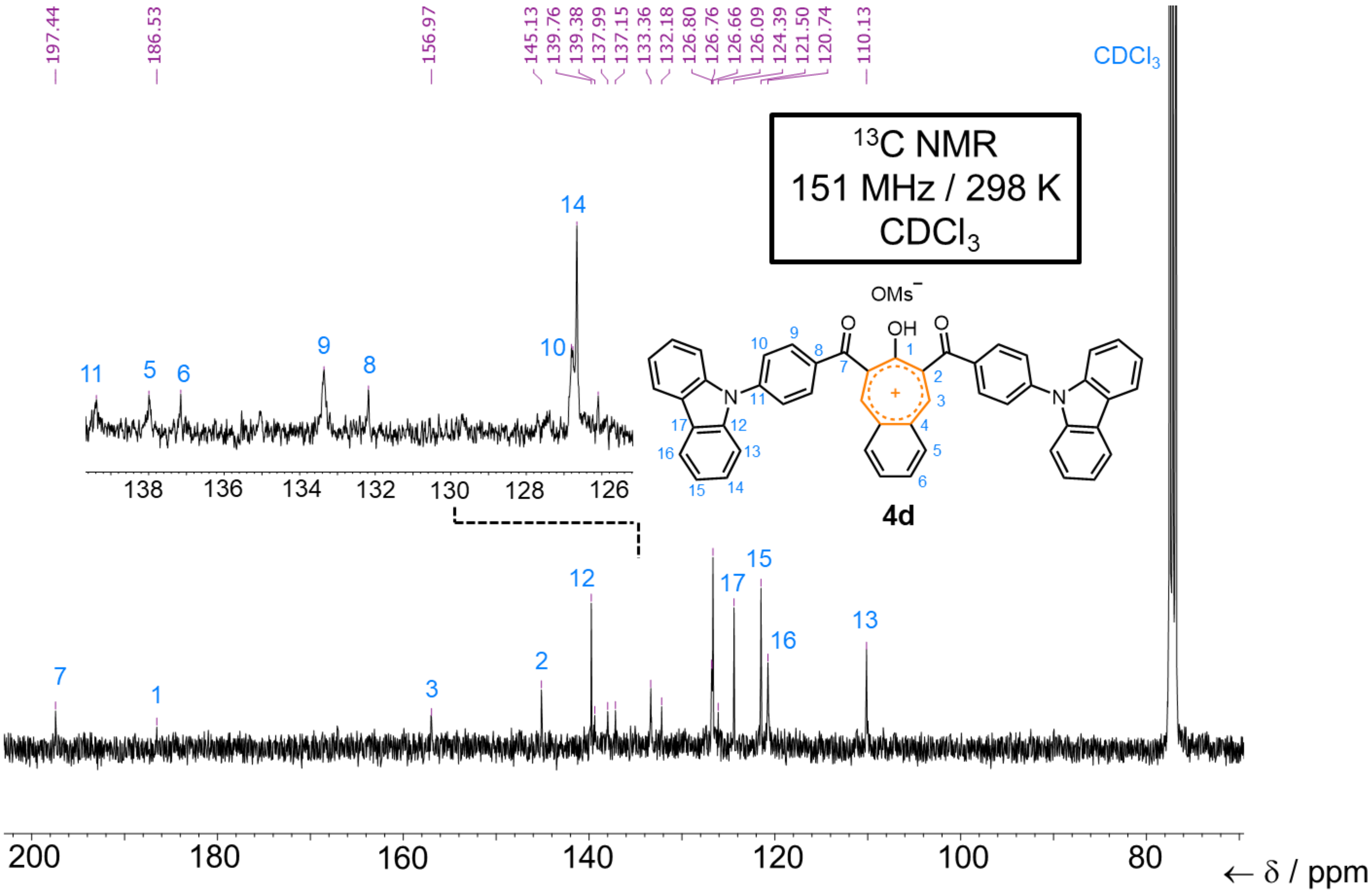

Fig. S15. ^{13}C NMR spectrum of **4d**·OMs.

3.1. pK_a Determination by NMR Spectroscopy

The pK_as of **4b** and **4d** were determined by a ^{1}H NMR titration (anhydrous $CDCl_3$, 298 K), where the change in chemical shifts of the α-proton (H_3) (as shown in Figures S16–S17) were measured at different concentrations of acid ([MsOH] with **4b**, [$HBF_4·Et_2O$] with **4d**). We found MsOH to be unsuitable for a titration with **4d**, as it led to broad signals. The pK_a of **4c** by ^{1}H NMR titration was not determined due to poor solubility. The data points were fitted to a dose-response sigmoidal curve **Equation S1** (below):[6]

$$\Delta\delta = \delta_i + \frac{\delta_i + \delta_f}{1 + 10^{\log(\mathrm{p}K_\mathrm{a} - \mathrm{pH})\cdot p}}$$

Equation S1. Where the chemical shift difference ($\Delta\delta$ / ppm) was determined as a function of the initial and final chemical shifts ($\Delta\delta_{i,f}$ / ppm respectively) and p is the sensitivity factor.

The pK_a corresponds to the inflection point of the fitted curve. During the titration, a solution of **7** (20 mM) and MsOH (2.0 M) in $CDCl_3$ was added in portions to a solution of **7** (20 mM) in $CDCl_3$. Maintaining the same initial concentration of the substrate [**7**] in both solutions ensures dilution effects are absent, so do not need to be included in the analysis.

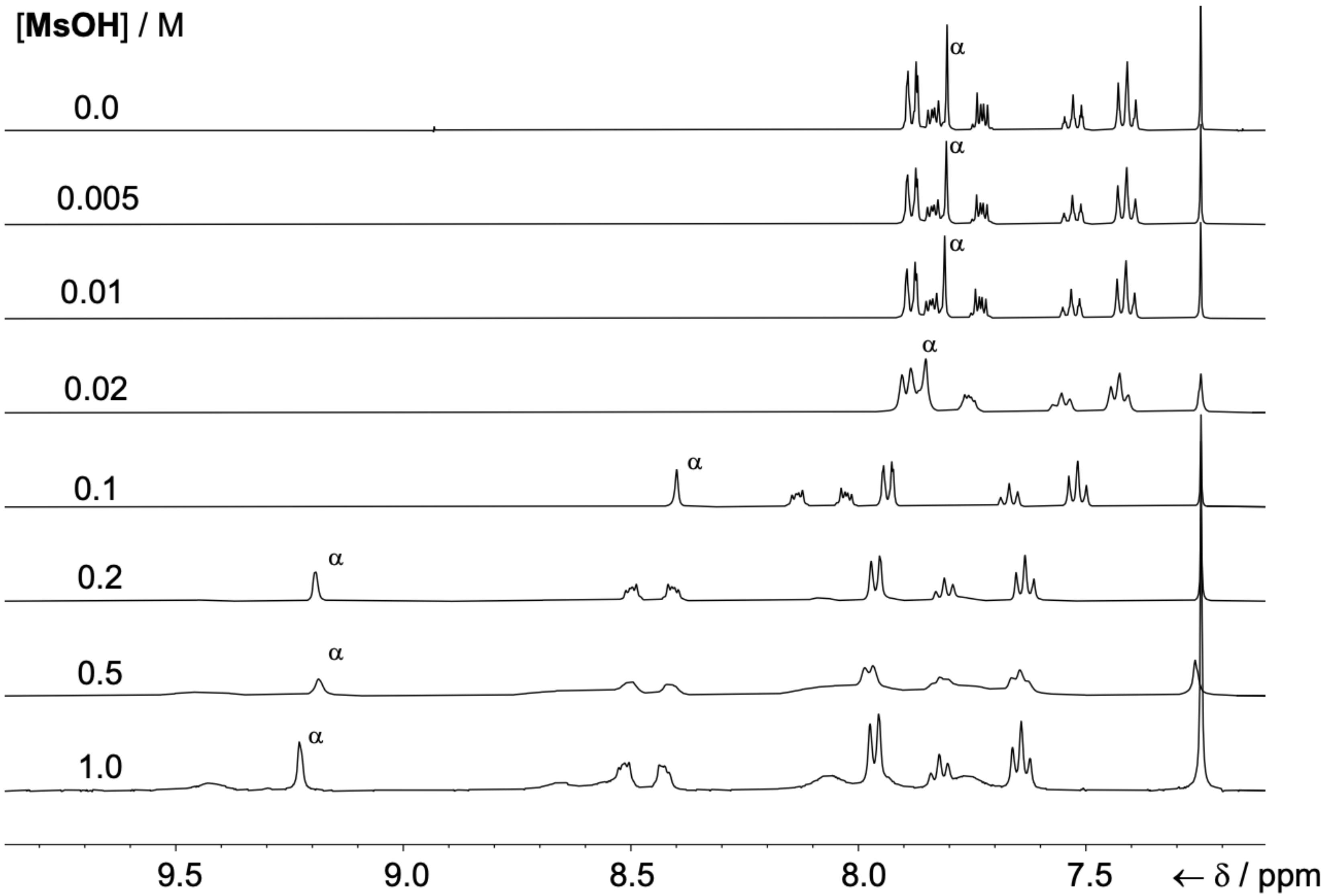

Fig. S16. Partial ^{1}H NMR spectra (400 MHz, $CDCl_3$, 298 K) of **7b** (α-proton environment labelled, see Figure 3 of the main text) in the presence of increasing concentrations of MsOH.

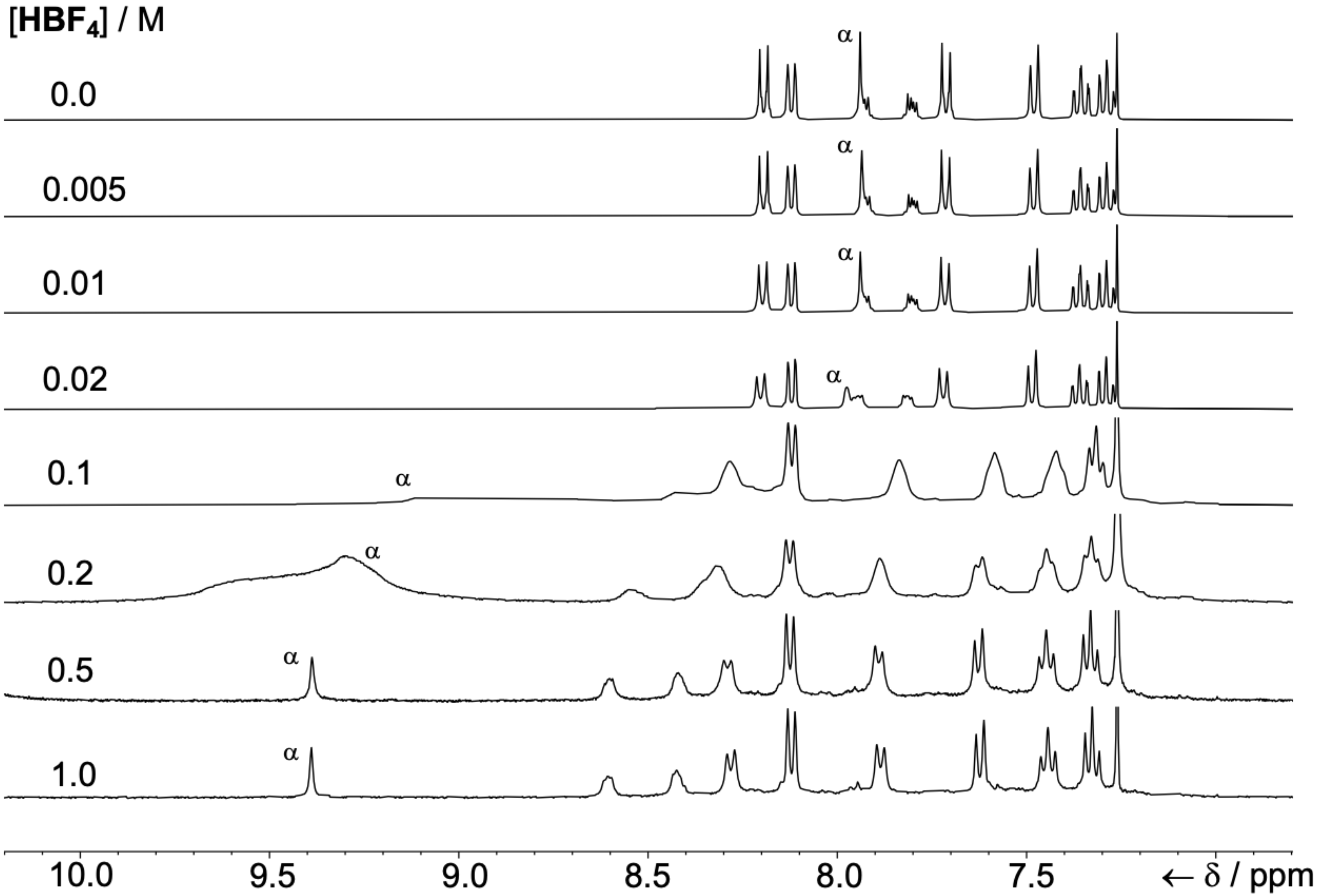

Fig. S17. Partial ^{1}H NMR spectra (400 MHz, $CDCl_3$, 298 K) of **7d** (α-proton environment labelled, see Figure 3 of the main text) in the presence of increasing concentrations of $HBF_4{\cdot}Et_2O$.

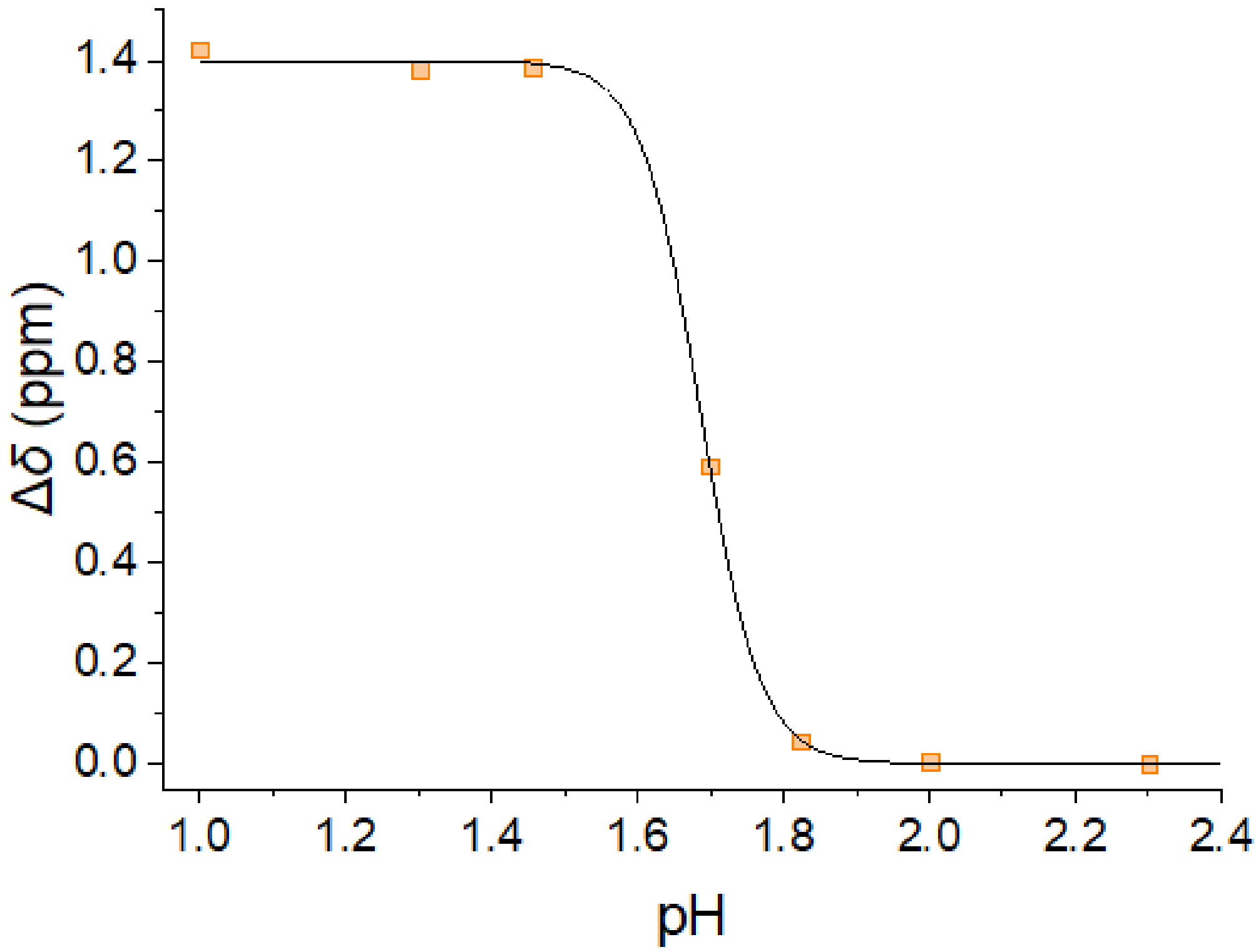

Fig. S18. Variation of the chemical shift of **7b** at different pH values where R^2 = 0.99953 using **Equation S1** to yield a pK_a of 1.69±0.02 for **4b**. The line of best fit corresponds to a one-step protonation equilibrium.

Table S1. Variation in chemical shift ($\Delta\delta$) of **7b** at different concentrations of MsOH.

[MsOH]	pH	δ	Δδ
0	2.50	7.805	0.000
0.005	2.30	7.807	0.002
0.01	2.00	7.810	0.005
0.02	1.82	7.851	0.046
0.10	1.70	8.399	0.594
0.20	1.46	9.192	1.387
0.50	1.30	9.189	1.383
1.00	1.00	9.228	1.423

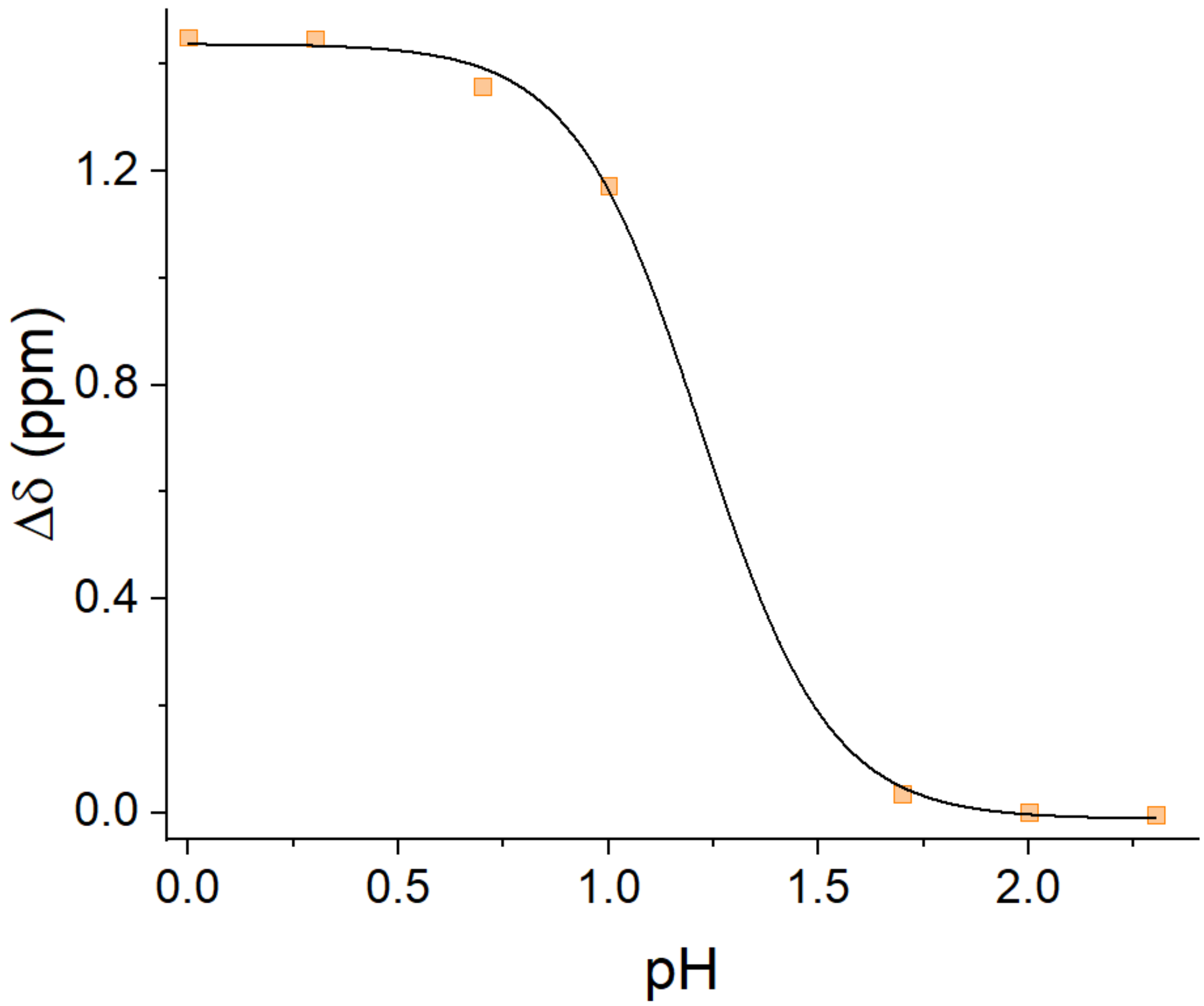

Fig. S19. Variation of the chemical shift of **7d** at different pH values where R^2 = 0.99884 using **Equation S1** to yield a pK_a of 1.22±0.03 for **4d**. The line of best fit corresponds to a one-step protonation equilibrium.

Table S2. Variation in chemical shift (Δ*δ*) of **7d** at different concentrations of $HBF_4{\cdot}Et_2O$

**[$HBF_4{\cdot}Et_2O$]**	**pH**	**δ**	**Δδ**
0.005	2.30	7.934	-0.004
0.01	2.00	7.938	0.000
0.02	1.70	7.974	0.046
0.10	1.00	9.111	1.173
0.20	0.70	9.296	1.358
0.50	0.30	9.386	1.448
1.00	0.00	9.388	1.450

4. X-ray Crystallographic Analysis

7d

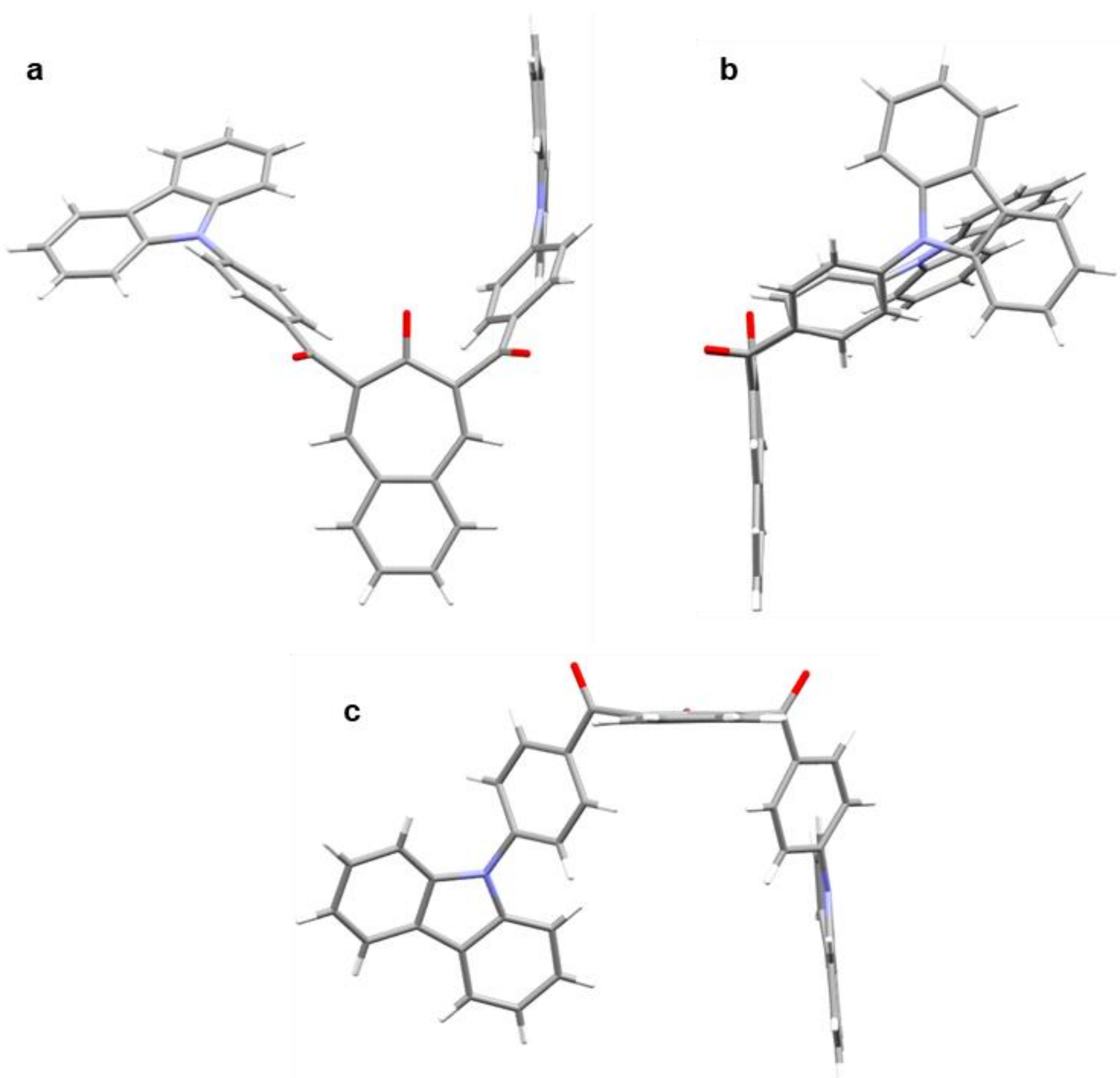

Fig. S20. Single-crystal XRD structure of **7d** viewed **(a)** face-on, **(b)** side-on, and **(c)** from the bottom of the benzotropone moiety

Table S3. Crystal data and structure refinement for **7d**.

	**7d**
Empirical formula	$C_{49}H_{30}N_2O_3$
Formula weight	694.75
Temperature/K	120.00
Crystal system	monoclinic
Space group	$P2_1/n$
a/Å	16.2592(14)
b/Å	9.2374(8)
c/Å	25.157(2)
α/°	90
β/°	92.693(3)
γ/°	90
Volume/Å^3	3774.3(6)
Z	4
ρ_{calc}g/cm^3	1.223
μ/mm^{-1}	0.076
F(000)	1448.0
Crystal size/mm^3	0.31 × 0.06 × 0.01
Radiation	Mo Kα (λ = 0.71073)
2Θ range for data collection/°	4.698 to 51.996
Index ranges	-20 ≤ h ≤ 20, -11 ≤ k ≤ 11, -31 ≤ l ≤ 31
Reflections collected	88594
Independent reflections	7416 [R_{int} = 0.1350, R_{sigma} = 0.0736]
Data/restraints/parameters	7416/0/488
Goodness-of-fit on F^2	1.066
Final R indexes [I>=2σ (I)]	R_1 = 0.0754, wR_2 = 0.1458
Final R indexes [all data]	R_1 = 0.1086, wR_2 = 0.1587
Largest diff. peak/hole / e Å^{-3}	0.26/-0.27

Crystals of **7d** suitable for X-ray diffraction were grown by slow evaporation of a saturated CH_2Cl_2 solution.

5. UV-Vis Absorption Spectroscopy

UV-vis measurements were performed in anhydrous CH_2Cl_2. Spectra were measured in 10 mm path-length cuvette at room temperature. **4b**, **4c** and **4d** (purple line) were prepared *in situ* from their corresponding precursors: **7b**, **7c** and **7d** (orange line), respectively. The samples were sparged briefly with $N_{2\,(g)}$. The intensities were plotted as molar absorptivity ε defined by the formula:

$$\varepsilon = \frac{I}{c \cdot l}$$

Equation S2. Molar absorptivity (ε) where *I* is the measured intensity, *c* is the concentration of sample, and *l* is path length of the cuvette.

Samples for UV-vis absorption spectroscopy were prepared using 1 mg·mL^{-1} CH_2Cl_2 stock solutions. The desired quantity was diluted to achieve the desired 10 µM concentration. The diluted solution was titrated with a solution mixture of MsOH (2.0 M) and **7b**, **7c** or **7d** (10 µM). After each addition of the acid, the UV-vis spectra were recorded. The data points were fitted to the dose-response function (**Equation S3** below) to determine the pK_a:

$$I = I_i + \frac{I_i + I_f}{1 + 10^{\log\,(\mathrm{p}K_\mathrm{a} - \mathrm{pH}) \cdot p}}$$

Equation S3. Where the absorption/emission intensity (*I*) was determined as a function of the initial and final absorption intensity (λ_{max} = 305 nm) or emission intensity (λ_{max} = 425 nm) ($I_{i,f}$ respectively) and *p* is the sensitivity factor. [**7**] was kept constant at 10 µM for this experiment.

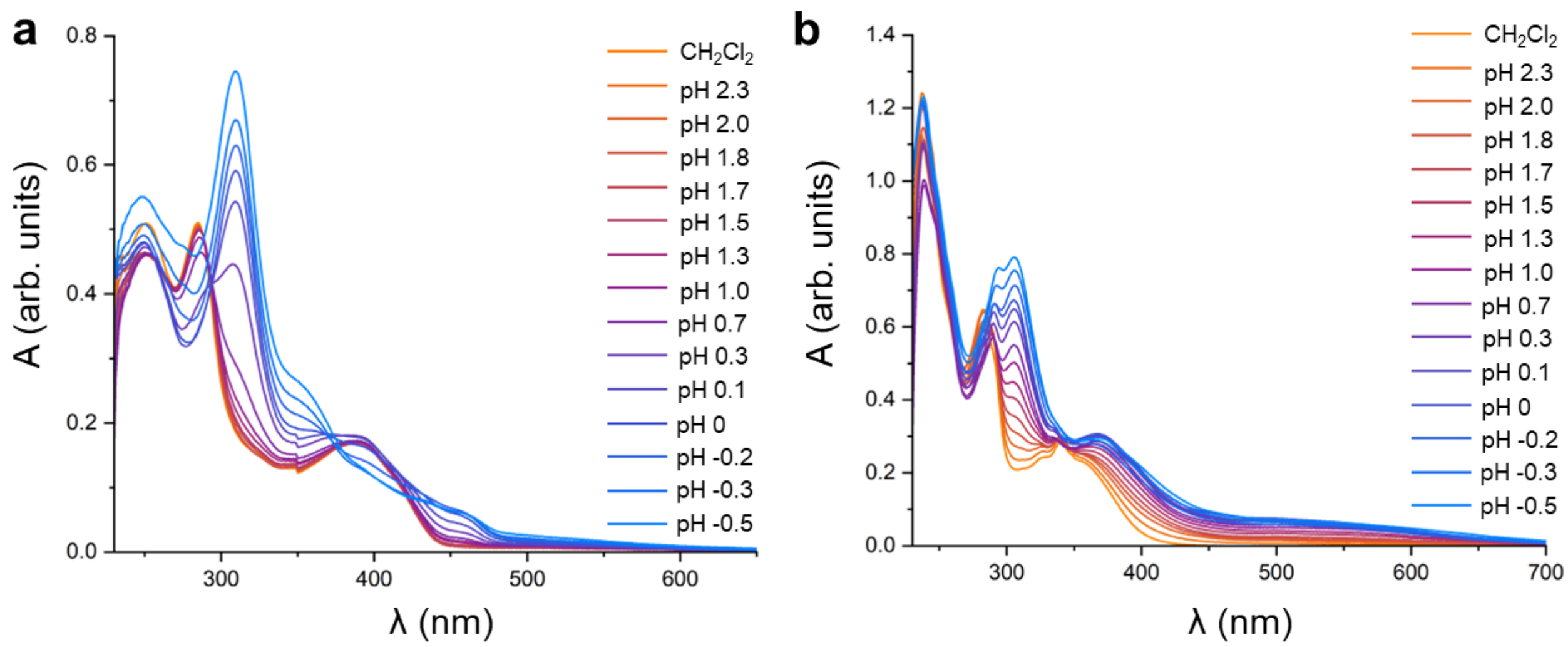

Fig. S21. UV-vis spectra of a) **7c** and b) **7d** in increasingly higher concentrations of MsOH, leading to the formation of **4c** and **4d** respectively (CH_2Cl_2/MsOH, *l* = 10 mm, *c* = 10 µM).

Table S4. Variation in absorbance of **7b** at 305 nm (A_{305}) at different pH values where R^2 = 0.99222 using **Equation S3**.

[MsOH]	pH	Abs
0.00	-	0.267
0.005	2.30	0.278
0.01	2.00	0.320
0.015	1.82	0.389
0.020	1.70	0.515
0.035	1.456	0.675
0.050	1.30	0.683
0.10	1.00	0.920
0.20	0.70	1.033
0.50	0.30	1.117
0.80	0.10	1.129
1.00	0	1.144
1.50	-0.18	1.160
2.00	-0.30	1.184

Table S5. Variation in absorbance of **7c** at 305 nm (A_{305}) at different pH values where R^2 = 0.99859 using **Equation S3**.

**[MsOH]**	**pH**	**Abs**
0.00	-	0.212
0.005	2.30	0.216
0.01	2.00	0.218
0.015	1.82	0.222
0.020	1.70	0.230
0.035	1.456	0.236
0.050	1.30	0.250
0.10	1.00	0.267
0.20	0.70	0.313
0.50	0.30	0.443
0.80	0.01	0.526
1.00	0.00	0.567
1.50	-0.18	0.604
2.00	-0.30	0.643
3.00	-0.48	0.719

Table S6. Variation in absorbance of **7d** at 305 nm (A_{305}) at different pH values

[MsOH]	pH	Abs
0.00	-	0.211
0.005	2.30	0.240
0.01	2.00	0.276
0.015	1.82	0.314
0.020	1.70	0.357
0.035	1.456	0.405
0.050	1.30	0.449
0.10	1.00	0.502
0.20	0.70	0.550
0.50	0.30	0.614
0.80	0.01	0.649
1.00	0.00	0.673
1.50	-0.18	0.712
2.00	-0.30	0.754
3.00	-0.48	0.791

6. Fluorescence Spectroscopy

Fluorescence measurements were performed in anhydrous CH_2Cl_2. Spectra were measured in 10 mm path-length cuvette at room temperature. **4b**, **4c** and **4d** (purple line) were prepared *in situ* from their corresponding precursors: **7b**, **7c** and **7d** (orange line), respectively. The samples were sparged briefly with $N_{2\,(g)}$. The intensities were plotted as fluorescence intensities.

Samples for steady-state emission spectroscopy were prepared using 1 mg/mL CH_2Cl_2 stock solutions. The desired quantity was diluted to achieve the desired 10 µM concentration. The diluted solution was titrated with a solution mixture of MsOH (2.0 M) and **7b**, **7c** and **7d** (10 µM). After each addition of the acid, the emission spectra were recorded. The data points were fitted to the dose-response function (**Equation S4** below) to determine the pK_a:

$$I = I_i + \frac{I_i + I_f}{1 + 10^{\log\,(\mathrm{p}K_\mathrm{a} - \mathrm{pH})\cdot p}}$$

Equation S4. Where the absorption/emission intensity (*I*) was determined as a function of the initial and final absorption intensity (λ_{max} = 305 nm) or emission intensity (λ_{max} = 425 nm) ($I_{i,f}$ respectively) and *p* is the sensitivity factor. [**7**] was kept constant at 10 µM for this experiment.

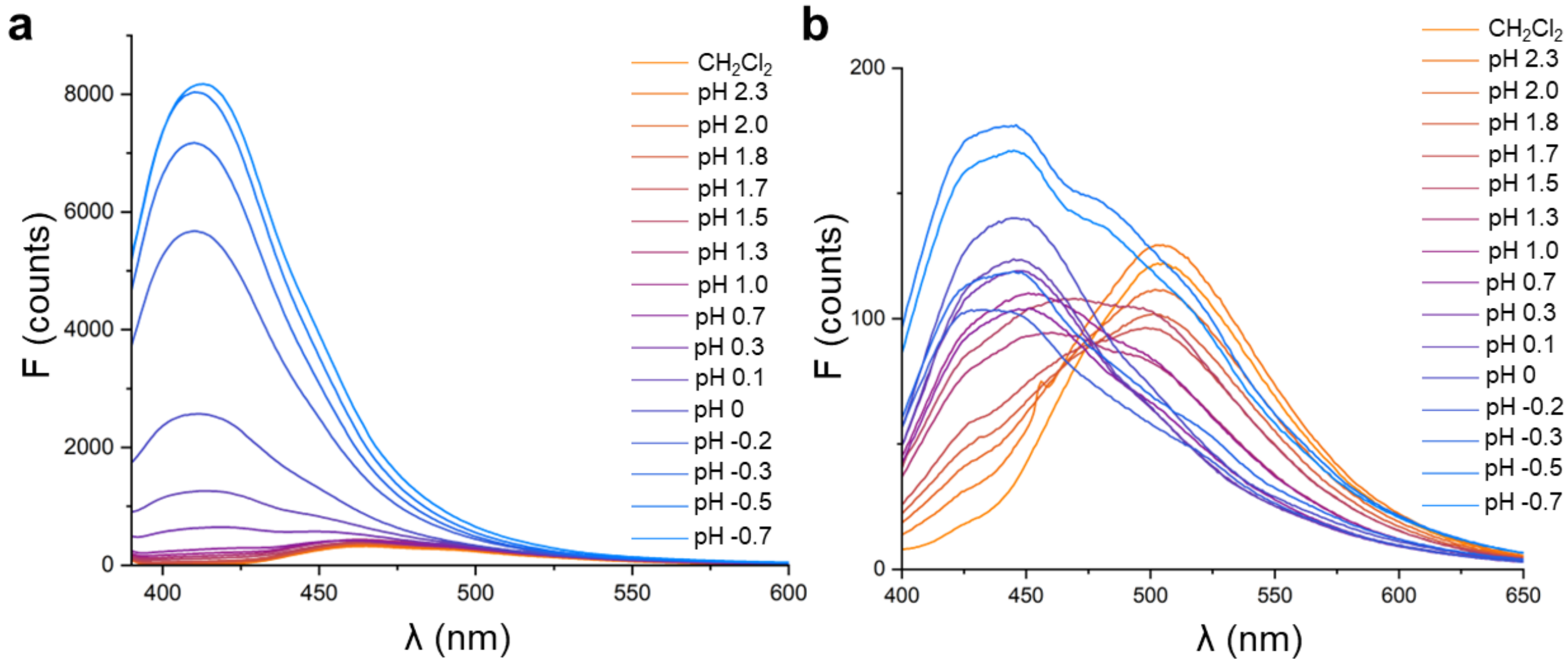

Fig. S22. Emission spectra of of a) **7c** and b) **7d** in increasingly higher concentrations of MsOH, leading to the formation of **4c** and **4d** respectively (CH_2Cl_2/MsOH, λ_{ex} = 375nm, *l* = 10 mm, *c* = 10 µM).

Table S7. Variation in emission of **7b** at 425 nm (*F*) at different pH values where R^2 = 0.99516 using **Equation S4**.

[MsOH]	pH	F
0	-	22.07
0.005	2.30	18.03
0.010	2.00	25.91
0.015	1.82	26.78
0.020	1.70	28.04
0.035	1.456	53.69
0.050	1.30	64.32
0.10	1.00	81.35
0.20	0.70	135.4
0.50	0.30	215.7
0.80	0.01	289.7
1.00	0.00	333.1
1.50	-0.18	368.8
2.00	-0.30	417.0

Table S8. Variation in emission of **7c** at 425 nm (*F*) at different pH values where R^2 = 0.99868 using **Equation S4**.

[MsOH]	pH	F
0	-	32.64
0.005	2.30	41.33
0.010	2.00	51.54
0.015	1.82	66.89
0.020	1.70	87.02
0.035	1.456	138.4
0.050	1.30	180.0
0.10	1.00	224.6
0.20	0.70	292.2
0.50	0.30	629.9
0.80	0.01	1179
1.00	0.00	2286
1.50	-0.18	4895
2.00	-0.30	6162
3.00	-0.48	6999
5.00	-0.70	7379

Table S9. Variation in emission of **7d** at 425 nm (*F*) at different pH values.

[MsOH]	pH	F
0	-	18.01
0.005	2.30	31.09
0.010	2.00	41.17
0.015	1.82	48.06
0.020	1.70	56.35
0.035	1.456	84.76
0.050	1.30	77.45
0.10	1.00	94.27
0.20	0.70	91.56
0.50	0.30	107.4
0.80	0.01	110.4
1.00	0.00	126.3
1.50	-0.18	102.1
2.00	-0.30	112.4
3.00	-0.48	170.7
5.00	-0.70	158.3

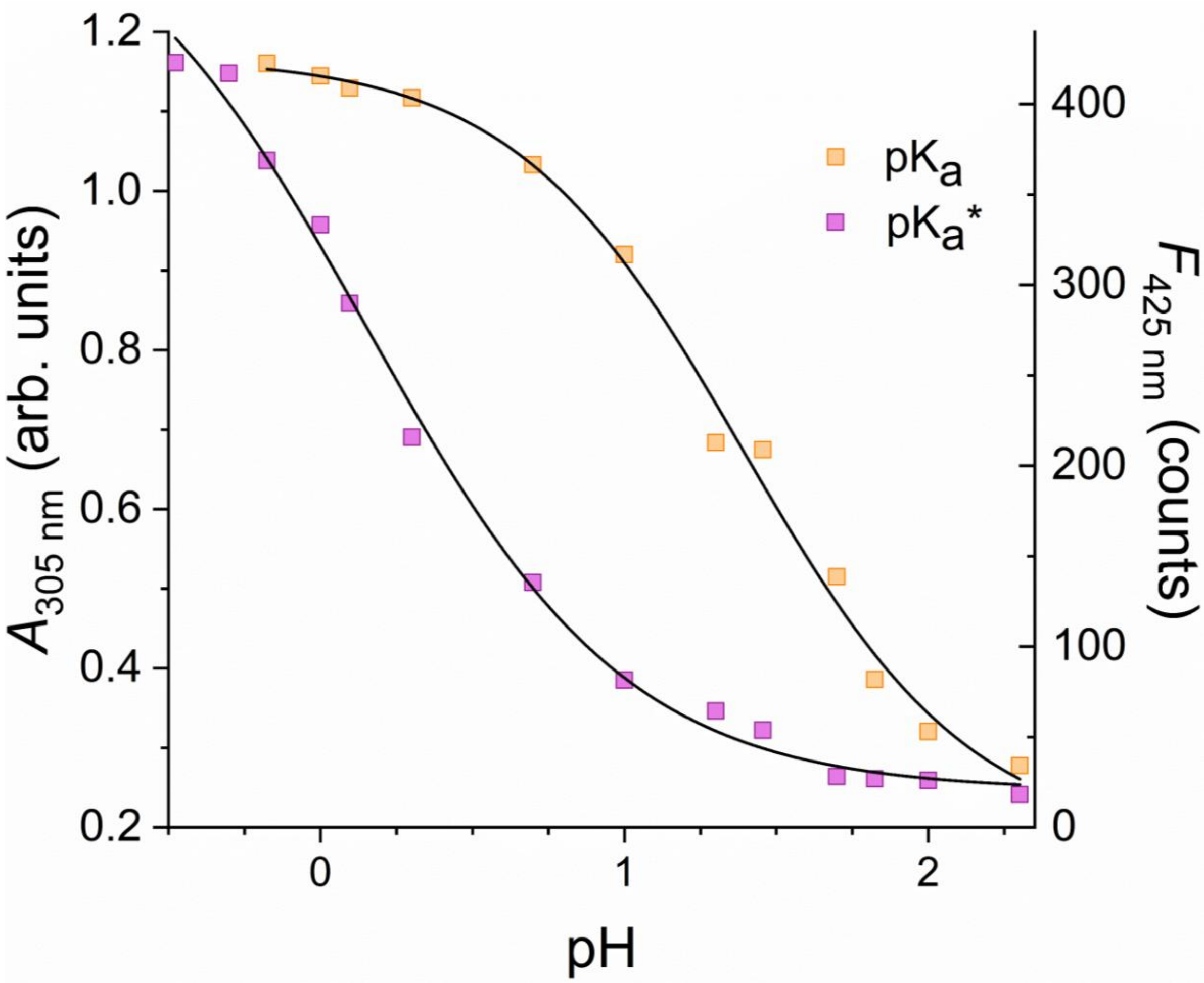

Fig. S23. Variation of absorbance (orange squares) at 305 nm (A_{305}) and emission (purple squares) at 425 nm (*F*) of **7b** at different pH values. The line of best fit corresponds to a one step protonation equilibrium.

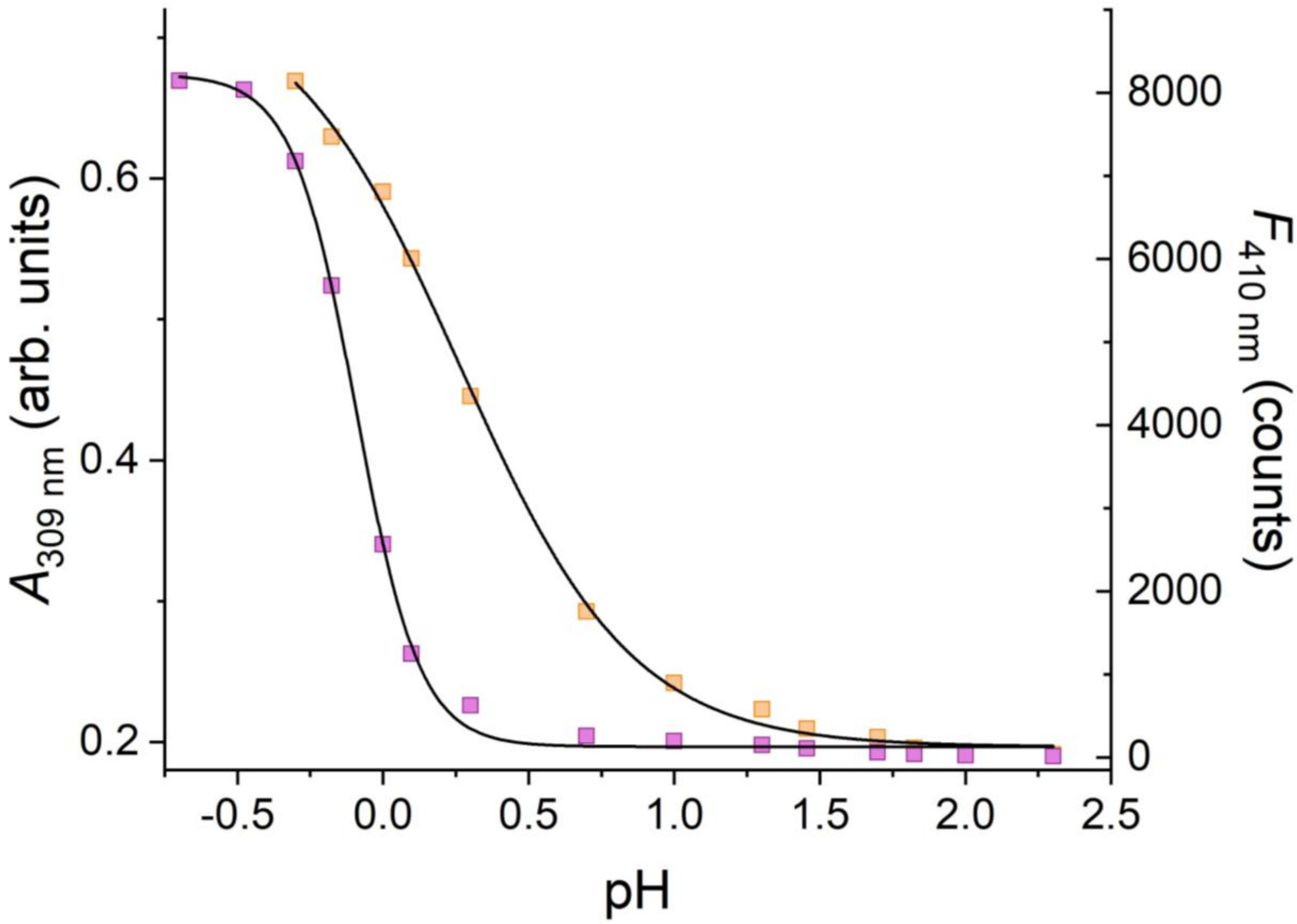

Fig. S24. Variation of absorbance (orange squares) at 305 nm (A_{305}) and emission (purple squares) at 410 nm (*F*) of **7c** at different pH values. The line of best fit corresponds to a one step protonation equilibrium.

7. Phosphorescence (ICCD)

Photoluminescence measurements were carried out using 2 μM solutions. Emission and excitation spectra were recorded with a Jobin Yvon Horiba Fluorolog-3, while absorption spectra were measured using a Shimadzu UV-VIS-NIR 3600 spectrophotometer.

Time-resolved measurements were performed using a gated iCCD camera system (250–950 nm), with excitation provided by a 150 ps Nd:YAG laser (355 nm). For temperature-dependent measurements, a Janis Research VNF-100 cryostat was employed.

Time-correlated single photon counting (TCSPC) measurements were conducted on a Horiba DeltaFlex system, using two excitation sources: a 301 nm delta diode and a 357 nm nanoLED. The resulting decay curves were deconvoluted using the FluorTools software package, DecayFit and fitted using a biexponential fit:

$$I(t) = a_1 e^{\left(-\frac{t}{t1}\right)} + (1 - a_1) e^{\left(-\frac{t}{t2}\right)}$$

Equation S6. Biexponential fit where *I* was the absorption/emission intensity, a_1 is the pre-factor for the first exponential term, t is the time variable, t_1 is the time for the first exponential decay and t_2 is the time for the first exponential decay

Steady-state photoluminescence measurements for compound **7b** in CH_2Cl_2 show a main emission band cantered at 360 nm. Excitation spectra recorded while monitoring this emission reveal a primary excitation band between 290 and 350 nm, which corresponds to the low-energy tail of the absorption spectrum shown in Fig. S25a. Time-resolved photoluminescence (TRPL) measurements were carried out by exciting at 355 nm, targeting this tail region. At 300 K, the emission consists of

a weak prompt signal (Fig. S25c). Due to the use of a 360 nm cut off filter, most of the peak emission is not detected, although the system dynamics are still observable, this emission was monitored using time-resolved single photon counting (TCSPC) and the fitted lifetime shows a bi-exponential behavior with lifetimes of 1.68 and 0.15 ns (Table S10), this biexponential behavior can be accredited to the low solubility of the molecule, having a degree of dimmer even at dilute 2 μM, a small visible and weak red shift is observed in the first nanoseconds of emission.

Upon cooling to 80 K, the prompt emission undergoes a red shift, with the main peak appearing at 500 nm. This shift is consistent with the molecules adopting more rigid conformations and increased molecular interactions, most probably due to aggregation as the solvent cools gradually. The redshifted species exhibits a faster decay than the monomer-like emission, which persists at approximately 100 ns. In the microsecond range, a new, structured, lower-energy species becomes emissive and remains visible into the millisecond regime, consistent with a ^{3}LE (triplet local excited) state (Fig. S25d).

Further evidence of solvent-induced aggregation is observed in additional temperature-dependent measurements (Fig. S26). As CH_2Cl_2 approaches its freezing point, the increasing viscosity provides a more restricted molecular environment, promoting interactions that lead to dimer formation, once the solvent is frozen at 120 K phosphorescence starts to be present in the microsecond time range, but weaker intensity than at 80 K, due to possibly still having active non-radiative pathways.

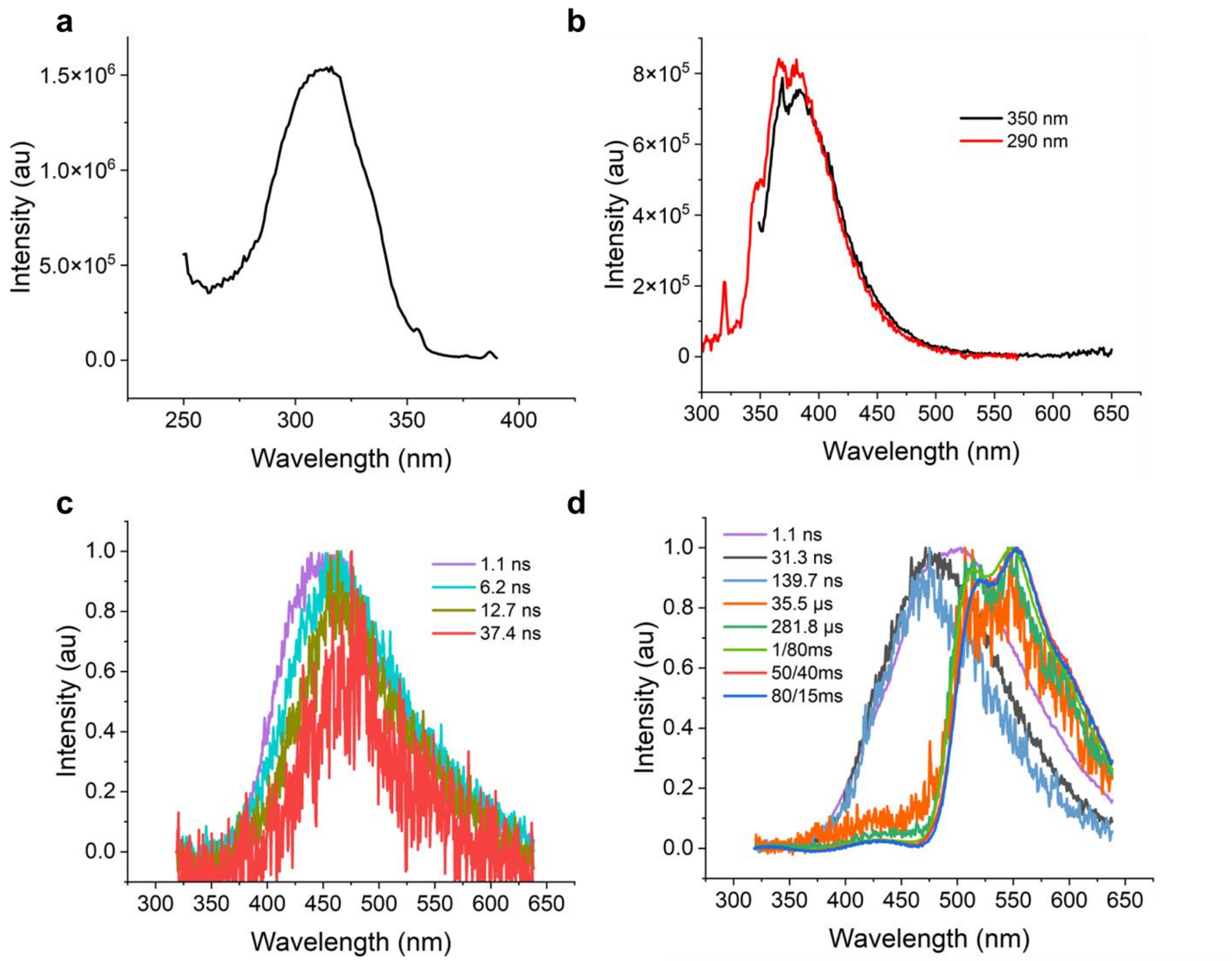

Fig. S25. a) Excitation spectrum λ_{em} = 400 nm, b) steady state photoluminescence (with different excitation wavelengths, λ_{ex}), and time resolved photoluminescence at c) 300 K and d) 80 K of **7b** in solution (CH_2Cl_2, *c* = 2 μM, λ_{ex} = 355 nm, 298 K). For ms-timescale measurements, the first value denotes the delay between excitation and measurement, while the second value refers to the gate width (that is, the duration over which the spectra were recorded).

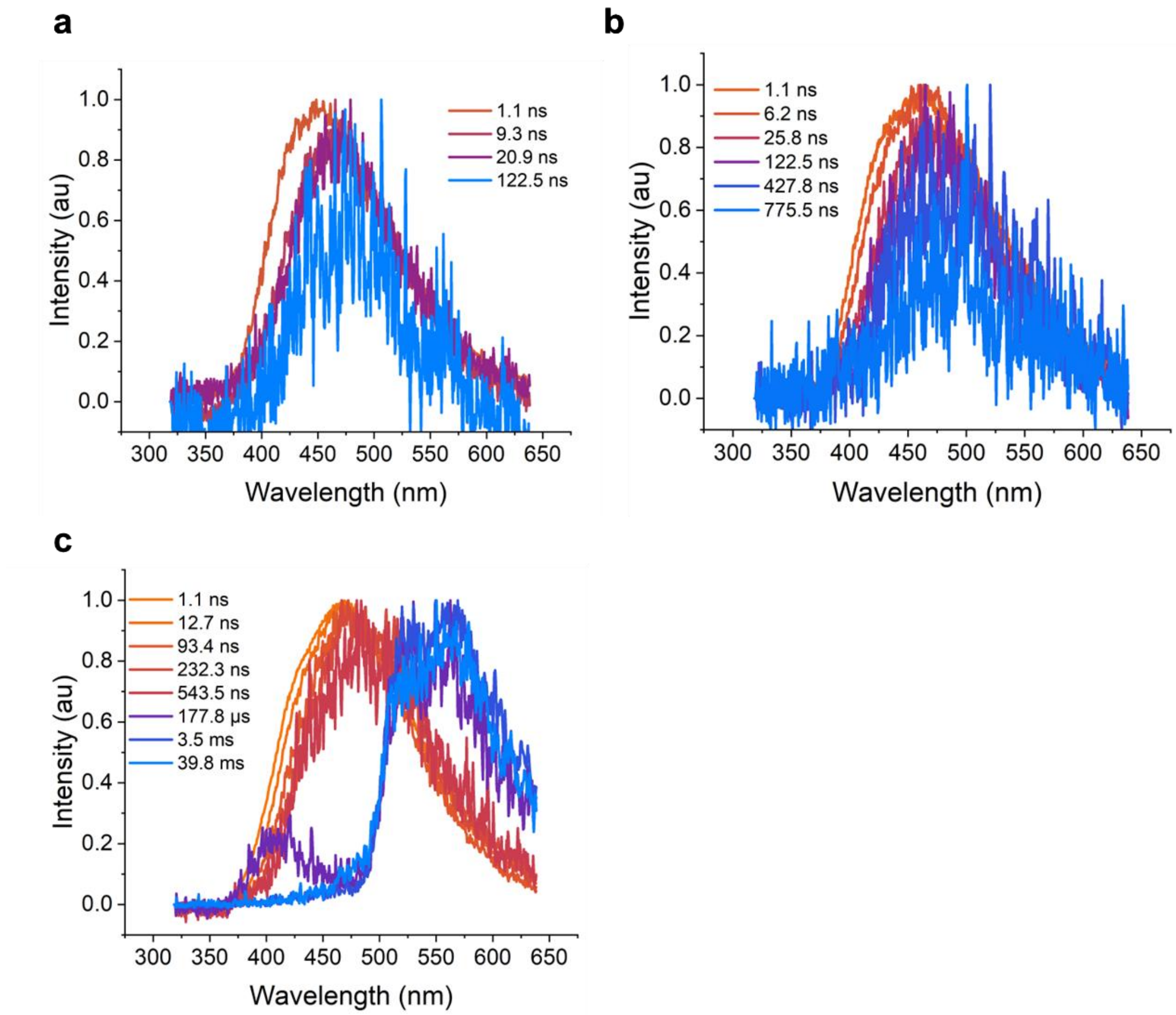

Fig. S26. Time-resolved photoluminescence at a) 240 K, b) 180 K and c) 120 K of **4b** in solution (*c* = 2 μM, CH_2Cl_2/MsOH, 1 M, λ_{ex} = 355 nm).

Table S10. TCSPC fitting parameters. All solutions had a concentration of 2 μM (λ_{ex} = 301 nm for **7b–c** , λ_{ex} = 357 nm for **4b–d**).

Molecule	Band [nm]	a1	T1 [ns]	T2 [ns]	S_1 [eV][a]	$_3$LE [eV][b]	ΔST [meV]
**7b**	400	0.8	1.68	0.147	3.74	2.91	830
**4b**	425	1	2.309	-	3.25	2.73	480
	510	0.76	1.493	6.29			
**7c**	375	0.39	0.486	1.985	4.27	2.60	1670
**4c**	425	1	2.003	-	3.13	2.70	430
	500	0.05	7.02	1.767			
**7d**	400	0.69	1.523	0.066	3.83	2.89	940
**4d**	420	1	1.935	-	3.42	2.88	540
	530	0.83	1.547	8.177			

[a] All S_1 energies were obtained using the steady state onset. [b] Local triplet energies were obtained using the onset of late ms times of TRPL spectra.

To investigate the effects of protonation, compound **7b** was dissolved in a solution of methanesulfonic acid (MsOH) in CH_2Cl_2 (1 M) to generate **4b**. Both species were studied spectroscopically. As shown in Fig. S27, both the steady-state emission and excitation spectra exhibit changes consistent with the previously observed absorption shifts. The emission spectrum displays two main bands, with maxima at 420 nm and 530 nm, corresponding to emission from **4b** in its E* and K* forms, respectively. The excitation spectrum monitored at 420 nm shows a defined profile, while excitation monitored at the redshifted emission band appears broad and structureless. The dynamics of this system at room temperature are shown in Fig. S27c. The E* tautomer emission decays rapidly with a lifetime of 2.30 ns, while the redshifted K* emission persists with a longer decay time of 6.29 ns. The results of TCSPC measurements show bi-exponential decay behavior in the lower energy region, with a fast lifetime of 1.49 and 6.29 ns.

To examine the origin of the peak in the red spectral region, sum-normalized prompt emission spectra were studied at two concentrations (2 and 100 µM analyte concentration), as shown in Fig.

S28. At the lower concentration, the emission at *t* = 0 ns begins in the red-shifted region with a 540 nm peak and rapidly evolves towards the profile of E* emission. Subsequently, this species undergoes decay, and after 9.3 ns, we observe a single peak at 510 nm which is assigned as the K*-**4b** emission. At higher concentrations, the initial emission is more distinctly red-shifted, with a peak at 540 nm. The subsequent dynamics resemble those of the dilute sample, but the increased intensity of this band and the limited solubility of the compounds suggest it arises from pre-formed (i.e., ground-state) dimer emission. This assignment is supported by the presence of two isoemissive points in Fig. S28b, indicating the coexistence of three distinct emissive species within the same short temporal window.

Fig. S27d shows the emission spectrum of K-**4b** at cryogenic temperatures. In the prompt region, the emission maximum appears at 540 nm, consistent with the previously identified dimer-associated band, accompanied by a bluer shoulder around 450 nm. Based on the behavior observed for **7b**, these results suggest an increase in cooling-assisted aggregation at 80 K compared to room temperature. At this lower temperature, a greater proportion of dimer emission is observed, while the monomer contribution is reduced, as indicated by the diminished prompt emission associated with the monomer state.

Following excitation, the dual emission from 450 and 540 nm persists beyond 60 ns, after which the band at 450 nm becomes the dominant feature. In the microsecond time domain, the emission is characterized by a main band with a maximum at 590 nm, followed by a final emission feature with an onset of 454 nm. This latter band is attributed to the ^{3}LE state of the **4b** monomer. The earlier, redshifted microsecond emission at 590 nm is distinct from any previously measured signals and is tentatively assigned to phosphorescence from dimeric species.

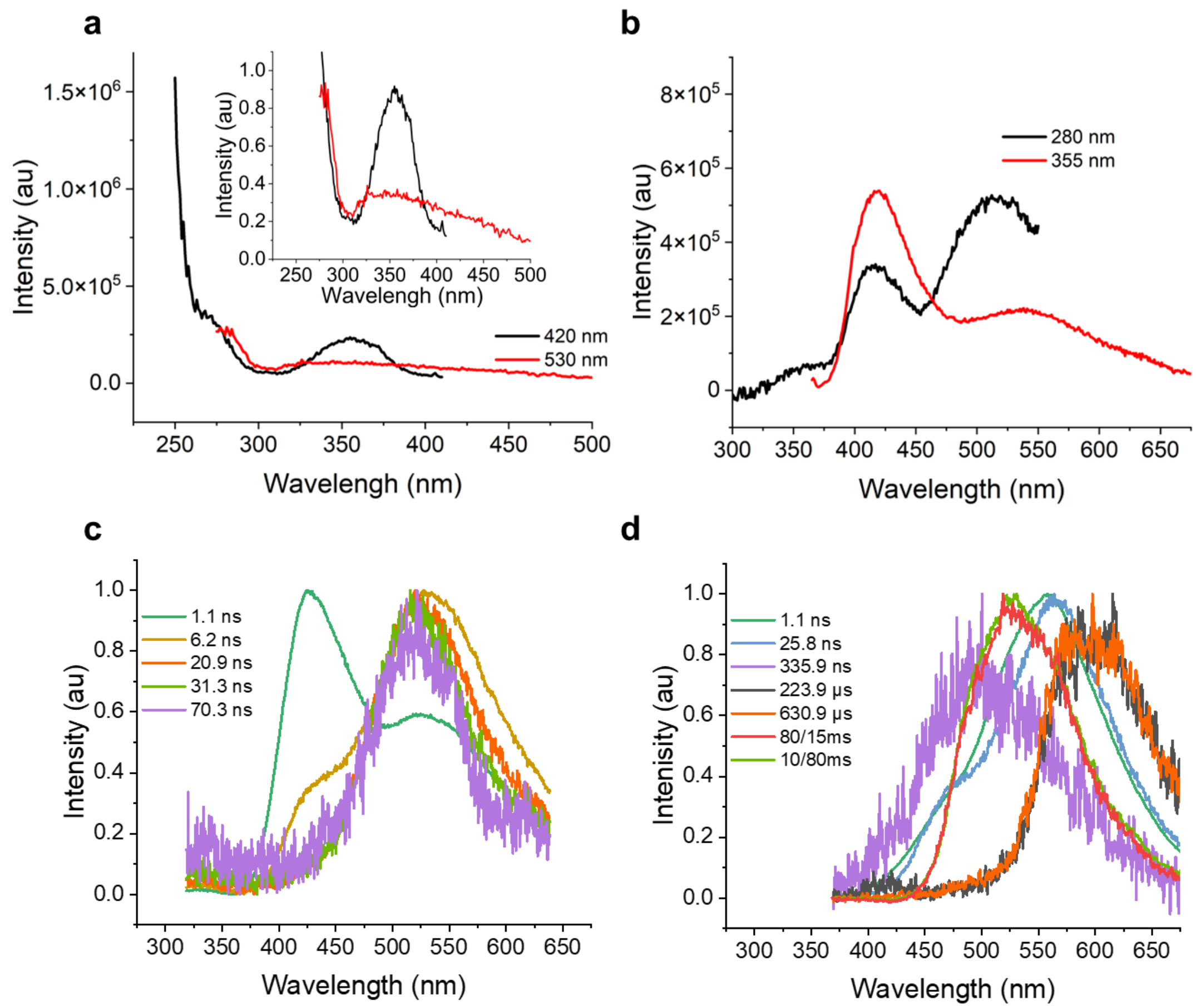

Fig. S27. a) Excitation spectra (normalized inset) monitored at different emission wavelengths (λ_{em}); b) steady state photoluminescence (with different excitation wavelengths, λ_{ex}) and time-resolved photoluminescence spectra at c) 300 K and d) 80 K of **4b** (c = 2 μM, MsOH/CH_2Cl_2, 1 M, λ_{ex} = 355 nm, 298 K). For ms-timescale measurements, the first value denotes the delay between excitation and measurement, while the second value refers to the gate width (that is, the duration over which the spectra were recorded).

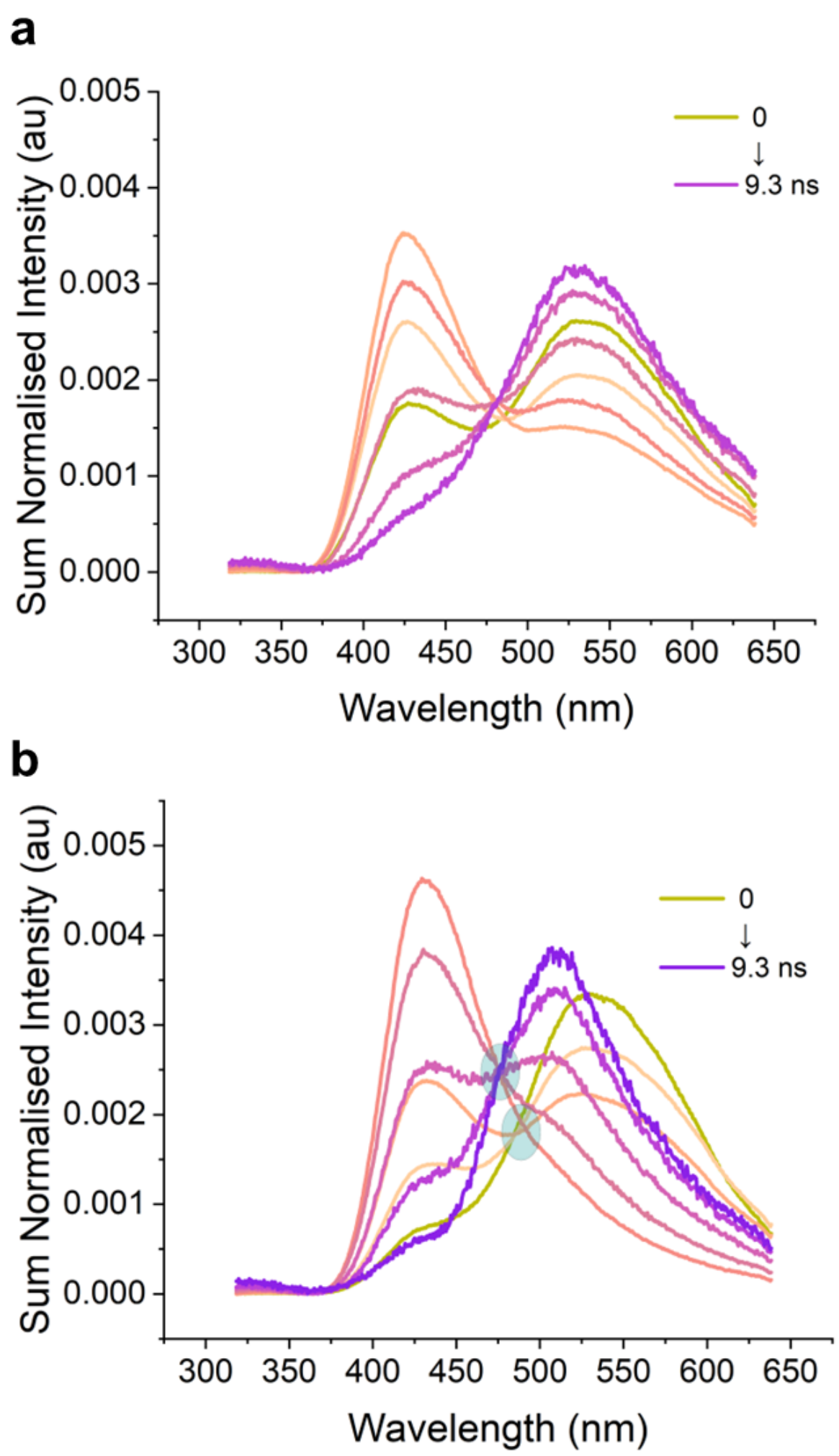

Fig. S28. Sum-normalized time-resolved photoluminescence prompt emission spectra of **4b** in solution (MsOH/CH_2Cl_2, 1 M, λ_{ex} = 355 nm, 298 K) at a concentration of a) 2 µM; and b) 100 µM.

To further investigate the emergence of the 450 nm peak in the late nanosecond range, time-resolved photoluminescence measurements were conducted in even more dilute solutions (0.2 µM analyte concentration). At 300 K (Fig. S29a), the prompt emission appears less disjointed compared to concentrated samples, with features from both the E* and K* tautomers contributing to a more uniform emission profile. After 1 ns, the blue shoulder peak decays, leaving only the 510 nm peak, consistent with previously observed behavior.

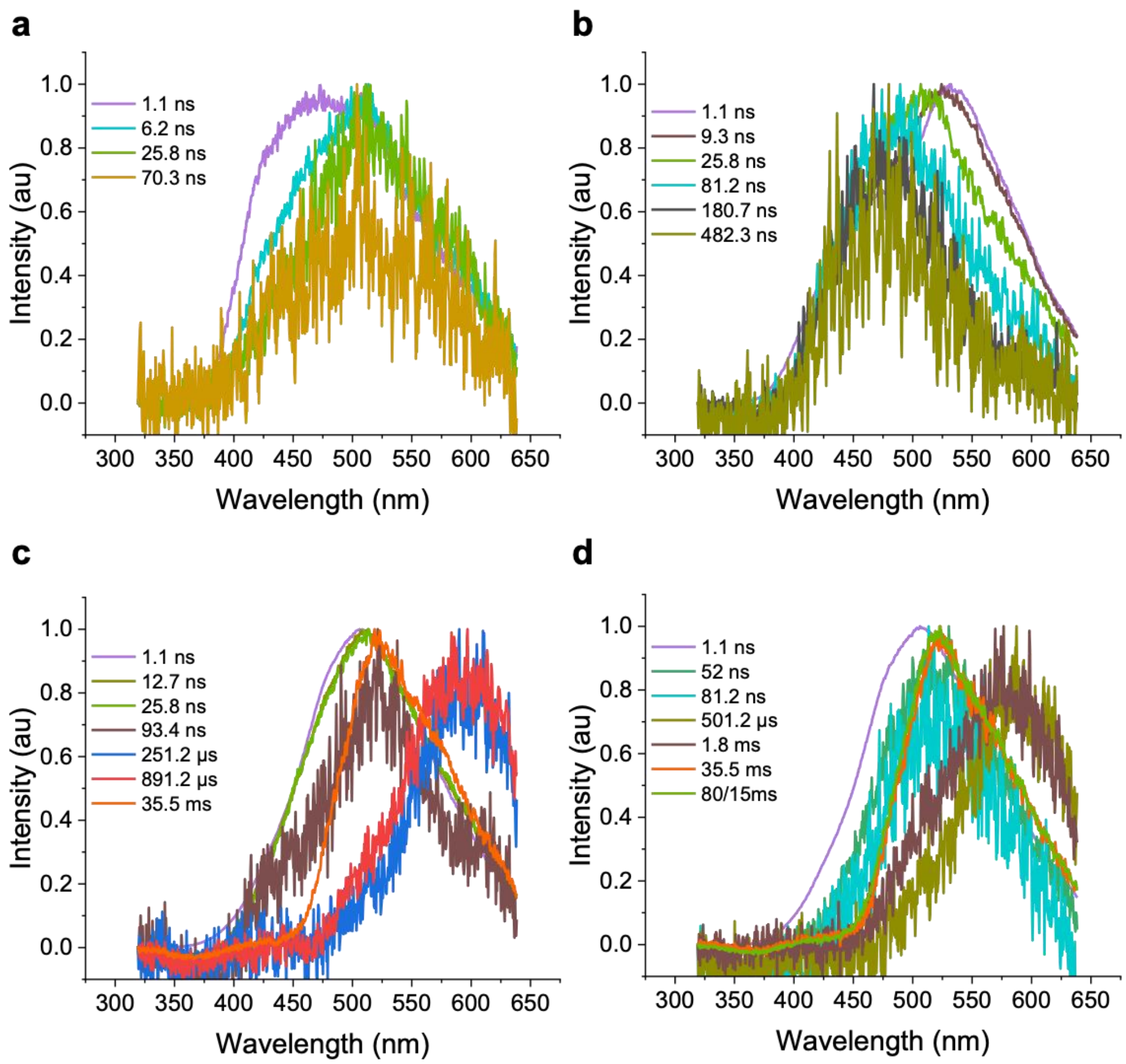

Fig. S29. Time resolved photoluminescence of **4b** at a) 300, b) 180, c) 120 and d) 80 K in dilute solution (*c* = 0.2 μM, MsOH/CH_2Cl_2, 1 M, λ_{ex} = 355 nm). For ms-timescale measurements, the first value denotes the delay between excitation and measurement, while the second value refers to the gate width (that is, the duration over which the spectra were recorded).

Upon cooling the sample to 180 K, the 550 nm aggregate-associated band reappears, even at concentrations one and three orders of magnitude lower than those used in earlier studies. This indicates a continued tendency toward aggregation under reduced thermal motion. After 25 ns, the redshifted band decays, and only the longer lived 450 nm emission remains. Further cooling to 120 K results in the disappearance of the 450 nm band, with the emission dominated by a persistent aggregate-associated band. At this temperature, the solution is frozen, and beyond 100 ns, this

aggregate emission is switched off. In the microsecond regime, aggregate phosphorescence emerges and persists into the millisecond range, showing the same onset as previously recorded. At both 120 K and 80 K, the initial decay frame with a delay of 1.1 ns reveals a minor contribution from the monomer emission, likely from the E*-**4b** form. However, the overall emission is primarily governed by dimer interactions.

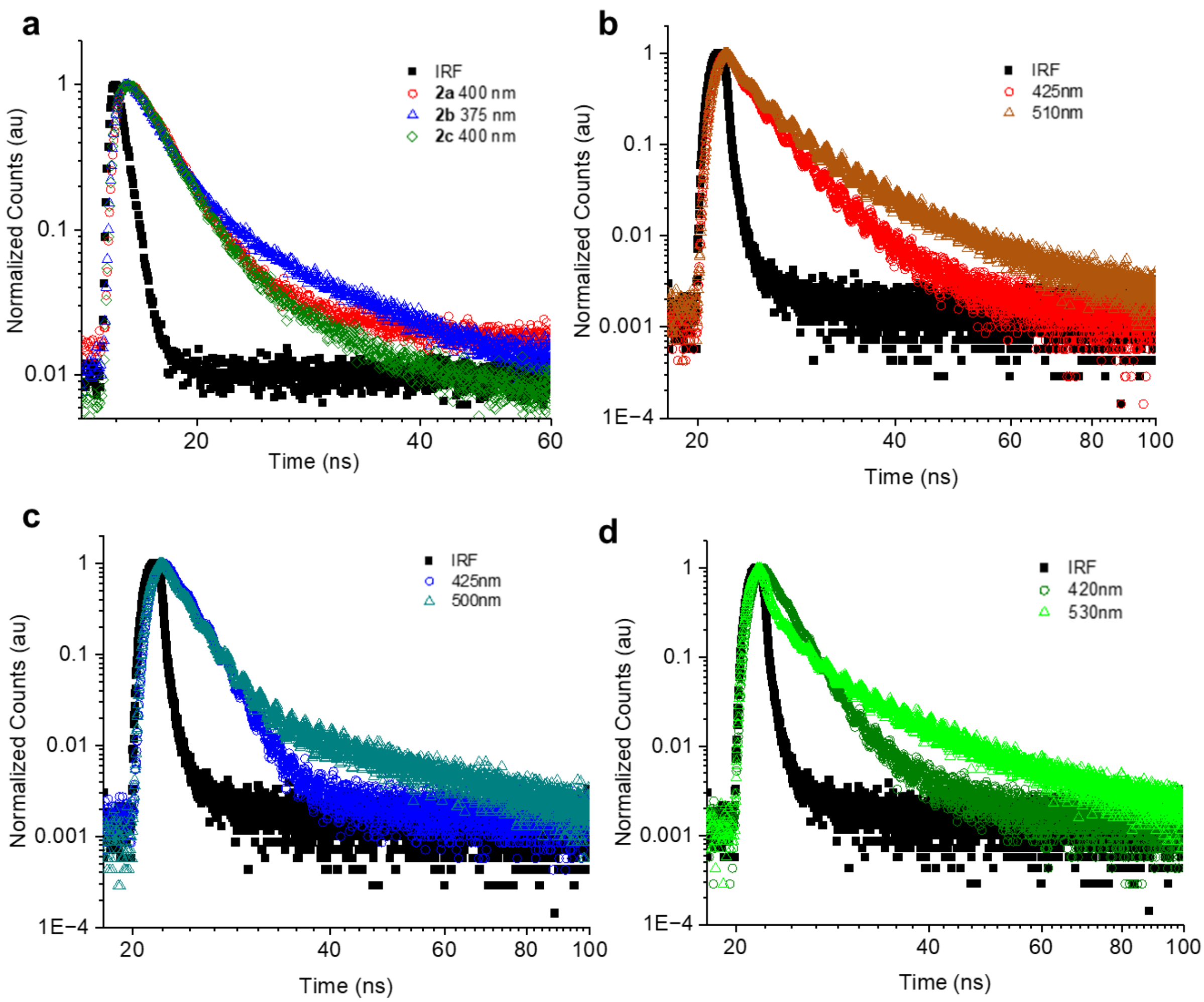

Fig. S30. TCSPC decay profiles: (a) the molecules **7b**, **7c** and **7d** in in solution (c = 2 μM, MsOH/CH_2Cl_2, 1 M) monitored at different wavelengths (λ_{ex} = 301 nm); (b) compound **4b** monitored at 425 and 510 nm; (c) compound **4c** monitored at 425 and 500 nm; (d) compound **4d** monitored at 420 and 530 nm (c = 2 μM, MsOH/CH_2Cl_2, 1 M, λ_{ex} = 357 nm).

The optical properties of **7c** closely resemble those of **7b**, following a consistent trend with only minor differences in spectral details. Under the same experimental conditions applied to **7b**, the steady-state photoluminescence of **7c** shows a primary emission band in the UV region, peaking at 375 nm (Fig. S31b). When this emission is monitored (Fig. S31a), a corresponding excitation band is observed, with a maximum at 320 nm.

Time-resolved measurements performed at room temperature (Fig. S31c), using excitation at the tail of the absorption band, show an initial emission peak at 440 nm during the first few nanoseconds. This emission subsequently decays over approximately 20 ns, giving way to a slightly redshifted and weaker band centered at 460 nm. This shift suggests the involvement of aggregation effects, like those seen in **7b.**

At 80 K, the behavior becomes more pronounced (Fig. S31d). The prompt emission shows a main peak at 460 nm, and after 300 ns the blue shoulder disappears, leaving a weaker but clearer emission associated with aggregated species. This emission then decays fully in the late nanosecond-to-early-microsecond range. Phosphorescence becomes evident after several hundred microseconds, with an onset energy of 2.60 eV, corresponding to the ^{3}LE state.

Figure S32 shows the optical behavior of **4c** (2 µM analyte concentration, MsOH/CH_2Cl_2, 1 M) in solution. The excitation spectrum in Figure S32a, collected while monitoring the 425 nm emission, presents a main peak around 330 nm, consistent with the redshifted absorption following protonation. The corresponding steady-state emission (Figure S32b) displays two main bands at 425 and 550 nm, associated with the E* and K* tautomers of **4b**, respectively, similarly to what is observed in **4b**. When excited with 280 nm light, an additional higher energy emission appears, which aligns well with the features seen in **7c**. Monitoring this emission also yields an excitation

spectrum matching that of **7c**, suggesting the presence of a small amount of unprotonated species under UV excitation.

At room temperature, TRPL data (Fig. S32c) show an initial emission centered at 425 nm that decays within the first few nanoseconds. This is followed by a broader, redshifted emission with a peak at 525 nm, which persists over a longer period. As seen in **4b**, this redshifted emission in **4c** also presents a biexponential decay in the TCSPC data, indicating the coexistence of K* emission and contributions from pre-formed dimers (further showed in Fig. S35a). When the sample is cooled to 80 K (Fig. S32d), the emission band at 1.1 ns features a maximum at 473 nm and a shoulder peak at 545 nm. Over ~15 ns, the intensities of both peaks decrease, while a new redshifted maximum at 501 nm emerges. Based on the data recorded at 300 K, we attribute the peak at 473 nm to E*-**4c** emission, while the initial redshifted band at 545 nm corresponds to emission from pre-formed dimers. The peak at 501 nm originates from K*-**4c** emission. As these spectra were recorded in frozen solutions, conformational changes are unlikely to be the source of this change in emission wavelength in the nanosecond timescale. As such, these spectra confirm the presence of ESPT in **4c**. After a few hundred nanoseconds, this red emission fades, and a broader, red shifted phosphorescence emerges in the microsecond range, like the dimer phosphorescence seen in **4b**. This emission gradually blue shifts over time, ending with a final onset at 2.70 eV, which is attributed to the ^{3}LE state of **4c**. These results are in line with the behavior observed for the other protonated derivative in this series.

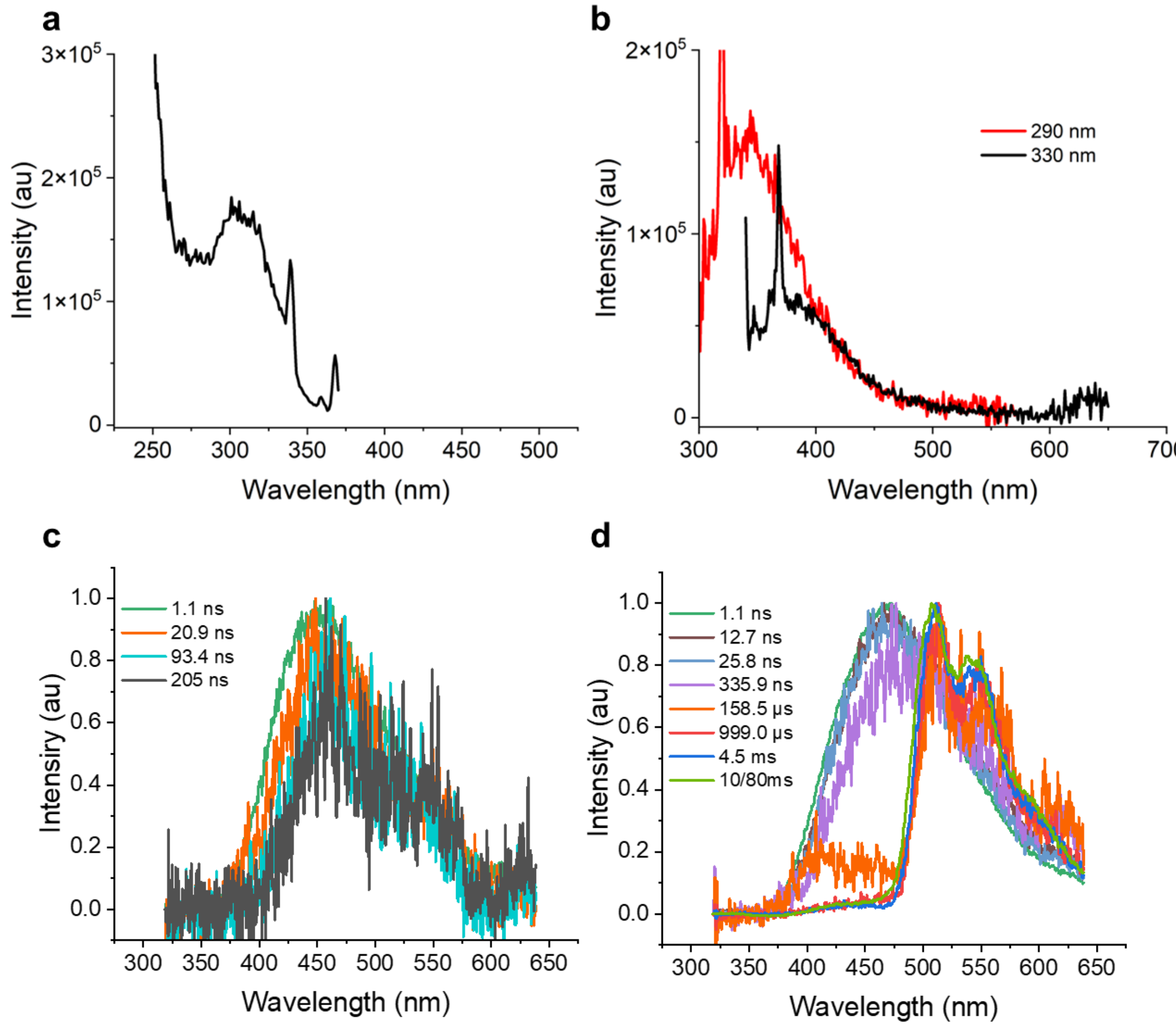

Fig. S31. a) Excitation spectrum (λ_{em} = 380 nm), b) steady state photoluminescence (with different excitation wavelengths, λ_{ex}) and time-resolved photoluminescence spectra at c) 300 K and d) 80 K of **7c** in solution (c = 2 µM, MsOH/CH_2Cl_2, 1 M, λ_{ex} = 355 nm). For ms-timescale measurements, the first value denotes the delay between excitation and measurement, while the second value refers to the gate width (that is, the duration over which the spectra were recorded).

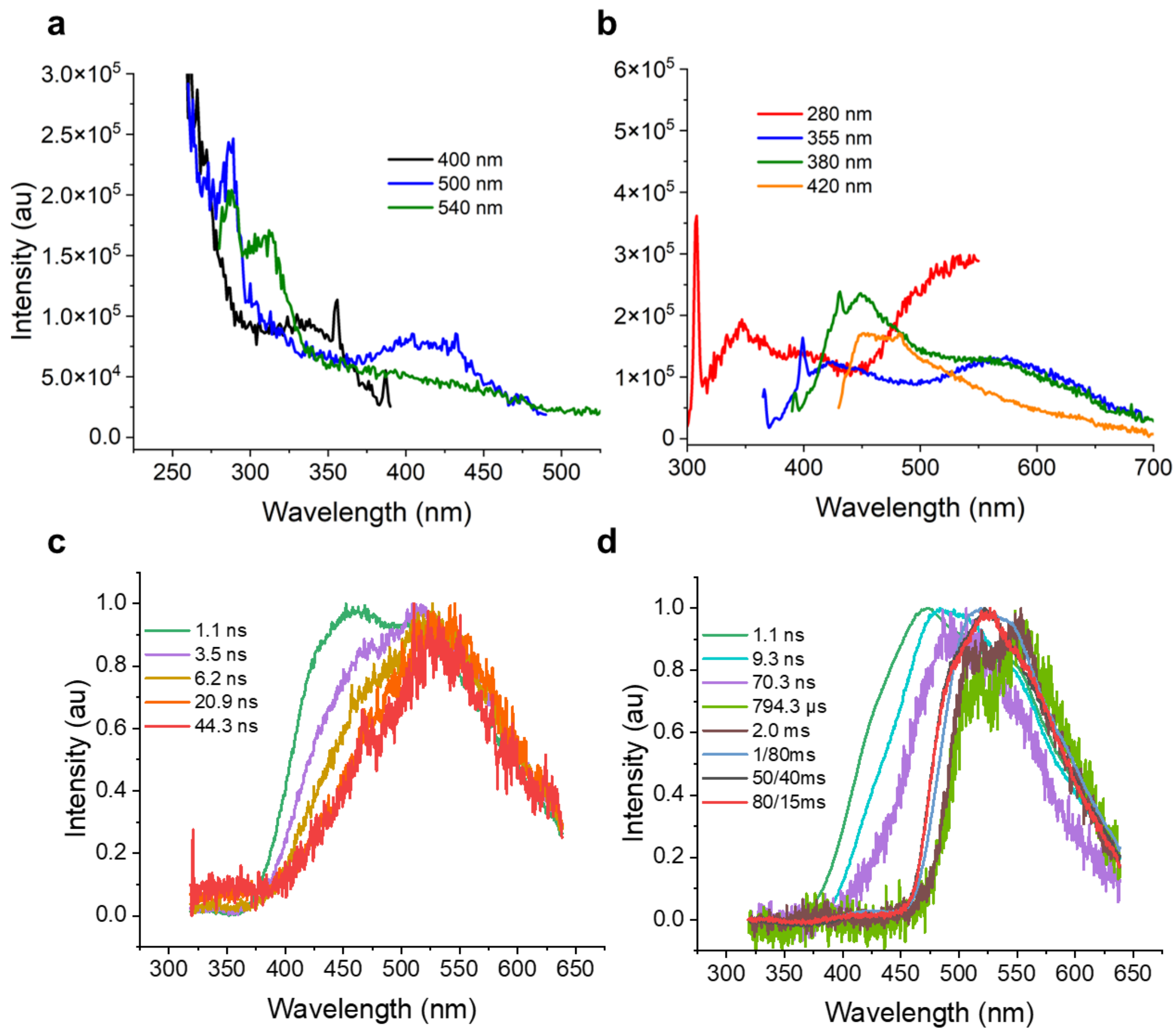

Fig. S32. a) Excitation spectra monitored at different emission wavelengths (λ_{em}); b) steady state photoluminescence (with different excitation wavelengths, λ_{ex}), and time resolved photoluminescence spectra at c) 300 K and d) 80 K of **4c** in (c = 2 μM, MsOH/CH_2Cl_2, 1 M, λ_{ex} = 355 nm). For ms-timescale measurements, the first value denotes the delay between excitation and measurement, while the second value refers to the gate width (that is, the duration over which the spectra were recorded).

Compound **7d** displays distinct photophysical behavior compared to the other two HBTs. Its steady-state emission spectrum shows a main peak at 370 nm. However, excitation at lower energy bands gives rise to a second emission band centered at 500 nm (Fig. S33b). When excitation spectra are collected for both emission bands, the 370 nm emission corresponds to a structured excitation profile like those observed in **7b** and **7c**. In contrast, the excitation band is weaker, characteristic of CT bands, while emission is broader. Both excitation and emission may include contributions from

aggregate emission as well as CT states, as suggested by the very broad PL spectrum when excited with 355 nm.

Time-resolved photoluminescence measurements were carried out at both 300 K and 80 K, Fig. S33c–d, respectively. At 300 K, the prompt emission is composed of a shoulder corresponding to the steady-state emission (partially cut off by the laser filter), with a peak around 400 nm. This feature decays within the first few nanoseconds, after which the spectrum is dominated by a redshifted emission band at 490 nm, likely arising from the CT band, consistent with the donor–acceptor architecture of **7d**.

In the late nanosecond regime, a new emission band appears at 375 nm. We assign this to ^{1}LE emission from the carbazole moiety, such as those observed in carbazole-based donor–acceptor systems.[7] This band decays over the microsecond range, followed by the emergence of a faint emission with a peak centered at 550 nm. Due to this band being lower in energy than the other emissive bands and requiring a relatively long time to form, it is most likely excimer emission.

At 80 K, the evolution of the prompt emission shows a redshift in the initial emission compared to room temperature peak emission located at 440 nm, accompanied by a shoulder associated with the aggregate emission. This emission decays within the first 200 ns, after which phosphorescence reappears from the late-microsecond-to-millisecond range. In this regime, the phosphorescence shows a slight blueshift in its onset. By using the last measured frame (80 ms delay time) energy onset the ^{3}LE is estimated to be 2.89 eV.

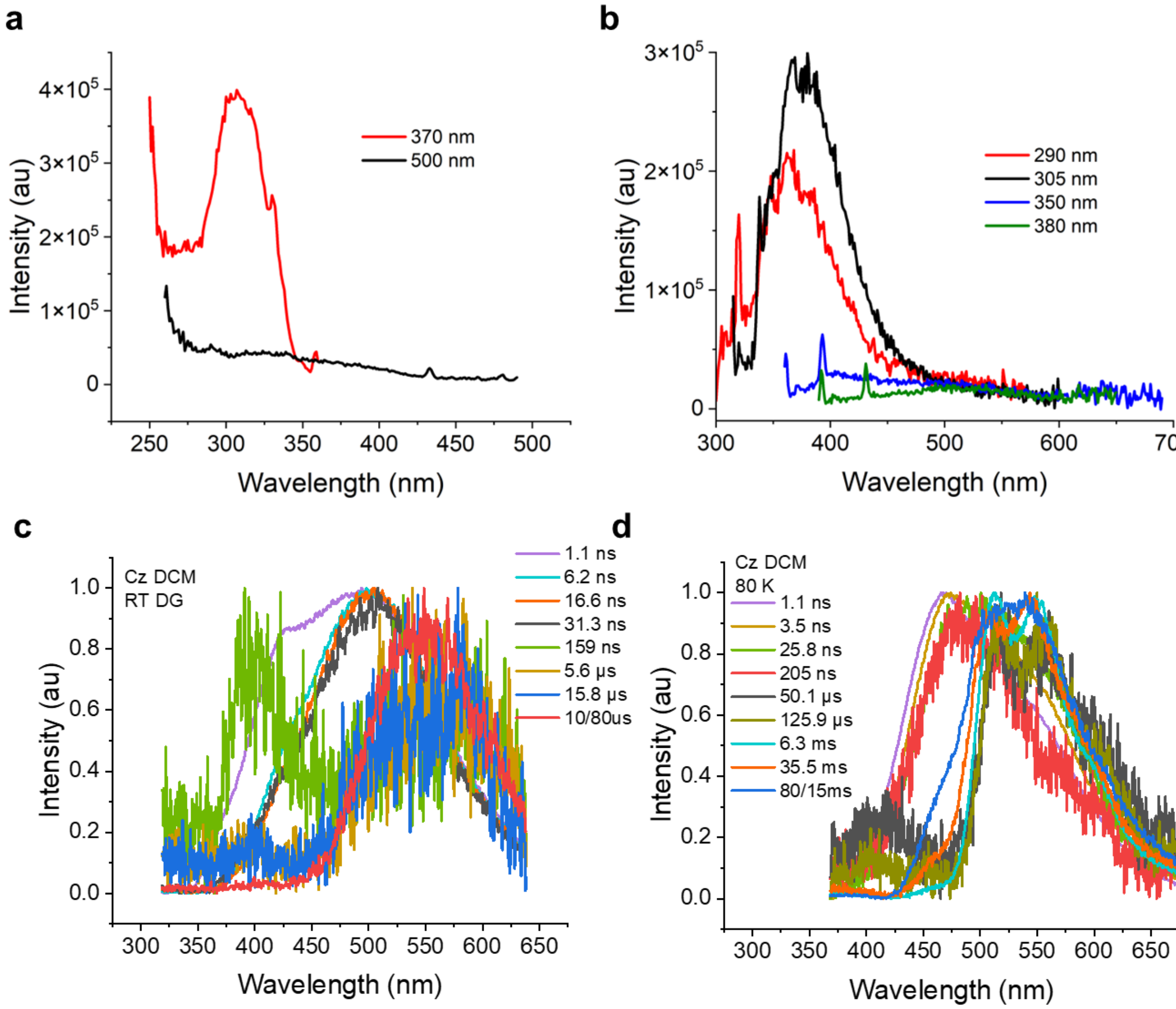

Fig. S33. a) Excitation spectra monitored at different emission wavelengths (λ_{em}); b) steady state photoluminescence (with different excitation wavelengths, λ_{ex}), and time resolved photoluminescence at c) 300 K and d) 80 K of **7d** in (c = 2 μM, MsOH/CH_2Cl_2, 1 M, λ_{ex} = 355 nm). For ms-timescale measurements, the first value denotes the delay between excitation and measurement, while the second value refers to the gate width (that is, the duration over which the spectra were recorded).

Compound **7d** was dissolved in an acidic medium (vide supra) to form **4d**, and the resulting changes in its photophysical behavior were monitored. The excitation profile of **4d** shows two distinct bands (Fig. S34a): when monitoring the 430 nm emission, a main excitation peak is seen at 330 nm, while excitation corresponding to the redder emission region reveals a broader peak centered at 440 nm.

The steady-state emission spectrum (Fig. S34b) displays two main peaks at 410 and 470 nm, along with a red tail extending into the 550–600 nm region.

The TRPL spectra in Fig. S34c at 300 K show that the 410 nm band decays rapidly, contributing minimally to the overall emission after 12.7 ns. Beyond this point, the emission evolves into a broader band with a peak at 525 nm, redshifted compared to the steady-state profile. Notably, the CT-like state previously observed in **7d** is also present in **4d**. We propose that the redshifting of the emission of **4d** occurs, due to CT-coupled ESPT. However, as this spectral evolution is evident in the parent benzotropylium **7d** as well, we are unable to attribute this redshift explicitly to ESPT.

At 80 K (Fig. S34d), the prompt emission of **4d** displays a redshifted peak in the 550–600 nm region, similar to what is seen in the room temperature photoluminescence spectrum. This is likely linked to dimer emission, as previously observed in **4b** and **4c**. This redshifted emission decays within the first 30 ns, after which a structured phosphorescence signal emerges in the late microsecond to millisecond time window. The triplet onset energy determined at this stage is 2.88 eV, which closely matches the ^{3}LE state observed in **7d**. This similarity suggests that the triplet state remains largely unaltered upon protonation, retaining a comparable local electronic character.

Additional insight is provided in Fig. S35b, which shows the sum-normalized prompt fluorescence spectra of **4d**. In contrast to **4b** and **4c**, the spectral evolution in **4d** suggests reduced contribution from pre-formed dimers. This is supported by the absence of dual phosphorescence bands in the microsecond regime, which were present in the other protonated analogues. These results indicate that **4d** is comparatively less prone to dimer formation, likely due to differences in molecular packing or steric interactions, leading to a more defined and singular emissive pathway.

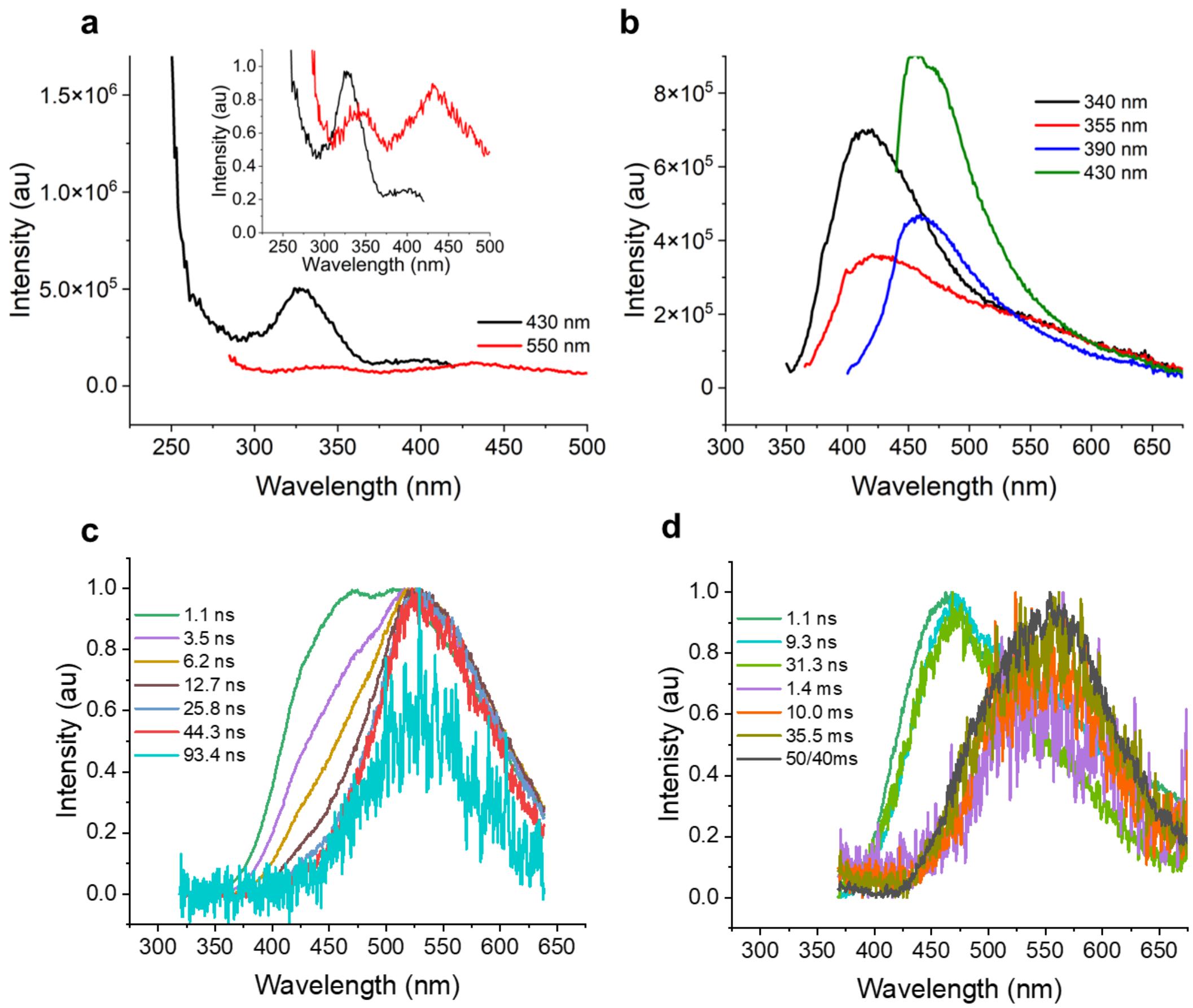

Fig. S34. a) Excitation spectra (normalized inset) monitored at different emission wavelengths (λ_{em}); b) steady state photoluminescence (with different excitation wavelengths, λ_{ex}), and time resolved photoluminescence at c) 300 K and d) 80 K of **4d** in (c = 2 μM, MsOH/CH_2Cl_2, 1 M, λ_{ex} = 355 nm). For ms-timescale measurements, the first value denotes the delay between excitation and measurement, while the second value refers to the gate width (that is, the duration over which the spectra were recorded).

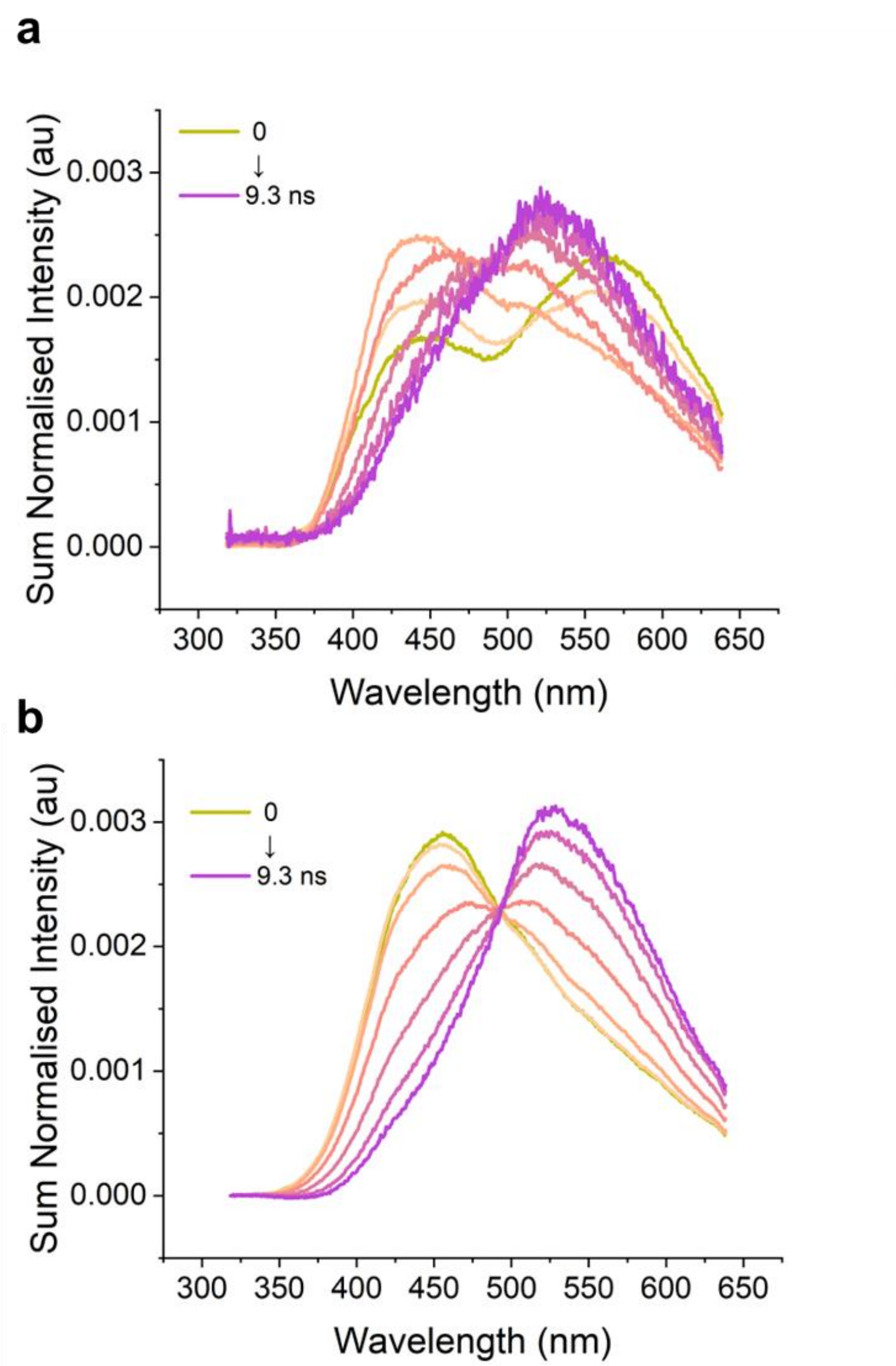

Fig. S35. Sum-normalized time-resolved photoluminescence prompt emission spectra of a) **4c** and b) **4d** in (c = 2 μM, MsOH/CH_2Cl_2, 1 M, λ_{ex} = 355 nm).

8. *In Silico* Modelling

DFT calculations were performed with Gaussian 16 revision C program package.[8] Geometry optimization and frequency calculations were performed in the gas phase in the singlet ground (S_0) and triplet excited (T_1) states with the (U)ωB97XD functional using 6-31G(d) Pople basis set families.[9,10] Frequency calculations confirm the lack of any negative vibrational frequencies for optimized structures and confirm presence of transition state structures. Time-dependent density functional theory (TD-DFT) calculations were performed using the TD-ωB97XD/6-31G(d) level of theory for the lowest singlet excited state (S_1).[11] The harmonic oscillator model of aromaticity (HOMA) values were calculated at these levels of theory as well. Nucleus-independent chemical shifts (NICS) using GIAO procedure[12] and anisotropy of the induced current density (ACID) plots using ACID 2.0.0 program with CGST method[13] were calculated on the DFT-optimized geometries at the (U)ωB97XD/6-311+G(d,p) levels. For the NICS scans in the S_1 state, state-specific CASSCF calculations using Dalton2016.0 with an active space of 6 electrons in 6 orbitals (6in6) for small molecules while 10 electrons in 10 orbitals (10in10) for the central core of the larger molecules were calculated using the TD-DFT optimized geometries, using the 6-311++G(d,p) basis set.[14,15] Electron density of the delocalized bonds (EDDB) index[16,17] calculations were performed using the CASSCF/def2-SVP level of theory, rather than 6-311++G(d,p), as this basis set is incompatible with the NBO6 program used for EDDB^k calculations.

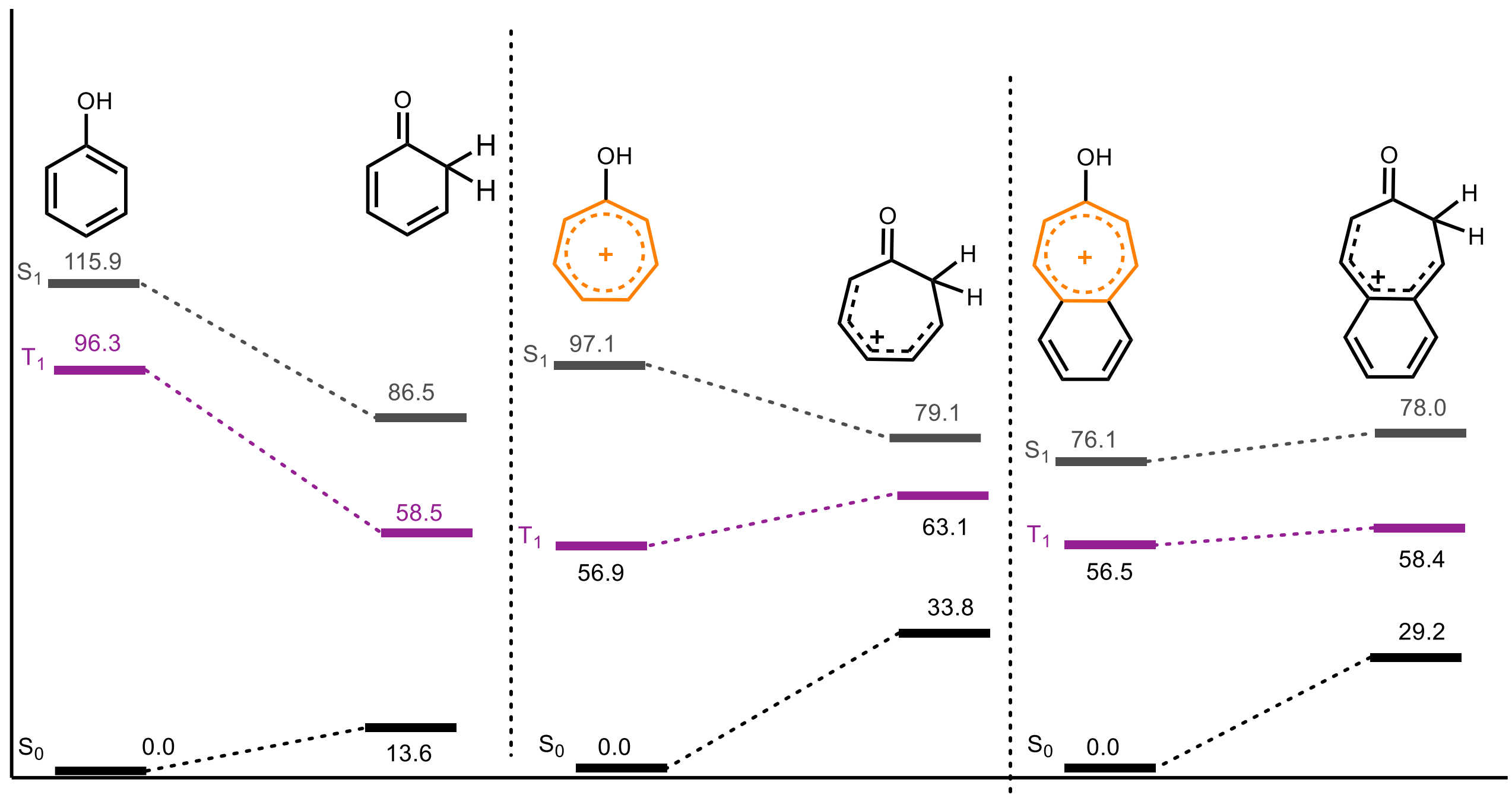

Fig. S36. Energies of S_0, T_1, and S_1 states. S_0 and T_1 states are calculated with (U)ωB97XD/6-31G(d) level. S_1 states are calculated with TD-ωB97XD/6-31G(d).

Table S11. Spin density (isosurface 0.005 a.u) at the (U)ωB97XD/6-31G(d) level in the T_1 state.

Before H-transfer	After H-transfer
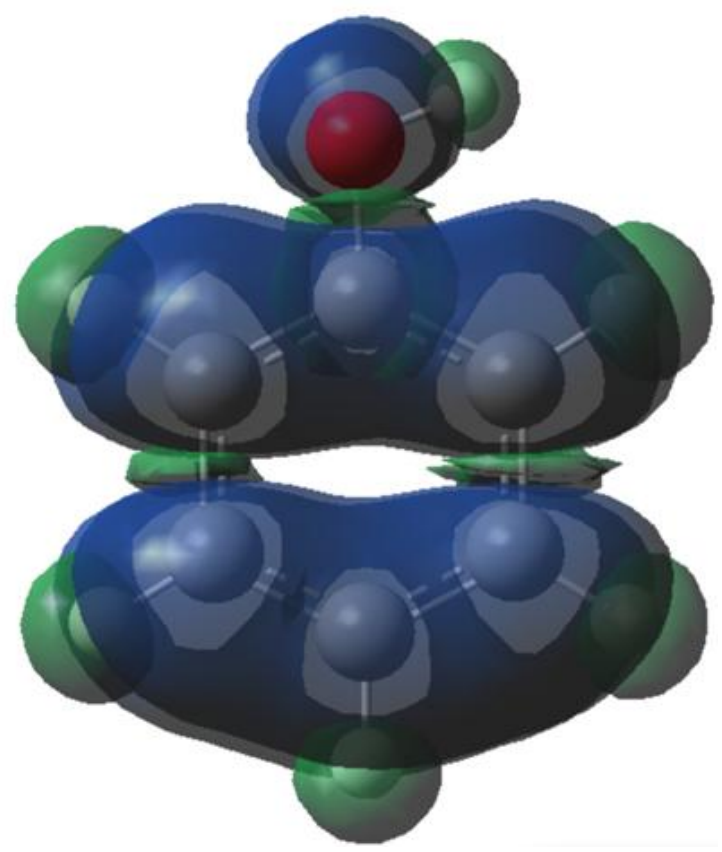	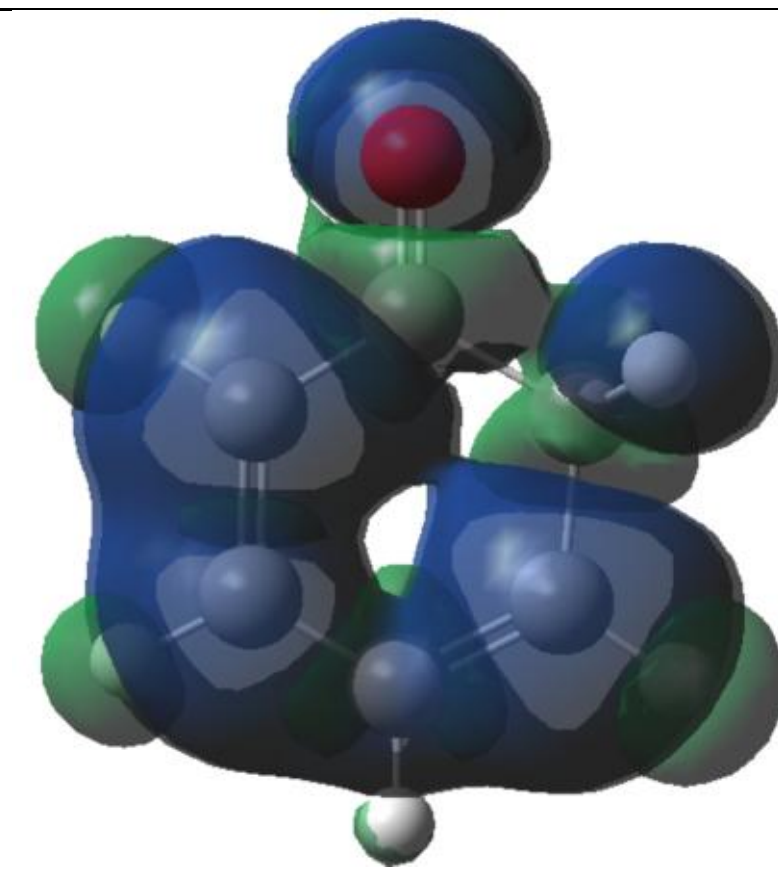
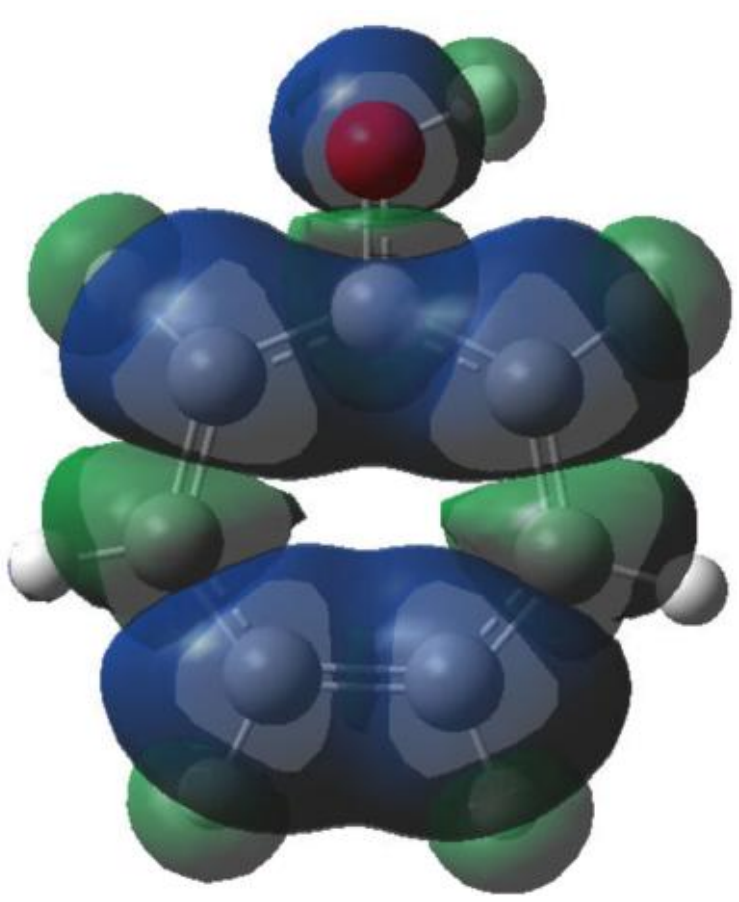	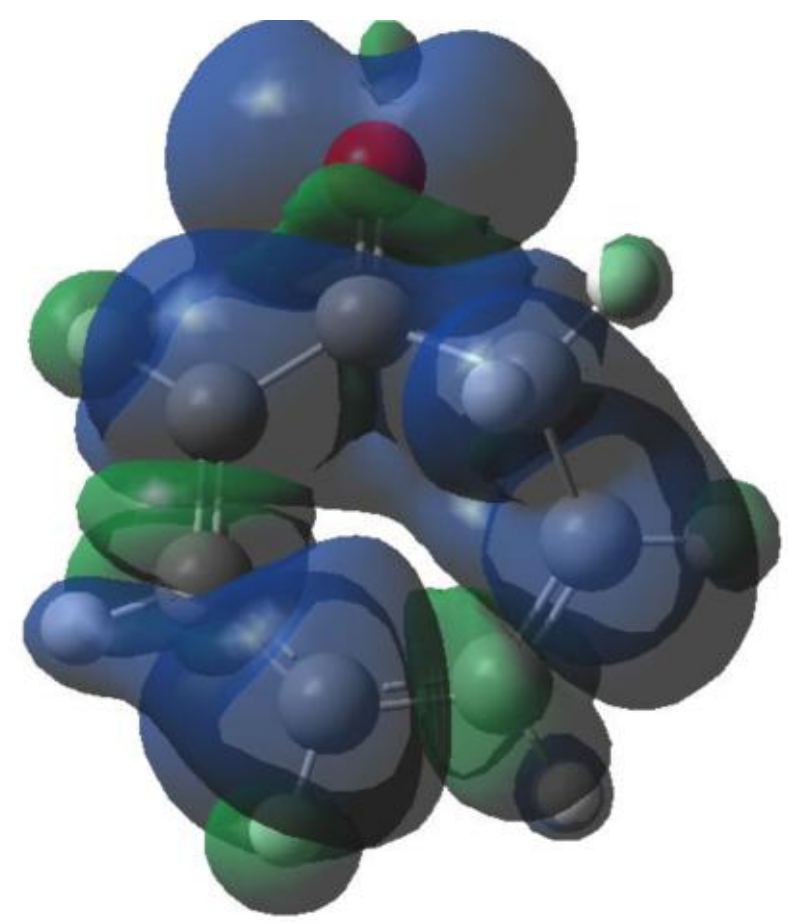
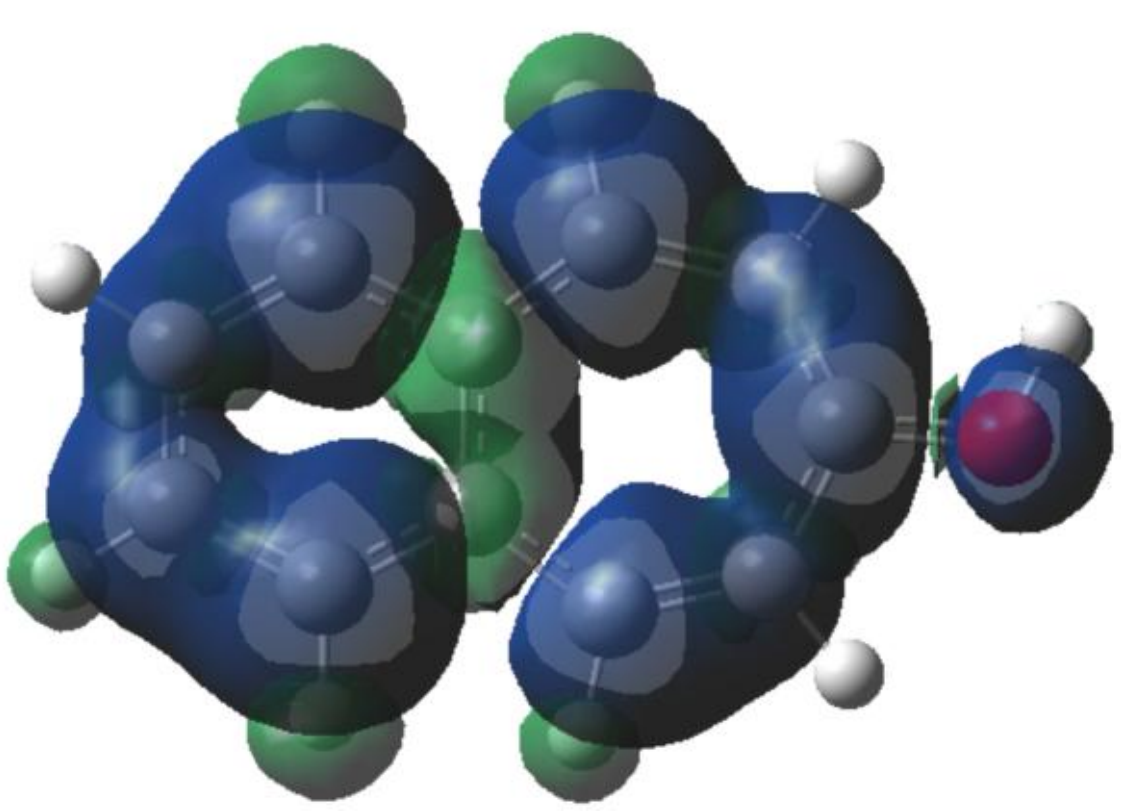	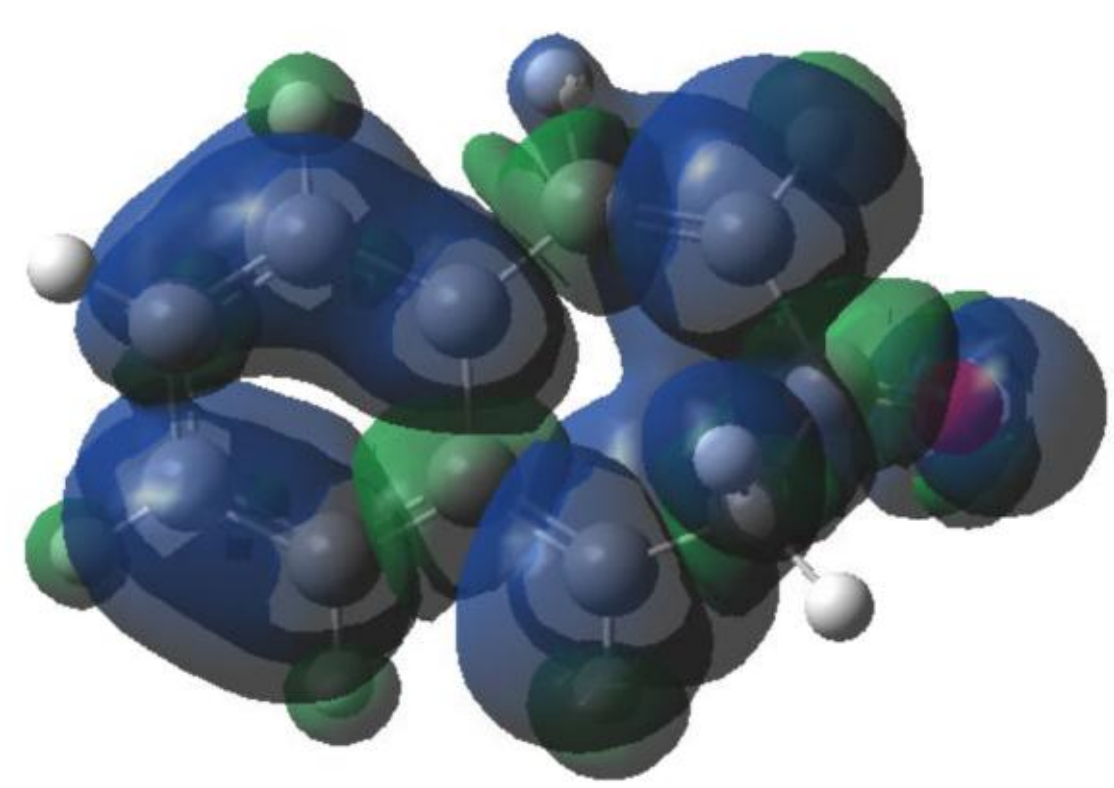

Table S12. HOMA [(U)ωB97XD/6-31G(d)] and NICS$_{zz}$(1) (ppm) (*italics*) [S_0, T_1: (U)ωB97XD/6-311+G(d,p)//(U)ωB97XD/6-31G(d); S_1: CASSCF*/6-311++G(d,p)//TD-ωB97XD/6-31G(d)] of model compounds before (E) and after (K) proton transfer in the S_0, T_1, ans S_1 states.

**Species**	**S_0** E	**S_0** K	**T_1** E	**T_1** K	**S_1** E	**S_1** K
Phenol	0.993<br>*-27.3*	-1.021<br>*1.3*	0.722<br>*26.1*	-0.681<br>*2.4*	0.736<br>60.5	-0.606<br>17.2
**TP**	0.987<br>*-26.7*	–	0.478<br>*17.4*	–	0.787	–
**HT**	0.907<br>*-21.3*	-0.384<br>*-0.03*	0.347<br>*61.2*	-0.49<br>*0.4*	0.653<br>63.6	-0.696<br>23.9
Benzotropylium (TP ring)	0.832<br>*-23.9*	–	0.801<br>*45.9*	–	0.771	–
Benzotropylium (benzene ring)	0.728<br>*-33.8*	–	0.677<br>*34.4*	–	0.788	–
Benzotropylium (perimeter)	0.885	–	0.778	–	0.757	–
**HBT** (TP ring)	0.742<br>*-16.1*	-0.713<br>*4.4*	0.759<br>*52.4*	-0.551<br>*11.2*	0.697<br>59.3	-1.306<br>22.5
**HBT** (benzene ring)	0.840<br>*-32.6*	0.706<br>*-15.9*	0.605<br>*52.0*	0.591<br>*26.5*	0.797<br>53.7	0.777<br>24.1
**HBT** (perimeter)	0.833	-0.07	0.689	0.028	0.715	-0.418

*phenol, **HT**, **TP**: 6in6; benzotropylium, **HBT**: 10in10.

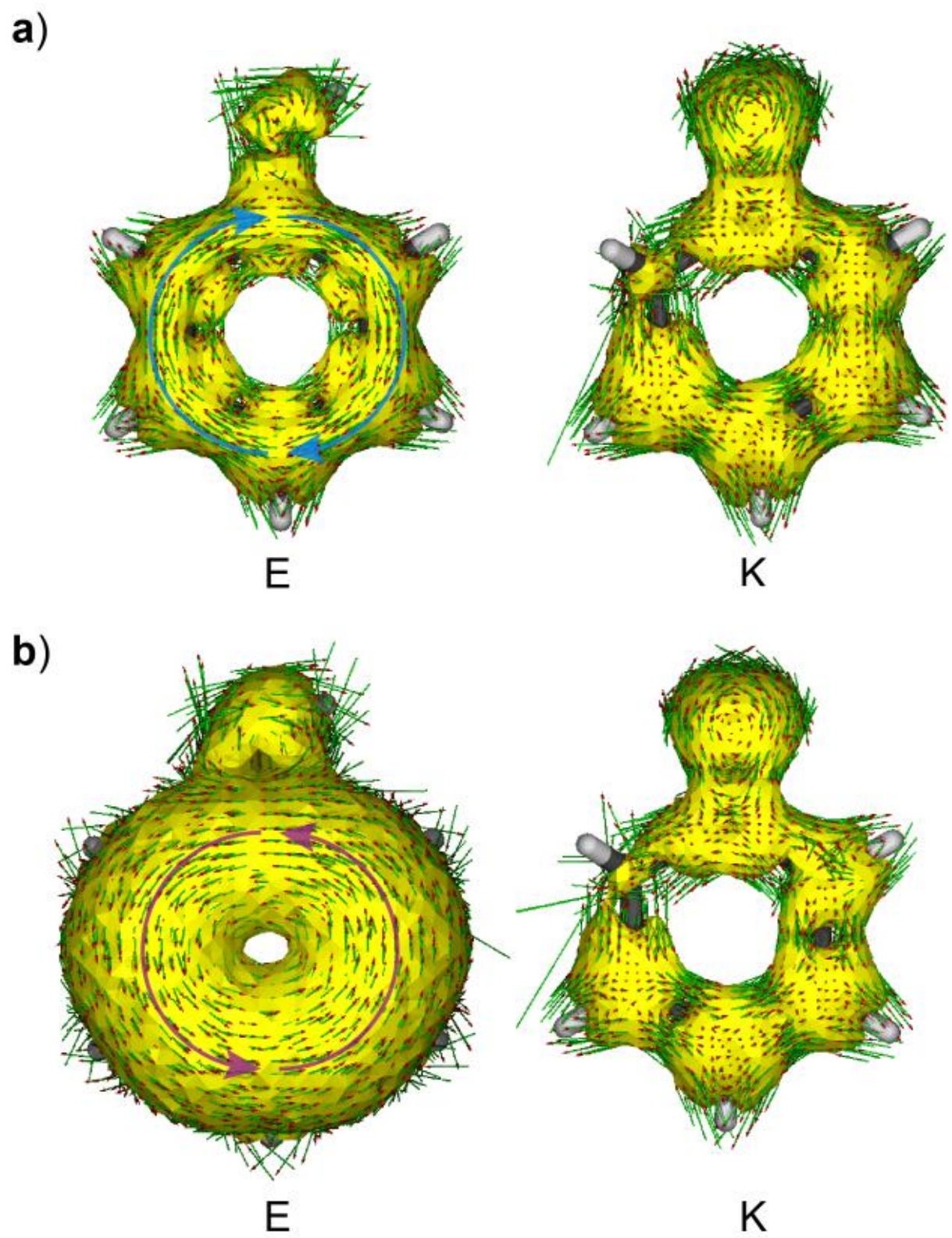

Fig. S37. AICD calculations of phenol before (E) and after (K) proton transfer in the **a**) S_0 and **b**) T_1 states [(U)ωB97XD/6-311+G(d,p)//(U)ωB97XD/6-31G(d)]. Blue and purple arrows represent the clockwise and anticlockwise direction of ring currents indicative of aromaticity and antiaromaticity, respectively.

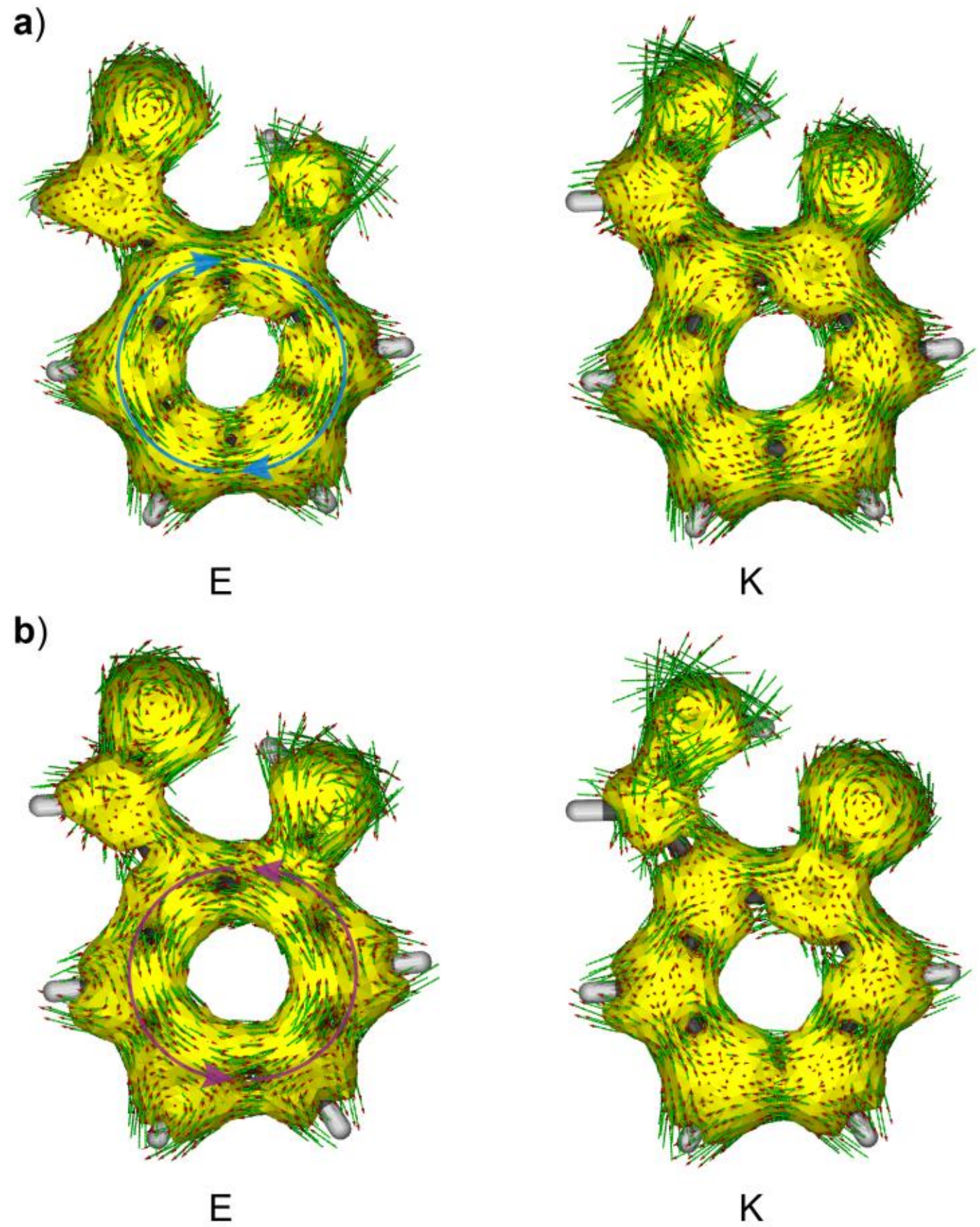

Fig. S38. AICD calculations of *o*-**SC** [(U)ωB97XD/6-311+G(d,p)//(U)ωB97XD/6-31G(d)] before (E) and after (K) proton transfer in the **a**) S_0 and **b**) T_1 states. Blue and purple arrows represent the clockwise and anticlockwise direction of ring currents indicative of aromaticity and antiaromaticity, respectively

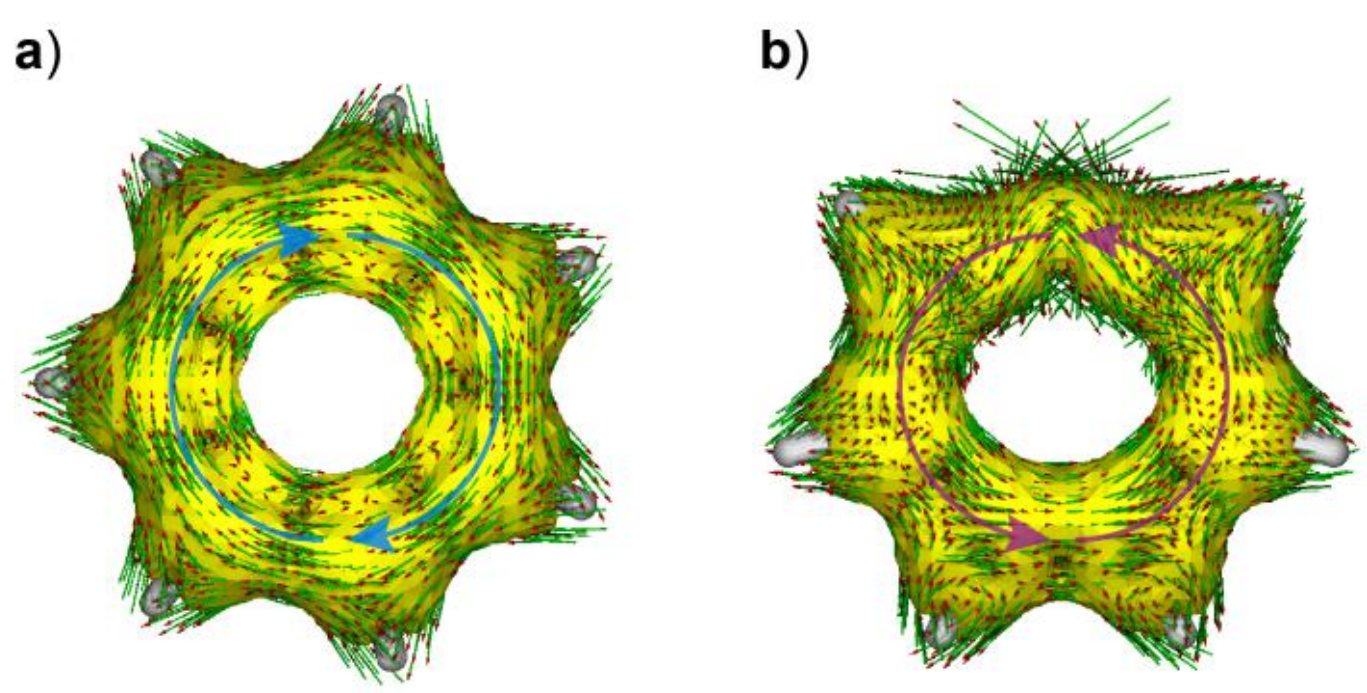

Fig. S39. AICD calculations of TP in the **a**) S_0 and **b**) T_1 states [(U)ωB97XD/6-311+G(d,p)//(U)ωB97XD/6-31G(d)]. Blue and purple arrows represent the clockwise and anticlockwise direction of ring currents indicative of aromaticity and antiaromaticity, respectively.

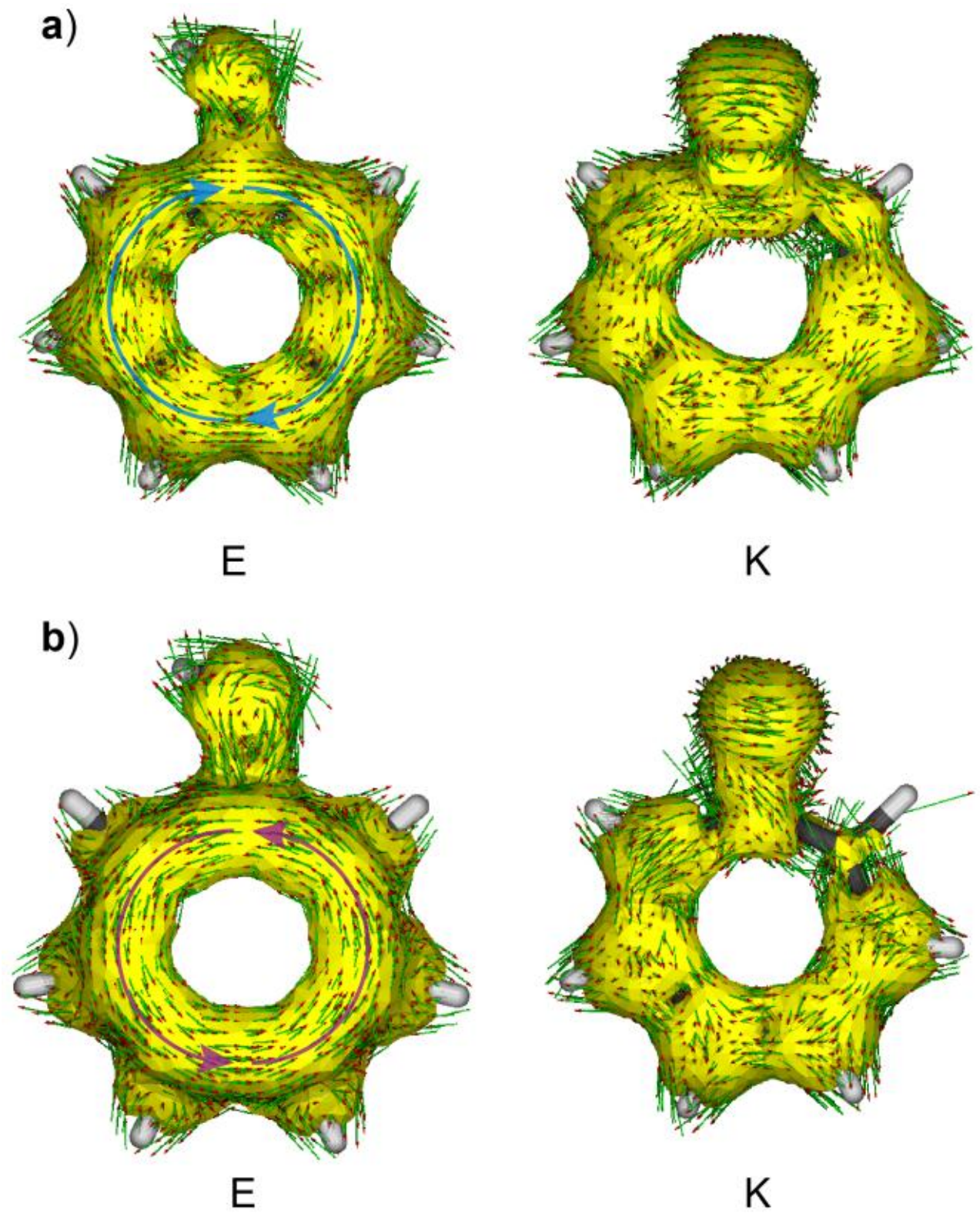

Fig. S40. AICD calculations of **HT** before (E) and after (K) proton transfer in the **a**) S_0 and **b**) T_1 states [(U)ωB97XD/6-311+G(d,p)//(U)ωB97XD/6-31G(d)]. Blue and purple arrows represent the clockwise and anticlockwise direction of ring currents indicative of aromaticity and antiaromaticity, respectively.

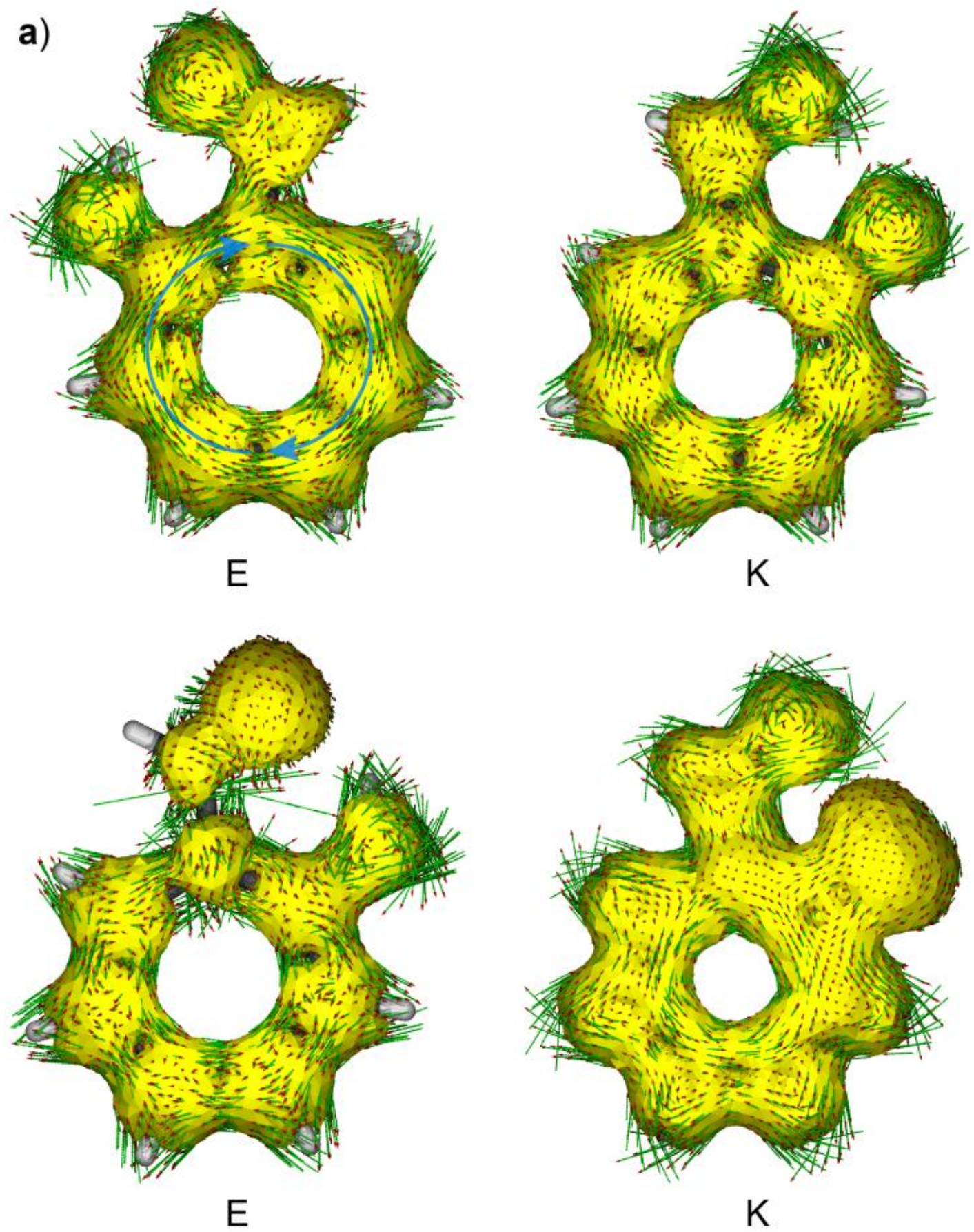

Fig. S41. AICD calculations of **1a** before (E) and after (K) proton transfer in the **a**) S_0 and **b**) T_1 states [(U)ωB97XD/6-311+G(d,p)//(U)ωB97XD/6-31G(d)]. Blue and purple arrows represent the clockwise and anticlockwise direction of ring currents indicative of aromaticity and antiaromaticity, respectively.

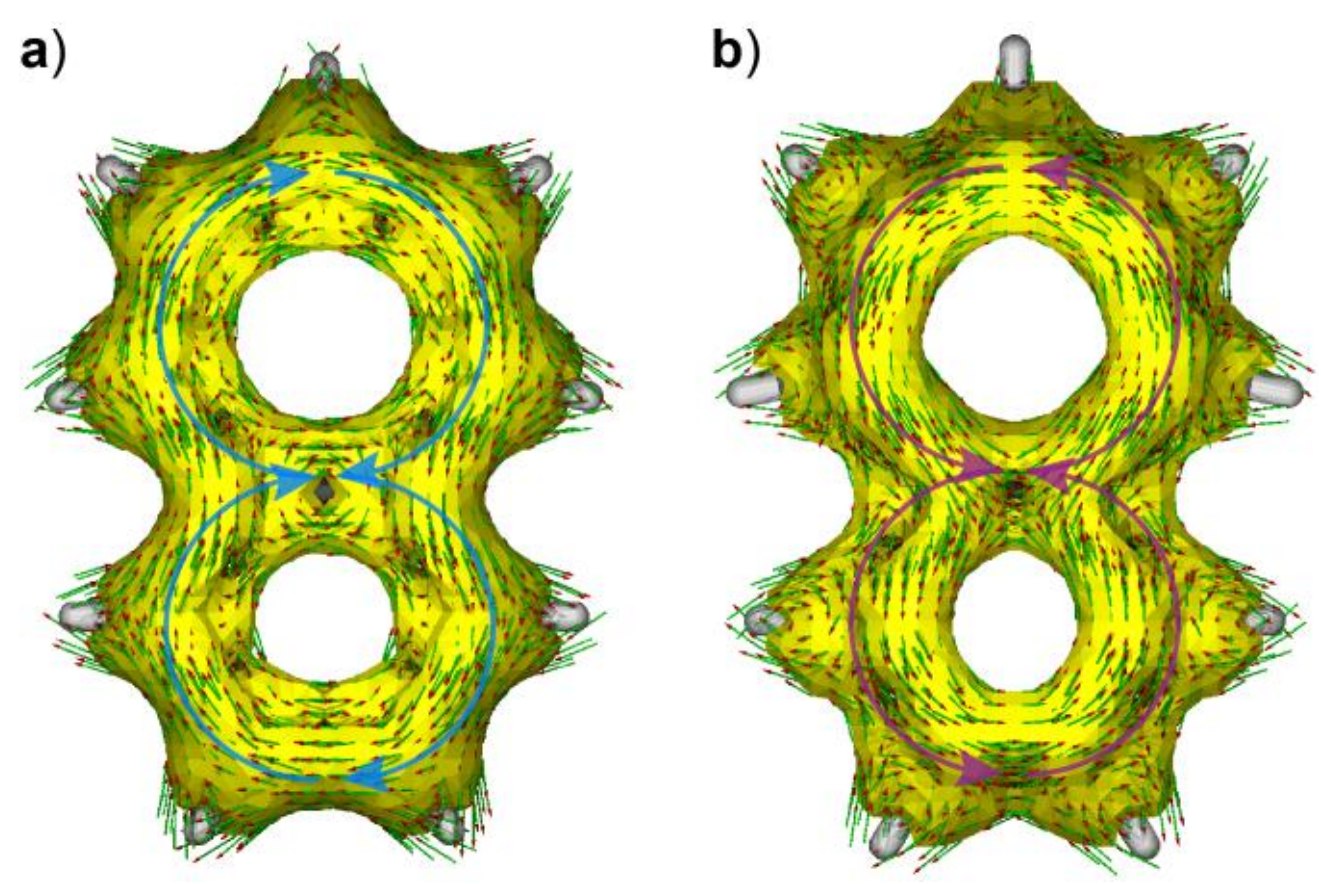

Fig. S42. AICD calculations of benzotropylium in the **a**) S_0 and **b**) T_1 states [(U)ωB97XD/6-311+G(d,p)]. Blue and purple arrows represent the clockwise and anticlockwise direction of ring currents indicative of aromaticity and antiaromaticity, respectively.

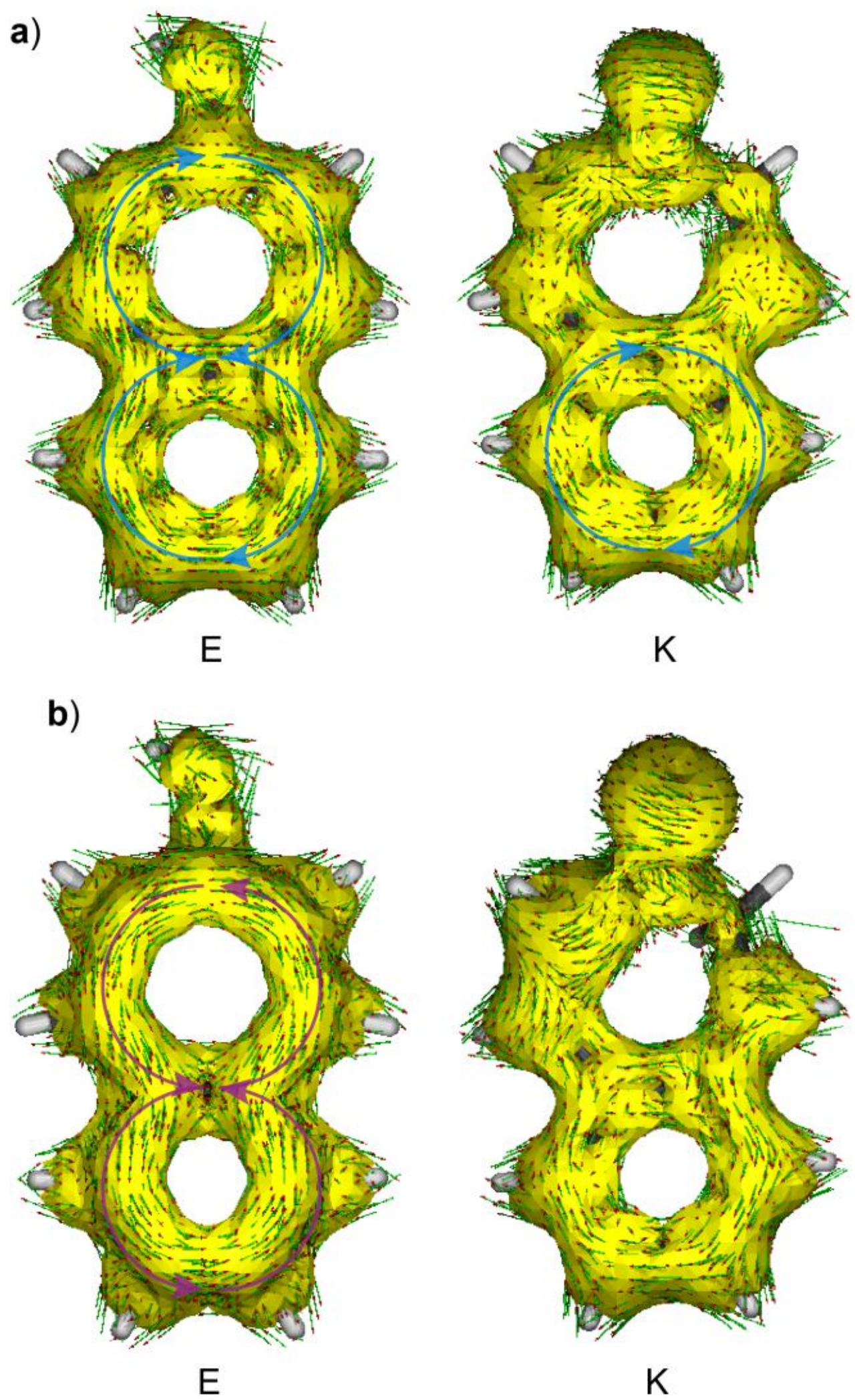

Fig. S43. AICD calculations of **HBT** before (E) and after (K) proton transfer in the **a**) S_0 and **b**) T_1 states [(U)ωB97XD/6-311+G(d,p)//(U)ωB97XD/6-31G(d)]. Blue and purple arrows represent the clockwise and anticlockwise direction of ring currents indicative of aromaticity and antiaromaticity, respectively.

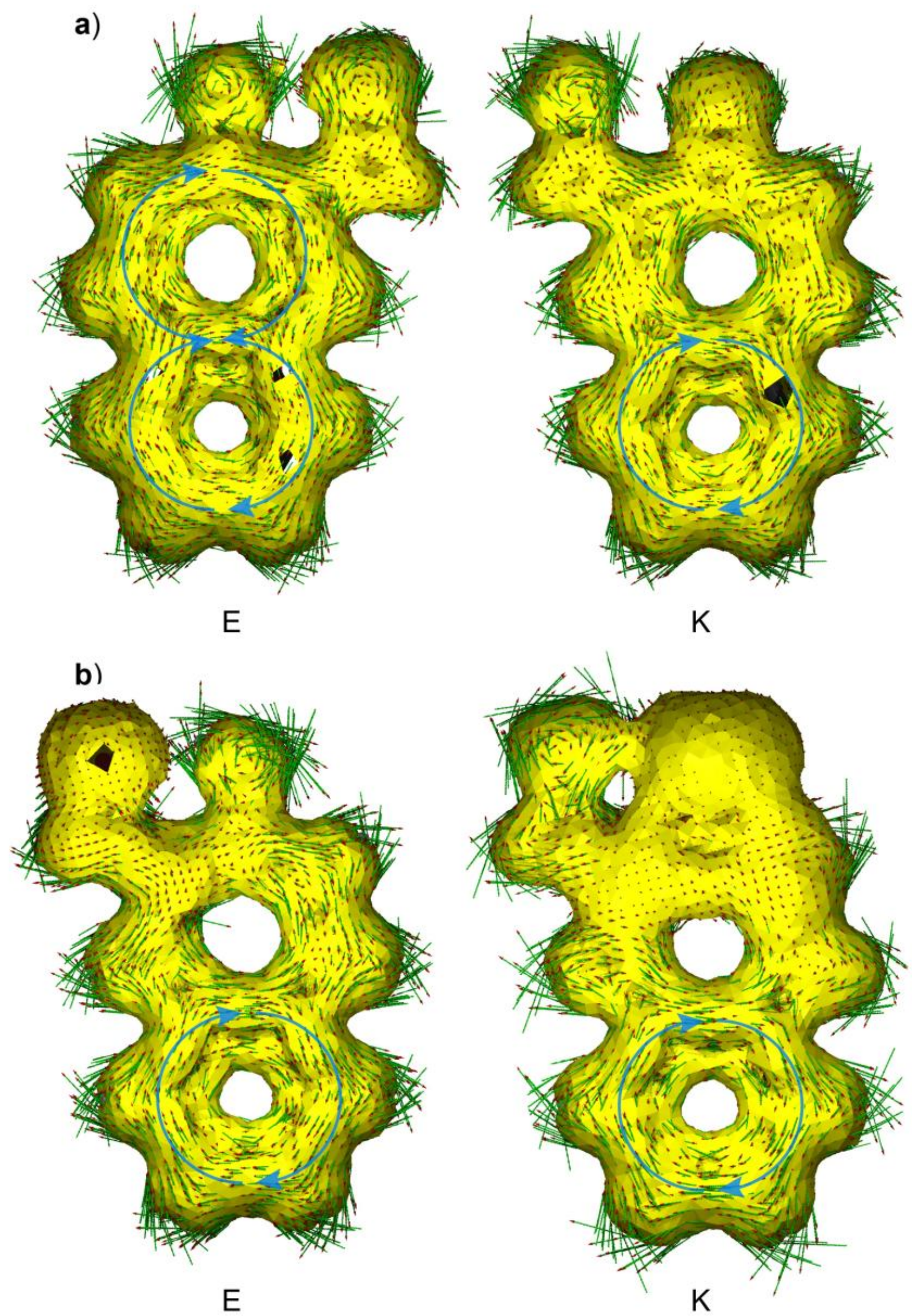

Fig. S44. AICD calculations of **2a** before (E) and after (K) proton transfer in the **a**) S_0 and **b**) T_1 states [(U)ωB97XD/6-311+G(d,p)//(U)ωB97XD/6-31G(d)]. Blue and purple arrows represent the clockwise and anticlockwise direction of ring currents indicative of aromaticity and antiaromaticity, respectively.

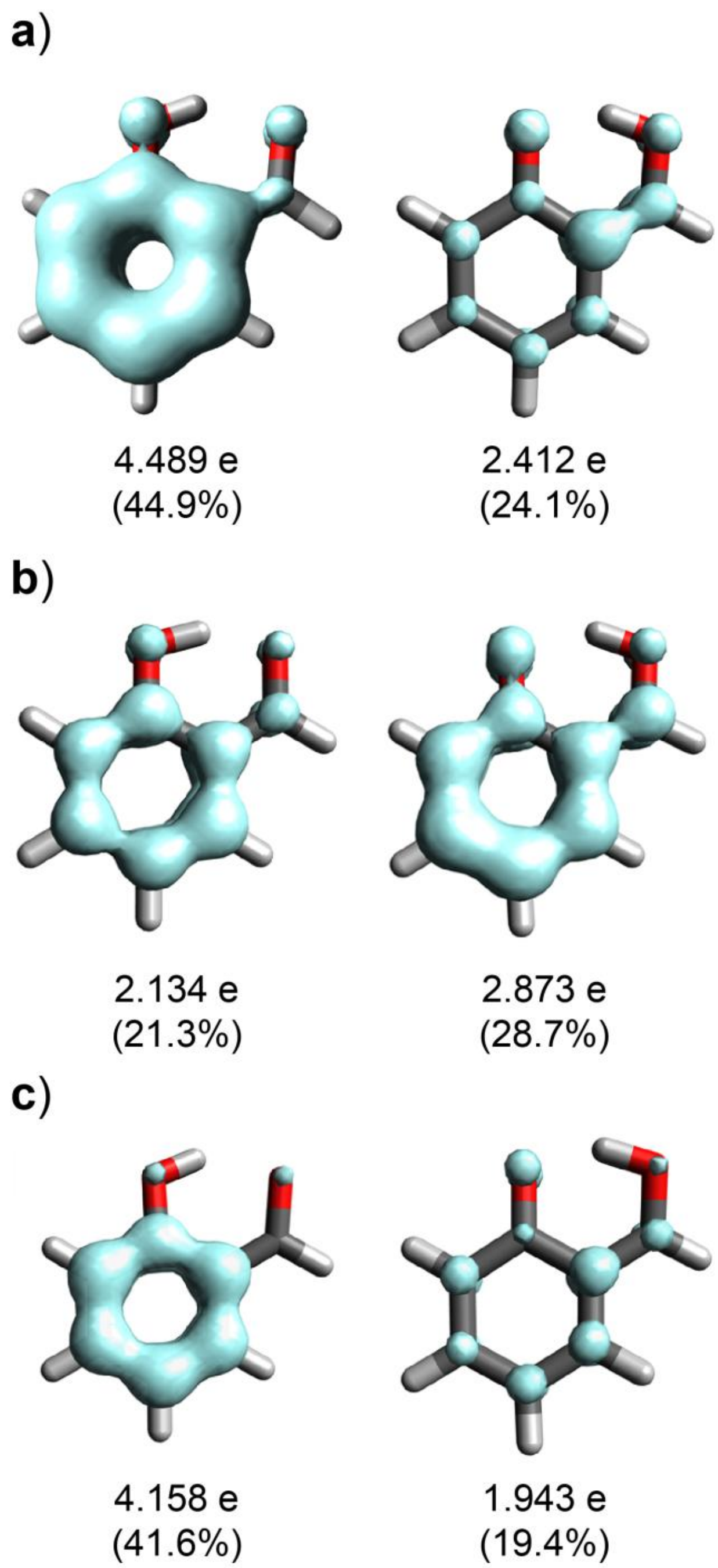

Fig. S45. EDDB$_P$ of *o*-**SC** before (E, left) and after (K, right) proton transfer in the **a**) S_0, **b**) T_1, and **c**) S_1 states. [S_0,T_1: (U)ωB97XD/6-311+G(d,p)//(U)ωB97XD/6-31G(d)]; S_1: CASSCF(10,10)/def2-SVP//TD-ωB97XD/6-31G(d) single point].

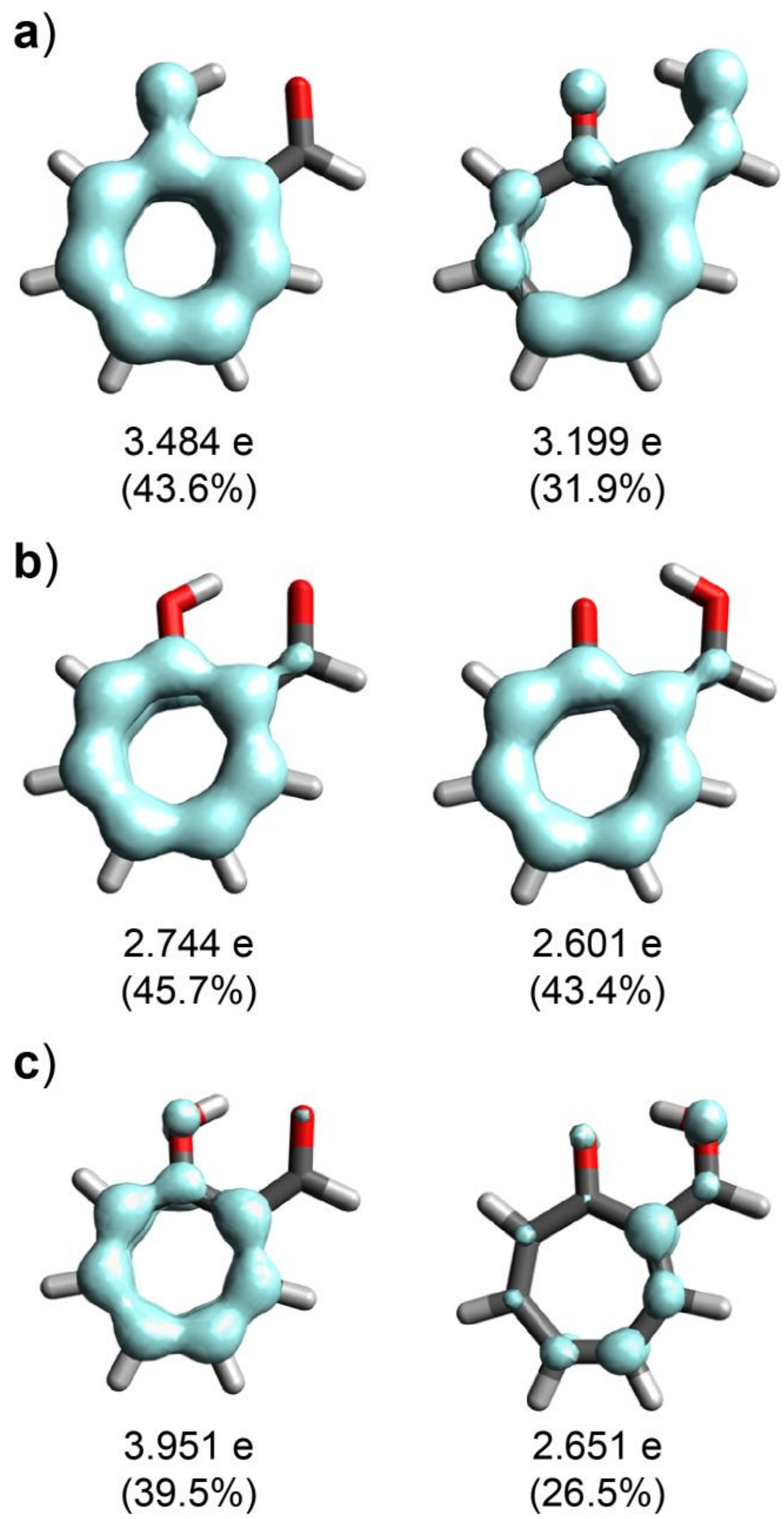

Fig. S46. EDDB$_P$ of **1a** [(U)ωB97XD/6-311+G(d,p)] before (E, left) and after (K, right) proton transfer in the **a**) S_0, **b**) T_1, and **c**) S_1 states [S_0,T_1: (U)ωB97XD/6-311+G(d,p)//(U)ωB97XD/6-31G(d)]; S_1: CASSCF(10,10)/def2-SVP//TD-ωB97XD/6-31G(d) single point].

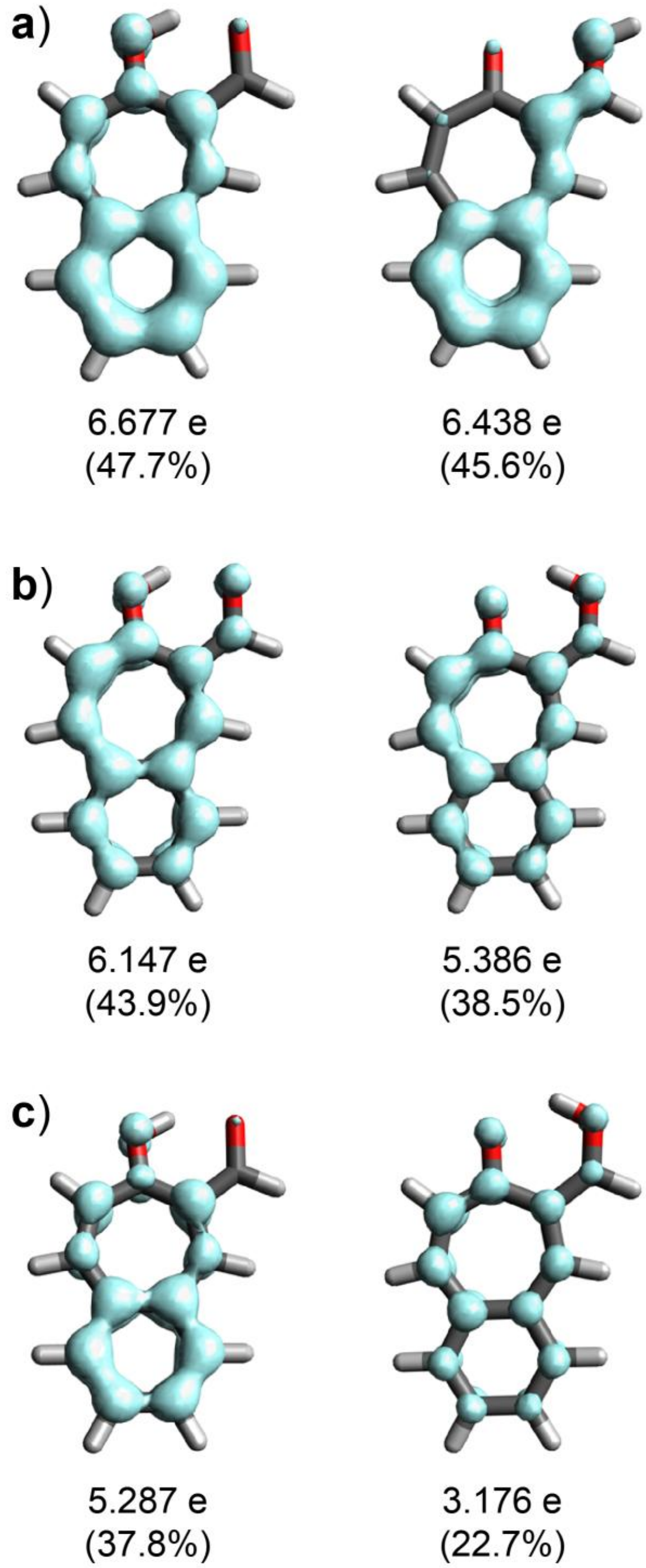

Fig. S47. $EDDB_P$ of **2a** before (E, left) and after (K, right) proton transfer in the **a**) S_0, **b**) T_1, and **c**) S_1 states. [S_0,T_1: (U)ωB97XD/6-311+G(d,p)//(U)ωB97XD/6-31G(d)]; S_1: CASSCF(14,14)/def2-SVP//TD-ωB97XD/6-31G(d) single point].

Conformational flexibility of HBTs

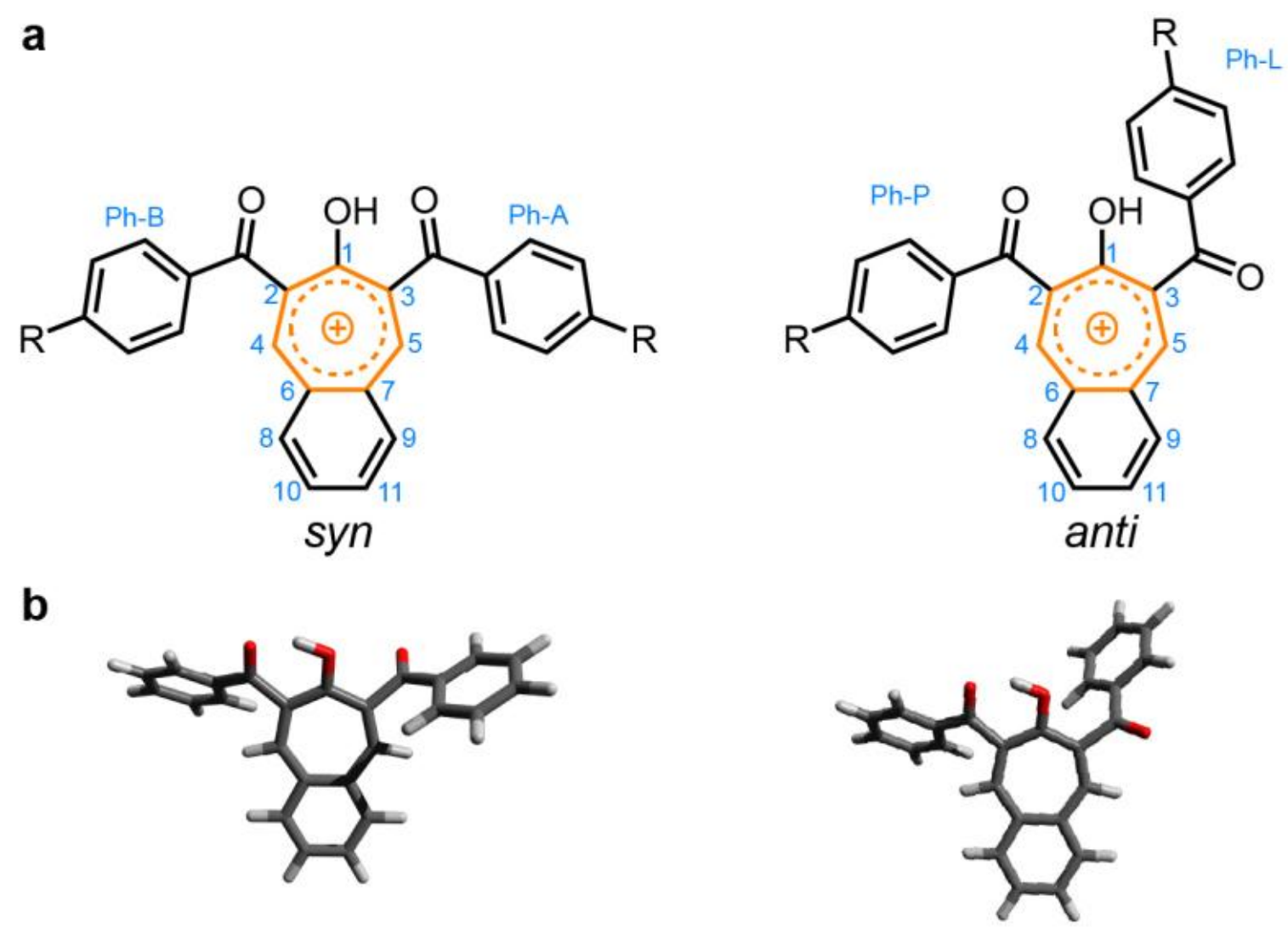

Fig. S48. (a) Conformations of **4b–d;** (b) DFT models of **4b** in the *syn*- and *anti*- conformations [(U)ωB97XD/6-31G(d)].

Our calculations revealed that HBTs **4b**–**d** show two different conformations (Fig. S48). In the *syn*-arrangement, the two appended carbonyl groups point in the same direction, while in the *anti*-arrangement, they point in opposite directions. In the S_0 ground-state, both isomers are nearly degenerate (Table S13). Importantly, the aromatic characters of both conformations are similar, as determined by EDDB calculations (Fig. S55–Fig. S56 and Fig. S60–Fig. S61). Since both species exist in a roughly equimolar ratio in solution in the ground state, photoexcitation is likely to reproduce this distribution of conformers in the S_1 excited state. E→K tautomerization remains endergonic in both conformations (i.e., $\Delta G_T^* < 0$), so we still expect to observe ESPT in both cases.

However, in the S_1 state, **4b** and **4c** favor the *syn*-conformation by 24 kJ·mol^{-1} and 23 kJ·mol^{-1} respectively, while **4d** favors the *anti*-conformation by 12 kJ·mol^{-1}. To eliminate conformational relaxation as the origin of the large Stokes shift observed for these compounds, we recorded time-resolved fluorescence spectra in frozen solutions (Fig. S27d and Fig. S32d). While aggregation effects complicate the analysis of **4b** (see discussion above), we find that at 80 K (where conformational flexibility is suppressed), **4c** still shows a prominent Stokes shift, as well as a gradual redshifting of the emission maximum over several ns.

Table S13. Relative energies of the *syn*- and *anti*-conformations of the E and K tautomers of **4b–d** in the S_0 and S_1 states [S_0, T_1: (U)ωB97XD/6-31G(d); S_1: TD-ωB97XD/6-31G(d)].

**Compound**	**S_0**		**S_1**	
	G_{rel} (kJ·mol^{-1})	ΔG_T (kJ·mol^{-1})	G_{rel} (kJ·mol^{-1})	ΔG_T* (kJ·mol^{-1})
syn-E-**4b**	0	12.1	264	−6.27
syn-K-**4b**	12.1		258	
anti-E-**4b**	0.887	10.8	288	–1.26
anti-K-**4b**	11.7		287	
syn-E-**4c**	0	14.6	273	−9.62
syn-K-**4c**	14.6		263	
anti-E-**4c**	0.548	11.6	296	−7.95
anti-K-**4c**	12.1		288	
syn-E-**4d**[a]	0	8.79	210	–16.3
syn-K-**4d**	8.79		194	
anti-E-**4d**	1.55	4.06	198	−23.4
anti-K-**4d**	5.61		175	

Harmonic Oscillator Model of Aromaticity (HOMA)

It is found that electronic changes upon excitation are localized at the tropylium center. When a HBT molecule is excited from its S_0 to its S_1/T_1 state, significant changes in the aromaticity of central tropylium take place which determine the likelihood of ESPT. Additionally, HOMA values of planar and perpendicularly substituted phenyl rings showed high degree of aromaticity in all the compounds. HOMA values of each individual ring in the ground and excited states are shown below in Table S14–Table S18.

Table S14. HOMA values of the **tropylium** ring of compounds **4b–d** studied in their S_0, T_1, and S_1 states [S_0, T_1: (U)ωB97XD/6-31G(d); S_1: TD-ωB97XD/6-31G(d)].

Species	E (S_0)	K (S_0)	E (T_1)	K (T_1)	E (S_1)	K (S_1)
**4b**	0.615	0.223	0.621	0.456	0.671	0.459
**4c**	0.624	0.252	0.624	0.449	0.669	0.458
**4d**	0.611	0.177	0.572	0.457	0.655	0.312

Table S15. HOMA values of the benzene ring of compounds **4b–d** studied in their S_0, T_1, and S_1 states [S_0, T_1: (U)ωB97XD/6-31G(d); S_1: TD-ωB97XD/6-31G(d)].

Species	E (S_0)	K (S_0)	E (T_1)	K (T_1)	E (S_1)	K (S_1)
**4b**	0.880	0.903	0.655	0.608	0.631	0.626
**4c**	0.879	0.899	0.658	0.615	0.630	0.632
**4d**	0.880	0.907	0.769	0.605	0.608	0.634

Table S16. HOMA values of the appended aryl groups in a planar conformation of compounds **4b–d** in their S_0, T_1, and S_1 states [S_0, T_1: (U)ωB97XD/6-31G(d); S_1: TD-ωB97XD/6-31G(d)].

Species	E (S_0)	K (S_0)	E (T_1)	K (T_1)	E (S_1)	K (S_1)
**4b**	0.979	0.964	0.983	0.985	0.984	0.978
**4c**	0.973	0.960	0.976	0.973	0.945	0.972
**4d**	0.951	0.889	0.979	0.954	0.975	0.964

Table S17. HOMA values of the appended aryl groups in a planar conformation in compounds **4b–d** in their S_0, T_1, and S_1 states [S_0, T_1: (U)ωB97XD/6-31G(d); S_1: TD-ωB97XD/6-31G(d)].

Species	E (S_0)	K (S_0)	E (T_1)	K (T_1)	E (S_1)	K (S_1)
**4b**	0.987	0.989	0.978	0.979	0.953	0.987
**4c**	0.979	0.981	0.971	0.978	0.978	0.980
**4d**	0.966	0.978	0.980	0.960	0.882	0.916

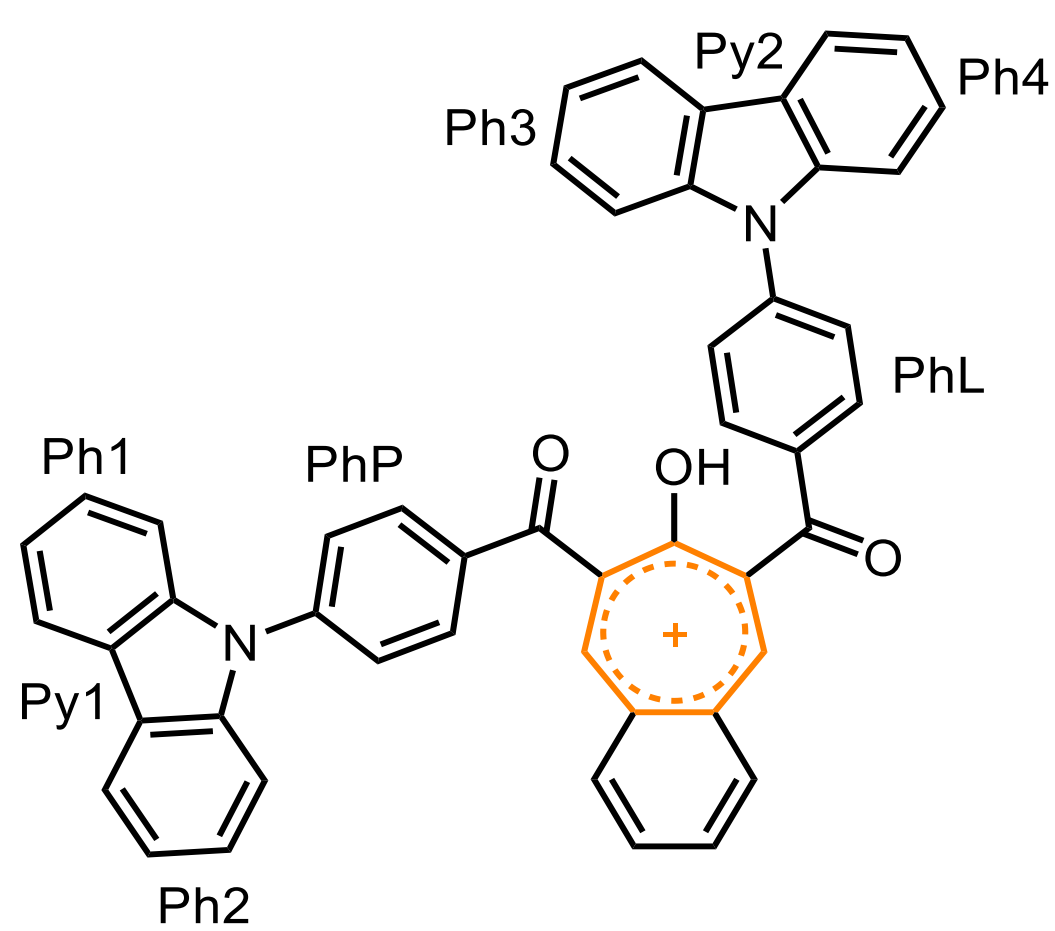

Table S18. HOMA values of the carbazole (Cbz) groups in **4d** in the S_0, T_1, and S_1 states [S_0, T_1: (U)ωB97XD/6-31G(d); S_1: TD-ωB97XD/6-31G(d)].

Species	Planar Cbz			Perpendicular Cbz		
	Ph1	Ph2	Py 1	Ph3	Ph4	Py 2
E S_0	0.978	0.978	0.566	0.975	0.975	0.589
K S_0	0.981	0.981	0.524	0.974	0.973	0.600
E T_1	0.946	0.945	0.520	0.969	0.971	0.618
K T_1	0.977	0.977	0.575	0.977	0.977	0.574
E S_1	0.979	0.980	0.507	0.974	0.974	0.599
K S_1	0.975	0.975	0.589	0.979	0.978	0.528

Nucleus-Independent Chemical Shifts (NICS)

The magnetic aromaticity indicator $NICS_{zz}(1)$ was calculated in different rings of compounds studied in this work (Table S19–Table S23). The changes in the aromatic characters of the tropylium and benzene rings of the HBT core of **4b**–**d** are discussed in the main text. An analysis of the $NICS_{zz}(1)$ values of these rings showed that regardless of whether the appended phenyl or carbazole groups were planar or perpendicular to HBT, negligible changes in aromaticity occurred as a result of proton transfer in the S_0 and T_1 states.

Table S19. $NICS_{zz}(1)$ values (ppm) of the TP ring of **4b–d** [S_0, T_1: (U)ωB97XD/6-311+G(d,p)//(U)ωB97XD/6-31G(d); S_1: CASSCF(10,10)/6-311++G(d,p)//TD-ωB97XD/6-31G(d).

**Species**	**E (S_0)**	**K (S_0)**	**E (T_1)**	**K (T_1)**	**E (S_1)**	**K (S_1)**
**4b**	-12.6	-5.5	16.0	13.3	50.3	10.9
**4c**	-12.1	-5.8	14.3	12.4	49.4	9.7
**4d**	-9.6	1.9	-2.1	-1.8	47.2	9.3

Table S20. $NICS_{zz}(1)$ values (ppm) of the benzene ring of **4b–d** [S_0, T_1: (U)ωB97XD/6-311+G(d,p)//(U)ωB97XD/6-31G(d); S_1: CASSCF(10,10)/6-311++G(d,p)//TD-ωB97XD/6-31G(d).

**Species**	**E (S_0)**	**K (S_0)**	**E (T_1)**	**K (T_1)**	**E (S_1)**	**K (S_1)**
**4b**	-28.4	-26.1	-9.6	-10.1	-7.9	-8.6
**4c**	-25.8	-24.4	-11.5	-10.7	-8.3	-8.4
**4d**	-21.3	-21.9	-20.6	-24.7	-12.3	-14.3

Table S21. $NICS_{zz}(1)$ values (ppm) of the planar aryl rings of **4b–d** [S_0, T_1: (U)ωB97XD/6-311+G(d,p)//(U)ωB97XD/6-31G(d); S_1: CASSCF(10,10)/6-311++G(d,p)//TD-ωB97XD/6-31G(d).

**Species**	**E (S_0)**	**K (S_0)**	**E (T_1)**	**K (T_1)**
**4b**	-3.6	-4.9	-7.4	-7.3
**4c**	-1.8	-3.0	-4.7	-6.9
**4d**	-10.7	-11.0	-18.7	-15.7

Table S22. $NICS_{zz}(1)$ values (ppm) of the perpendicular aryl rings of **4b–d** [S_0, T_1: (U)ωB97XD/6-311+G(d,p)//(U)ωB97XD/6-31G(d); S_1: CASSCF(10,10)/6-311++G(d,p)//TD-ωB97XD/6-31G(d).

**Species**	**E (S_0)**	**K (S_0)**	**E (T_1)**	**K (T_1)**
**4b**	-15.6	-18.8	-13.6	-12.9
**4c**	-15.8	-18.6	-10.6	-12.4
**4d**	-17.9	-20.6	-14.9	-12.1

Table S23. $NICS_{zz}(1)$ values (ppm) of the carbazole ring of **4b–d** [S_0, T_1: (U)ωB97XD/6-311+G(d,p)//(U)ωB97XD/6-31G(d); S_1: CASSCF(10,10)/6-311++G(d,p)//TD-ωB97XD/6-31G(d).

**Species**	**Planar Cbz**			**Perpendicular Cbz**		
	**Benzene 1**	**Benzene 2**	**Pyrrole 1**	**Benzene 3**	**Benzene 4**	**Pyrrole 2**
S_0 (E)	-15.3	-14.4	-7.9	-24.3	-23.8	-12.6
S_0 (K)	-17.3	-19.2	-8.2	-21.4	-21.7	-11.6
T_1 (E)	-19.1	-19.7	-10.7	-25.2	-25.7	-14.3
T_1 (K)	-25.7	-25.1	-12.8	-26.1	-26.9	-15.5

Anisotropy of the Induced Current Density (AICD) Plots

a)

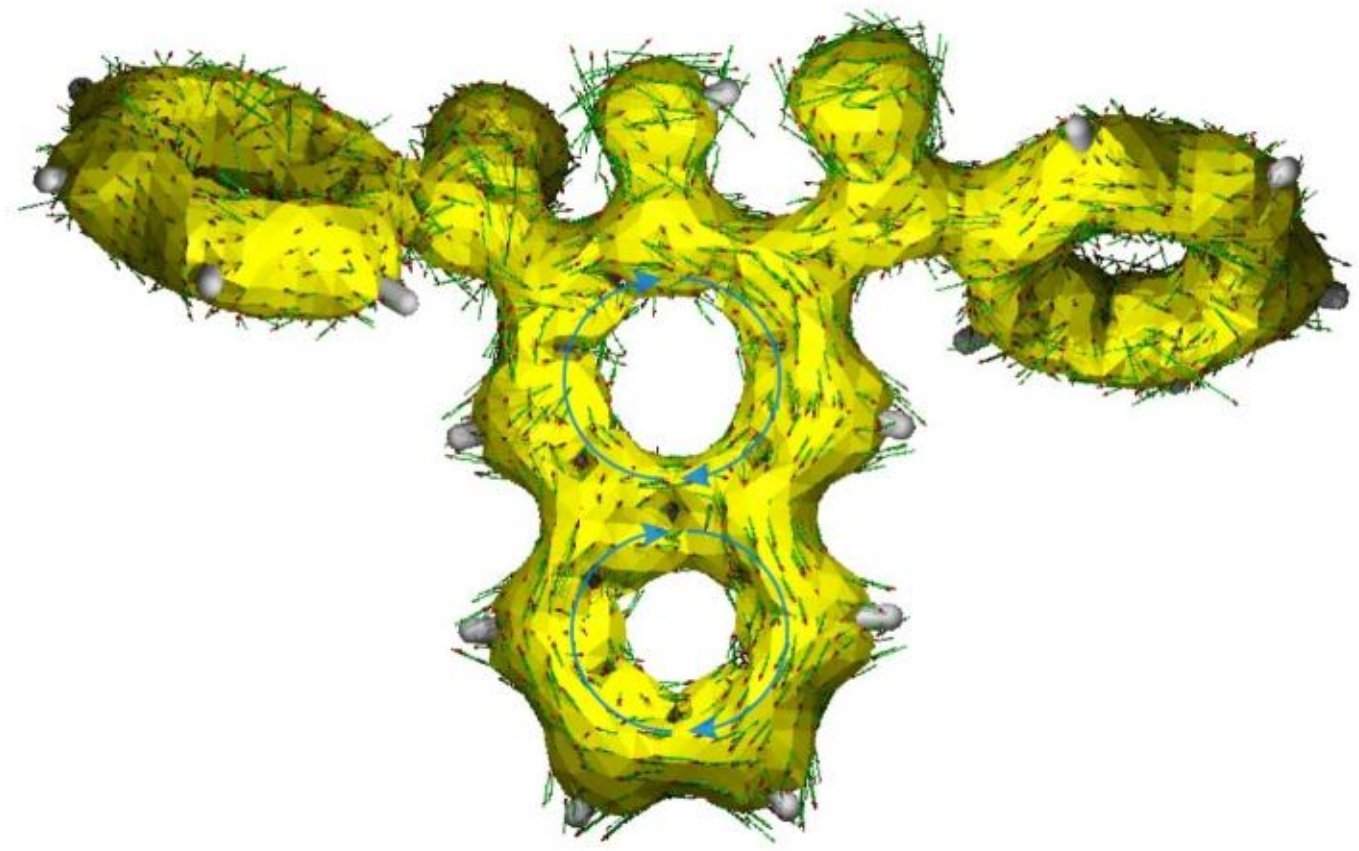

syn-E

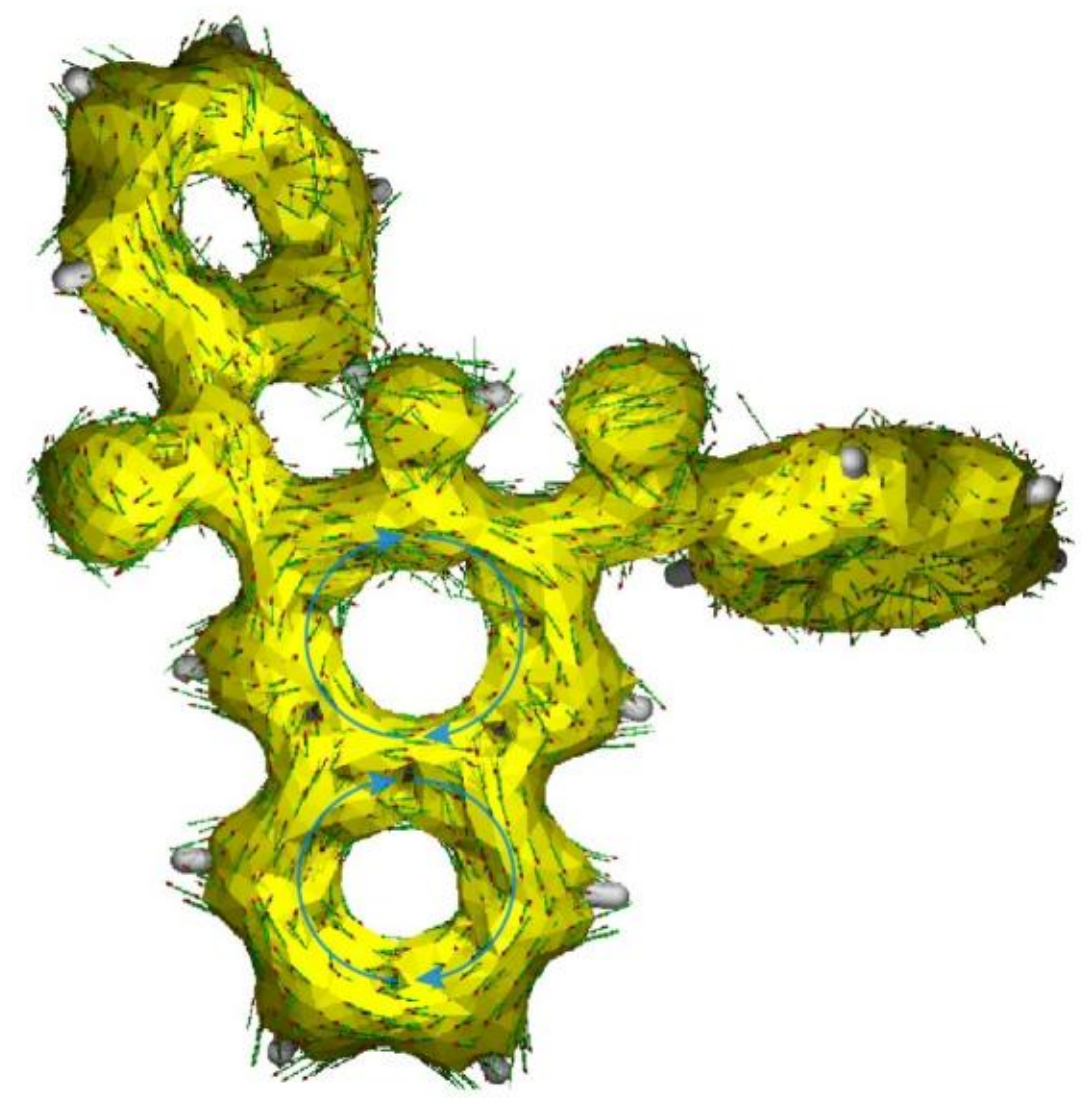

anti-E

b)

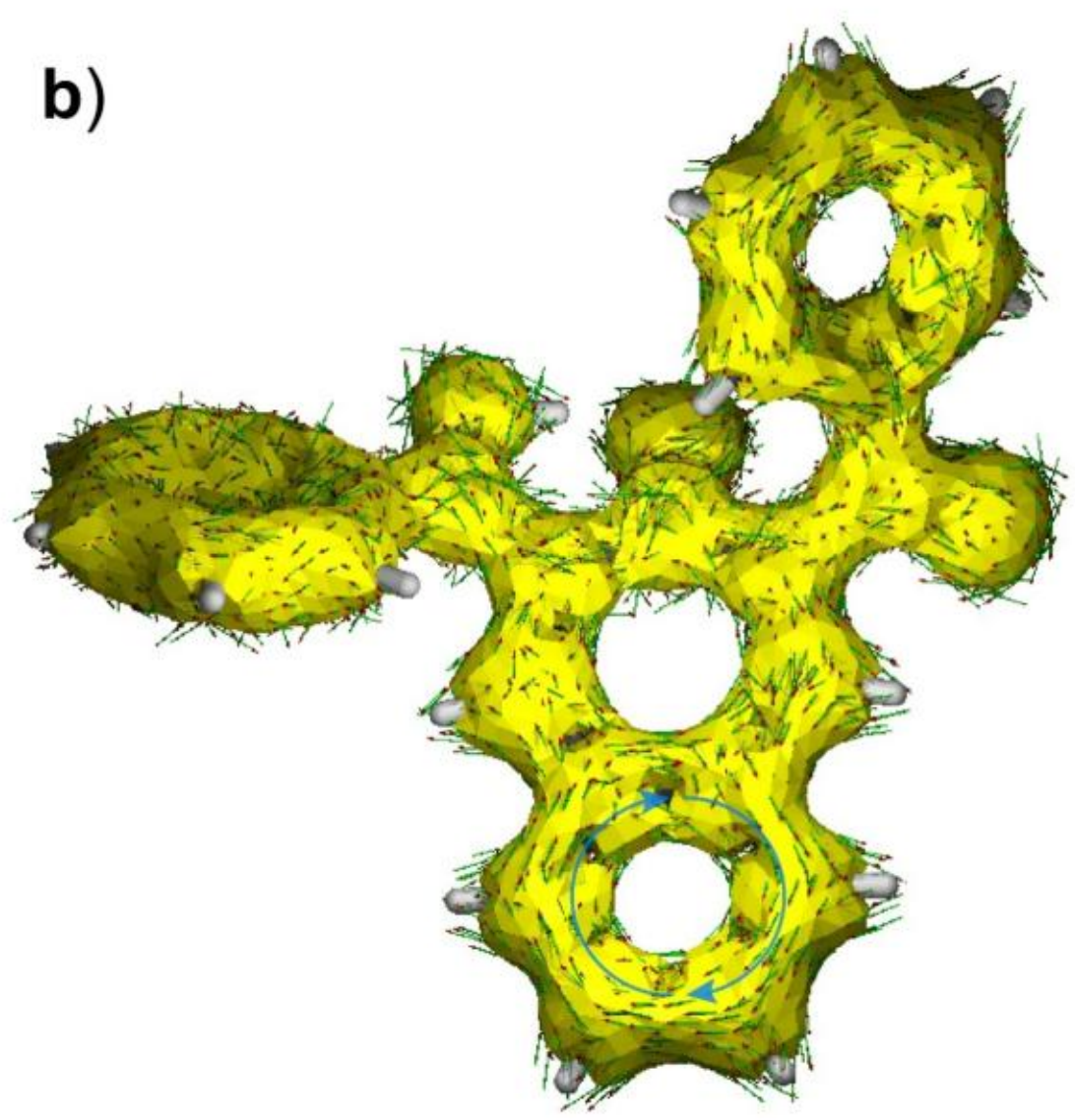

anti-K

Fig. S49. AICD (isosurface: 0.05) plots of **a**) *syn*- and *anti*-**4b** before (E) and **b**) *anti*-**4b** after (K) proton transfer in the S_0 state [ωB97XD/6-311+G(d,p)//ωB97XD/6-31G(d)]. Blue arrows represent the clockwise direction of ring currents indicative of aromaticity.

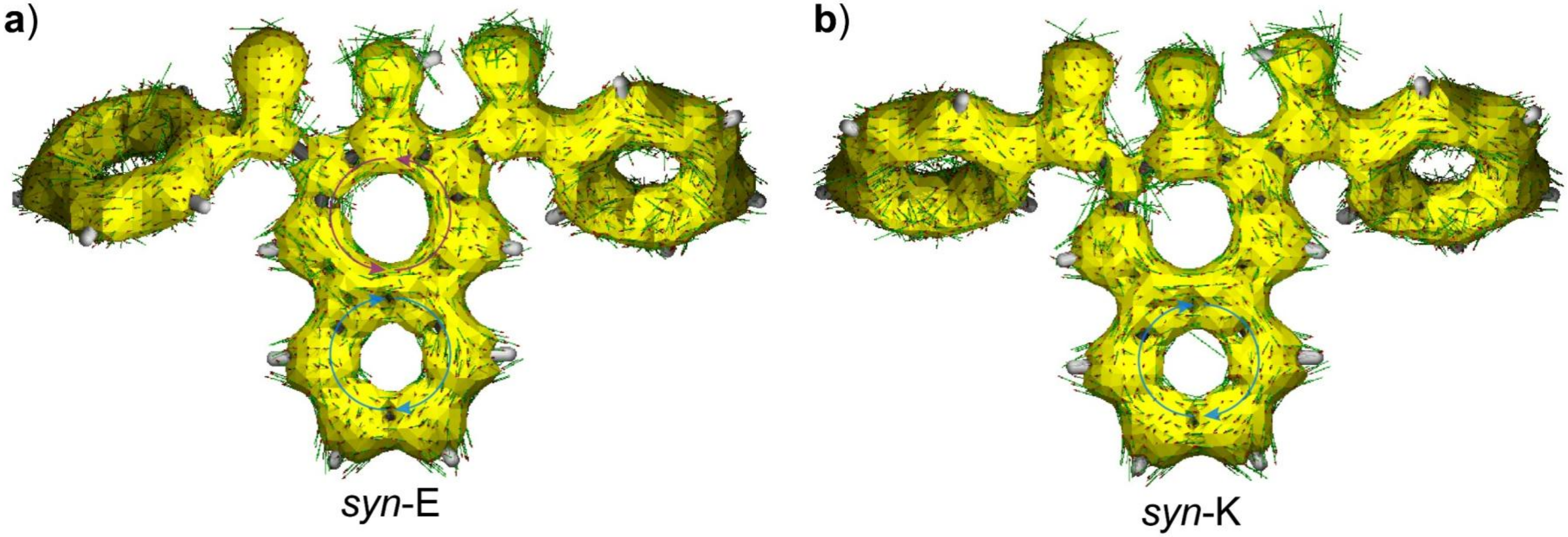

Fig. S50. AICD (isosurface: 0.05) plots of *syn*-**4b** in the T_1 state **a**) before (E) and **b**) after (K) proton transfer [(U)ωB97XD/6-311+G(d,p)//(U)ωB97XD/6-31G(d)]. Blue and purple arrows represent the clockwise and anticlockwise direction of ring currents indicative of aromaticity and antiaromaticity, respectively.

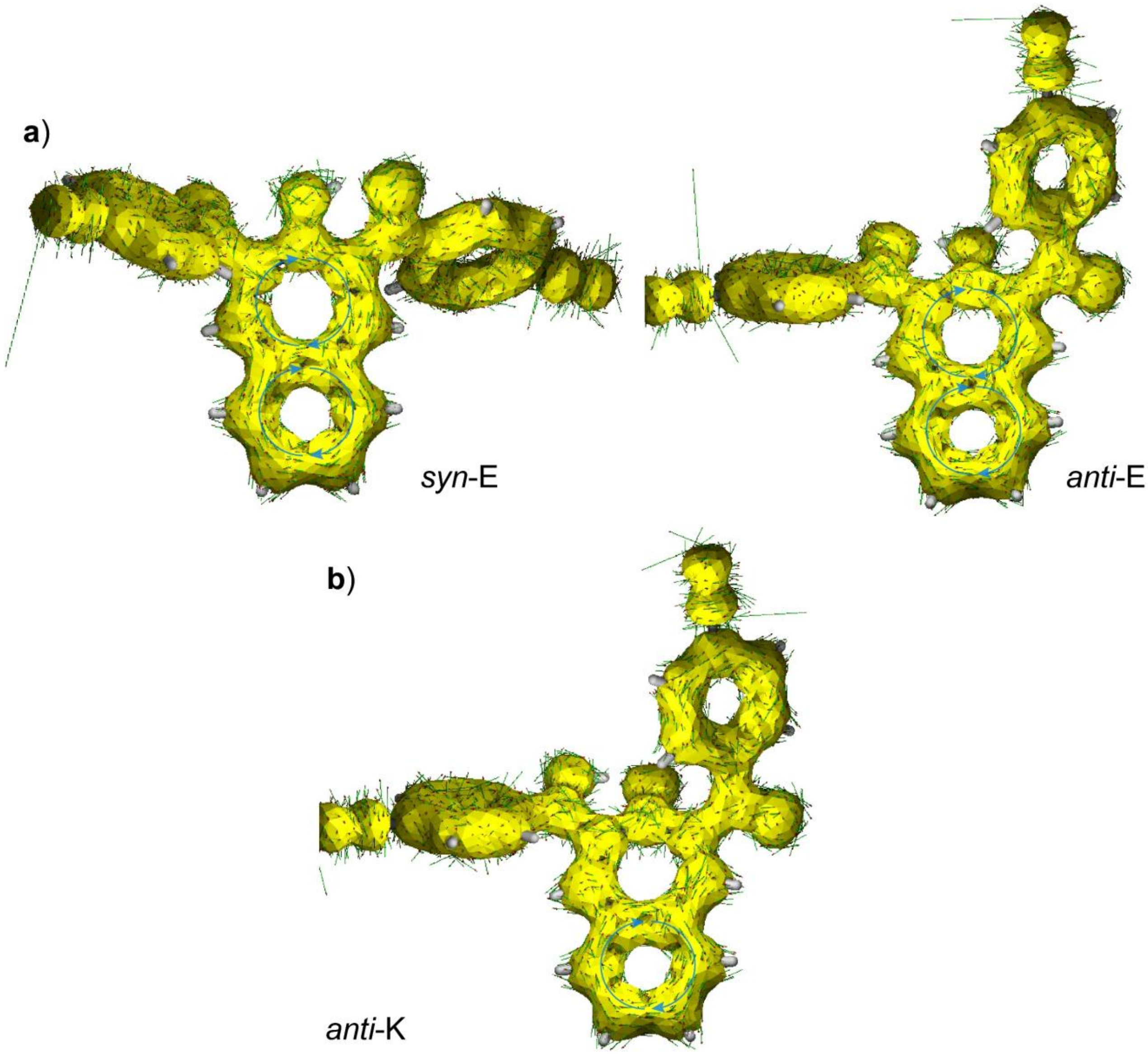

Fig. S51. AICD (isosurface: 0.05) plots of **a**) *syn*- and *anti*-**4c** before (E) and **b**) after (K) proton transfer in the S_0 state [ωB97XD/6-311+G(d,p)//ωB97XD/6-31G(d)]. Blue arrows represent the clockwise direction of ring currents indicative of aromaticity.

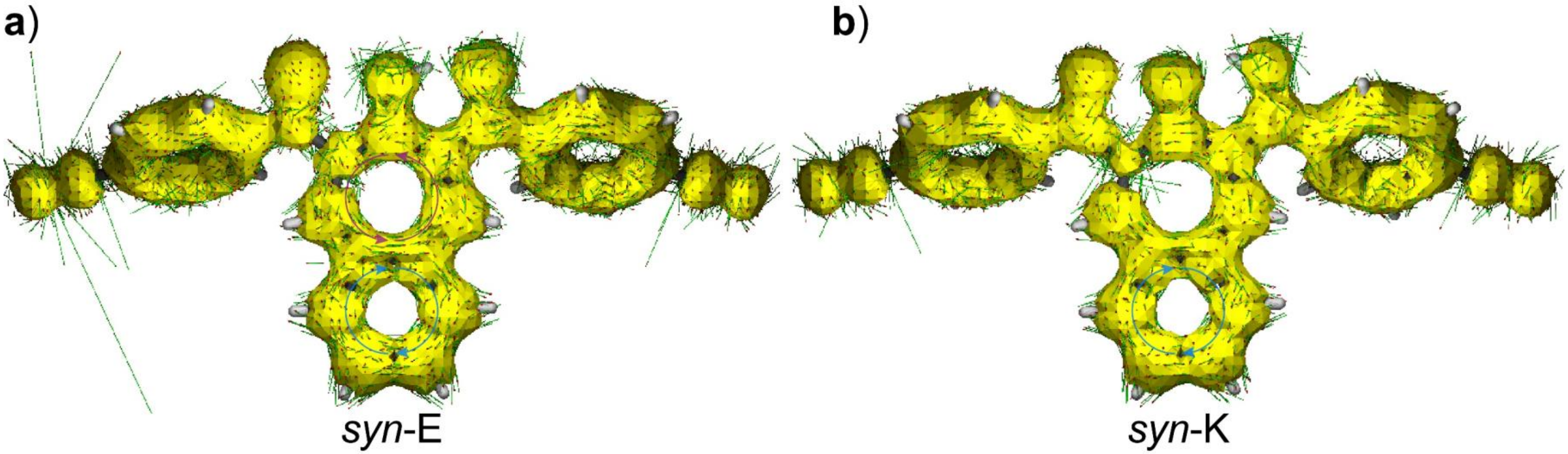

Fig. S52. AICD (isosurface: 0.05) plots of *syn*-**4c** in the T_1 state before (E) and after (K) proton transfer [(U)ωB97XD/6-311+G(d,p)//(U)ωB97XD/6-31G(d)]. Blue and purple arrows represent the clockwise and anticlockwise direction of ring currents indicative of aromaticity and antiaromaticity, respectively.

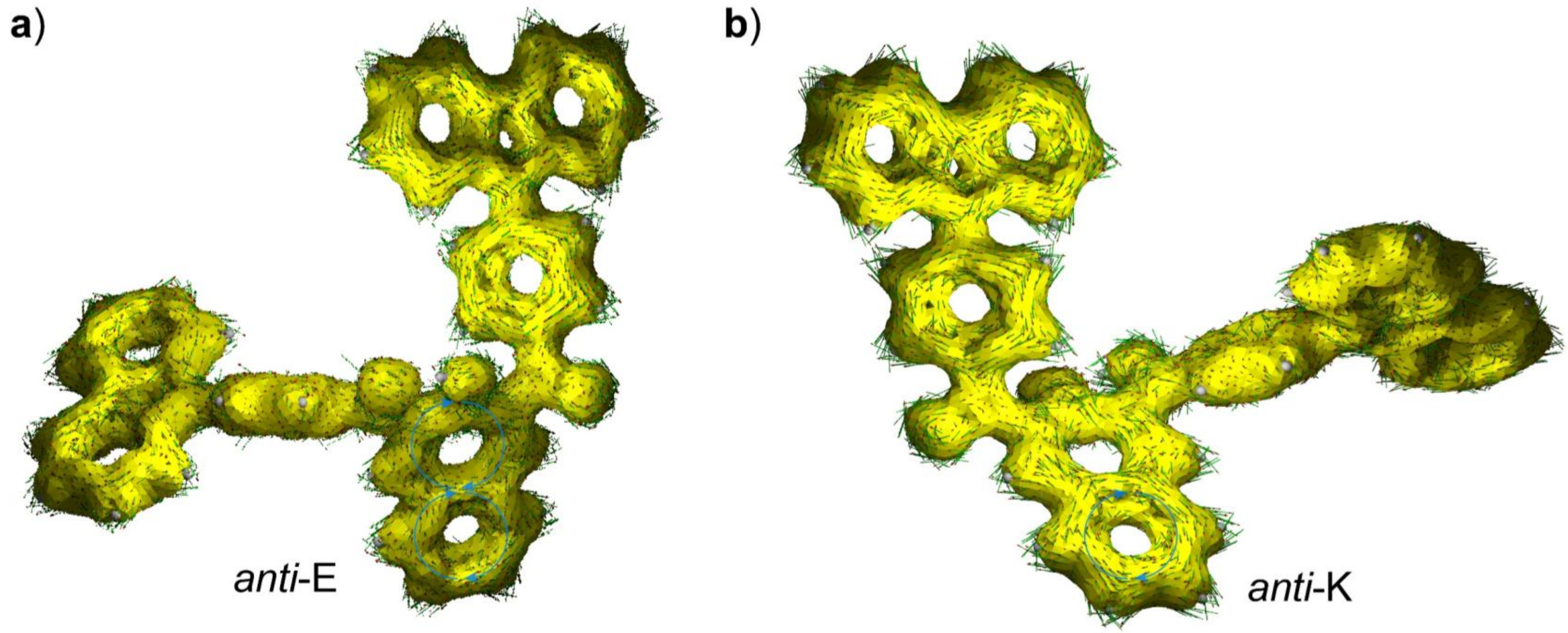

Fig. S53. AICD (isosurface: 0.05) plots of *anti*-**4d** before (E) and after (K) proton transfer in the S_0 state [ωB97XD/6-311+G(d,p)//ωB97XD/6-31G(d)]. Blue arrows represent the clockwise direction of ring currents indicative of aromaticity.

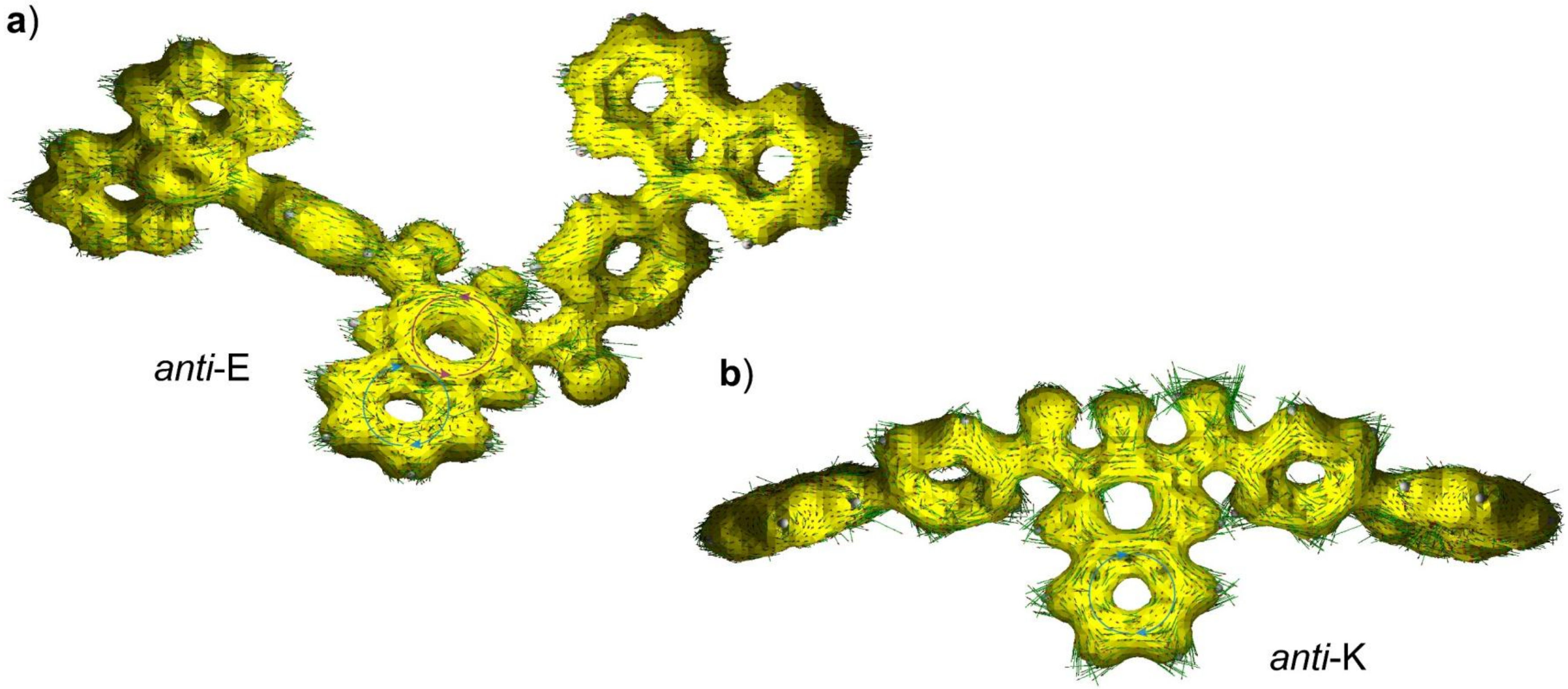

Fig. S54. AICD (isosurface: 0.05) plots of *anti*-**4d** **a**) before (E) and **b**) after proton transfer in the T_1 state [(U)ωB97XD/6-311+G(d,p)//(U)ωB97XD/6-31G(d)]. Blue and purple arrows represent the clockwise and anticlockwise direction of ring currents indicative of aromaticity and antiaromaticity, respectively.

EDDB^k

Table S24. EDDB calculations [(U)ωB97XD/6-311+G(d,p)//(U)ωB97XD/6-31G(d)] of **4b** before (E) and after (K) proton transfer in the S_0 and T_1 states. $EDDB_G$ – density of globally delocalized electrons in the bonds, $EDDB_H$ – density of globally delocalized electrons in bonds of heavy atoms, $EDDB_F$ – density of delocalized bonds in bonds of fragments (local contributions), $EDDB_E$ – density of delocalized electrons in bonds of fragments (local + non-local contributions). α and β represent the two unpaired electrons in the triplet state which contribute differently to the EDDB value.

**Species**	**$EDDB_G$**	**$EDDB_H$**	**$EDDB_F$**	**$EDDB_E$**
S_0<br>L-shaped<br>(E)	22.904	21.199	C_1: 3.595<br>C_{1-7}: 2.629<br>$C_{6–11}$: 3.804<br>PhL: 5.005<br>Php: 4.956	C_1: 5.768<br>C_{1-7}: 4.945<br>$C_{6–11}$: 5.249<br>PhL: 5.809<br>Php: 5.778
S_0<br>Propeller<br>(E)	22.774	21.092	C_1: 3.568<br>C_{1-7}: 2.609<br>$C_{6–11}$: 3.828<br>PhL: 2.294<br>Php: 2.580	C_1: 5.698<br>C_{1-7}: 4.887<br>$C_{6–11}$: 4.348<br>PhL: 4.750<br>Php: 5.736
S_0 (K)	22.419	20.653	C_1: 2.402<br>C_{1-7}: 1.734<br>$C_{6–11}$: 4.048<br>PhL: 5.065<br>Php: 4.718	C_1: 5.056<br>C_{1-7}: 4.412<br>$C_{6–11}$: 5.350<br>PhL: 5.868<br>Php: 5.568
T_1<br>Propeller<br>(E)	23.389<br>(α: 11.203;<br>β: 12.187)	21.597<br>(α: 10.309<br>β: 11.288)	C_1: 3.453<br>C_{1-7}: 2.537<br>$C_{6–11}$: 2.665<br>PhA: 5.045<br>PhB: 4.899	C_1: 6.416<br>C_{1-7}: 5.349<br>$C_{6–11}$: 4.343<br>PhA: 5.831<br>PhB: 5.829
T_1 (K)	23.260<br>(α: 11.121;<br>β: 12.139)	21.642<br>(α: 10.309;<br>β: 11.333)	C_1: 2.904<br>C_{1-7}: 2.199<br>$C_{6–11}$: 2.603<br>PhL: 4.999<br>Php: 5.109	C_1: 6.090<br>C_{1-7}: 5.284<br>$C_{6–11}$: 4.226<br>PhL: 5.806<br>Php: 5.926

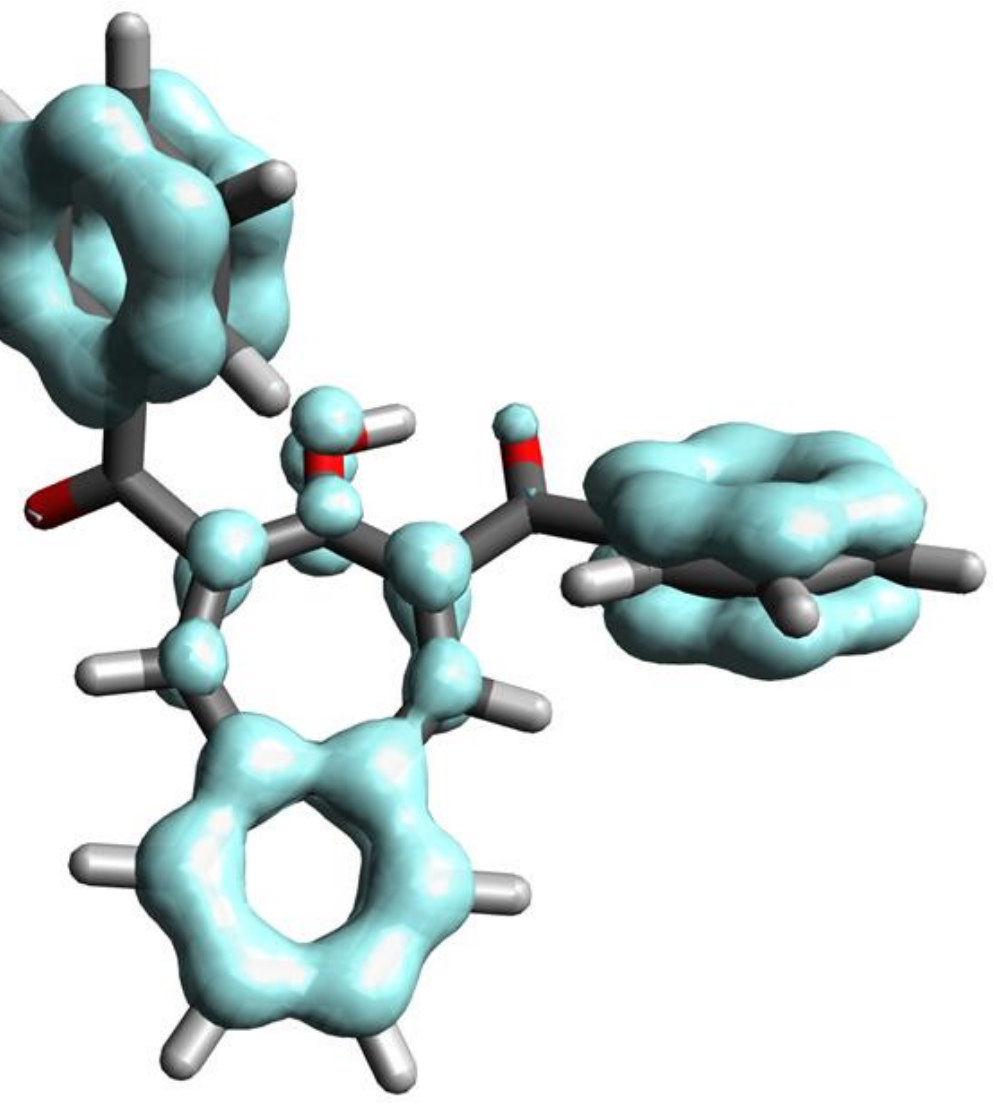

15.899e (56.8%)

Fig. S55. EDDB$_P$ plot of *anti*-E-**4b** in the S_0 state [ωB97XD/6-311+G(d,p)//ωB97XD/6-31G(d)]. The percentage value was calculated assuming a 28 π-electron system.

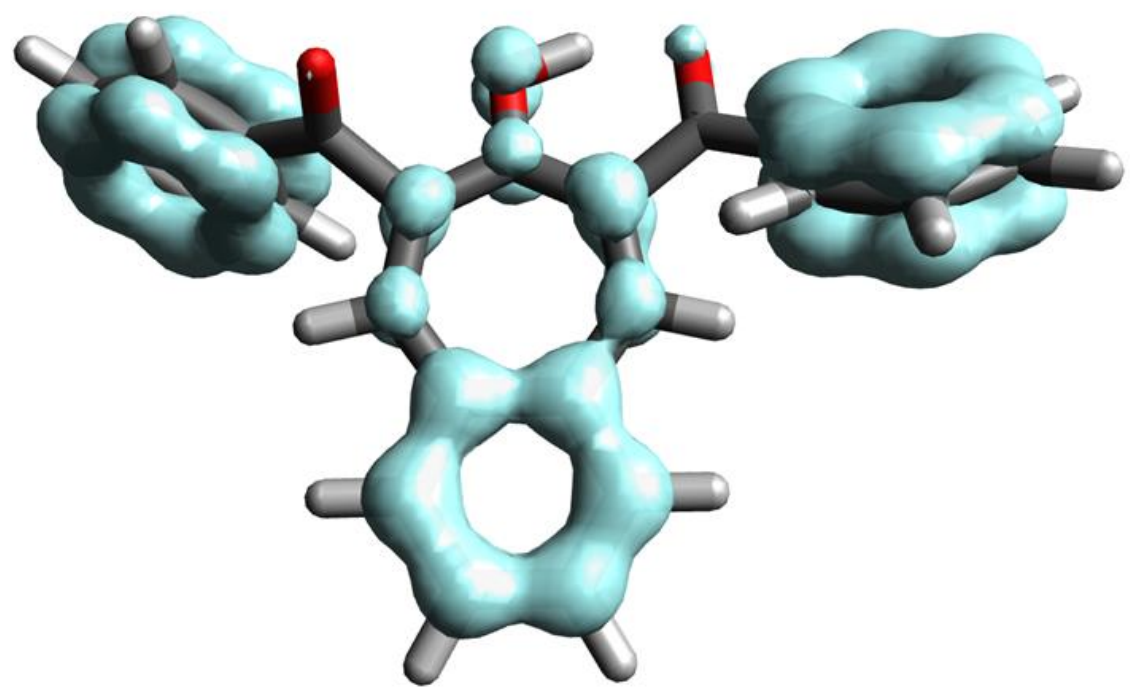

15.781e (56.4%)

Fig. S56. EDDB$_P$ plot of *syn*-E-**4b** in the S_0 state [ωB97XD/6-311+G(d,p)//ωB97XD/6-31G(d)]. The percentage value was calculated assuming a 28 π-electron system.

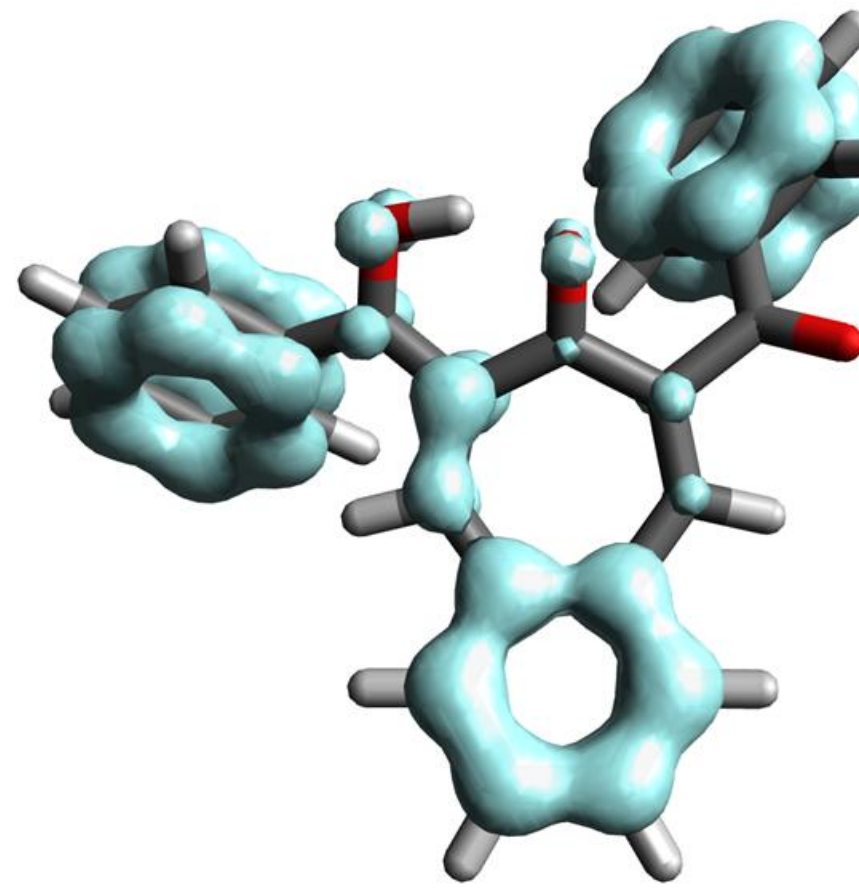

15.389e (54.9%)

Fig. S57. $EDDB_P$ plot of *anti*-K-**4b** in the S_0 state [ωB97XD/6-311+G(d,p)//ωB97XD/6-31G(d)]. The percentage value was calculated assuming a 28 π-electron system.

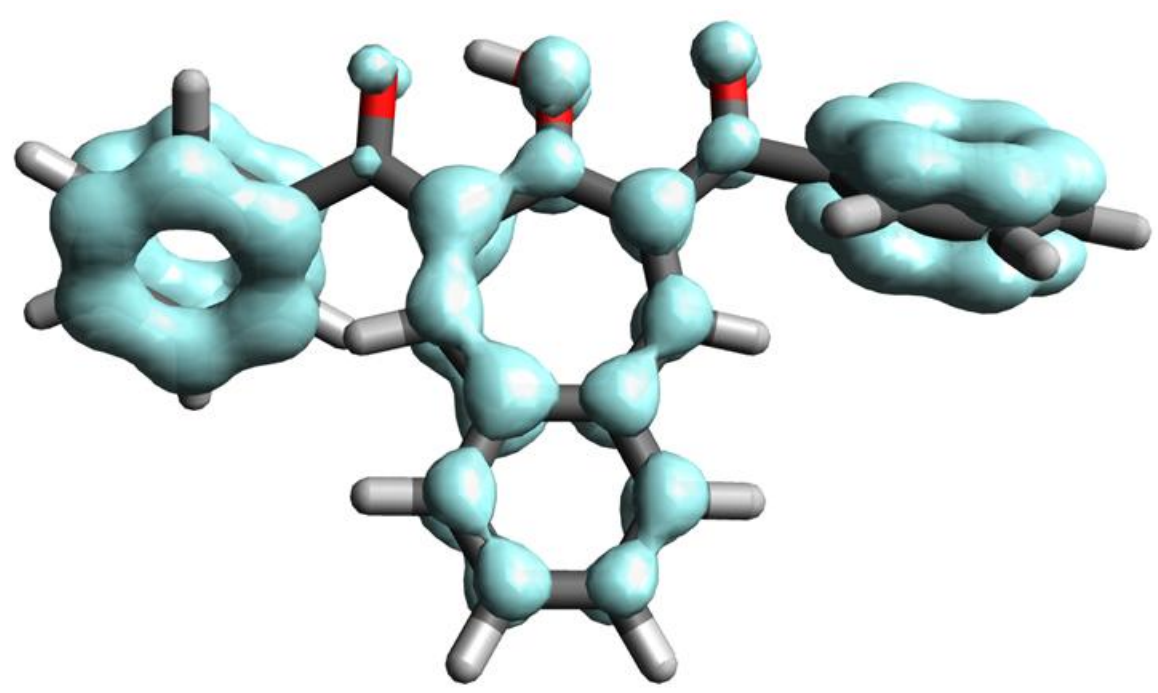

15.353e (54.8%)

Fig. S58. $EDDB_P$ plot of *syn*-E-**4b** in the T_1 state [(U)ωB97XD/6-311+G(d,p)//(U)ωB97XD/6-31G(d)]. The percentage value was calculated assuming a 28 π-electron system.

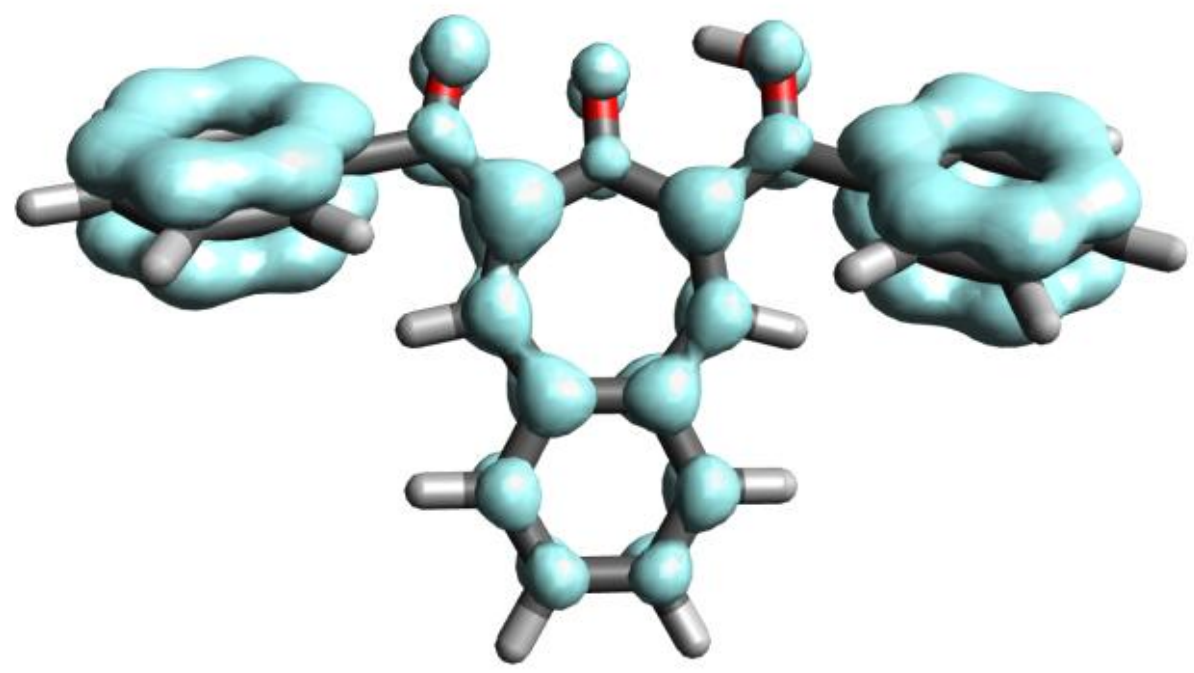

15.3814e (54.9%)

Fig. S59. $EDDB_P$ plot of *syn*-K-**4b** in the T_1 state [(U)ωB97XD/6-311+G(d,p)//(U)ωB97XD/6-31G(d)]. The percentage value was calculated assuming a 28 π-electron system.

Table S25. EDDB calculations [(U)ωB97XD/6-311+G(d,p)//(U)ωB97XD/6-31G(d)] of compound **4c** before (E) and after (K) proton transfer in the S_0 and T_1 states. $EDDB_G$ – density of globally delocalized electrons in the bonds, $EDDB_H$ – density of globally delocalized electrons in bonds of heavy atoms, $EDDB_F$ – density of delocalized bonds in bonds of fragments (local contributions), $EDDB_E$ – density of delocalized electrons in bonds of fragments (local + non-local contributions).

Species	$EDDB_G$	$EDDB_H$	$EDDB_F$	$EDDB_E$
S_0 L-shaped (E)	23.468	21.964	C_1: 3.681<br>$C_{1\text{-}7}$: 2.704<br>$C_{6\text{–}11}$: 1.862<br>PhL: 4.757<br>PhP: 4.729	C_1: 5.866<br>$C_{1\text{-}7}$: 5.038<br>$C_{6\text{–}11}$: 4.388<br>PhL: 5.782<br>PhP: 5.761
S_0 Propeller (E)	23.352	21.824	C_1: 3.651<br>$C_{1\text{-}7}$: 2.689<br>$C_{6\text{–}11}$: 3.775<br>PhA: 4.686<br>PhB: 4.737	C_1: 5.801<br>$C_{1\text{-}7}$: 4.993<br>$C_{6\text{–}11}$: 5.253<br>PhA: 5.703<br>PhB: 5.769
S_0 L-shaped (K)	23.162	21.526	C_1: 2.529<br>$C_{1\text{-}7}$: 1.844<br>$C_{6\text{–}11}$: 4.001<br>PhL: 4.801<br>PhP: 4.539	C_1: 5.239<br>$C_{1\text{-}7}$: 4.571<br>$C_{6\text{–}11}$: 5.378<br>PhL: 5.823<br>PhP: 5.568
T_1 (E)	24.397	22.757	C_1: 3.585<br>$C_{1\text{-}7}$: 2.652<br>$C_{6\text{–}11}$: 2.656<br>PhL: 4.698<br>PhP: 4.448	C_1: 6.716<br>$C_{1\text{-}7}$: 5.636<br>$C_{6\text{–}11}$: 4.487<br>PhL: 5.729<br>PhP: 5.563
T_1 (K)	23.793	22.358	C_1: 2.913<br>$C_{1\text{-}7}$: 2.211<br>$C_{6\text{–}11}$: 2.610<br>PhL: 4.759<br>PhP: 4.855	C_1: 6.092<br>$C_{1\text{-}7}$: 5.294<br>$C_{6\text{–}11}$: 4.253<br>PhL: 5.784<br>PhP: 5.889

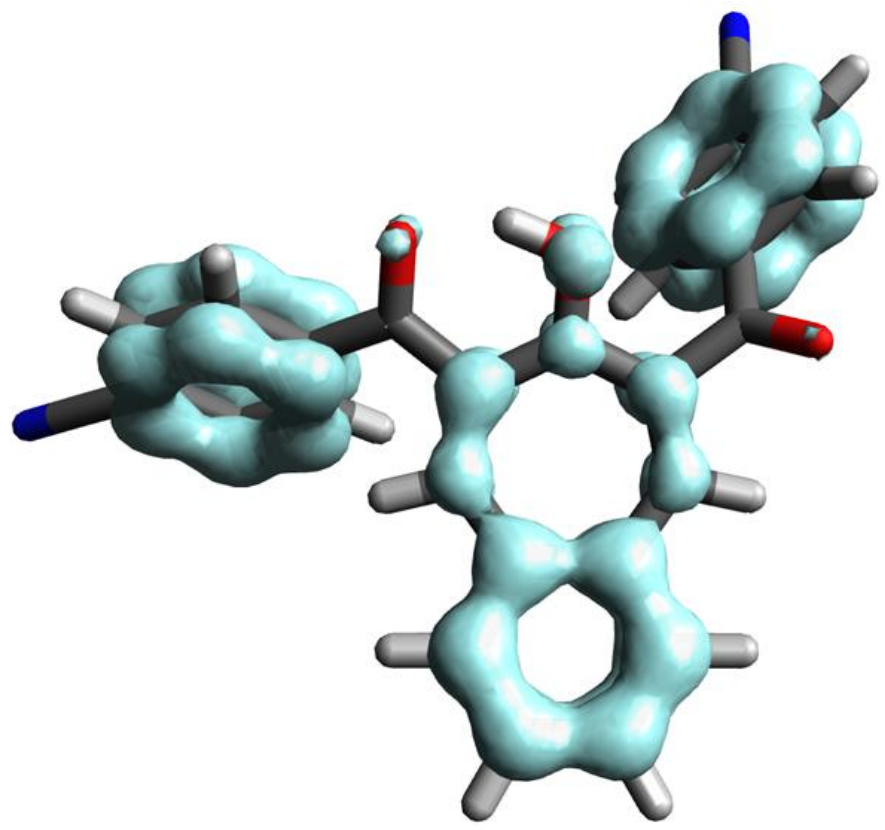

15.698e (56.1%)

Fig. S60. EDDB$_P$ plot of *anti*-E-**4c** in the S_0 state [ωB97XD/6-311+G(d,p)//ωB97XD/6-31G(d)]. The percentage value was calculated assuming a 28 π-electron system.

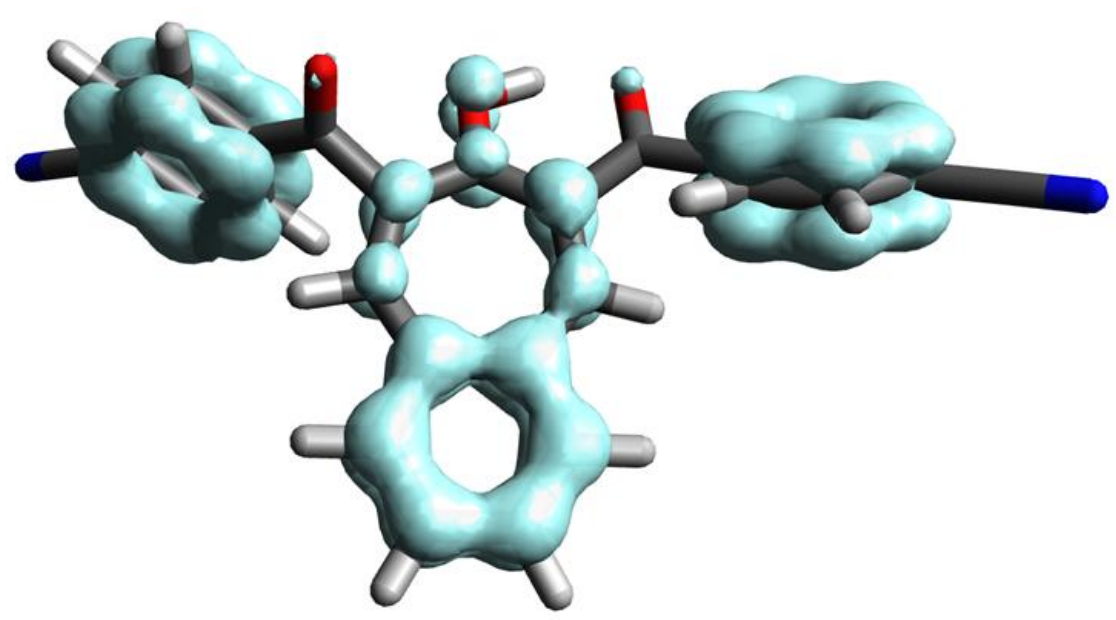

15.491e (55.3%)

Fig. S61. EDDB$_P$ plot of *syn*-E-**4c** in the S_0 state [ωB97XD/6-311+G(d,p)//ωB97XD/6-31G(d)]. The percentage value was calculated assuming a 28 π-electron system.

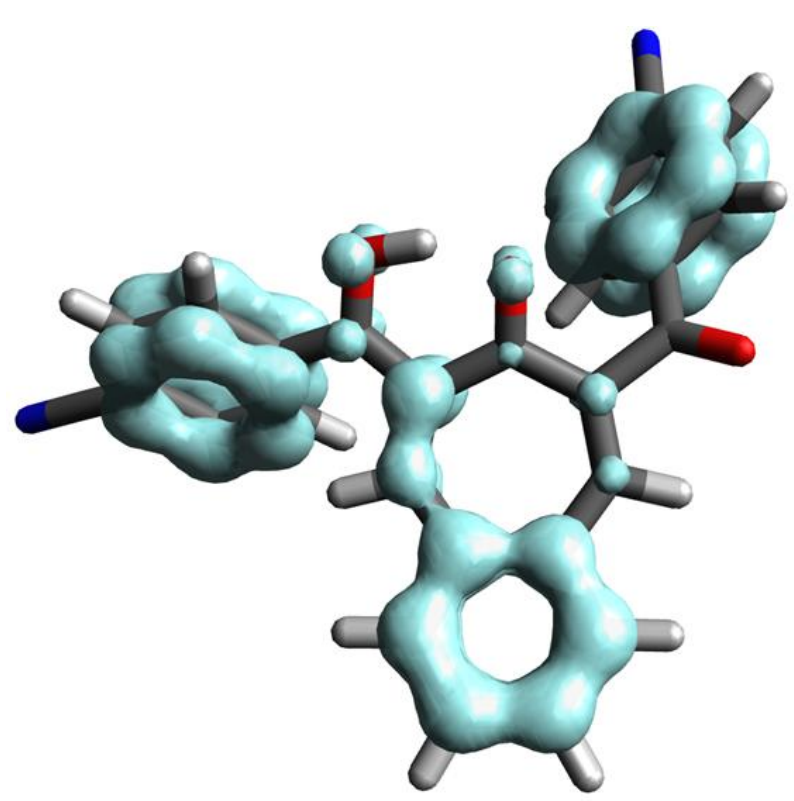

15.135e (54.1%)

Fig. S62. EDDB$_P$ plot of *anti*-K-**4c** in the S_0 state [ωB97XD/6-311+G(d,p)//ωB97XD/6-31G(d)]. The percentage value was calculated assuming a 28 π-electron system.

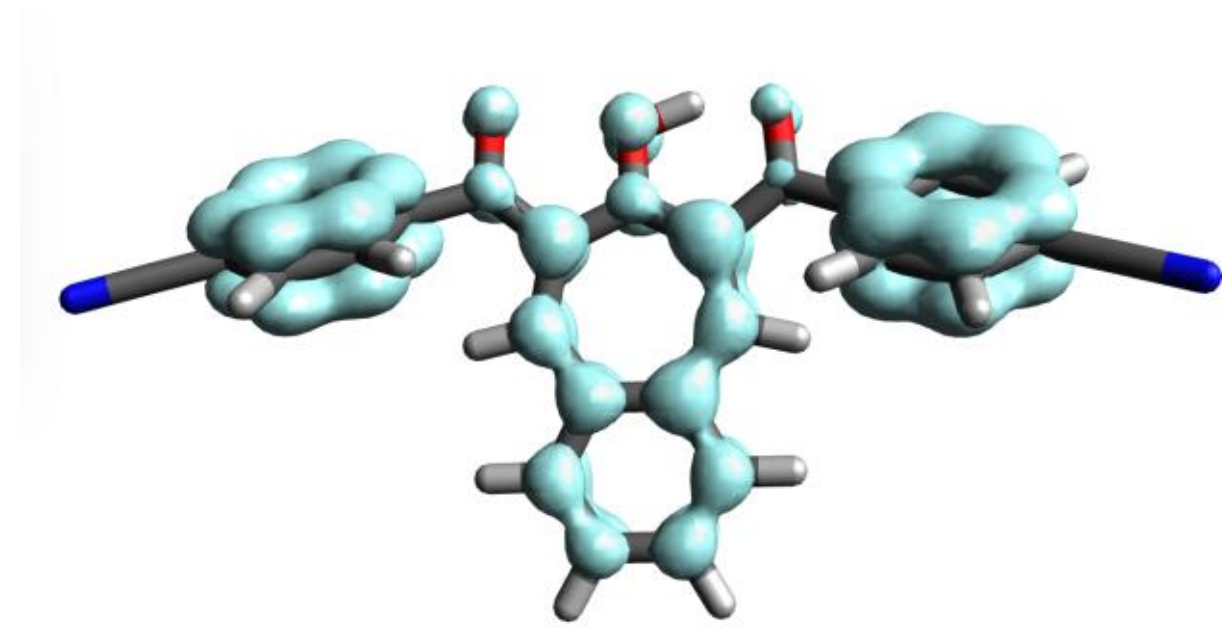

15.036e (53.7%)

Fig. S63. $EDDB_P$ plot of *syn*-E-**4c** in the T_1 state [(U)ωB97XD/6-311+G(d,p)//(U)ωB97XD/6-31G(d)]. The percentage value was calculated assuming a 28 π-electron system.

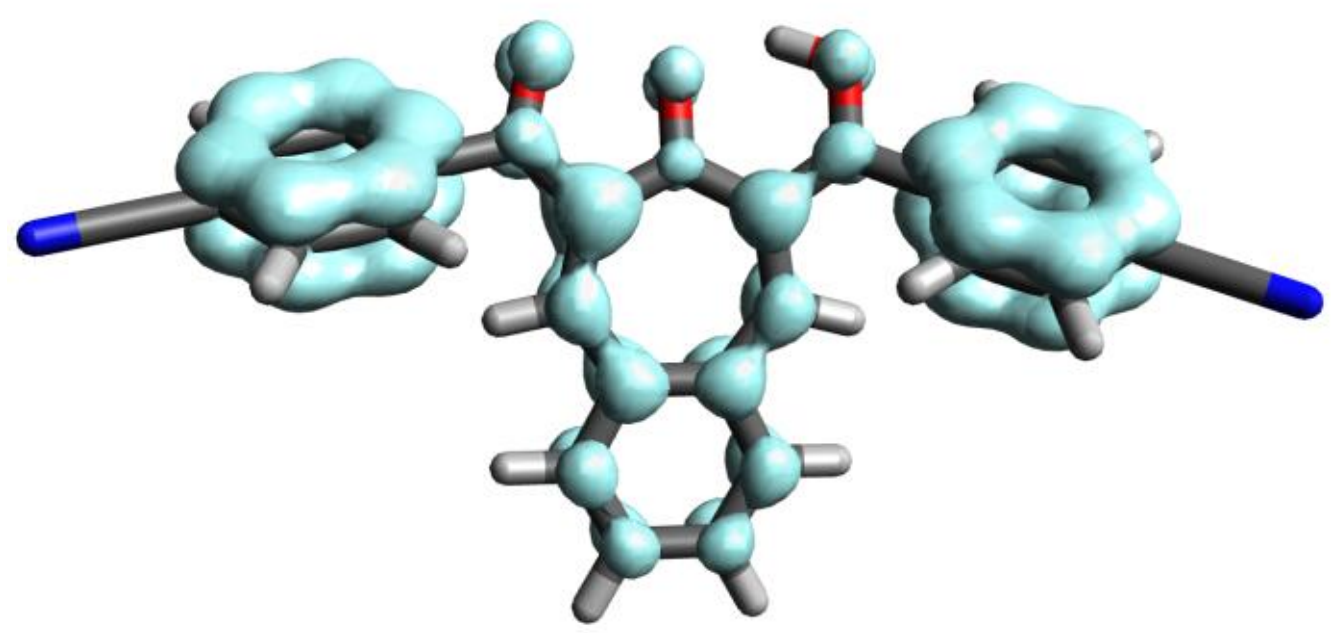

15.004 e (53.6%)

Fig. S64. $EDDB_P$ plot of *anti*-K-**4c** in the T_1 state [(U)ωB97XD/6-311+G(d,p)//(U)ωB97XD/6-31G(d)]. The percentage value was calculated assuming a 28 π-electron system.

Table S26. EDDB calculations [(U)ωB97XD/6-311+G(d,p)//(U)ωB97XD/6-31G(d)] of compound **4d** before (E) and after (K) proton transfer in the S_0 and T_1 states. $EDDB_G$ – density of globally delocalized electrons in the bonds, $EDDB_H$ – density of globally delocalized electrons in bonds of heavy atoms, $EDDB_F$ – density of delocalized bonds in bonds of fragments (local contributions), $EDDB_E$ – density of delocalized electrons in bonds of fragments (local + non-local contributions).

Species	$EDDB_G$	$EDDB_H$	$EDDB_F$	$EDDB_E$
S_0	48.764	45.682	C_1: 3.570	C_1: 5.724
L-shaped			C_{1-7}: 2.597	C_{1-7}:4.895
(E)			C_6: 3.829	C_6: 5.226
			PhL: 2.484	PhL: 5.190
			PhP: 4.481	PhP: 5.632
			Cbz(L): 8.019	Cbz(L): 9.836
			Cbz(P): 8.671	Cbz(P): 11.954
S_0	48.071	44.968	C_1: 2.263	C_1: 4.915
Propeller			C_{1-7}: 1.164	C_{1-7}: 4.281
(K)			C_6: 4.131	C_6: 5.329

T_1 (E)	48.641	45.457	PhL: 4.784<br>PhP: 3.768<br>Cbz(L): 11.718<br>Cbz(P): 11.744<br>C_1: 3.333<br>C_{1-7}: 2.431<br>C_6: 3.287	PhL: 4.912<br>PhP: 5.513<br>Cbz(L): 12.746<br>Cbz(P): 13.028<br>C_1: 6.098<br>C_{1-7}: 5.231<br>C_6: 4.913
T_1 (K)	49.282	46.238	PhL: 4.8975<br>Cbz(L): 11.685<br>PhP: 4.819<br>Cbz(P): 11.032<br>C_1: 2.903<br>C_{1-7}: 2.195<br>C_6: 2.599	PhL: 5.954<br>Cbz(L): 12.687<br>PhP: 5.871<br>Cbz(P): 12.055<br>C_1: 6.129<br>C_{1-7}: 5.309<br>C_6: 4.219
			PhA: 4.697<br>CbzA: 11.749<br>PhPB: 4.553<br>CbzB: 11.745	PhA: 5.682<br>PhPB: 5.848<br>CbzA: 12.904<br>CbzB: 12.905

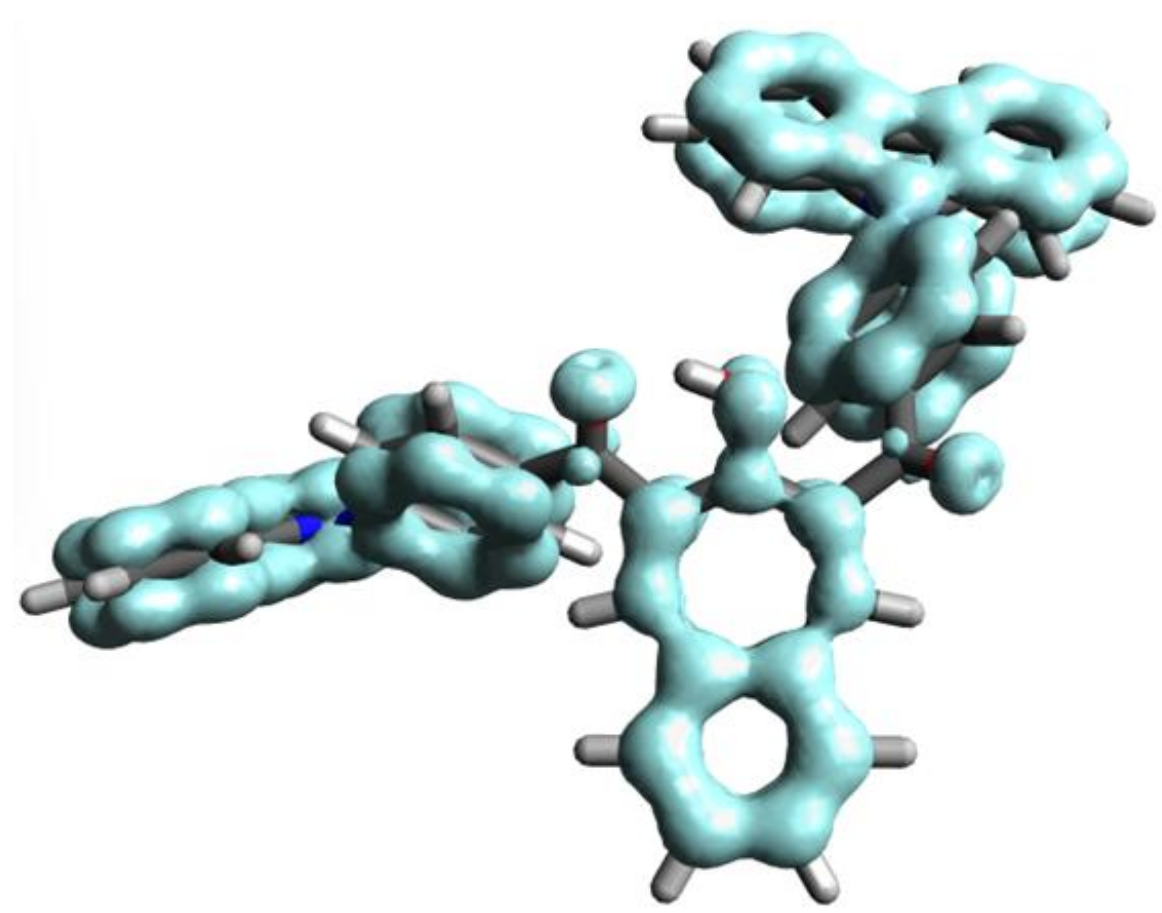

40.529e (77.9%)

Fig. S65. EDDB$_P$ plot of *anti*-E-**4d** in the S_0 state [ωB97XD/6-311+G(d,p)//ωB97XD/6-31G(d)]. The percentage value was calculated assuming a 52 π-electron system [ωB97XD/6-311+G(d,p)//ωB97XD/6-31G(d)].

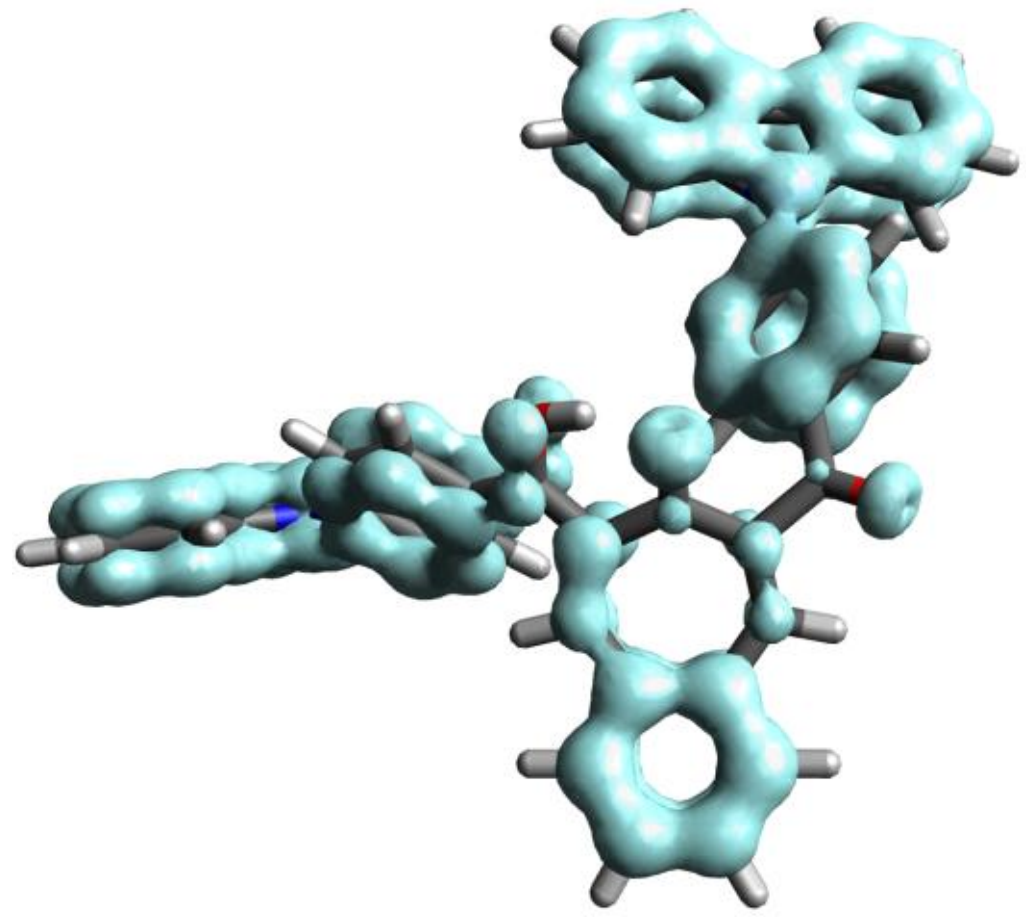

39.738e (76.4%)

Fig. S66. $EDDB_P$ plot of *anti*-K-**4d** in the S_0 state [ωB97XD/6-311+G(d,p)//ωB97XD/6-31G(d)]. The percentage value was calculated assuming a 52 π-electron system.

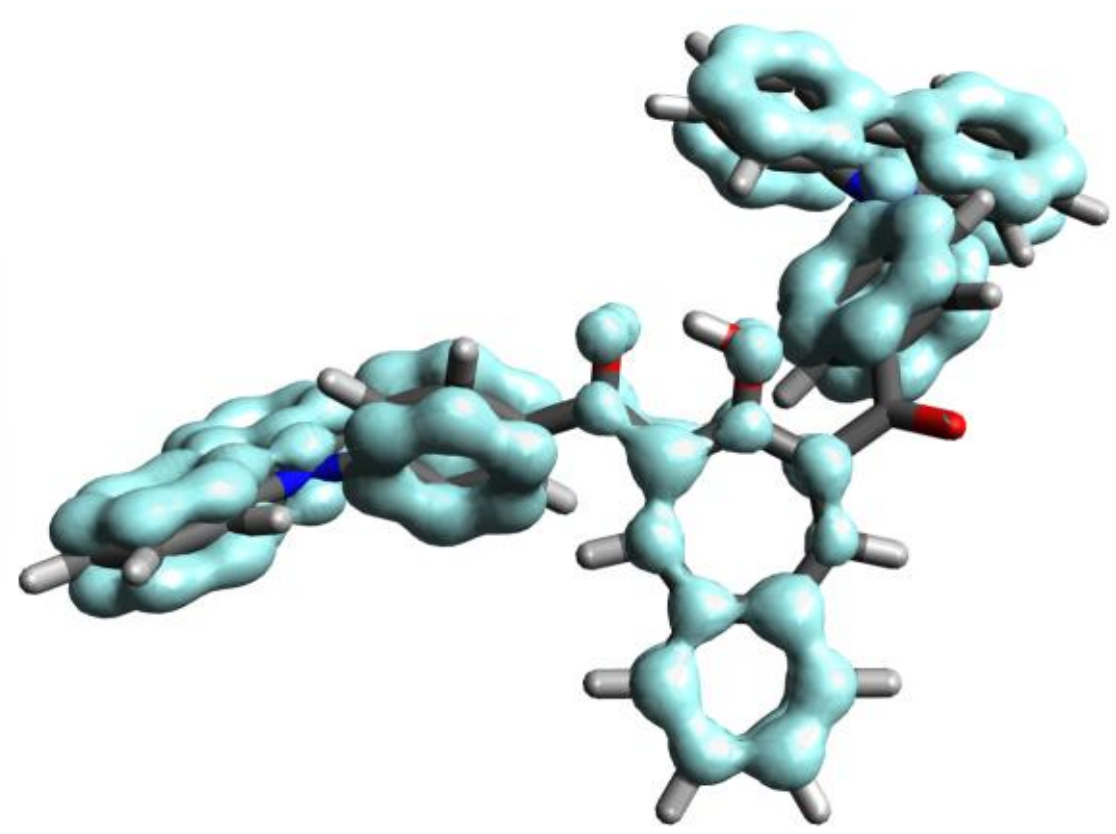

34.355 e (66.1%)

Fig. S67. $EDDB_P$ plot of *anti*-E-**4d** in the T_1 state [(U)ωB97XD/6-311+G(d,p)//(U)ωB97XD/6-31G(d)]. The percentage value was calculated assuming a 52 π-electron system.

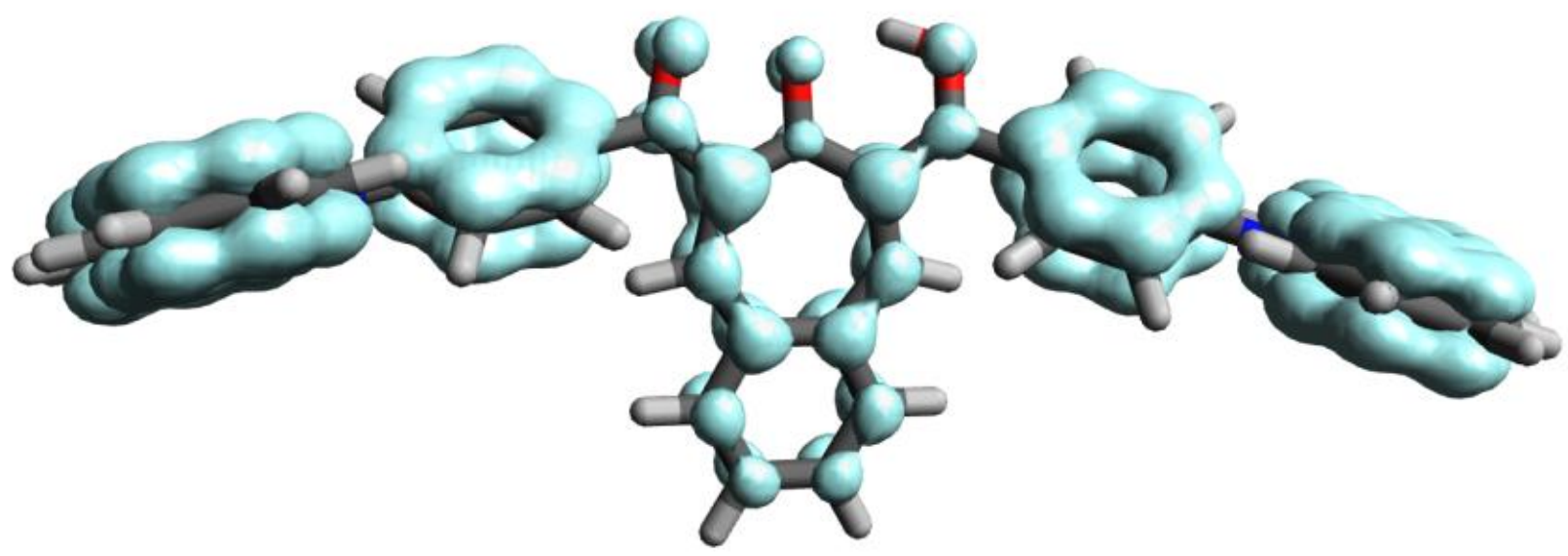

34.094 e (65.6 %)

Fig. S68. EDDB$_P$ plot of *syn*-K-**4d** in the T_1 state [(U)ωB97XD/6-311+G(d,p)//(U)ωB97XD/6-31G(d)]. The percentage value was calculated assuming a 52 π-electron system.

Tautomerization Energies of HTs and HBTs

Table S27. Calculated tautomerization energies (ΔG_T*) of ***o*-SC** and HT/HBT derivatives in the S_0, T_1, and S_1 states [S_0, T_1: (U)ωB97XD/6-31G(d)]; S_1: TD-ωB97XD/6-31G(d)].

Species	$\Delta G_T(S_0)$ (kJ mol^{-1})	$\Delta G_T(T_1)$ (kJ mol^{-1})	$\Delta G_T(S_1)$ (kJ mol^{-1})
phenol	57	−158	−123
**HT**	141	−75	26
**HBT**	122	7.9	7.9
o-**SC**	58.2	−46.7	−5.6
**1a**	60.8*	9.2	5.1
**2a**	98.8	10.7	−35.7[a]
**1b**	11.5	−32.5	55.2[b]
**2b**	80.6	−31.2	17.9[c]
**3b**	18.4	−13.7	−3.8
**3c**	15.2	−15.8	−4.8
**3d**	9.6	1.7	5.4
**3a**	44.2	−21.9	−21.5
**4a**	31.4	−22.3	−24.4
**4b**	11.7	−13.4	−6.7
**4c**	12.3	−16.5	−9.9
**4d**	5.9	−28	−23

*In the S_0 state, the K-isomer only converged with certain bond lengths constrained (the ones involved in proton transfer). The T_1 state competes with the **DT** isomer and is a second order saddle point.

[a] The S_1 state of the HBT has n–π* character, therefore, proton transfer is highly favorable, in contrast to the T_1 state, which is a purely π–π*.

[b] First singlet excited state of HBT is dominated by the charge-transfer character. The HOMO is located on the phenacyl part while the LUMO is located on the tropylium part. Domination of charge-transfer character in the S_1 excited state makes ESPT highly disfavored. The S_2 state also has charge transfer character. Additionally, excitation to the S_1 state is forbidden (0 oscillator strength).

[c] Same behavior as in (b) is observed for the **2b** where charge-transfer character still dominates, leading to unfavorable ESPT in the S_1 state.

The correlation between tautomerization energies (ΔG_T*) in the T_1 state and the S_0–T_1 Gibbs energy gaps (summarized in Fig. 2i of the main text of the manuscript) is shown below in Fig. S70. We find that in general, a smaller $\Delta G(S_0–T_1)$, i.e., a lower degree of ESAA typically leads to a smaller driving force for ESPT. Two notable exceptions to this trend are **1a** and **2a**. Despite the high $\Delta G(S_0–T_1)$ of the E tautomers of these compounds, aromaticity indices based on structural, magnetic, and

electronic criteria (Table 1) show that the T_1 states of E-**1a** and E-**2a** have non-aromatic character. ESPT to the K form does not have a strong driving force, as it does not relieve ESAA.

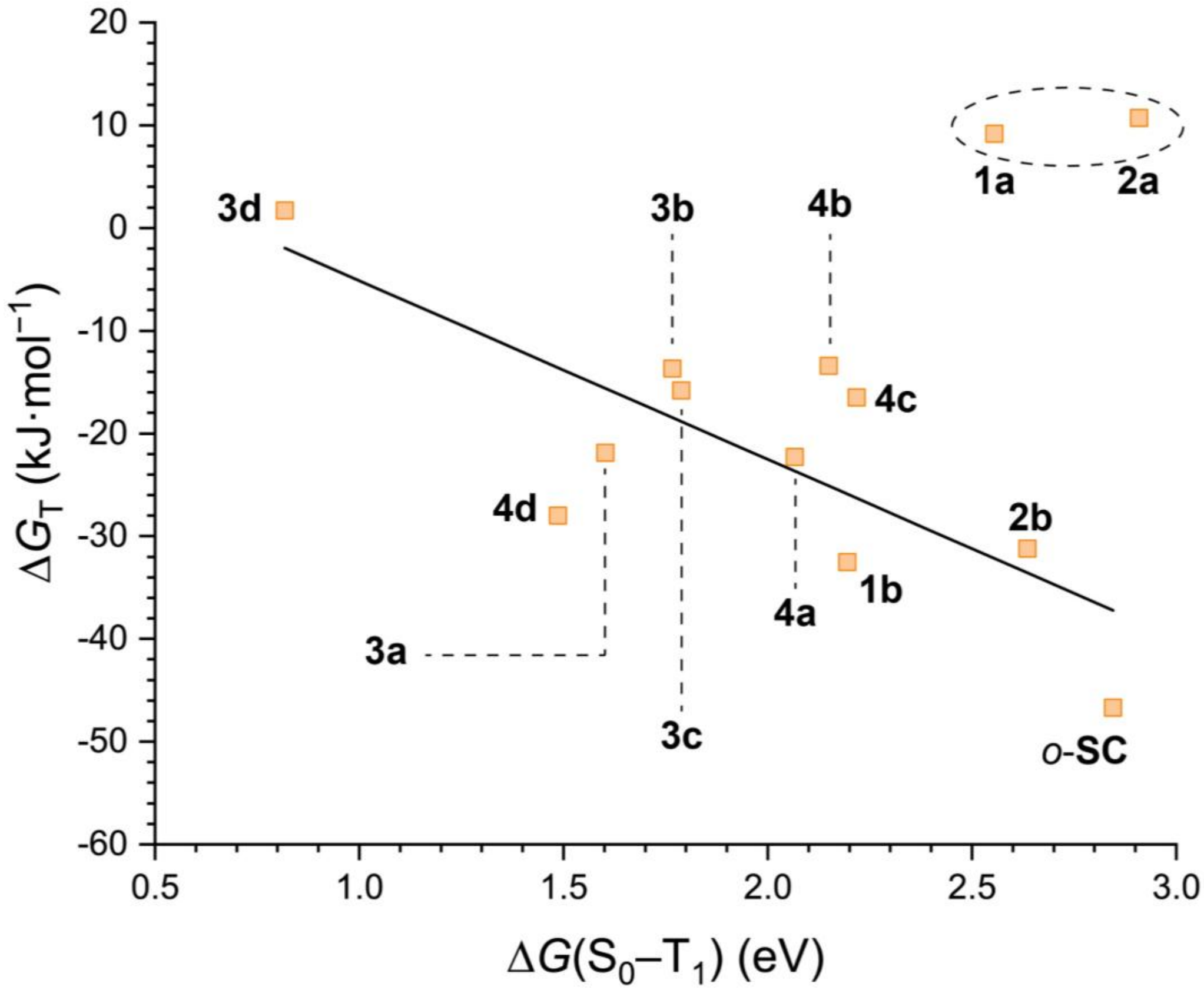

Fig. S69. Correlation between ΔG_T* in the T_1 state and $\Delta G(S_0–T_1)$ of the series of annulenes modelled. The line of best fit has a correlation coefficient r = 0.77. Compounds **1a** and **2a** were omitted from the line of best fit.

Molecular Orbitals of 7d and E-4d

HOMO **LUMO**

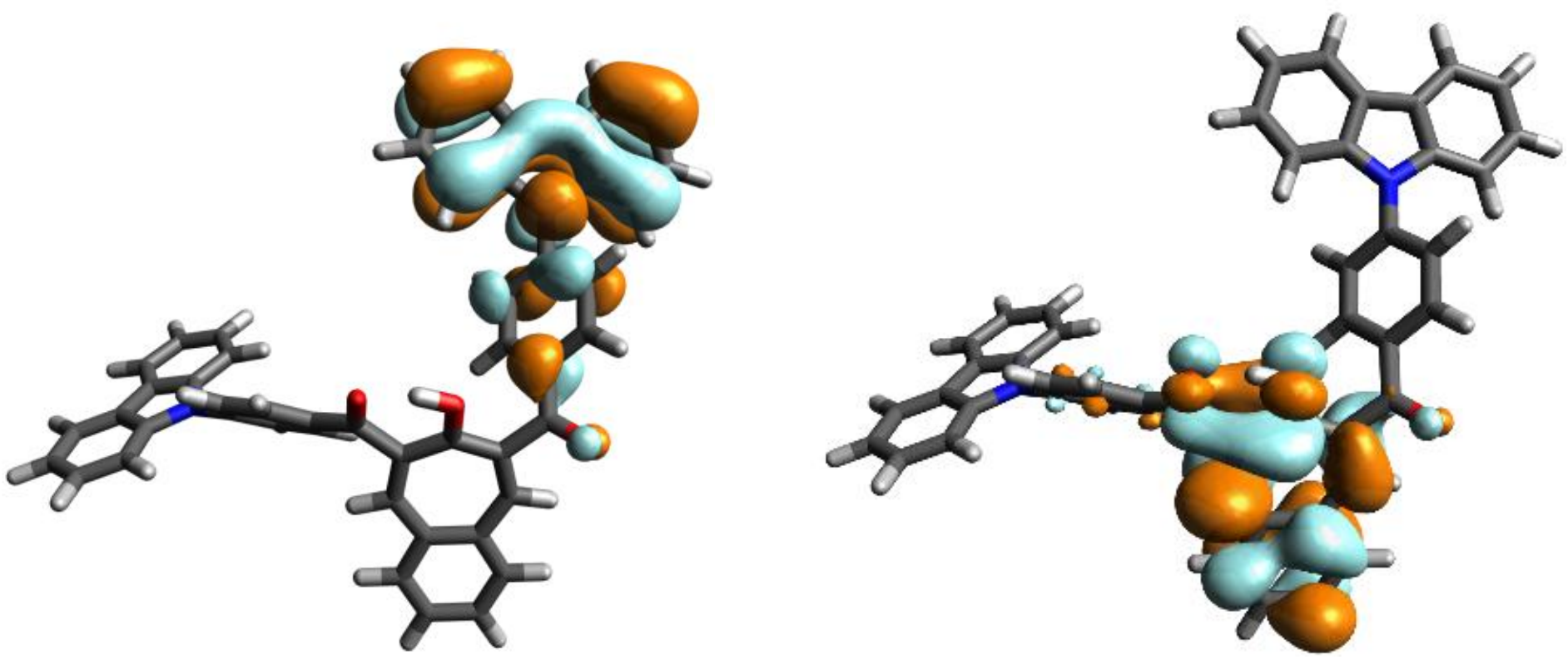

-9.088 eV -4.775 eV

Fig. S70. Highest occupied molecular orbital (HOMO) and lowest unoccupied molecular orbital (LUMO) diagrams of E-**4d** (ωB97XD/6-31G(d) level and isosurface value: 0.02).

HOMO **LUMO**

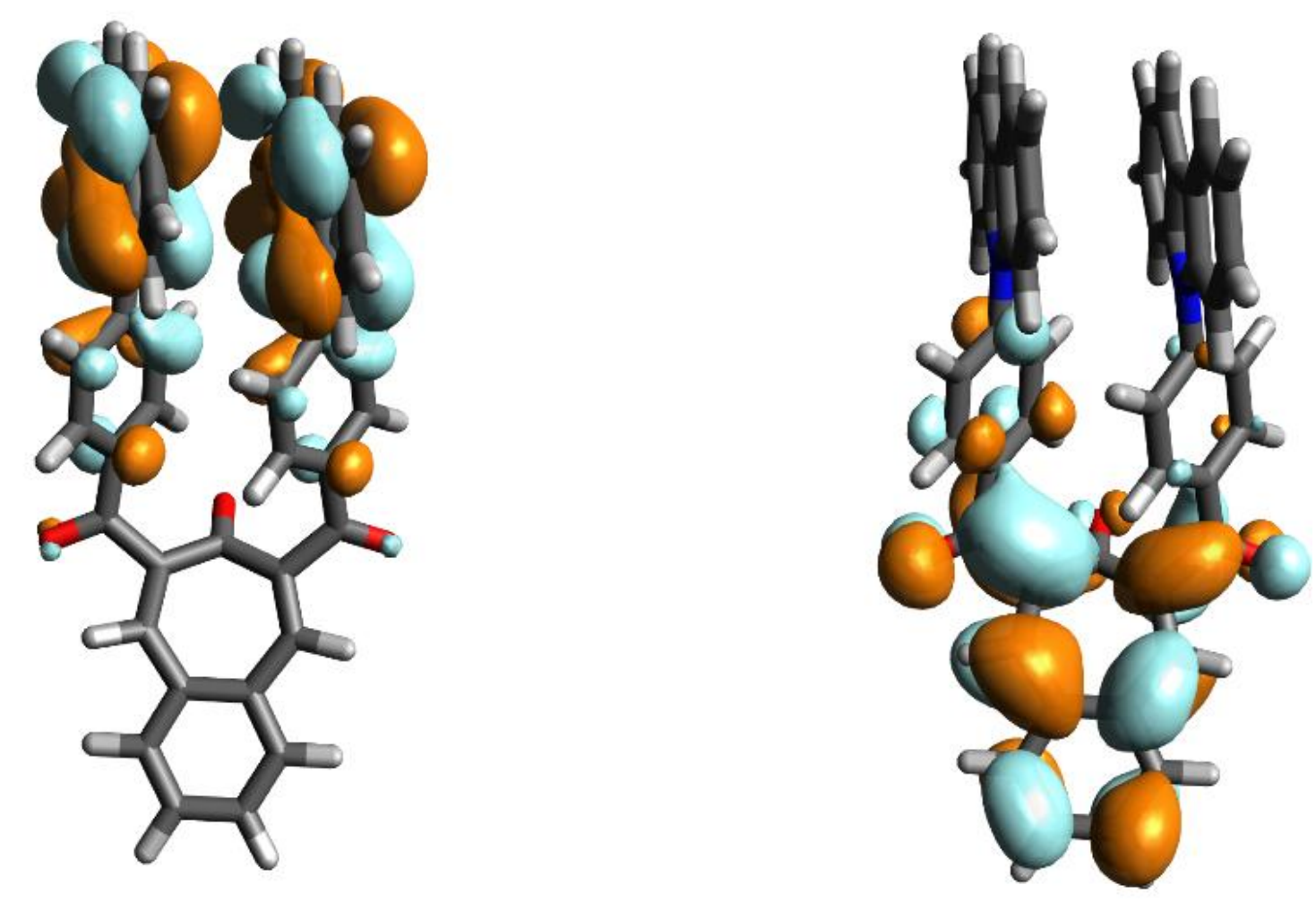

7.412 eV −1.198 eV

Fig. S71. Highest occupied molecular orbital (HOMO) and lowest unoccupied molecular orbital (LUMO) diagrams of **7d** in S_0 state in the U-shaped conformation (ωB97XD/6-31G(d) level and isosurface value: 0.02).

Functional Screen

Table S28. Comparison of ΔG_T values (kJ·mol^{-1}) of selected compounds using various functionals [6-31G(d)].

Species	ωB97XD	M06-2X	B3LYP-D3	CAM-B3LYP
syn-**4b** (S_0)	11.7	3.8	9.9	−0.3
syn-**4b** (T_1)	−13.4	−14.3[a]	−21.9[a]	−16.1[a]
syn-**4b** (S_1)	−6.7	−2.4	−3.1	−2.4

[a] Geometry optimization performed using frozen C–O and O–H bond coordinates for E-**4b**.

Qualitatively, ωB97XD, M06-2X, and B3LYP-D3 predict endergonic GSPT in the S_0 state for **4b**, as expected, while CAM-B3LYP predicts slightly endergonic GSPT. All four functionals screened predict that ESPT in the T_1 and S_1 states becomes exergonic. This screen suggests that the Δ$G_T^{(*)}$ values are not functional dependent. We chose the ωB97XD functional for our calculations, as it best describes both short- and long-range van der Waals interactions, as well as non-covalent hydrogen-bonding interactions, which are relevant to proton transfer.[18–20]

Cartesian Coordinates

Table S29. Coordinates for the optimized geometry of E-*o*-**SC** (S_0).

Atom	*x*	*y*	*z*
C	-0.733015	-1.461540	-0.000075
C	0.333151	-0.547367	-0.000084
C	0.062968	0.840562	-0.000039
C	-1.267560	1.278535	0.000025
C	-2.297037	0.353952	0.000033
C	-2.042690	-1.024795	-0.000022
H	-0.507531	-2.525767	-0.000109
H	-1.460966	2.345652	0.000057
H	-3.323857	0.708167	0.000079
H	-2.863004	-1.734075	-0.000016
O	1.035403	1.755067	-0.000081
H	1.894141	1.275591	-0.000184
C	1.706338	-1.029100	-0.000130
O	2.696249	-0.305388	0.000303
H	1.835071	-2.128473	0.000148

Table S30. Coordinates for the optimized geometry of E-*o*-**SC** (S_0).

Atom	*x*	*y*	*z*
C	0.789622	-1.478448	-0.000058
C	-0.325725	-0.572253	-0.000122
C	-0.109350	0.877659	-0.000369
C	1.268756	1.318000	0.000119
C	2.285337	0.415670	0.000243
C	2.059343	-1.003164	0.000077
H	0.593149	-2.548198	-0.000052
H	1.447136	2.387870	0.000240
H	3.311816	0.773447	0.000436
H	2.907600	-1.678765	0.000171
O	-1.075408	1.681375	-0.000238
H	-2.347162	0.661007	0.000738
C	-1.617640	-1.040202	-0.000101
O	-2.694346	-0.256000	0.000226
H	-1.856566	-2.101930	-0.000178

Table S31. Coordinates for the optimized geometry of E-*o*-**SC** (T_1).

Atom	*x*	*y*	*z*
C	-0.729186	-1.500050	-0.000007
C	0.372318	-0.618099	0.000130
C	0.039903	0.837337	0.000055
C	-1.290511	1.290775	0.000005
C	-2.333200	0.388741	-0.000065
C	-2.023364	-1.012282	-0.000113
H	-0.550907	-2.571119	0.000025
H	-1.455043	2.363345	-0.000018
H	-3.364854	0.716551	-0.000119
H	-2.845587	-1.723318	-0.000186
O	1.033233	1.695029	-0.000091
H	1.896728	1.130616	-0.000365
C	1.727308	-1.025169	0.000356
O	2.691258	-0.192791	-0.000055
H	1.944123	-2.101496	-0.000340

Table S32. Coordinates for the optimized geometry of K-*o*-**SC** (T_1).

Atom	*x*	*y*	*z*
C	-0.694796	-1.468739	0.000021
C	0.366462	-0.575911	0.000021
C	0.043657	0.866617	-0.000016
C	-1.329404	1.261484	-0.000047
C	-2.371974	0.322544	-0.000057
C	-2.050914	-1.018565	-0.000023
H	-0.498180	-2.537266	0.000044
H	-1.522278	2.329570	-0.000054
H	-3.405954	0.648307	-0.000082
H	-2.840630	-1.764422	-0.000024
O	0.960950	1.733974	0.000067
H	2.406914	0.731454	-0.000150
C	1.714370	-1.006932	0.000059
O	2.766958	-0.187711	-0.000020
H	1.972453	-2.060735	0.000134

Table S33. Coordinates for the optimized geometry of E-*o*-**SC** (S_1).

Atom	*x*	*y*	*z*
C	0.768151	-1.46828	0.024611
C	-0.33204	-0.57661	0.009295
C	-0.07153	0.82258	0.009379
C	1.241948	1.280958	-0.02131
C	2.304216	0.385604	-0.02555
C	2.063516	-0.99231	0.003889
H	0.576247	-2.5374	0.033476
H	1.405855	2.353348	-0.0248
H	3.32168	0.762065	-0.04542
H	2.892826	-1.69242	0.00188
O	-1.05667	1.750404	0.027505
H	-1.91905	1.3466	0.216628
C	-1.66646	-1.0621	0.001027
O	-2.72385	-0.30516	-0.06254
H	-1.88026	-2.13319	0.090539

Table S34. Coordinates for the optimized geometry of K-*o*-**SC** (S_1).

Atom	*x*	*y*	*z*
C	0.741178	-1.44748	-0.04389
C	-0.33429	-0.57925	0.031211
C	-0.07474	0.865337	0.008796
C	1.272942	1.287436	0.002312
C	2.344959	0.367884	0.003587
C	2.087604	-0.98623	-0.01916
H	0.554572	-2.51229	-0.16211
H	1.454053	2.354701	0.074942
H	3.36509	0.737037	-0.00036
H	2.896103	-1.70889	-0.04411
O	-1.05997	1.677097	0.020581
H	-2.32596	0.70795	-0.14066
C	-1.68137	-1.02429	0.103668
O	-2.70824	-0.23283	-0.10362
H	-1.93595	-2.03312	0.417506

Table S35. Coordinates for the optimized geometry of E-phenol (S_0).

Atom	*x*	*y*	*z*
C	-1.128280	-1.215267	0.000124
C	0.264000	-1.196441	0.000032
C	0.939321	0.023945	-0.000063
C	0.220956	1.220423	-0.000065
C	-1.167308	1.187221	0.000028
C	-1.851798	-0.027597	0.000123
H	-1.645910	-2.170094	0.000198
H	0.766087	2.158498	-0.000139
H	-1.720752	2.121804	0.000026
H	-2.936795	-0.046287	0.000195
O	2.297677	0.111282	-0.000156
H	2.671172	-0.777977	-0.000144
H	0.823441	-2.129905	0.000034

Table S36. Coordinates for the optimized geometry of K-phenol (S_0).

Atom	*x*	*y*	*z*
C	-1.205975	-1.149815	0.000129
C	0.284930	-1.277422	-0.000307
C	1.080234	0.025501	0.000006
C	0.313259	1.281247	-0.000192
C	-1.033311	1.276109	-0.000067
C	-1.813673	0.043296	0.000196
H	-1.787994	-2.067917	0.000309
H	0.888748	2.201352	-0.000290
H	-1.573883	2.219615	-0.000131
H	-2.896726	0.116597	0.000468
O	2.298341	0.007733	0.000319
H	0.615471	-1.862974	0.868720
H	0.614872	-1.862044	-0.870211

Table S37. Coordinates for the optimized geometry of E-phenol (T_1).

Atom	*x*	*y*	*z*
C	1.124124	1.269571	0.000126
C	-0.302094	1.244783	0.000032
C	-0.929554	-0.038227	-0.000062
C	-0.232562	-1.272549	-0.000068
C	1.193844	-1.226398	0.000027
C	1.814442	0.039620	0.000121
H	1.668701	2.204749	0.000201
H	-0.802768	-2.192336	-0.000144
H	1.785396	-2.131969	0.000028
H	2.902926	0.072278	0.000193
O	-2.253086	-0.121280	-0.000153
H	-2.633598	0.769762	-0.000141
H	-0.905163	2.146947	0.000030

Table S38. Coordinates for the optimized geometry of K-phenol (T_1).

Atom	*x*	*y*	*z*
C	1.113521	-1.214414	-0.000184
C	-0.376877	-1.283728	0.000292
C	-1.071534	0.074243	0.000103
C	-0.273002	1.257235	-0.000138
C	1.188666	1.222959	-0.000136
C	1.826796	0.022793	-0.000165
H	1.671869	-2.145449	-0.000865
H	-0.804201	2.203585	-0.000454
H	1.740499	2.156196	0.000182
H	2.912932	-0.011393	0.000003
O	-2.308724	0.127282	0.000191
H	-0.748605	-1.848015	-0.869729
H	-0.748132	-1.847709	0.870700

Table S39. Coordinates for the optimized geometry of E-phenol (S_1).

Atom	*x*	*y*	*z*
C	1.128008	1.261836	0.000126
C	-0.293084	1.237434	0.000032
C	-0.934387	-0.033226	-0.000063
C	-0.233553	-1.265111	-0.000068
C	1.188792	-1.222896	0.000027
C	1.839687	0.035258	0.000122
H	1.662619	2.203827	0.000201
H	-0.793839	-2.191383	-0.000143
H	1.767539	-2.138162	0.000027
H	2.925549	0.063636	0.000195
O	-2.275276	-0.117721	-0.000154
H	-2.652603	0.773062	-0.000142
H	-0.879846	2.151032	0.000032

Table S40. Coordinates for the optimized geometry of K-phenol (S_1).

Atom	*x*	*y*	*z*
C	-1.036834	1.219425	-0.153089
C	0.357773	1.229660	0.315764
C	1.046707	-0.172676	-0.030693
C	0.223439	-1.298718	0.045879
C	-1.171876	-1.181694	0.102610
C	-1.788840	0.072284	-0.102854
H	-1.445293	2.137697	-0.564706
H	0.713379	-2.265411	0.092881
H	-1.773632	-2.081947	0.156233
H	-2.853202	0.112703	-0.317327
O	2.269835	-0.053050	-0.217060
H	0.955366	2.039788	-0.108648
H	0.462489	1.271874	1.412348

Table S41. Coordinates for the optimized geometry of E-**HT** (S_0).

Atom	*x*	*y*	*z*
C	-1.892449	-0.672301	-0.000054
C	-1.879163	0.705096	0.000111
C	-0.759523	1.560144	0.000197
C	-0.789637	-1.551653	-0.000176
C	0.584588	1.256790	0.000142
C	0.556938	-1.272097	-0.000161
C	1.200192	-0.012866	-0.000018
H	-2.869883	-1.146422	-0.000097
H	-2.846175	1.199716	0.000185
H	-0.989174	2.622448	0.000327
H	-1.039641	-2.609191	-0.000300
H	1.242937	-2.114810	-0.000272
O	2.518365	-0.110956	-0.000047
H	2.968148	0.747677	0.000050
H	1.261183	2.109555	0.000234

Table S42. Coordinates for the optimized geometry of K-**HT** (S_0).

Atom	*x*	*y*	*z*
C	1.739101	-0.703653	-0.154724
C	1.752414	0.692812	-0.293229
C	0.735246	1.563485	0.016862
C	0.697027	-1.616847	0.174530
C	-0.624625	1.260190	0.502361
C	-0.629963	-1.336763	0.269665
C	-1.273127	-0.015170	0.003185
H	2.704102	-1.178595	-0.325600
H	2.694578	1.129616	-0.608686
H	0.968678	2.623569	-0.065530
H	1.001332	-2.653265	0.284844
H	-1.335529	-2.151825	0.415142
O	-2.319296	-0.013033	-0.590500
H	-1.304557	2.094957	0.305819
H	-0.550673	1.175483	1.606115

Table S43. Coordinates for the optimized geometry of E-**HT** (T_1).

Atom	*x*	*y*	*z*
C	-1.876084	-0.716752	-0.000062
C	-1.840268	0.777715	0.000117
C	-0.753077	1.615689	0.000203
C	-0.827965	-1.592448	-0.000181
C	0.613904	1.290935	0.000147
C	0.555941	-1.318295	-0.000165
C	1.186164	-0.026253	-0.000018
H	-2.875273	-1.139966	-0.000101
H	-2.818107	1.248926	0.000187
H	-0.970227	2.679942	0.000334
H	-1.082321	-2.648327	-0.000305
H	1.253058	-2.149029	-0.000274
O	2.484433	-0.127889	-0.000046
H	2.945450	0.730992	0.000051
H	1.320276	2.117026	0.000237

Table S44. Coordinates for the optimized geometry of E-**HT** (T_1).

Atom	*x*	*y*	*z*
C	-1.876084	-0.716752	-0.000062
C	-1.840268	0.777715	0.000117
C	-0.753077	1.615689	0.000203
C	-0.827965	-1.592448	-0.000181
C	0.613904	1.290935	0.000147
C	0.555941	-1.318295	-0.000165
C	1.186164	-0.026253	-0.000018
H	-2.875273	-1.139966	-0.000101
H	-2.818107	1.248926	0.000187
H	-0.970227	2.679942	0.000334
H	-1.082321	-2.648327	-0.000305
H	1.253058	-2.149029	-0.000274
O	2.484433	-0.127889	-0.000046
H	2.945450	0.730992	0.000051
H	1.320276	2.117026	0.000237

Table S45. Coordinates for the optimized geometry of K-**HT** (T_1).

Atom	*x*	*y*	*z*
C	-1.830604	0.604101	0.066535
C	-1.729579	-0.780058	-0.273277
C	-0.629818	-1.563371	-0.157964
C	-0.801071	1.547684	0.143486
C	0.633758	-1.169605	0.542491
C	0.566086	1.375902	-0.053912
C	1.241054	0.133028	-0.026759
H	-2.838059	1.002627	0.146600
H	-2.635326	-1.242013	-0.656805
H	-0.676411	-2.590295	-0.507764
H	-1.119071	2.582300	0.241927
H	1.189557	2.249292	-0.215977
O	2.443310	0.001189	-0.318831
H	1.383322	-1.960320	0.496131
H	0.450546	-0.937194	1.602937

Table S46. Coordinates for the optimized geometry of E-**HT** (S_1).

Atom	*x*	*y*	*z*
C	1.847383	-0.693218	0.168166
C	1.825312	0.735156	-0.138186
C	0.764897	1.604280	-0.059919
C	0.810614	-1.587733	0.041913
C	-0.596354	1.276155	0.156918
C	-0.547549	-1.291489	-0.180318
C	-1.159171	-0.009742	-0.004259
H	2.819991	-1.090142	0.449368
H	2.789199	1.155753	-0.417798
H	0.981954	2.659542	-0.202629
H	1.054273	-2.642008	0.146406
H	-1.240220	-2.105348	-0.367066
O	-2.481541	-0.125203	0.001814
H	-2.940598	0.725226	0.127773
H	-1.283055	2.098143	0.343542

Table S47. Coordinates for the optimized geometry of K-**HT** (S_1).

Atom	*x*	*y*	*z*
C	-1.825086	0.609263	0.068634
C	-1.727701	-0.772530	-0.305220
C	-0.634880	-1.556110	-0.156423
C	-0.801143	1.541146	0.172757
C	0.610180	-1.156509	0.574043
C	0.557859	1.358395	-0.103660
C	1.264472	0.113616	-0.044128
H	-2.833215	1.001363	0.176802
H	-2.626020	-1.220476	-0.720672
H	-0.677692	-2.587055	-0.495644
H	-1.105853	2.572694	0.326674
H	1.191022	2.232715	-0.223067
O	2.454892	0.001366	-0.311157
H	1.355835	-1.950298	0.570934
H	0.394588	-0.883489	1.618211

Table S48. Coordinates for the optimized geometry of E-**HBT** (S_0).

Atom	*x*	*y*	*z*
C	0.699729	0.716344	-0.000097
C	0.695920	-0.714157	0.000102
C	-0.465583	-1.542189	0.000213
C	-0.459076	1.551855	-0.000217
C	-1.804151	-1.257316	0.000169
C	-1.796724	1.273057	-0.000183
C	-2.429007	0.009353	-0.000010
H	-0.242845	-2.606920	0.000362
H	-0.231407	2.615439	-0.000363
H	-2.484211	2.113433	-0.000302
O	-3.746344	0.090234	-0.000026
C	1.937049	-1.393726	0.000201
C	1.944898	1.386170	-0.000187
C	3.131610	-0.710069	0.000109
C	3.135262	0.694028	-0.000086
H	1.939433	-2.479303	0.000352
H	4.069110	-1.255510	0.000188
H	4.076032	1.233937	-0.000159
H	1.954578	2.471610	-0.000338
H	-4.177362	-0.778269	0.000094
H	-2.472138	-2.116389	0.000286

Table S49. Coordinates for the optimized geometry of K-**HBT** (S_0).

Atom	*x*	*y*	*z*
C	-0.632332	0.740943	0.120167
C	-0.637182	-0.706317	0.024525
C	0.458142	-1.531041	0.199263
C	0.535059	1.614269	0.203350
C	1.848272	-1.205414	0.539922
C	1.846255	1.347644	0.078813
C	2.485427	0.025681	-0.110308
H	0.255940	-2.598298	0.118268
H	0.273594	2.665173	0.296163
H	2.543208	2.179533	0.028390
O	3.533623	-0.079592	-0.692200
C	-1.885134	-1.389522	-0.178317
C	-1.868757	1.382759	0.076399
C	-3.076357	-0.713575	-0.238423
C	-3.060369	0.680744	-0.095497
H	-1.871444	-2.471632	-0.267907
H	-4.010548	-1.242942	-0.384851
H	-3.994781	1.232912	-0.127843
H	-1.906898	2.463811	0.159470
H	2.502416	-2.062936	0.367096

H	1.861392	-1.005903	1.629455

Table S50. Coordinates for the optimized geometry of E-**HBT** (T_1).

Atom	*x*	*y*	*z*
C	-0.689091	-0.703374	-0.000095
C	-0.681775	0.720421	0.000102
C	0.461010	1.559295	0.000216
C	0.468613	-1.556638	-0.000219
C	1.827072	1.244041	0.000167
C	1.804347	-1.255247	-0.000182
C	2.461321	0.008679	-0.000007
H	0.248457	2.623710	0.000364
H	0.244871	-2.619654	-0.000367
H	2.489550	-2.099330	-0.000303
O	3.801535	-0.107177	-0.000028
C	-1.955848	1.371777	0.000197
C	-1.941292	-1.363204	-0.000182
C	-3.205980	0.672970	0.000104
C	-3.206985	-0.682684	-0.000082
H	-1.972499	2.457442	0.000347
H	-4.128084	1.243335	0.000186
H	-4.123126	-1.260923	-0.000159
H	-1.945281	-2.449646	-0.000332
H	4.240652	0.753917	0.000092
H	2.484822	2.112348	0.000286

Table S51. Coordinates for the optimized geometry of K-**HBT** (T_1).

Atom	*x*	*y*	*z*
C	-0.655348	0.711809	0.178678
C	-0.569920	-0.733344	-0.005388
C	0.597180	-1.492908	0.170774
C	0.412613	1.599337	0.072729
C	1.836270	-1.005849	0.825584
C	1.766944	1.335432	-0.216572
C	2.481311	0.059939	-0.064442
H	0.574903	-2.525170	-0.167405
H	0.156893	2.656141	0.122830
H	2.384478	2.161251	-0.559482
O	3.542496	-0.107728	-0.628435
C	-1.759310	-1.406991	-0.332159
C	-1.969189	1.315417	0.246822
C	-3.004288	-0.763584	-0.314122
C	-3.116511	0.595882	0.014075
H	-1.719587	-2.469309	-0.553199
H	-3.896279	-1.339298	-0.540823
H	-4.088225	1.073524	0.055665
H	-2.031337	2.381378	0.446780

H	2.555574	-1.812355	0.966271
H	1.605098	-0.555188	1.800970

Table S52. Coordinates for the optimized geometry of E-**HBT** (S_1).

Atom	*x*	*y*	*z*
C	-0.690638	-0.702191	-0.000096
C	-0.685063	0.705851	0.000100
C	0.471744	1.570656	0.000217
C	0.461306	-1.573841	-0.000221
C	1.817551	1.253305	0.000169
C	1.801389	-1.264548	-0.000182
C	2.449580	-0.002674	-0.000008
H	0.249873	2.632510	0.000365
H	0.233769	-2.634835	-0.000367
H	2.489131	-2.106115	-0.000302
O	3.791801	-0.102835	-0.000026
C	-1.938351	1.366190	0.000196
C	-1.949848	-1.359166	-0.000182
C	-3.188503	0.689509	0.000106
C	-3.194755	-0.678576	-0.000084
H	-1.948429	2.452282	0.000347
H	-4.107767	1.264174	0.000189
H	-4.118360	-1.245887	-0.000159
H	-1.961626	-2.445214	-0.000334
H	4.218362	0.764133	0.000093
H	2.484178	2.114549	0.000286

Table S53. Coordinates for the optimized geometry of K-**HBT** (S_1).

Atom	*x*	*y*	*z*
C	-0.673090	0.725672	0.143063
C	-0.562302	-0.709863	-0.014684
C	0.674765	-1.401920	0.141826
C	0.427120	1.616197	0.048394
C	1.765894	-0.908036	0.958167
C	1.770273	1.352621	-0.191583
C	2.455852	0.072239	-0.113640
H	0.802666	-2.352762	-0.368746
H	0.166083	2.672100	0.045425
H	2.435293	2.201953	-0.331321
O	3.466108	-0.263718	-0.671424
C	-1.720438	-1.435546	-0.289541
C	-1.967581	1.311304	0.180220
C	-2.975990	-0.821213	-0.266229
C	-3.104965	0.550229	0.000783
H	-1.649977	-2.503382	-0.470864
H	-3.862401	-1.418521	-0.453549
H	-4.084892	1.012655	0.026555

H	-2.051239	2.386971	0.304461
H	2.518768	-1.657144	1.198011
H	1.459609	-0.342236	1.840763

Table S54. Coordinates for the optimized geometry of E-**1a** (S_0).

Atom	*x*	*y*	*z*
C	2.581718	-0.329671	-0.000108
C	1.800427	-1.458664	-0.000170
C	0.392066	-1.534614	-0.000096
C	2.157550	1.022167	0.000017
C	-0.577142	-0.550538	-0.000015
C	0.889392	1.538070	0.000057
C	-0.367456	0.867968	-0.000015
H	3.657178	-0.483769	-0.000149
H	2.319038	-2.412695	-0.000245
H	0.002854	-2.552139	-0.000071
H	2.956525	1.758670	0.000066
H	0.787862	2.620153	0.000098
C	-1.990018	-1.048047	0.000174
H	-2.122024	-2.141835	-0.000282
O	-2.954513	-0.310336	0.000507
O	-1.380397	1.684256	-0.000253
H	-2.241379	1.180226	-0.000512

Table S55. Coordinates for the optimized geometry of K-**1a** (S_0).

Atom	*x*	*y*	*z*
C	2.574073	-0.329043	-0.000137
C	1.806758	-1.466612	-0.000150
C	0.402276	-1.550473	-0.000002
C	2.169727	1.043809	0.000122
C	-0.567697	-0.550311	0.000044
C	0.919612	1.570479	0.000214
C	-0.381202	0.911366	-0.000012
H	3.649967	-0.485924	-0.000287
H	2.334137	-2.414977	-0.000273
H	0.018356	-2.569774	0.000045
H	2.986673	1.759790	0.000247
C	-1.903938	-1.035070	0.000103
H	-2.126334	-2.100149	0.000212
O	-2.973861	-0.286510	0.000060
O	-1.411556	1.659560	-0.000238
H	-2.715134	0.609398	-0.000012
H	0.818017	2.652363	0.000401

Table S56. Coordinates for the optimized geometry of E-**1a** (T_1).

Atom	*x*	*y*	*z*
C	2.589668	-0.269073	-0.048213
C	1.812315	-1.436036	0.067326
C	0.445227	-1.563453	0.093903
C	2.138776	1.039858	-0.108317
C	-0.587225	-0.574136	0.026495
C	0.833609	1.534230	-0.039798
C	-0.381548	0.860652	0.056513
H	3.665079	-0.412181	-0.090244
H	2.364987	-2.369463	0.119864
H	0.081014	-2.586205	0.151513
H	2.906629	1.803625	-0.193512
H	0.720857	2.614377	-0.047272
C	-1.897442	-1.059958	-0.062909
H	-2.114219	-2.137679	-0.047703
O	-2.967107	-0.360670	-0.162568
O	-1.418153	1.686493	0.136586
H	-2.262545	1.288433	0.405204

Table S57. Coordinates for the optimized geometry of K-**1a** (T_1).

Atom	*x*	*y*	*z*
C	2.600364	-0.330992	-0.000034
C	1.774650	-1.472570	-0.000066
C	0.404085	-1.549426	-0.000042
C	2.209804	0.995794	0.000015
C	-0.606067	-0.534108	0.000026
C	0.922769	1.550889	0.000065
C	-0.305945	0.894617	0.000090
H	3.670317	-0.515366	-0.000056
H	2.291402	-2.427616	-0.000118
H	0.000939	-2.559499	-0.000087
H	3.009533	1.731341	0.000021
H	0.856783	2.634268	0.000068
C	-1.929843	-0.975041	-0.000003
H	-2.133531	-2.041771	-0.000073
O	-3.038996	-0.285108	0.000026
O	-1.358645	1.662498	-0.000047
H	-2.933213	0.684546	0.000102

Table S58. Coordinates for the optimized geometry of E-**1a** (S_1).

Atom	*x*	*y*	*z*
C	2.58657	-0.2607	-0.05441
C	1.81402	-1.4327	0.07214
C	0.4486	-1.5646	0.10235
C	2.1354	1.0421	-0.11677
C	-0.57513	-0.57325	0.02782
C	0.82484	1.53551	-0.03632
C	-0.38488	0.85997	0.06336
H	3.66184	-0.40275	-0.10331
H	2.37119	-2.36336	0.12592
H	0.0874	-2.58793	0.16414
H	2.89992	1.80803	-0.2101
H	0.71101	2.61548	-0.0344
C	-1.90159	-1.04845	-0.06586
H	-2.0991	-2.13879	-0.03757
O	-2.94965	-0.37274	-0.17914
O	-1.43394	1.67678	0.13579
H	-2.25053	1.28972	0.48828

Table S59. Coordinates for the optimized geometry of E-**1a** (S_1).

Atom	*x*	*y*	*z*
C	-2.60209	-0.32713	-0.01923
C	-1.77995	-1.46720	0.02104
C	-0.41158	-1.54225	0.03258
C	-2.20720	0.99601	-0.03829
C	0.60313	-0.53915	0.01000
C	-0.92617	1.55398	-0.01151
C	0.31960	0.90315	0.02253
H	-3.67251	-0.50811	-0.03504
H	-2.29583	-2.42256	0.03966
H	-0.00760	-2.55260	0.05703
H	-3.00348	1.73565	-0.06681
H	-0.85547	2.63589	-0.01748
C	1.93409	-0.96884	-0.02143
H	2.13980	-2.03579	-0.03744
O	3.03555	-0.27516	-0.04574
O	1.36392	1.62546	0.06254
H	2.92043	0.69375	-0.04836

Table S60. Coordinates for the optimized geometry of E-**1b** (S_0).

Atom	*x*	*y*	*z*
C	-3.250513	-1.876952	-0.329950
C	-1.961203	-1.975437	-0.786915
C	-0.915202	-1.033580	-0.662807
C	-3.851558	-0.770667	0.316451
C	-0.897712	0.258623	-0.175826
C	-3.337370	0.476209	0.546677
C	-2.054442	1.009003	0.230276
H	-3.888166	-2.744523	-0.474350
H	-1.684519	-2.909184	-1.267551
H	0.044348	-1.390843	-1.026652
H	-4.879644	-0.916611	0.636424
H	-3.994053	1.213750	1.000683
C	0.430516	0.999503	-0.179709
O	0.410513	2.221919	-0.315068
O	-1.981851	2.295294	0.400163
H	-1.074619	2.615538	0.110998
C	1.702314	0.274095	-0.025258
C	1.816595	-0.888921	0.752834
C	2.845218	0.838186	-0.610774
C	3.058290	-1.484576	0.926763
H	0.951024	-1.304606	1.260709
C	4.077951	0.223443	-0.451634
H	2.750643	1.750063	-1.190756
C	4.184437	-0.935898	0.315904
H	3.150585	-2.371208	1.544763
H	4.958923	0.650848	-0.918102
H	5.152253	-1.409121	0.446889

Table S61. Coordinates for the optimized geometry of K-**1b** (S_0).

Atom	*x*	*y*	*z*
C	-3.287320	-1.844126	-0.311835
C	-1.999560	-1.987893	-0.741273
C	-0.924297	-1.072646	-0.600170
C	-3.886318	-0.715468	0.328087
C	-0.897654	0.224356	-0.136054
C	-3.366348	0.520090	0.539683
C	-2.066694	1.058771	0.180311
H	-3.946021	-2.694768	-0.464853
H	-1.740945	-2.931926	-1.210719
H	0.029809	-1.462663	-0.943221
H	-4.914339	-0.858178	0.649603
H	-4.006033	1.275202	0.988825
C	0.407190	0.918366	-0.092780
O	0.417929	2.190535	-0.131200

O	-1.942503	2.302600	0.202109
H	-0.597753	2.516943	-0.072919
C	1.692539	0.249634	0.003821
C	1.851144	-0.956735	0.712248
C	2.814212	0.883807	-0.561980
C	3.110044	-1.524689	0.829875
H	1.007185	-1.415409	1.217444
C	4.062706	0.293724	-0.458963
H	2.689815	1.821124	-1.093315
C	4.210282	-0.907813	0.235114
H	3.238235	-2.441398	1.394853
H	4.924516	0.770471	-0.912703
H	5.192572	-1.360740	0.323240

Table S62. Coordinates for the optimized geometry of E-**1b** (T_1).

Atom	*x*	*y*	*z*
C	-3.324283	-1.910191	-0.235658
C	-1.933073	-2.126032	-0.328261
C	-0.912483	-1.215407	-0.246733
C	-3.969934	-0.702268	-0.046336
C	-0.918844	0.201936	-0.039513
C	-3.435829	0.581031	0.105027
C	-2.108316	1.013679	0.116053
H	-3.954510	-2.789138	-0.329362
H	-1.626282	-3.154679	-0.494401
H	0.083029	-1.635393	-0.370530
H	-5.054992	-0.745118	-0.006816
H	-4.144461	1.391261	0.247197
C	0.329241	0.845564	-0.012164
O	0.582862	2.087683	0.087456
O	-2.001423	2.319707	0.302035
H	-1.076878	2.631829	0.342138
C	1.692221	0.244241	0.013008
C	2.583212	0.552509	-1.046475
C	2.128302	-0.498865	1.134313
C	3.854353	0.009190	-1.040478
H	2.248700	1.192779	-1.855130
C	3.398645	-1.030810	1.124814
H	1.452587	-0.670715	1.965673
C	4.262763	-0.775600	0.041002
H	4.536507	0.208750	-1.859055
H	3.739949	-1.640483	1.954296
H	5.268982	-1.182067	0.058477

Table S63. Coordinates for the optimized geometry of K-**1b** (T_1).

Atom	*x*	*y*	*z*
C	3.197004	-1.927027	0.069552
C	1.901785	-1.946461	0.740449
C	0.898221	-1.010737	0.748378
C	3.855585	-0.832811	-0.395605
C	0.873736	0.287163	0.171616
C	3.423884	0.519440	-0.353093
C	2.119694	1.067961	-0.102302
H	3.694570	-2.888837	-0.005111
H	1.683855	-2.867002	1.275874
H	-0.007037	-1.309794	1.268039
H	4.851103	-0.988889	-0.801470
H	4.153281	1.292334	-0.575505
C	-0.401927	0.956845	0.032411
O	-0.475442	2.247840	-0.052513
O	2.028120	2.315125	-0.110649
H	0.447902	2.631941	-0.003412
C	-1.676600	0.265739	-0.029253
C	-2.827035	0.943916	0.420331
C	-1.800612	-1.019430	-0.593540
C	-4.064228	0.327732	0.346284
H	-2.733431	1.937970	0.843120
C	-3.047286	-1.616650	-0.683401
H	-0.934276	-1.524834	-1.006819
C	-4.175128	-0.950051	-0.204362
H	-4.946104	0.841526	0.712698
H	-3.145189	-2.596646	-1.137308
H	-5.148649	-1.425270	-0.269596

Table S64. Coordinates for the optimized geometry of E-**1b** (S_1).

Atom	*x*	*y*	*z*
C	-3.258163	-1.980391	-0.000044
C	-1.860861	-2.163993	-0.001167
C	-0.865915	-1.218943	-0.001288
C	-3.936586	-0.779497	0.000751
C	-0.915551	0.207140	-0.000411
C	-3.439380	0.530553	0.000538
C	-2.129422	0.998737	-0.000047
H	-3.861721	-2.882147	0.000137
H	-1.521865	-3.195714	-0.001965
H	0.139062	-1.631219	-0.002099
H	-5.020904	-0.847408	0.001517
H	-4.173507	1.329997	0.000942
C	0.316860	0.893510	-0.000062
O	0.547982	2.129259	0.000646

O	-2.048481	2.321191	-0.000188
H	-1.123403	2.639701	-0.000098
C	1.684119	0.295995	-0.000005
C	2.355508	0.081985	-1.236029
C	2.354580	0.080430	1.236302
C	3.601630	-0.498726	-1.228271
H	1.861169	0.345439	-2.163862
C	3.600702	-0.500288	1.228823
H	1.859460	0.342765	2.164041
C	4.225514	-0.788811	0.000345
H	4.113323	-0.715762	-2.158663
H	4.111678	-0.718431	2.159346
H	5.222483	-1.217032	0.000426

Table S65. Coordinates for the optimized geometry of K-**1b** (S_1).

Atom	*x*	*y*	*z*
C	3.125906	-1.945403	0.066938
C	1.869975	-1.958279	0.725624
C	0.908491	-0.973308	0.801610
C	3.792868	-0.843807	-0.435425
C	0.881534	0.323681	0.204436
C	3.401488	0.499153	-0.378728
C	2.130071	1.047753	-0.082317
H	3.645708	-2.897374	0.013306
H	1.610716	-2.895767	1.210407
H	0.009194	-1.245636	1.346619
H	4.789557	-1.020021	-0.830373
H	4.135516	1.249994	-0.656936
C	-0.378313	0.984718	0.036840
O	-0.506948	2.280208	-0.106507
O	2.028554	2.309732	-0.051081
H	0.370203	2.720122	-0.043847
C	-1.649234	0.272216	-0.030467
C	-2.811693	0.942668	0.399965
C	-1.758691	-1.021198	-0.577632
C	-4.043021	0.313000	0.324625
H	-2.732770	1.943320	0.809556
C	-2.997544	-1.633553	-0.666871
H	-0.883838	-1.525587	-0.974728
C	-4.138266	-0.972743	-0.207443
H	-4.932140	0.824223	0.677249
H	-3.080584	-2.622229	-1.105054
H	-5.105850	-1.459944	-0.272418

Table S66. Coordinates for the optimized geometry of E-**2a** (S_0).

Atom	*x*	*y*	*z*
C	-1.421792	0.760046	0.000138
C	-1.061984	-0.622982	0.000018
C	0.267212	-1.135075	-0.000098
C	-0.499381	1.858596	0.000158
C	1.512814	-0.553950	-0.000108
C	0.860714	1.914131	0.000107
C	1.806935	0.853582	0.000011
H	0.298807	-2.224842	-0.000242
H	-0.982000	2.833267	0.000250
H	1.323511	2.896419	0.000194
C	2.658429	-1.504085	-0.000385
H	2.403186	-2.576499	-0.000007
O	3.823719	-1.153379	-0.000670
O	3.040886	1.256893	0.000241
C	-2.092117	-1.592085	-0.000014
C	-2.789032	1.097568	0.000239
C	-3.422227	-1.229157	0.000086
C	-3.772484	0.127056	0.000218
H	-1.824901	-2.644394	-0.000118
H	-4.193452	-1.991584	0.000063
H	-4.817561	0.417822	0.000300
H	-3.071788	2.145490	0.000335
H	3.664834	0.474329	0.000437

Table S67. Coordinates for the optimized geometry of K-**2a** (S_0).

Atom	*x*	*y*	*z*
C	1.429980	0.757618	-0.071959
C	1.045673	-0.617647	0.003106
C	-0.279181	-1.112509	-0.018823
C	0.529835	1.895831	-0.184033
C	-1.530515	-0.496249	-0.008584
C	-0.808770	1.990626	-0.116856
C	-1.839520	0.967643	0.121723
H	-0.315674	-2.201639	-0.039076
H	1.051954	2.839645	-0.321469
H	-1.250474	2.980273	-0.190071
C	-2.585647	-1.414693	-0.106223
H	-2.358826	-2.480434	-0.173524
O	-3.827360	-1.074974	-0.148734
O	-2.968917	1.315071	0.403272
C	2.056189	-1.613594	0.081937
C	2.797304	1.052333	-0.059932
C	3.394057	-1.287909	0.107681
C	3.762558	0.058194	0.035801

H	1.760451	-2.657695	0.129207
H	4.148128	-2.063545	0.176866
H	4.812180	0.333651	0.049031
H	3.111453	2.089068	-0.124268
H	-4.420749	-1.837964	-0.246029

Table S68. Coordinates for the optimized geometry of E-**2a** (T_1).

Atom	*x*	*y*	*z*
C	1.419678	0.762466	-0.035573
C	1.058798	-0.631375	0.030473
C	-0.239198	-1.158624	0.036625
C	0.503468	1.832033	-0.060307
C	-1.535052	-0.582014	0.002269
C	-0.876888	1.875008	-0.007592
C	-1.810817	0.840605	0.051388
H	-0.259183	-2.245880	0.056507
H	0.965399	2.815460	-0.108247
H	-1.331870	2.860424	0.004572
C	-2.614026	-1.477974	-0.061231
H	-2.467735	-2.565077	-0.050033
O	-3.867402	-1.163714	-0.147202
O	-3.052935	1.284868	0.141338
C	2.118038	-1.589050	0.070592
C	2.797130	1.109452	-0.067808
C	3.434086	-1.210850	0.043043
C	3.782295	0.156246	-0.029462
H	1.863820	-2.643126	0.121992
H	4.213490	-1.964651	0.075447
H	4.826114	0.449253	-0.053583
H	3.064846	2.160165	-0.120853
H	-3.737248	0.608656	0.286616

Table S69. Coordinates for the optimized geometry of K-**2a** (T_1).

Atom	*x*	*y*	*z*
C	1.441485	0.761091	0.000044
C	1.045356	-0.630326	-0.000064
C	-0.257830	-1.127938	-0.000065
C	0.561815	1.852954	0.000094
C	-1.553007	-0.537990	0.000012
C	-0.825245	1.940391	0.000053
C	-1.761277	0.908898	-0.000021
H	-0.305245	-2.214935	-0.000166
H	1.047721	2.825955	0.000240
H	-1.250583	2.938218	0.000106
C	-2.644119	-1.405872	0.000065

H	-2.471597	-2.477239	0.000049
O	-3.932174	-1.135276	0.000109
O	-3.008441	1.270137	-0.000070
C	2.084756	-1.614912	-0.000177
C	2.831077	1.072146	0.000112
C	3.407626	-1.268719	-0.000139
C	3.789526	0.094221	0.000015
H	1.804084	-2.663501	-0.000276
H	4.169011	-2.041293	-0.000214
H	4.840568	0.361369	0.000056
H	3.126466	2.116754	0.000227
H	-4.156486	-0.187872	0.000086

Table S70. Coordinates for the optimized geometry of E-**2a** (S_1).

Atom	*x*	*y*	*z*
C	1.414896	0.765327	-0.043667
C	1.056565	-0.628456	0.041081
C	-0.239134	-1.156156	0.048146
C	0.500428	1.834163	-0.076786
C	-1.526597	-0.577880	-0.000916
C	-0.880035	1.875088	-0.010910
C	-1.811895	0.839802	0.065278
H	-0.257444	-2.243230	0.074922
H	0.961324	2.817058	-0.138406
H	-1.336478	2.859839	0.007733
C	-2.618959	-1.471346	-0.084357
H	-2.447050	-2.562133	-0.064459
O	-3.848217	-1.168812	-0.182608
O	-3.054747	1.276058	0.182319
C	2.116107	-1.588652	0.090088
C	2.795362	1.109518	-0.085768
C	3.429666	-1.212264	0.054395
C	3.776777	0.156491	-0.038027
H	1.859878	-2.641425	0.155556
H	4.209954	-1.964584	0.095453
H	4.820935	0.447835	-0.069656
H	3.063813	2.159026	-0.153580
H	-3.730320	0.595838	0.343406

Table S71. Coordinates for the optimized geometry of K-**2a** (S_1).

Atom	*x*	*y*	*z*
C	1.453034	0.736670	0.000028
C	1.042246	-0.644581	-0.000054
C	-0.298795	-1.155773	-0.000056
C	0.579335	1.849696	0.000102

C	-1.567788	-0.558898	0.000003
C	-0.796854	1.923141	0.000104
C	-1.829168	0.887327	0.000028
H	-0.328076	-2.242669	-0.000120
H	1.072606	2.819188	0.000172
H	-1.238353	2.916112	0.000177
C	-2.677583	-1.421439	0.000005
H	-2.526041	-2.497991	0.000016
O	-3.934119	-1.091095	-0.000042
O	-2.993245	1.307464	0.000144
C	2.062780	-1.592479	-0.000134
C	2.848228	1.059947	0.000040
C	3.428993	-1.244159	-0.000129
C	3.832615	0.092827	-0.000039
H	1.805817	-2.647022	-0.000203
H	4.170025	-2.037139	-0.000195
H	4.881775	0.361849	-0.000031
H	3.126986	2.109550	0.000111
H	-4.008093	-0.106511	-0.000134

Table S72. Coordinates for the optimized geometry of E-**2b** (S_0).

Atom	*x*	*y*	*z*
C	-2.964061	-0.011097	0.163956
C	-1.802904	-0.720651	-0.262615
C	-0.472782	-0.201194	-0.320157
C	-2.991480	1.372867	0.537683
C	0.080291	1.039418	-0.116087
C	-2.033154	2.336571	0.511964
C	-0.665861	2.249136	0.123054
H	0.256184	-0.957913	-0.598228
H	-3.968850	1.722778	0.862068
H	-2.328765	3.345945	0.782382
C	1.578456	1.178828	-0.266883
O	2.041455	2.272805	-0.593048
O	-0.082735	3.402696	0.031023
C	-1.937509	-2.081471	-0.623212
C	-4.193067	-0.698264	0.218312
C	-3.156378	-2.723647	-0.573972
C	-4.293358	-2.026214	-0.145799
H	-1.056390	-2.628395	-0.944701
H	-3.232729	-3.766684	-0.861129
H	-5.253668	-2.528557	-0.099255
H	-5.078709	-0.164337	0.548256
H	0.864604	3.266501	-0.289379
C	2.479581	0.034734	-0.022668
C	2.225348	-0.923021	0.970645
C	3.672391	-0.021017	-0.756402

C	3.149053	-1.931550	1.211789
H	1.332981	-0.857627	1.586644
C	4.579088	-1.045337	-0.526034
H	3.872506	0.738876	-1.504389
C	4.318161	-1.999188	0.456746
H	2.962638	-2.658922	1.994748
H	5.494808	-1.096250	-1.105221
H	5.034732	-2.792844	0.642476

Table S73. Coordinates for the optimized geometry of K-**2b** (S_0).

Atom	*x*	*y*	*z*
C	-3.003034	0.053602	0.045359
C	-1.833955	-0.748429	0.166485
C	-0.485035	-0.268826	0.235660
C	-3.039914	1.507538	-0.031238
C	0.064500	0.978544	0.070632
C	-2.066370	2.418967	-0.197532
C	-0.623133	2.232896	-0.388135
H	0.223940	-1.051929	0.494335
H	-4.047981	1.912837	0.018523
H	-2.353753	3.462831	-0.285208
C	1.474823	1.093343	0.346987
O	1.858590	2.217525	0.869696
O	0.040904	3.103982	-0.917920
C	-1.973590	-2.151147	0.270590
C	-4.245427	-0.595383	0.026927
C	-3.209530	-2.764335	0.226193
C	-4.354258	-1.975730	0.103246
H	-1.080659	-2.759845	0.382001
H	-3.288338	-3.843513	0.296198
H	-5.334866	-2.439380	0.075768
H	-5.147126	0.003595	-0.053790
H	2.827265	2.294136	0.907739
C	2.458954	0.051154	0.109409
C	3.552750	-0.095709	0.984536
C	2.355597	-0.767090	-1.029812
C	4.516977	-1.055383	0.725693
H	3.616998	0.493220	1.896582
C	3.339479	-1.709634	-1.290040
H	1.534921	-0.625530	-1.725399
C	4.413607	-1.855627	-0.413849
H	5.346324	-1.187757	1.411688
H	3.274901	-2.324795	-2.180773
H	5.177779	-2.598420	-0.618541

Table S74. Coordinates for the optimized geometry of E-**2b** (T_1).

Atom	*x*	*y*	*z*
C	-3.029289	0.006897	0.013622
C	-1.823605	-0.768116	-0.127682
C	-0.512530	-0.277229	-0.160133
C	-3.073283	1.412016	0.130849
C	0.063520	1.018672	-0.052799
C	-2.086737	2.374646	0.156005
C	-0.693513	2.244448	0.084927
H	0.232479	-1.056152	-0.305749
H	-4.078060	1.819451	0.220256
H	-2.410979	3.404730	0.264158
C	1.464915	1.116042	-0.111429
O	2.182580	2.184096	-0.144403
O	-0.072474	3.401730	0.176088
C	-1.965760	-2.185556	-0.257799
C	-4.280269	-0.667963	0.027228
C	-3.190320	-2.795964	-0.240429
C	-4.369511	-2.030159	-0.094052
H	-1.067993	-2.785531	-0.371935
H	-3.257687	-3.874254	-0.340329
H	-5.337311	-2.519035	-0.081019
H	-5.184154	-0.075942	0.135121
H	0.903557	3.316781	0.169306
C	2.477760	0.036594	-0.009147
C	2.542406	-0.785128	1.138845
C	3.459450	-0.067664	-1.025091
C	3.505168	-1.769138	1.204049
H	1.820045	-0.653363	1.937829
C	4.422129	-1.059232	-0.942273
H	3.431816	0.618950	-1.863593
C	4.447206	-1.905168	0.166715
H	3.545855	-2.434125	2.059998
H	5.161949	-1.165748	-1.727630
H	5.213205	-2.670359	0.241670

Table S75. Coordinates for the optimized geometry of K-**2b** (T_1).

Atom	*x*	*y*	*z*
C	2.974554	0.019332	0.171817
C	1.762068	-0.681463	-0.292499
C	0.451697	-0.175858	-0.365183
C	3.066940	1.364321	0.410561
C	-0.126033	1.075019	-0.084388
C	2.093870	2.388118	0.264346
C	0.660366	2.325683	0.027611
H	-0.261979	-0.918509	-0.708218
H	4.044426	1.730693	0.715470
H	2.438081	3.412837	0.364372

C	-1.548130	1.173249	-0.064890
O	-2.164886	2.319657	-0.139405
O	0.095975	3.426685	-0.075854
C	1.918565	-2.035685	-0.662222
C	4.205030	-0.755095	0.323565
C	3.120838	-2.707827	-0.539730
C	4.283362	-2.061697	-0.021226
H	1.054252	-2.575242	-1.037200
H	3.175468	-3.751750	-0.831553
H	5.209301	-2.614521	0.084248
H	5.077564	-0.234967	0.707043
H	-1.488557	3.046656	-0.217435
C	-2.455618	0.027694	0.050281
C	-3.669702	0.062352	-0.652728
C	-2.174868	-1.054792	0.899327
C	-4.565171	-0.990708	-0.539883
H	-3.896004	0.908838	-1.291877
C	-3.085889	-2.094775	1.021722
H	-1.269302	-1.058244	1.498354
C	-4.274709	-2.068480	0.295389
H	-5.495166	-0.969023	-1.097723
H	-2.876255	-2.919059	1.695129
H	-4.983548	-2.884780	0.390235

Table S76. Coordinates for the optimized geometry of E-**2b** (S_1).

Atom	*x*	*y*	*z*
C	-3.011972	-0.026367	0.000202
C	-1.789843	-0.786148	-0.000337
C	-0.485780	-0.272197	-0.000646
C	-3.086108	1.381098	0.000261
C	0.056024	1.036873	-0.000218
C	-2.119272	2.364581	0.000055
C	-0.723426	2.254912	-0.000111
H	0.273040	-1.050394	-0.001183
H	-4.101318	1.772019	0.000515
H	-2.465059	3.393218	0.000117
C	1.457679	1.172745	-0.000156
O	2.172124	2.221964	-0.000134
O	-0.111834	3.419175	-0.000158
C	-1.903980	-2.212120	-0.000602
C	-4.252928	-0.724621	0.000576
C	-3.117053	-2.842616	-0.000224
C	-4.315593	-2.091263	0.000401
H	-0.992747	-2.802107	-0.001081
H	-3.161971	-3.926645	-0.000403
H	-5.274592	-2.596923	0.000709
H	-5.169487	-0.142394	0.001000

H	0.868058	3.323846	-0.000258
C	2.477901	0.088662	-0.000071
C	3.007260	-0.377351	1.234270
C	3.007382	-0.377806	-1.234183
C	3.919926	-1.407191	1.227096
H	2.657785	0.061271	2.161779
C	3.920027	-1.407616	-1.226491
H	2.657922	0.060435	-2.161879
C	4.376693	-1.921846	0.000443
H	4.302393	-1.809034	2.158234
H	4.302612	-1.809864	-2.157405
H	5.119420	-2.712911	0.000595

Table S77. Coordinates for the optimized geometry of K-**2b** (S_1).

Atom	*x*	*y*	*z*
C	2.974352	0.011195	0.138433
C	1.776653	-0.685330	-0.301370
C	0.458927	-0.154557	-0.430595
C	3.051732	1.399899	0.368761
C	-0.133039	1.080816	-0.110212
C	2.091293	2.382093	0.258752
C	0.650210	2.317187	0.035985
H	-0.243055	-0.890750	-0.807625
H	4.041020	1.769675	0.629293
H	2.417504	3.409970	0.391706
C	-1.556647	1.175718	-0.065523
O	-2.195449	2.321774	-0.122323
O	0.090806	3.425330	-0.011269
C	1.925235	-2.046197	-0.608608
C	4.196554	-0.713157	0.287538
C	3.137637	-2.716591	-0.466017
C	4.289450	-2.052165	0.000476
H	1.061048	-2.601363	-0.958952
H	3.190379	-3.770881	-0.718303
H	5.226170	-2.584349	0.115206
H	5.073484	-0.170141	0.626822
H	-1.542039	3.058233	-0.188645
C	-2.453043	0.022946	0.042392
C	-3.699402	0.078801	-0.604258
C	-2.137541	-1.093866	0.836552
C	-4.588795	-0.978801	-0.491981
H	-3.954013	0.947871	-1.200668
C	-3.041248	-2.138456	0.959315
H	-1.205249	-1.121103	1.392093
C	-4.262182	-2.087759	0.287664
H	-5.542111	-0.937733	-1.007886
H	-2.801650	-2.987726	1.590441

H	-4.965216	-2.909184	0.381435

Table S78. Coordinates for the optimized geometry of E-**3a** (S_0).

Atom	*x*	*y*	*z*
C	0.766807	2.552131	-0.073645
C	-0.595101	2.608389	0.054414
C	-1.487429	1.517554	0.145537
C	1.587644	1.396844	-0.103261
C	-1.276360	0.158916	0.050124
C	1.283586	0.058495	-0.090454
C	-0.026937	-0.536525	-0.133093
H	1.293677	3.500537	-0.128733
H	-1.048683	3.593890	0.099273
H	-2.528747	1.805554	0.284943
H	2.654956	1.614715	-0.133682
C	-2.524544	-0.669202	0.128170
H	-3.455381	-0.123416	0.349388
O	-2.541901	-1.871208	-0.042502
O	-0.036899	-1.811068	-0.334917
C	2.505347	-0.854151	-0.003593
H	3.445598	-0.357605	-0.312930
O	2.480111	-1.974590	0.415346
H	-0.969998	-2.173456	-0.306871

Table S79. Coordinates for the optimized geometry of K-**3a** (S_0).

Atom	*x*	*y*	*z*
C	1.047917	2.438243	-0.141239
C	-0.289849	2.660256	0.041449
C	-1.293749	1.685632	0.228292
C	1.767501	1.198911	-0.122403
C	-1.233197	0.304726	0.094425
C	1.319314	-0.083576	-0.106818
C	-0.071608	-0.542716	-0.252442
H	1.668691	3.323870	-0.250928
H	-0.621105	3.693099	0.075972
H	-2.276089	2.091999	0.465539
H	2.850619	1.316511	-0.104508
C	-2.487631	-0.353092	0.238968
H	-3.382157	0.169140	0.572153
O	-2.696313	-1.639275	-0.016972
O	-0.283236	-1.720512	-0.629645
C	2.381585	-1.151774	0.090194
H	3.405987	-0.827167	-0.176343
O	2.147258	-2.227067	0.563799
H	-1.829308	-2.012275	-0.341897

Table S80. Coordinates for the optimized geometry of E-**3a** (T_1).

Atom	*x*	*y*	*z*
C	1.157836	2.434727	-0.000135
C	-0.231982	2.675032	-0.000025
C	-1.255014	1.746926	0.000095
C	1.818941	1.232094	-0.000096
C	-1.229639	0.337658	0.000054
C	1.300312	-0.098973	-0.000008
C	-0.086572	-0.504770	-0.000093
H	1.790103	3.317490	-0.000228
H	-0.535343	3.717169	-0.000008
H	-2.257420	2.171729	0.000232
H	2.904280	1.295611	-0.000148
C	-2.542345	-0.331807	0.000260
H	-3.431966	0.314724	0.000093
O	-2.682576	-1.549794	0.000222
O	-0.243535	-1.795611	-0.000392
H	-1.245829	-2.024230	-0.000498
C	2.229724	-1.150873	0.000088
H	3.310234	-0.944801	0.000170
O	1.988407	-2.390569	0.000112

Table S81. Coordinates for the optimized geometry of K-**3a** (T_1).

Atom	*x*	*y*	*z*
C	-2.034899	1.803267	0.000067
C	-0.834373	2.538683	0.000128
C	0.465922	2.080776	0.000101
C	-2.191226	0.431192	-0.000015
C	1.002855	0.760416	0.000052
C	-1.210488	-0.580063	-0.000020
C	0.235945	-0.483472	0.000139
H	-2.950551	2.386100	0.000070
H	-0.946057	3.618617	0.000169
H	1.220827	2.864135	0.000096
H	-3.218654	0.073879	-0.000064
C	2.403138	0.674217	-0.000146
H	3.000477	1.583398	-0.000308
O	3.127396	-0.391831	-0.000097
O	0.865146	-1.569848	-0.000806
C	-1.605876	-1.946828	-0.000140
H	-2.666617	-2.229967	-0.000007
O	-0.790700	-2.883960	0.000762
H	2.559857	-1.200172	0.000174

Table S82. Coordinates for the optimized geometry of E-**3a** (S_1).

Atom	*x*	*y*	*z*
C	-1.111075	2.453566	-0.000003

C	0.283487	2.669532	-0.000047
C	1.286017	1.728092	-0.000049
C	-1.790683	1.260989	0.000042
C	1.236384	0.314180	-0.000014
C	-1.287000	-0.068463	0.000052
C	0.081393	-0.511706	0.000017
H	-1.729274	3.346138	-0.000002
H	0.603452	3.706575	-0.000080
H	2.296135	2.133448	-0.000077
H	-2.874034	1.344484	0.000073
C	2.530525	-0.379436	-0.000054
H	3.433299	0.248220	-0.000142
O	2.649704	-1.601885	-0.000069
O	0.220702	-1.804007	0.000014
H	1.221768	-2.046847	-0.000045
C	-2.248484	-1.123498	0.000083
H	-3.326817	-0.859362	0.000084
O	-2.058896	-2.335632	0.000058

Table S83. Coordinates for the optimized geometry of K-**3a** (S_1).

Atom	*x*	*y*	*z*
C	1.906717	-1.928708	0.000094
C	0.663705	-2.588275	0.000000
C	-0.600493	-2.047734	-0.000083
C	2.152901	-0.570746	0.000102
C	-1.053425	-0.698978	-0.000083
C	1.237985	0.494409	0.000030
C	-0.209196	0.500856	-0.000058
H	2.783787	-2.568401	0.000118
H	0.707585	-3.672855	-0.000014
H	-1.405415	-2.779933	-0.000156
H	3.201168	-0.282242	0.000115
C	-2.444595	-0.516399	-0.000018
H	-3.100689	-1.384019	0.000127
O	-3.096487	0.596344	-0.000121
O	-0.775139	1.612817	0.000557
C	1.734698	1.847629	0.000050
H	2.823462	2.028373	0.000234
O	1.013665	2.833645	-0.000471
H	-2.475996	1.364296	-0.000335

Table S84. Coordinates for the optimized geometry of *syn*-E-**3b** (S_0).

Atom	*x*	*y*	*z*
C	-0.369533	3.036661	-0.517403
C	0.902947	2.732770	-0.934078
C	1.584609	1.506889	-0.801969
C	-1.304055	2.176905	0.100217
C	1.149671	0.263415	-0.382075
C	-1.245079	0.821820	0.308092
C	-0.202709	-0.085091	-0.058123
H	-0.696003	4.063754	-0.654975
H	1.479396	3.540356	-1.375327
H	2.627204	1.541539	-1.106266
H	-2.227371	2.653029	0.424199
C	2.161217	-0.871570	-0.381694
O	1.758395	-2.008564	-0.623354
O	-0.565258	-1.333211	-0.003418
C	-2.400018	0.195246	1.107400
O	-2.202984	0.107126	2.299287
H	0.201958	-1.913766	-0.293285
C	-3.636377	-0.189988	0.411916
C	-4.704884	-0.671798	1.179066
C	-3.757600	-0.094443	-0.979163
C	-5.884751	-1.048399	0.555650
H	-4.590780	-0.743420	2.255660
C	-4.940269	-0.475378	-1.599066
H	-2.931101	0.271353	-1.582812
C	-6.001826	-0.949834	-0.831034
H	-6.715033	-1.420474	1.146402
H	-5.036371	-0.405071	-2.677353
H	-6.926346	-1.246332	-1.316317
C	3.582217	-0.612310	-0.097425
C	4.526270	-1.489127	-0.651500
C	4.000574	0.410410	0.768374
C	5.873837	-1.319392	-0.372299
H	4.190437	-2.290646	-1.300827
C	5.349426	0.558907	1.060938
H	3.276084	1.060592	1.250122
C	6.284773	-0.297131	0.482618
H	6.605384	-1.987952	-0.813118
H	5.671376	1.335828	1.745993
H	7.339295	-0.172956	0.707111

Table S85. Coordinates for the optimized geometry of *syn*-K-**3b** (S_0).

Atom	*x*	*y*	*z*
C	0.322878	-3.509293	-0.901397
C	-0.996008	-3.217967	-0.688945

C	-1.543571	-2.016818	-0.173503
C	1.468161	-2.700125	-0.632329
C	-0.923037	-0.937321	0.421932
C	1.557893	-1.536583	0.067321
C	0.494785	-0.859649	0.807587
H	0.536594	-4.468228	-1.366883
H	-1.721587	-3.966456	-0.992869
H	-2.628531	-1.970519	-0.216282
H	2.412526	-3.096058	-0.999757
C	-1.768837	0.176477	0.881514
O	-1.365326	0.899964	1.856140
O	0.829796	-0.129354	1.758519
C	2.957853	-0.950859	0.267547
O	3.807549	-1.682046	0.730123
H	-0.407848	0.589713	2.112396
C	3.204784	0.450196	-0.142213
C	4.368955	1.079835	0.312167
C	2.326778	1.135987	-0.989834
C	4.640495	2.388611	-0.066186
H	5.044985	0.534682	0.963106
C	2.606780	2.441779	-1.376023
H	1.435551	0.646394	-1.377891
C	3.761151	3.069165	-0.908852
H	5.539145	2.879755	0.293117
H	1.933063	2.968409	-2.044801
H	3.978793	4.090297	-1.206943
C	-3.046564	0.530802	0.293250
C	-3.308188	0.336402	-1.077473
C	-4.012299	1.147808	1.111830
C	-4.524228	0.737819	-1.609931
H	-2.546026	-0.074276	-1.732588
C	-5.232996	1.522318	0.573313
H	-3.803722	1.306497	2.164732
C	-5.488439	1.318114	-0.784315
H	-4.718991	0.609772	-2.669481
H	-5.986082	1.978248	1.207201
H	-6.442084	1.623063	-1.204076

Table S86. Coordinates for the optimized geometry of *syn*-E-**3b** (T_1).

Atom	*x*	*y*	*z*
C	0.698971	2.403868	-1.099091
C	-0.705046	2.359349	-1.215017
C	-1.553805	1.314868	-0.906923
C	1.549325	1.404414	-0.704124
C	-1.298927	0.002465	-0.454941
C	1.277026	0.050907	-0.343346
C	-0.010505	-0.611384	-0.352435
H	1.168598	3.346781	-1.362879
H	-1.183115	3.263313	-1.579416
H	-2.605970	1.530769	-1.063559
H	2.603267	1.665993	-0.695213
C	-2.470888	-0.876742	-0.171418
O	-2.330008	-2.111625	-0.173117
O	0.063341	-1.896577	-0.200338
H	-0.907331	-2.277167	-0.209466
C	-3.806409	-0.320326	0.132644
C	-4.927371	-1.038940	-0.302279
C	-3.976284	0.829311	0.916871
C	-6.202296	-0.588118	0.008754
H	-4.787069	-1.942587	-0.885929
C	-5.254920	1.260685	1.245248
H	-3.113952	1.364736	1.303559
C	-6.366329	0.559953	0.781875
H	-7.069554	-1.136409	-0.343404
H	-5.384772	2.139505	1.867946
H	-7.364477	0.904742	1.032325
C	2.381690	-0.758976	0.012143
O	2.368406	-1.993271	0.272668
C	3.794011	-0.307520	0.213437
C	4.800935	-0.882236	-0.576182
C	4.101611	0.601892	1.237474
C	6.116341	-0.477668	-0.390372
H	4.546847	-1.618824	-1.330765
C	5.419522	0.989696	1.412893
H	3.315456	1.000714	1.870813
C	6.423369	0.452175	0.600131
H	6.901960	-0.897884	-1.008538
H	5.672012	1.706286	2.187000
H	7.454072	0.755785	0.751768

Table S87. Coordinates for the optimized geometry of *syn*-K-**3b** (T_1).

Atom	*x*	*y*	*z*
C	0.698971	2.403868	-1.099091
C	-0.705046	2.359349	-1.215017
C	-1.553805	1.314868	-0.906923
C	1.549325	1.404414	-0.704124
C	-1.298927	0.002465	-0.454941
C	1.277026	0.050907	-0.343346
C	-0.010505	-0.611384	-0.352435
H	1.168598	3.346781	-1.362879
H	-1.183115	3.263313	-1.579416
H	-2.605970	1.530769	-1.063559
H	2.603267	1.665993	-0.695213
C	-2.470888	-0.876742	-0.171418
O	-2.330008	-2.111625	-0.173117
O	0.063341	-1.896577	-0.200338
H	-0.907331	-2.277167	-0.209466
C	-3.806409	-0.320326	0.132644
C	-4.927371	-1.038940	-0.302279
C	-3.976284	0.829311	0.916871
C	-6.202296	-0.588118	0.008754
H	-4.787069	-1.942587	-0.885929
C	-5.254920	1.260685	1.245248
H	-3.113952	1.364736	1.303559
C	-6.366329	0.559953	0.781875
H	-7.069554	-1.136409	-0.343404
H	-5.384772	2.139505	1.867946
H	-7.364477	0.904742	1.032325
C	2.381690	-0.758976	0.012143
O	2.368406	-1.993271	0.272668
C	3.794011	-0.307520	0.213437
C	4.800935	-0.882236	-0.576182
C	4.101611	0.601892	1.237474
C	6.116341	-0.477668	-0.390372
H	4.546847	-1.618824	-1.330765
C	5.419522	0.989696	1.412893
H	3.315456	1.000714	1.870813
C	6.423369	0.452175	0.600131
H	6.901960	-0.897884	-1.008538
H	5.672012	1.706286	2.187000
H	7.454072	0.755785	0.751768

Table S88. Coordinates for the optimized geometry of *syn*-E-**3b** (S_1).

Atom	*x*	*y*	*z*
C	0.711157	2.447694	-0.722567
C	-0.679476	2.410809	-0.945843
C	-1.532123	1.345376	-0.775901
C	1.541417	1.408404	-0.387641
C	-1.299792	0.001014	-0.393120
C	1.263458	0.026432	-0.210185
C	-0.028071	-0.626265	-0.271179
H	1.188503	3.415720	-0.840729
H	-1.138629	3.340751	-1.265354
H	-2.574177	1.567226	-0.982500
H	2.585674	1.679386	-0.270712
C	-2.495025	-0.876034	-0.211033
O	-2.379627	-2.108547	-0.280890
O	0.039886	-1.919965	-0.162122
H	-0.908455	-2.296407	-0.242694
C	-3.834245	-0.308865	0.075126
C	-4.949417	-0.970128	-0.453223
C	-4.014742	0.787702	0.928399
C	-6.227700	-0.513602	-0.163700
H	-4.802021	-1.835552	-1.090616
C	-5.297545	1.224495	1.234107
H	-3.158514	1.278691	1.381981
C	-6.402136	0.582159	0.679541
H	-7.089757	-1.017419	-0.587820
H	-5.435761	2.062338	1.909361
H	-7.403005	0.931299	0.912510
C	2.390189	-0.810380	0.047454
O	2.477914	-2.027270	0.231151
C	3.817524	-0.351680	0.171759
C	4.645135	-0.357004	-0.969530
C	4.326444	-0.033030	1.447277
C	5.942624	0.102684	-0.850003
H	4.252459	-0.690291	-1.923673
C	5.626493	0.424921	1.547774
H	3.692738	-0.121933	2.322593
C	6.431277	0.493001	0.403663
H	6.586289	0.144392	-1.721242
H	6.027754	0.712793	2.512769
H	7.458843	0.829040	0.493963

Table S89. Coordinates for the optimized geometry of *syn*-K-**3b** (S_1).

Atom	*x*	*y*	*z*
C	0.674953	2.412683	-1.155412
C	-0.724228	2.342371	-1.264804
C	-1.561725	1.303398	-0.929681
C	1.547585	1.431404	-0.737613
C	-1.298735	-0.014309	-0.468093
C	1.284658	0.094529	-0.388588
C	0.032463	-0.639780	-0.444119
H	1.127437	3.361248	-1.428503
H	-1.215141	3.234335	-1.641386
H	-2.615808	1.516673	-1.073650
H	2.595239	1.714184	-0.717208
C	-2.413880	-0.829930	-0.100773
O	-2.325348	-2.121898	0.030844
O	0.105028	-1.886159	-0.382860
C	2.395358	-0.781372	-0.048107
O	2.235369	-2.000350	0.063487
H	-1.397416	-2.415616	-0.171879
C	3.790851	-0.313528	0.205666
C	4.844985	-0.986580	-0.419770
C	4.032480	0.716708	1.122043
C	6.149621	-0.580063	-0.169814
H	4.640234	-1.803812	-1.102852
C	5.342810	1.099656	1.375018
H	3.210609	1.199873	1.641063
C	6.397096	0.457883	0.725250
H	6.973234	-1.081796	-0.665876
H	5.542156	1.895972	2.083951
H	7.418419	0.763904	0.926657
C	-3.758010	-0.313697	0.181454
C	-3.953155	0.863734	0.919931
C	-4.865616	-1.070288	-0.229532
C	-5.240287	1.286218	1.221162
H	-3.100870	1.423353	1.293000
C	-6.148750	-0.628856	0.057842
H	-4.713403	-1.990514	-0.783019
C	-6.336850	0.547237	0.781894
H	-5.388457	2.186713	1.807451
H	-7.004196	-1.205590	-0.276874
H	-7.342175	0.883808	1.013622

Table S90. Coordinates for the optimized geometry of *anti*-E-**3c** (S_0).

Atom	*x*	*y*	*z*
C	0.159603	-3.60461	-1.55788
C	-1.13711	-3.28225	-1.24488
C	-1.58955	-2.22977	-0.42335
C	1.34111	-2.98208	-1.09728
C	-0.90109	-1.34553	0.386055
C	1.513379	-1.9934	-0.15932
C	0.511145	-1.3678	0.647179
H	0.304763	-4.4265	-2.25337
H	-1.91684	-3.87708	-1.71115
H	-2.66991	-2.11424	-0.40522
H	2.264987	-3.37736	-1.51302
C	-1.71866	-0.33327	1.170307
O	-1.30009	0.042858	2.261991
O	0.999171	-0.72267	1.664833
H	0.250447	-0.32954	2.206236
C	-2.99045	0.195074	0.633531
C	-3.96534	0.595379	1.557253
C	-3.20404	0.388249	-0.73896
C	-5.15663	1.146165	1.114802
H	-3.78262	0.465019	2.618324
C	-4.38549	0.960026	-1.18423
H	-2.43776	0.131511	-1.46403
C	-5.36755	1.327799	-0.25741
H	-5.92234	1.44316	1.822518
H	-4.55207	1.129024	-2.24206
C	2.975842	-1.66237	0.185485
O	3.665571	-2.58386	0.557779
C	3.470384	-0.27992	-0.00369
C	2.733006	0.683302	-0.69925
C	4.732071	0.038704	0.509203
C	3.244415	1.95889	-0.87764
H	1.763721	0.44099	-1.12705
C	5.246376	1.314545	0.343534
H	5.296149	-0.72126	1.038832
C	4.501194	2.275838	-0.34967
H	2.684705	2.710461	-1.42286
H	6.21996	1.573402	0.744374
C	5.026942	3.601594	-0.52592
C	-6.60001	1.901553	-0.72223
N	5.442533	4.673017	-0.66964
N	-7.59365	2.359124	-1.1021

Table S91. Coordinates for the optimized geometry of *anti*-K-**3c** (S_0).

Atom	*x*	*y*	*z*
C	0.230362	-3.86616	-1.25369
C	-1.05858	-3.50962	-0.97196
C	-1.50594	-2.34744	-0.29754
C	1.436659	-3.17653	-0.92779
C	-0.79567	-1.38957	0.397285
C	1.628337	-2.09848	-0.12001
C	0.637771	-1.43646	0.7235
H	0.364026	-4.77766	-1.83019
H	-1.84269	-4.16409	-1.3394
H	-2.58575	-2.2269	-0.29196
H	2.343252	-3.59959	-1.35434
C	-1.54858	-0.29644	1.033671
O	-1.05282	0.283025	2.05471
O	1.056333	-0.82484	1.725474
C	3.07762	-1.64844	0.087598
O	3.900662	-2.49972	0.337376
H	-0.08989	-0.11196	2.210087
C	3.414819	-0.21126	-0.07474
C	4.641139	0.235556	0.425033
C	2.56843	0.676175	-0.74568
C	5.009173	1.563826	0.278305
H	5.294076	-0.4671	0.931161
C	2.934303	2.002838	-0.9093
H	1.62937	0.331969	-1.17176
C	4.153959	2.448979	-0.38802
H	5.953736	1.921324	0.672758
H	2.290551	2.695793	-1.43947
C	-2.83728	0.196901	0.569483
C	-3.18074	0.194948	-0.79441
C	-3.71494	0.756467	1.514967
C	-4.39241	0.724597	-1.20307
H	-2.48391	-0.17098	-1.54106
C	-4.93537	1.265339	1.109077
H	-3.44164	0.770553	2.564236
C	-5.27524	1.24826	-0.25025
H	-4.65795	0.745665	-2.25383
H	-5.62689	1.680364	1.833427
C	4.525246	3.828589	-0.54337
C	-6.53919	1.781069	-0.67538
N	4.813102	4.943457	-0.66871
N	-7.55925	2.205972	-1.02163

Table S92. Coordinates for the optimized geometry of *syn*-E-**3c** (T_1).

Atom	*x*	*y*	*z*
C	0.693856	-2.08876	1.504716
C	-0.70982	-2.02717	1.611792
C	-1.5561	-1.02684	1.175988
C	1.54787	-1.15102	0.98419
C	-1.29744	0.219272	0.565183
C	1.278216	0.143709	0.448109
C	-0.00756	0.807008	0.37718
H	1.160859	-2.99054	1.889151
H	-1.19002	-2.8772	2.08635
H	-2.60825	-1.21957	1.360737
H	2.60065	-1.4153	1.012689
C	-2.46509	1.063556	0.182053
O	-2.32202	2.284219	0.0131
O	0.075027	2.061068	0.057387
H	-0.88652	2.45236	0.016374
C	-3.81381	0.483062	-0.02849
C	-4.9204	1.251324	0.351607
C	-4.00303	-0.74226	-0.68041
C	-6.20406	0.782268	0.12284
H	-4.76705	2.212696	0.829578
C	-5.28562	-1.20426	-0.93334
H	-3.15288	-1.32315	-1.02479
C	-6.3872	-0.44749	-0.51976
H	-7.0657	1.364146	0.43018
H	-5.44048	-2.14339	-1.45247
C	2.379204	0.899786	-0.01784
O	2.35238	2.094026	-0.43235
C	3.790586	0.422134	-0.16468
C	4.086696	-0.62355	-1.05109
C	4.80421	1.097724	0.527318
C	5.39894	-1.03887	-1.18867
H	3.296788	-1.10685	-1.61679
C	6.11752	0.673478	0.390786
H	4.562428	1.932561	1.175869
C	6.413919	-0.39309	-0.46446
H	5.649689	-1.85578	-1.85599
H	6.913838	1.169301	0.934089
C	7.773131	-0.83181	-0.61121
C	-7.71615	-0.93693	-0.76367
N	8.867757	-1.19167	-0.72774
N	-8.78543	-1.33711	-0.95784

Table S93. Coordinates for the optimized geometry of *syn*-K-**3c** (T_1).

Atom	*x*	*y*	*z*
C	0.640439	-2.16514	1.52029
C	-0.75746	-2.05217	1.627043
C	-1.58327	-1.03459	1.199569
C	1.528233	-1.24068	1.011719
C	-1.30127	0.217755	0.582544
C	1.287926	0.056356	0.514853
C	0.039293	0.79701	0.465629
H	1.075817	-3.08637	1.894579
H	-1.26196	-2.88802	2.102363
H	-2.63643	-1.20505	1.396455
H	2.573765	-1.53288	1.039826
C	-2.4063	1.003284	0.13626
O	-2.31159	2.269638	-0.1485
O	0.141826	2.02939	0.236912
C	2.384026	0.88329	0.086939
O	2.196696	2.100025	-0.15719
H	-1.38607	2.588771	0.00237
C	3.781899	0.419155	-0.11132
C	4.825812	1.188482	0.413389
C	4.048461	-0.72402	-0.87423
C	6.138275	0.788095	0.214447
H	4.610296	2.084691	0.984934
C	5.361746	-1.11378	-1.08647
H	3.238993	-1.2915	-1.32239
C	6.406279	-0.36304	-0.53429
H	6.956564	1.365248	0.629887
H	5.584157	-1.99244	-1.68137
C	-3.76183	0.47608	-0.07639
C	-3.97624	-0.77194	-0.67999
C	-4.85523	1.287702	0.258782
C	-5.26661	-1.21327	-0.92385
H	-3.13614	-1.38188	-0.99594
C	-6.14651	0.839283	0.035855
H	-4.69	2.261561	0.705908
C	-6.35343	-0.41267	-0.55502
H	-5.43895	-2.1692	-1.40525
H	-6.99638	1.453665	0.310678
C	7.765747	-0.77832	-0.74406
C	-7.69145	-0.87885	-0.79254
N	8.86001	-1.11838	-0.91104
N	-8.76863	-1.25981	-0.98137

Table S94. Coordinates for the optimized geometry of *syn*-E-**3c** (S_1).

Atom	*x*	*y*	*z*
C	0.708709	-2.21079	1.033526
C	-0.67905	-2.14294	1.266616
C	-1.52929	-1.0957	0.999152
C	1.540739	-1.21558	0.586567
C	-1.29574	0.201393	0.477411
C	1.265269	0.141814	0.270978
C	-0.02294	0.80409	0.271958
H	1.182374	-3.16462	1.244703
H	-1.1384	-3.03285	1.684576
H	-2.56875	-1.29119	1.242242
H	2.582707	-1.50282	0.488247
C	-2.48643	1.062812	0.221115
O	-2.36823	2.292566	0.142813
O	0.05265	2.077093	0.018428
H	-0.88743	2.473015	0.063758
C	-3.84089	0.479528	0.028966
C	-4.9348	1.19409	0.529077
C	-4.04975	-0.68642	-0.7173
C	-6.22363	0.727876	0.323278
H	-4.76749	2.112909	1.080431
C	-5.33851	-1.14437	-0.94674
H	-3.211	-1.22398	-1.14899
C	-6.42599	-0.44347	-0.41514
H	-7.07481	1.267895	0.722466
H	-5.50884	-2.03758	-1.53721
C	2.388716	0.947416	-0.08004
O	2.466344	2.140692	-0.38738
C	3.817899	0.478391	-0.16442
C	4.320137	0.034571	-1.40208
C	4.648558	0.601102	0.965295
C	5.620237	-0.42504	-1.46972
H	3.684345	0.029566	-2.28027
C	5.947802	0.140763	0.892012
H	4.261844	1.025304	1.88511
C	6.432434	-0.37755	-0.32265
H	6.023387	-0.80838	-2.39997
H	6.60009	0.186684	1.756461
C	7.784006	-0.84477	-0.39806
C	-7.76036	-0.92988	-0.63548
N	8.875528	-1.22947	-0.45705
N	-8.83368	-1.32778	-0.81129

Table S95. Coordinates for the optimized geometry of *syn*-K-**3c** (S_1).

Atom	*x*	*y*	*z*
C	0.664382	-2.12253	1.509862
C	-0.73436	-2.03303	1.612845
C	-1.5683	-1.0345	1.166182
C	1.541356	-1.19848	0.984043
C	-1.30189	0.222034	0.556236
C	1.283625	0.091169	0.48608
C	0.033826	0.829469	0.447911
H	1.112251	-3.03688	1.887183
H	-1.22811	-2.87486	2.088112
H	-2.6223	-1.22629	1.338332
H	2.587565	-1.48738	0.996221
C	-2.41235	0.997977	0.107927
O	-2.32353	2.261023	-0.19081
O	0.11117	2.057385	0.226676
C	2.3933	0.924525	0.048899
O	2.226868	2.119359	-0.21213
H	-1.39449	2.579902	-0.04567
C	3.795656	0.434128	-0.12583
C	4.835882	1.170344	0.447368
C	4.055206	-0.68669	-0.92161
C	6.145381	0.747451	0.27244
H	4.620863	2.055692	1.035435
C	5.36609	-1.09664	-1.10925
H	3.246108	-1.225	-1.4046
C	6.40983	-0.38502	-0.50467
H	6.963433	1.294996	0.726522
H	5.587355	-1.96147	-1.72441
C	-3.77118	0.465171	-0.08377
C	-3.99095	-0.7759	-0.69815
C	-4.86102	1.26677	0.282767
C	-5.28387	-1.22134	-0.9217
H	-3.15393	-1.3779	-1.03703
C	-6.15453	0.81455	0.079406
H	-4.69168	2.236459	0.737434
C	-6.36721	-0.43105	-0.52224
H	-5.46071	-2.17254	-1.41081
H	-7.0017	1.421671	0.377733
C	7.765838	-0.82214	-0.6914
C	-7.70743	-0.90123	-0.73919
N	8.857005	-1.1801	-0.83985
N	-8.78621	-1.28546	-0.91147

Table S96. Coordinates for the optimized geometry of *anti*-E-**3d** (S_0).

Atom	*x*	*y*	*z*
C	0.137247	5.285289	2.139954
C	-1.11395	4.923674	1.707944
C	-1.44014	4.011621	0.683401
C	1.38397	4.853643	1.635884
C	-0.64533	3.379626	-0.25251
C	1.677631	4.0971	0.52781
C	0.758996	3.604699	-0.45148
H	-2.50143	3.79379	0.597338
H	2.255283	5.214794	2.177128
C	-1.32637	2.463016	-1.25601
O	-0.87749	2.433393	-2.40506
O	1.316125	3.273637	-1.57659
H	0.604431	2.948852	-2.21268
C	-2.47119	1.643136	-0.86117
C	-3.34109	1.199585	-1.87264
C	-2.6828	1.202587	0.457173
C	-4.40812	0.378044	-1.57265
H	-3.15762	1.502247	-2.89805
C	-3.74193	0.368409	0.759112
H	-2.00512	1.491061	1.25516
C	-4.62556	-0.04767	-0.25068
H	-5.06159	0.022934	-2.36119
H	-3.91156	0.050576	1.781286
C	3.176284	3.920093	0.227268
O	3.833422	4.935452	0.136783
C	3.72361	2.558787	0.129625
C	2.987541	1.422564	0.487483
C	5.044128	2.405565	-0.31289
C	3.552419	0.162517	0.407044
H	1.975451	1.51508	0.87426
C	5.607519	1.147277	-0.41643
H	5.608734	3.287097	-0.59758
C	4.867701	0.011965	-0.05491
H	2.997198	-0.71228	0.726367
H	6.613864	1.027241	-0.80184
C	6.71069	-1.62919	0.308234
C	4.822427	-2.39241	-0.70951
C	7.664584	-0.8721	0.985552
C	6.908512	-2.99575	0.041232

C	3.588177	-2.51197	-1.34555
C	5.70797	-3.48106	-0.61078
C	8.843092	-1.50679	1.358289
H	7.499691	0.171336	1.231225
C	8.098469	-3.61268	0.425151
C	3.240741	-3.75948	-1.84974
H	2.923136	-1.66351	-1.46576
C	5.339058	-4.72367	-1.12389
C	9.065302	-2.86045	1.076883
H	9.604353	-0.93827	1.883161
H	8.261187	-4.66681	0.221901
C	4.100435	-4.85861	-1.73545
H	2.284451	-3.87925	-2.34959
H	6.015803	-5.56976	-1.05256
H	9.997927	-3.32468	1.380022
H	3.799142	-5.81948	-2.13951
C	-5.65715	-2.005	0.895568
C	-7.01715	-0.72512	-0.41767
C	-4.571	-2.60108	1.532754
C	-6.9443	-2.56615	0.958099
C	-7.56772	0.274557	-1.21655
C	-7.80958	-1.74951	0.128311
C	-4.81127	-3.75076	2.27631
H	-3.56438	-2.20766	1.443163
C	-7.16356	-3.71996	1.708308
C	-8.92765	0.200841	-1.49483
H	-6.97463	1.09622	-1.6025
C	-9.17157	-1.80425	-0.16193
C	-6.0937	-4.3025	2.373613
H	-3.982	-4.2327	2.784429
H	-8.15463	-4.15989	1.76218
C	-9.72329	-0.83007	-0.98225
H	-9.37937	0.964552	-2.11992
H	-9.79061	-2.59303	0.254337
H	-6.24786	-5.2004	2.962714
H	-10.7813	-0.85972	-1.22044
N	5.434552	-1.26053	-0.14903
N	-5.69783	-0.87682	0.053241
H	-1.95947	5.34801	2.241151
H	0.184853	5.964856	2.986209

Table S97. Coordinates for the optimized geometry of *anti*-K-**3d** (S_0).

Atom	*x*	*y*	*z*
C	0.314784	-5.84831	-1.65291
C	-0.90252	-5.44121	-1.18864
C	-1.18038	-4.32883	-0.34963
C	1.597127	-5.27061	-1.40107
C	-0.3215	-3.55293	0.392718
C	1.955491	-4.34937	-0.46861
C	1.109139	-3.83597	0.608512
H	-2.23734	-4.09813	-0.24447
H	2.414019	-5.64985	-2.01108
C	-0.87972	-2.45687	1.205246
O	-0.3057	-2.16741	2.323703
O	1.636944	-3.54665	1.690898
C	3.430832	-3.95022	-0.41624
O	4.263107	-4.82293	-0.54533
H	0.538608	-2.73395	2.408889
C	3.769118	-2.51829	-0.26312
C	5.075327	-2.18105	0.107532
C	2.847143	-1.49622	-0.51413
C	5.442804	-0.85507	0.258576
H	5.787849	-2.97587	0.301079
C	3.21552	-0.16734	-0.39398
H	1.841297	-1.73008	-0.85564
C	4.515609	0.164178	0.007552
H	6.441884	-0.59666	0.592056
H	2.514532	0.625069	-0.63241
C	-2.01489	-1.67257	0.832253
C	-2.4255	-1.52009	-0.51331
C	-2.71116	-0.95559	1.835314
C	-3.49381	-0.7169	-0.83666
H	-1.88052	-2.00985	-1.31299
C	-3.79586	-0.1763	1.517346
H	-2.38303	-1.02699	2.866328
C	-4.21186	-0.042	0.173753
H	-3.81198	-0.62946	-1.86815
H	-4.30624	0.384066	2.291237
C	6.039623	2.104599	-0.36934
C	4.149263	2.486614	0.834662
C	7.042952	1.551068	-1.16257
C	6.043124	3.46708	-0.01894

C	2.965826	2.364729	1.561052
C	4.839806	3.710361	0.751768
C	8.073559	2.387963	-1.57145
H	7.023244	0.509668	-1.46529
C	7.087793	4.288842	-0.44127
C	2.467253	3.505839	2.1775
H	2.456303	1.412286	1.662318
C	4.320591	4.84254	1.378272
C	8.103534	3.74147	-1.21167
H	8.869982	1.981232	-2.18675
H	7.101221	5.341389	-0.17461
C	3.130136	4.735829	2.083806
H	1.547555	3.437888	2.750735
H	4.845958	5.791079	1.320554
H	8.924999	4.366496	-1.54605
H	2.71434	5.608359	2.577027
C	-5.40052	1.609982	-1.2602
C	-6.50452	0.825743	0.589268
C	-4.44654	1.951823	-2.21537
C	-6.66061	2.228373	-1.22907
C	-6.91913	0.1034	1.70525
C	-7.36276	1.725772	-0.06192
C	-4.8061	2.886889	-3.18013
H	-3.44522	1.53695	-2.20985
C	-7.00173	3.164042	-2.20227
C	-8.19878	0.347684	2.192186
H	-6.292	-0.64222	2.180222
C	-8.64174	1.955116	0.438906
C	-6.07231	3.480185	-3.18429
H	-4.08145	3.166016	-3.93812
H	-7.97416	3.646033	-2.18443
C	-9.04993	1.270099	1.575643
H	-8.5421	-0.19735	3.065473
H	-9.31046	2.650417	-0.0587
H	-6.32205	4.205871	-3.95096
H	-10.0418	1.438823	1.981102
N	4.881826	1.507678	0.150979
N	-5.29555	0.745402	-0.14387
H	-1.77302	-5.98663	-1.5397
H	0.314159	-6.68722	-2.34368

Table S98. Coordinates for the optimized geometry of *anti*-E-**3d** (T_1).

Atom	*x*	*y*	*z*
C	-0.1703	5.359471	1.470521
C	-1.38376	4.75837	1.047653
C	-1.55302	3.625264	0.30022
C	1.105871	4.953992	1.181289
C	-0.61091	2.743925	-0.31551
C	1.581725	3.870896	0.398629
C	0.804516	2.977035	-0.38949
H	-2.58834	3.36775	0.10113
H	1.906882	5.559701	1.596277
C	-1.12794	1.559145	-1.0086
O	-0.44569	0.947502	-1.86053
O	1.505502	2.273112	-1.25337
C	3.064298	3.788332	0.329716
O	3.769141	4.773871	0.47998
C	3.772187	2.460111	0.198809
C	5.005089	2.440123	-0.45643
C	3.290459	1.297121	0.80642
C	5.734502	1.266476	-0.54823
H	5.376564	3.355095	-0.9043
C	4.008111	0.11622	0.732715
H	2.351796	1.319885	1.350803
C	5.226631	0.104838	0.043553
H	6.663847	1.233252	-1.10753
H	3.656533	-0.7799	1.233564
C	-2.4755	1.00433	-0.70297
C	-2.98635	0.931456	0.597848
C	-3.20832	0.43783	-1.75145
C	-4.21359	0.333123	0.84213
H	-2.42216	1.340554	1.430704
C	-4.4354	-0.15964	-1.5137
H	-2.79795	0.45881	-2.75546
C	-4.94973	-0.21252	-0.21379
H	-4.61635	0.297863	1.848659
H	-4.99434	-0.60996	-2.32704
C	7.299073	-1.26254	0.250552
C	5.429575	-2.3174	-0.48718
C	8.18968	-0.32363	0.77625
C	7.660745	-2.60899	0.018545
C	4.144461	-2.59272	-0.95972
C	6.45499	-3.28914	-0.45757

C	9.489479	-0.75583	1.023076
H	7.882717	0.692256	0.997337
C	8.950359	-3.02077	0.278603
C	3.886886	-3.90023	-1.3619
H	3.384469	-1.8226	-1.02779
C	6.183355	-4.57651	-0.86914
C	9.866253	-2.07707	0.774083
H	10.21475	-0.05745	1.425086
H	9.257041	-4.04852	0.115459
C	4.883898	-4.87633	-1.31231
H	2.900483	-4.15829	-1.73043
H	6.950772	-5.3433	-0.86335
H	10.88496	-2.38728	0.980955
H	4.656264	-5.88584	-1.6377
C	-6.46031	-1.77776	1.00989
C	-7.38151	-0.55033	-0.66305
C	-5.59052	-2.39076	1.910592
C	-7.82161	-2.13115	0.942697
C	-7.62507	0.363303	-1.68745
C	-8.4101	-1.34524	-0.12305
C	-6.11984	-3.3476	2.767264
H	-4.53481	-2.14246	1.939166
C	-8.3306	-3.09504	1.812639
C	-8.92027	0.444064	-2.18252
H	-6.83973	0.999425	-2.0817
C	-9.70338	-1.24672	-0.63606
C	-7.47636	-3.69534	2.726703
H	-5.4644	-3.83788	3.480706
H	-9.37931	-3.37381	1.7699
C	-9.95084	-0.35465	-1.66948
H	-9.13645	1.145846	-2.98217
H	-10.5041	-1.85592	-0.22733
H	-7.85835	-4.44539	3.411641
H	-10.9514	-0.26738	-2.08038
N	5.952629	-1.10219	-0.05713
N	-6.19442	-0.8154	0.029851
H	-2.29602	5.259334	1.358924
H	-0.26434	6.246465	2.090075
H	0.860307	1.641925	-1.72785

Table S99. Coordinates for the optimized geometry of *anti*-K-**3d** (T_1).

Atom	*x*	*y*	*z*
C	0.612577	-1.13446	-2.37343
C	-0.78425	-1.3009	-2.32707
C	-1.60618	-1.43115	-1.22849
C	1.498942	-1.13	-1.31894
C	-1.32028	-1.49918	0.162973
C	1.262646	-1.33564	0.056308
C	0.020259	-1.69282	0.719522
H	-2.65933	-1.53475	-1.4686
H	2.543738	-1.00058	-1.58549
C	-2.42703	-1.49107	1.073733
O	-2.32075	-1.91471	2.303528
O	0.128663	-2.13579	1.891252
C	2.363884	-1.34802	0.981501
O	2.192379	-1.75531	2.159917
H	-1.39498	-2.22539	2.473726
C	3.739346	-0.89858	0.683917
C	4.820758	-1.66182	1.14533
C	3.975278	0.320916	0.032601
C	6.116658	-1.23804	0.914282
H	4.641523	-2.58651	1.683527
C	5.272015	0.74535	-0.19249
H	3.145315	0.941501	-0.29124
C	6.358125	-0.03225	0.239635
H	6.953179	-1.82006	1.283885
H	5.456502	1.671525	-0.7248
C	-3.75692	-0.99986	0.743832
C	-3.96108	0.103425	-0.10469
C	-4.87201	-1.59044	1.366314
C	-5.23511	0.576778	-0.35047
H	-3.11486	0.612145	-0.55513
C	-6.14682	-1.12845	1.110241
H	-4.72762	-2.41648	2.053867
C	-6.34726	-0.04057	0.244169
H	-5.38351	1.414422	-1.02197
H	-6.99852	-1.57936	1.606354
C	8.710717	-0.40578	-0.4929
C	8.155085	1.694873	0.199094
C	8.698512	-1.73877	-0.89801
C	9.863367	0.390042	-0.61512
C	7.5203	2.813078	0.735862

C	9.511348	1.724774	-0.17043
C	9.882477	-2.27224	-1.39329
H	7.801777	-2.34677	-0.84852
C	11.04013	-0.16492	-1.11528
C	8.264814	3.980619	0.855407
H	6.4896	2.782673	1.072283
C	10.23991	2.905975	-0.04094
C	11.04531	-1.49972	-1.49537
H	9.900563	-3.31002	-1.71092
H	11.93558	0.441475	-1.21157
C	9.607958	4.033538	0.464632
H	7.793066	4.866484	1.268766
H	11.28768	2.93873	-0.32361
H	11.95391	-1.94745	-1.88407
H	10.16089	4.960946	0.570805
C	-8.03271	1.773304	-0.045
C	-8.75355	-0.37629	-0.30438
C	-7.30477	2.924533	0.248371
C	-9.40311	1.828582	-0.35302
C	-8.8454	-1.7581	-0.45686
C	-9.86001	0.462586	-0.52331
C	-7.97104	4.142571	0.181196
H	-6.26141	2.886449	0.541609
C	-10.0524	3.0603	-0.41394
C	-10.0859	-2.28815	-0.7919
H	-7.98628	-2.40896	-0.33751
C	-11.0947	-0.08935	-0.85997
C	-9.32775	4.21539	-0.15486
H	-7.42548	5.054366	0.402879
H	-11.1105	3.112154	-0.65125
C	-11.2032	-1.46742	-0.98423
H	-10.1848	-3.36246	-0.91103
H	-11.9547	0.551209	-1.02935
H	-9.81799	5.182241	-0.19916
H	-12.1578	-1.91332	-1.24301
N	7.663424	0.391919	0.00305
N	-7.63196	0.42408	-0.01399
H	-1.28927	-1.31828	-3.28828
H	1.046835	-1.00757	-3.36028

Table S100. Coordinates for the optimized geometry of *anti*-E-**3d** (S_1).

Atom	*x*	*y*	*z*
C	-0.16107	5.34188	1.55717
C	-1.35789	4.71575	1.12591
C	-1.49953	3.58010	0.37430
C	1.12462	4.96684	1.27212
C	-0.53958	2.71987	-0.23875
C	1.62186	3.89692	0.48319
C	0.87249	2.98535	-0.30250
H	-2.52820	3.29981	0.17451
H	1.91032	5.58753	1.69207
C	-1.02764	1.52575	-0.93350
O	-0.31846	0.91270	-1.76177
O	1.59295	2.29358	-1.15879
C	3.10610	3.82896	0.42860
O	3.80995	4.80580	0.60263
C	3.80814	2.49674	0.28858
C	4.91630	2.40530	-0.55618
C	3.42539	1.39968	1.06127
C	5.60925	1.21432	-0.66994
H	5.21456	3.27045	-1.13755
C	4.10503	0.19902	0.95740
H	2.58890	1.48504	1.74678
C	5.19105	0.10806	0.08028
H	6.43539	1.11775	-1.36652
H	3.83215	-0.64997	1.57518
C	-2.38407	0.96772	-0.66462
C	-2.91216	0.85984	0.62545
C	-3.10914	0.44885	-1.74099
C	-4.15361	0.27536	0.83326
H	-2.35341	1.23393	1.47841
C	-4.34877	-0.13814	-1.53870
H	-2.68649	0.49848	-2.73893
C	-4.88271	-0.22208	-0.24962
H	-4.57134	0.21269	1.83236
H	-4.90470	-0.55106	-2.37359
C	7.25713	-1.28122	0.03095
C	5.27847	-2.34923	-0.26413
C	8.23607	-0.32605	0.30875
C	7.56059	-2.64893	-0.13879
C	3.92765	-2.64217	-0.48098
C	6.28719	-3.33839	-0.32928

C	9.55482	-0.76759	0.36006
H	7.98580	0.71162	0.49398
C	8.87387	-3.06990	-0.07331
C	3.59672	-3.97308	-0.71132
H	3.17116	-1.86742	-0.49078
C	5.93894	-4.65067	-0.56990
C	9.87190	-2.11307	0.16656
H	10.34495	-0.05484	0.56603
H	9.13473	-4.11578	-0.19415
C	4.58030	-4.96288	-0.74872
H	2.56021	-4.24091	-0.87876
H	6.69189	-5.42891	-0.63204
H	10.90777	-2.42915	0.21809
H	4.29435	-5.99153	-0.93729
C	-6.44292	-1.80417	0.88948
C	-7.32141	-0.46888	-0.72145
C	-5.59502	-2.48326	1.76320
C	-7.81506	-2.10947	0.80651
C	-7.53266	0.49784	-1.70335
C	-8.37630	-1.25486	-0.22003
C	-6.15617	-3.46016	2.57566
H	-4.53224	-2.26888	1.80468
C	-8.35595	-3.09503	1.63230
C	-8.82358	0.64416	-2.19431
H	-6.72542	1.12361	-2.06908
C	-9.66476	-1.09017	-0.72889
C	-7.52302	-3.76286	2.51838
H	-5.51833	-4.00221	3.26729
H	-9.41316	-3.33723	1.57748
C	-9.88099	-0.14294	-1.71906
H	-9.01505	1.38847	-2.96119
H	-10.48560	-1.69200	-0.35023
H	-7.92985	-4.53045	3.16868
H	-10.87728	-0.00389	-2.12597
N	5.87451	-1.11587	-0.04637
N	-6.14500	-0.80642	-0.04351
H	-2.28205	5.19466	1.43413
H	-0.27819	6.22394	2.17852
H	0.96779	1.63694	-1.62590

Table S101. Coordinates for the optimized geometry of *anti*-K-**3d** (S_1).

Atom	*x*	*y*	*z*
C	0.14646	-5.80522	-1.10643
C	-1.01769	-5.16458	-0.65609
C	-1.15578	-3.93625	-0.04619
C	1.45419	-5.35168	-1.00097
C	-0.17795	-3.00650	0.41096
C	1.95787	-4.22694	-0.35272
C	1.26023	-3.31391	0.52705
H	-2.18091	-3.64815	0.16468
H	2.21729	-5.96438	-1.47491
C	-0.59344	-1.74070	0.88193
O	0.20384	-0.97267	1.59532
O	1.94954	-2.68303	1.36496
C	3.42285	-4.00184	-0.49149
O	4.21706	-4.88535	-0.74492
H	1.03825	-1.52138	1.76297
C	3.92917	-2.57920	-0.41722
C	5.08012	-2.29822	0.32355
C	3.27681	-1.55737	-1.11125
C	5.52398	-0.99571	0.44993
H	5.59089	-3.10516	0.83678
C	3.70251	-0.24907	-0.99111
H	2.41699	-1.78670	-1.73182
C	4.81174	0.03268	-0.18397
H	6.36765	-0.75813	1.08914
H	3.21026	0.54968	-1.53540
C	-1.90976	-1.13660	0.61854
C	-2.54839	-1.25199	-0.62223
C	-2.51703	-0.37366	1.62385
C	-3.77896	-0.65158	-0.84216
H	-2.07967	-1.81209	-1.42540
C	-3.74183	0.23575	1.40383
H	-2.02043	-0.26006	2.58137
C	-4.38681	0.09450	0.17118
H	-4.28123	-0.76476	-1.79674
H	-4.20433	0.83472	2.18085
C	6.49447	1.87236	-0.11409
C	4.33432	2.40349	0.33987
C	7.65578	1.20542	-0.50158
C	6.46713	3.25836	0.14255
C	2.96889	2.34240	0.64368

C	5.07812	3.60287	0.43527
C	8.82391	1.95910	-0.57388
H	7.65391	0.15158	-0.75329
C	7.63412	3.99251	0.05432
C	2.34292	3.53645	0.98565
H	2.42197	1.40771	0.63882
C	4.43980	4.77269	0.78806
C	8.81737	3.32677	-0.29514
H	9.74955	1.47535	-0.86375
H	7.63995	5.06110	0.24045
C	3.05918	4.73234	1.04970
H	1.28544	3.53151	1.22114
H	4.98608	5.70568	0.87517
H	9.74165	3.88924	-0.36567
H	2.54659	5.64676	1.32630
C	-5.97498	1.50624	-1.13980
C	-6.75291	0.59268	0.78819
C	-5.18712	1.93243	-2.20784
C	-7.31451	1.91664	-1.00139
C	-6.91355	-0.13713	1.96482
C	-7.81167	1.33098	0.22710
C	-5.77826	2.75970	-3.15432
H	-4.14619	1.64016	-2.29864
C	-7.88662	2.74813	-1.96431
C	-8.15483	-0.08992	2.58633
H	-6.10759	-0.73246	2.38081
C	-9.04971	1.36377	0.86942
C	-7.11549	3.16180	-3.04087
H	-5.18776	3.10265	-3.99847
H	-8.91967	3.06880	-1.86831
C	-9.21335	0.65593	2.05109
H	-8.30626	-0.64812	3.50510
H	-9.87312	1.93088	0.44555
H	-7.54760	3.80646	-3.79928
H	-10.16991	0.67214	2.56308
N	5.19544	1.36907	0.00478
N	-5.63448	0.70158	-0.04695
H	-1.94775	-5.69470	-0.84054
H	0.00789	-6.75194	-1.61860

Table S102. Coordinates for the optimized geometry of E-**4a** (S_0).

Atom	*x*	*y*	*z*
C	1.535726	0.708521	-0.023693
C	1.549548	-0.715713	0.035842
C	0.389567	-1.542051	0.072602
C	0.359520	1.525981	-0.029101
C	-0.958252	-1.290037	0.023901
C	-0.985219	1.279836	-0.025810
C	-1.624538	-0.008849	-0.061501
H	0.630130	-2.603473	0.135759
H	0.597157	2.590313	-0.034170
C	-1.812059	-2.511799	0.047305
H	-1.281181	-3.471863	0.152395
O	-3.025318	-2.494828	-0.044006
O	-2.906752	0.013070	-0.172648
H	-3.282321	-0.919079	-0.166688
C	-1.835267	2.539745	0.027604
H	-1.228890	3.462845	-0.079260
O	-3.020044	2.584552	0.194049
C	2.766487	1.391348	-0.061306
C	2.791527	-1.385259	0.058452
C	3.969563	0.709452	-0.045134
C	3.983188	-0.688532	0.016459
H	2.767874	2.476044	-0.103281
H	4.902915	1.261196	-0.077204
H	4.925900	-1.224430	0.032447
H	2.806588	-2.469771	0.107120

Table S103. Coordinates for the optimized geometry of K-**4a** (S_0).

Atom	*x*	*y*	*z*
C	-1.542231	-0.705962	-0.113182
C	-1.532618	0.712743	0.044218
C	-0.356001	1.562243	0.014829
C	-0.391006	-1.528193	-0.232560
C	0.973885	1.319708	0.035833
C	0.960423	-1.266745	-0.087666
C	1.640220	0.021890	0.209011
H	-0.612063	2.621042	-0.040894
H	-0.628722	-2.574162	-0.425562
C	1.854507	2.536638	-0.143435
H	1.308992	3.495568	-0.024207
O	3.015370	2.501348	-0.441110
O	2.817594	-0.017886	0.568408
C	1.784873	-2.422443	-0.158084
H	1.384953	-3.395661	-0.438806
O	3.066719	-2.437691	0.102802
C	-2.762030	1.368354	0.169993
C	-2.782250	-1.386271	-0.149596

C	-3.979487	-0.712881	-0.004677
C	-3.965390	0.672551	0.160863
H	-2.776649	2.448439	0.276534
H	-4.897968	1.215932	0.271553
H	-4.917999	-1.255246	-0.024951
H	-2.788552	-2.463948	-0.283761
H	3.313178	-1.487908	0.376027

Table S104. Coordinates for the optimized geometry of E-**4a** (T_1).

Atom	*x*	*y*	*z*
C	-1.550603	0.735622	-0.000108
C	-1.546276	-0.712491	0.000113
C	-0.405844	-1.522024	0.000104
C	-0.431353	1.567003	-0.000063
C	0.968115	-1.260062	-0.000044
C	0.969240	1.304495	0.000051
C	1.607914	0.008615	-0.000028
H	-0.632114	-2.588057	0.000233
H	-0.670379	2.627917	-0.000153
C	1.843422	-2.442134	-0.000119
H	1.349962	-3.425988	-0.000128
O	3.068250	-2.385307	-0.000209
O	2.903331	0.067338	0.000010
H	3.292765	-0.881959	-0.000080
C	1.833940	2.403282	0.000134
H	1.438961	3.428383	0.000114
O	3.108286	2.399434	0.000195
C	-2.797833	-1.396888	0.000276
C	-2.821514	1.390553	-0.000297
C	-3.997433	0.689951	-0.000165
C	-3.990581	-0.725185	0.000167
H	-2.792704	-2.482720	0.000473
H	-4.926538	-1.272508	0.000320
H	-4.942314	1.222978	-0.000316
H	-2.843747	2.475800	-0.000548

Table S105. Coordinates for the optimized geometry of K-**4a** (T_1).

Atom	*x*	*y*	*z*
C	-1.583060	0.723859	-0.000012
C	-1.519604	-0.726884	-0.000042
C	-0.369944	-1.513828	-0.000046
C	-0.488745	1.585425	-0.000090
C	1.025876	-1.231923	-0.000033
C	0.895900	1.345003	-0.000073
C	1.631287	0.098241	0.000017

H	-0.580085	-2.581607	-0.000054
H	-0.748909	2.642431	-0.000147
C	1.871661	-2.343145	-0.000070
H	1.448097	-3.344778	-0.000319
O	3.167126	-2.359343	0.000060
O	2.880834	0.188179	0.000150
H	3.523795	-1.441375	0.000322
C	1.796840	2.439157	-0.000079
H	1.430977	3.474288	-0.000114
O	3.034242	2.302989	0.000010
C	-2.760670	-1.440898	-0.000039
C	-2.870495	1.343944	0.000053
C	-4.027363	0.614419	0.000102
C	-3.969269	-0.800827	0.000036
H	-2.730308	-2.526108	-0.000096
H	-4.887601	-1.377993	0.000036
H	-4.988490	1.116642	0.000189
H	-2.919577	2.428646	0.000083

Table S106. Coordinates for the optimized geometry of E-**4a** (S_1).

Atom	*x*	*y*	*z*
C	1.548311	0.730831	0.000003
C	1.542978	-0.716207	-0.000063
C	0.406297	-1.524496	-0.000063
C	0.427988	1.559645	0.000063
C	-0.970836	-1.263209	-0.000024
C	-0.962216	1.288168	0.000050
C	-1.613797	0.006144	0.000000
H	0.632178	-2.590352	-0.000055
H	0.669007	2.619401	0.000110
C	-1.848657	-2.437586	0.000151
H	-1.361221	-3.423972	-0.000419
O	-3.075771	-2.376890	0.000371
O	-2.908001	0.061115	-0.000257
H	-3.294063	-0.893234	-0.000589
C	-1.839937	2.402263	0.000055
H	-1.402356	3.421381	0.000064
O	-3.077081	2.430969	0.000052
C	2.800621	-1.399791	-0.000100
C	2.819164	1.389607	0.000014
C	3.993179	0.691793	-0.000032
C	3.987823	-0.726838	-0.000088
H	2.795803	-2.485485	-0.000140
H	4.925542	-1.271088	-0.000118
H	4.937903	1.224994	-0.000022
H	2.838529	2.474846	0.000060

Table S107. Coordinates for the optimized geometry of K-**4a** (S_1).

Atom	*x*	*y*	*z*
C	1.579166	0.724502	0.000005
C	1.520902	-0.718457	0.000021
C	0.369451	-1.507853	0.000040
C	0.476040	1.585721	0.000013
C	-1.015378	-1.238693	0.000048
C	-0.896278	1.333572	0.000037
C	-1.636508	0.095919	0.000070
H	0.585373	-2.575003	0.000032
H	0.735933	2.642057	0.000000
C	-1.863565	-2.354537	0.000039
H	-1.436803	-3.354609	0.000074
O	-3.158849	-2.370239	-0.000035
O	-2.880129	0.160096	-0.000041
H	-3.513705	-1.451106	-0.000127
C	-1.807532	2.448000	0.000024
H	-1.406041	3.476329	-0.000020
O	-3.025077	2.339651	-0.000033
C	2.761590	-1.437080	-0.000001
C	2.863863	1.346650	-0.000030
C	4.019805	0.616557	-0.000051
C	3.968309	-0.799363	-0.000035
H	2.727509	-2.522216	0.000007
H	4.888196	-1.373560	-0.000051
H	4.979857	1.121294	-0.000081
H	2.912937	2.431113	-0.000044

Table S108. Coordinates for the optimized geometry of *syn*-E-**4b** (S_0).

Atom	*x*	*y*	*z*
C	0.500916	2.307211	0.206944
C	-0.84999	2.144712	-0.21511
C	-1.53847	0.900681	-0.34424
C	1.398397	1.238409	0.535627
C	-1.14624	-0.413	-0.27207
C	1.300004	-0.1149	0.4262
C	0.207815	-0.87414	-0.10148
H	-2.59206	1.026785	-0.58047
H	2.361754	1.581849	0.910913
C	-2.19634	-1.47339	-0.51637
O	-1.84914	-2.54767	-1.00988
O	0.512884	-2.09685	-0.39401
C	2.457649	-0.94844	0.992823
O	2.223744	-1.56947	2.006117
H	-0.30432	-2.56097	-0.76706
C	3.774401	-0.90255	0.325198

C	3.950387	-0.31871	-0.93479
C	4.863163	-1.49319	0.979372
C	5.204732	-0.32129	-1.5322
H	3.108981	0.12441	-1.45998
C	6.114919	-1.48695	0.382567
H	4.707081	-1.95026	1.950887
C	6.285562	-0.90117	-0.87187
H	5.340056	0.123796	-2.51242
H	6.959527	-1.94058	0.890437
C	-3.61027	-1.23503	-0.16761
C	-3.9894	-0.46064	0.939277
C	-4.58762	-1.89103	-0.92859
C	-5.33292	-0.33486	1.267213
H	-3.23882	0.005161	1.57125
C	-5.9289	-1.74176	-0.60831
H	-4.28278	-2.50812	-1.76717
C	-6.30116	-0.96498	0.488136
H	-5.62468	0.245816	2.135828
H	-6.68563	-2.23805	-1.20638
C	1.01574	3.613755	0.331028
C	-1.61618	3.299859	-0.49656
C	0.247415	4.72174	0.03683
H	0.671775	5.715211	0.135357
C	-1.08052	4.564326	-0.38277
H	-1.6868	5.434122	-0.61101
H	2.041198	3.743977	0.662452
H	-2.6486	3.180278	-0.81072
H	7.265641	-0.9005	-1.33848
H	-7.35077	-0.8591	0.743135

Table S109. Coordinates for the optimized geometry of *anti*-E-**4b** (S_0).

Atom	*x*	*y*	*z*
C	-0.11071	2.93711	0.12544
C	-1.39349	2.334626	-0.02065
C	-1.63561	0.958271	-0.3133
C	1.142966	2.259466	-0.01542
C	-0.82384	-0.08734	-0.67562
C	1.480571	1.001276	-0.41956
C	0.595179	-0.00275	-0.92257
H	-2.69098	0.699999	-0.27919
H	2.007756	2.885304	0.197763
C	-1.48165	-1.41566	-0.97181
O	-0.96112	-2.16394	-1.80116

O	1.178268	-0.92517	-1.61754
H	0.48563	-1.5941	-1.92804
C	-2.7204	-1.81992	-0.27866
C	-3.59772	-2.67407	-0.9609
C	-2.99327	-1.45321	1.047878
C	-4.75126	-3.12396	-0.33553
H	-3.36622	-2.97104	-1.9783
C	-4.13872	-1.9258	1.674679
H	-2.29513	-0.83553	1.605428
C	-5.02145	-2.75068	0.980375
H	-5.43717	-3.77279	-0.86953
H	-4.33951	-1.65832	2.70666
C	2.989736	0.727729	-0.49952
O	3.681705	1.565914	-1.0367
C	3.535798	-0.48971	0.142701
C	2.805681	-1.23144	1.07771
C	4.845802	-0.8655	-0.17284
C	3.376576	-2.34177	1.68529
H	1.798536	-0.9315	1.357731
C	5.409299	-1.9834	0.426293
H	5.404223	-0.2744	-0.89088
C	4.675979	-2.72094	1.354173
H	2.813534	-2.90964	2.418678
H	6.421878	-2.27985	0.173601
H	-5.91974	-3.11233	1.470451
H	5.120084	-3.59225	1.825016
C	-0.04216	4.307183	0.450651
C	-2.54402	3.133862	0.166663
C	-2.44591	4.475657	0.468152
H	-3.34373	5.07003	0.598336
C	-1.18364	5.066752	0.61128
H	-1.10159	6.121132	0.852568
H	0.932719	4.769498	0.568908
H	-3.52352	2.676602	0.064545

Table S110. Coordinates for the optimized geometry of *syn*-K-**4b** (S_0).

Atom	*x*	*y*	*z*
C	0.584562	2.243078	0.15197
C	-0.79464	2.179577	-0.19112
C	-1.57101	0.983166	-0.3189
C	1.444315	1.105224	0.413764
C	-1.22334	-0.3427	-0.38048
C	1.286266	-0.21629	0.189406

C	0.15315	-0.87469	-0.45956
H	-2.63261	1.179387	-0.44586
H	2.408707	1.382646	0.836895
C	-2.29621	-1.3226	-0.56582
O	-2.03507	-2.4342	-1.14413
O	0.3493	-1.95151	-1.04112
C	2.34271	-1.18632	0.711919
O	1.958567	-2.03431	1.489728
C	3.759053	-1.01914	0.31283
C	4.137993	-0.25805	-0.79893
C	4.733421	-1.68963	1.062492
C	5.478807	-0.16321	-1.1519
H	3.387114	0.24144	-1.40409
C	6.071658	-1.58311	0.713604
H	4.421641	-2.28749	1.912491
C	6.44435	-0.81993	-0.3927
H	5.770899	0.417256	-2.02095
H	6.826778	-2.09731	1.298983
C	-3.66505	-1.13349	-0.11901
C	-3.9654	-0.38437	1.034054
C	-4.69582	-1.78329	-0.82236
C	-5.28055	-0.27293	1.458529
H	-3.16965	0.057484	1.624921
C	-6.00779	-1.64697	-0.40085
H	-4.45785	-2.37193	-1.70166
C	-6.29951	-0.89324	0.736922
H	-5.51156	0.284318	2.359755
H	-6.80558	-2.13218	-0.95231
C	1.176852	3.50815	0.287908
C	-1.50614	3.384948	-0.38524
C	0.462073	4.674617	0.069933
H	0.955812	5.635179	0.173225
C	-0.89121	4.615346	-0.27112
H	-1.4567	5.525818	-0.43574
H	2.224379	3.569102	0.566257
H	-2.56193	3.336364	-0.6356
H	7.491705	-0.74176	-0.66715
H	-7.32805	-0.79942	1.070372
H	-1.01672	-2.46384	-1.32741

Table S111. Coordinates for the optimized geometry of *anti*-K-**4b** (S_0).

Atom	*x*	*y*	*z*
C	-0.22413	3.00274	0.14123
C	-1.47478	2.342607	-0.02141
C	-1.65635	0.946682	-0.27692
C	1.077901	2.374812	0.075341
C	-0.78006	-0.05131	-0.6257
C	1.478547	1.150207	-0.33315
C	0.649264	0.127404	-0.95816
H	-2.69941	0.642946	-0.24338
H	1.898618	3.028431	0.366067
C	-1.32885	-1.38557	-0.87691
O	-0.71549	-2.1725	-1.67829
O	1.183309	-0.66156	-1.75476
C	-2.53647	-1.90738	-0.25973
C	-3.28371	-2.87073	-0.9616
C	-2.92559	-1.52637	1.037999
C	-4.42361	-3.40904	-0.38807
H	-2.97262	-3.1715	-1.95612
C	-4.05684	-2.08773	1.610134
H	-2.3166	-0.83747	1.614465
C	-4.80959	-3.01821	0.894742
H	-5.01111	-4.13784	-0.93553
H	-4.34601	-1.81149	2.618211
C	2.984715	0.889126	-0.30768
O	3.730009	1.783594	-0.64711
C	3.476305	-0.41838	0.196048
C	2.694819	-1.24293	1.011592
C	4.785815	-0.79404	-0.11713
C	3.213452	-2.43442	1.501774
H	1.68861	-0.94306	1.296509
C	5.297172	-1.99319	0.360567
H	5.386264	-0.13794	-0.73828
C	4.512435	-2.81308	1.169135
H	2.610728	-3.06548	2.147007
H	6.310075	-2.28819	0.107088
H	-5.69752	-3.45094	1.344397
H	4.916113	-3.74723	1.546827
C	-0.23042	4.377552	0.423946
C	-2.66488	3.093368	0.106907
C	-2.64041	4.449757	0.36246
H	-3.56743	5.006219	0.445105
C	-1.41166	5.095024	0.521412
H	-1.37867	6.159408	0.728883

H	0.717522	4.888105	0.561237
H	-3.61961	2.587993	-0.00626
H	0.159954	-1.69962	-1.97597

Table S112. Coordinates for the optimized geometry of *syn*-E-**4b** (T_1).

Atom	*x*	*y*	*z*
C	0.688523	1.973409	0.06984
C	-0.70827	1.959021	-0.29344
C	-1.51503	0.815079	-0.38253
C	1.509178	0.857079	0.208367
C	-1.28397	-0.55261	-0.22189
C	1.2853	-0.5339	-0.01653
C	0.005923	-1.18319	-0.17665
H	-2.55174	1.045748	-0.61056
H	2.532134	1.089654	0.489545
C	-2.46233	-1.46056	-0.24043
O	-2.30803	-2.6741	-0.46881
O	0.069226	-2.46872	-0.28085
C	2.408265	-1.38454	-0.02721
O	2.406461	-2.65142	-0.13933
H	-0.90743	-2.82918	-0.42963
C	3.841237	-0.96338	0.050397
C	4.388575	-0.15178	-0.95411
C	4.637022	-1.46163	1.090276
C	5.723868	0.210289	-0.87267
H	3.769187	0.191433	-1.77688
C	5.972939	-1.08569	1.160526
H	4.206221	-2.12075	1.836331
C	6.513451	-0.25457	0.182958
H	6.156349	0.848938	-1.6355
H	6.593446	-1.45136	1.971351
C	-3.83214	-0.96972	0.028528
C	-4.10291	-0.013	1.01687
C	-4.8888	-1.56638	-0.67071
C	-5.41574	0.352491	1.286146
H	-3.29555	0.415262	1.604061
C	-6.19626	-1.1788	-0.41343
H	-4.67336	-2.32659	-1.41411
C	-6.46001	-0.2207	0.563933
H	-5.62483	1.077762	2.065471
H	-7.01204	-1.63058	-0.96771
C	1.305005	3.249551	0.270824
C	-1.37194	3.204441	-0.49971
C	0.624418	4.419446	0.076319

H	1.127946	5.3686	0.227514
C	-0.73337	4.402545	-0.32726
H	-1.26432	5.334514	-0.48612
H	2.349061	3.274968	0.567745
H	-2.41968	3.188215	-0.78567
H	7.560038	0.027557	0.234345
H	-7.4843	0.072326	0.771079

Table S113. Coordinates for the optimized geometry of *syn*-K-**4b** (T_1).

Atom	*x*	*y*	*z*
C	0.675578	2.060614	-0.2971
C	-0.76184	1.994748	-0.44491
C	-1.55065	0.8446	-0.44479
C	1.532562	0.966027	-0.19676
C	-1.27752	-0.54598	-0.3597
C	1.304567	-0.41709	-0.25452
C	0.060392	-1.12916	-0.48674
H	-2.60837	1.057822	-0.55981
H	2.582605	1.230365	-0.10626
C	-2.38355	-1.43127	-0.23977
O	-2.29656	-2.7178	-0.45791
O	0.166574	-2.34969	-0.75474
C	2.410823	-1.32734	-0.19672
O	2.243312	-2.54868	-0.44738
H	-1.37691	-2.95386	-0.73017
C	3.806052	-0.96234	0.158886
C	4.851854	-1.44139	-0.63762
C	4.072567	-0.21587	1.313571
C	6.163005	-1.13184	-0.29972
H	4.634791	-2.03673	-1.51822
C	5.388872	0.073043	1.64916
H	3.260392	0.110816	1.956107
C	6.430904	-0.37733	0.840286
H	6.976834	-1.48721	-0.92254
H	5.602249	0.64307	2.547175
C	-3.73485	-1.01691	0.160843
C	-3.94335	-0.08751	1.191279
C	-4.8355	-1.63224	-0.4528
C	-5.23519	0.235357	1.583186
H	-3.09638	0.350149	1.710985
C	-6.12374	-1.28904	-0.06847
H	-4.67411	-2.36631	-1.23477
C	-6.32467	-0.35664	0.947714
H	-5.39237	0.940253	2.392702

H	-6.97318	-1.75439	-0.55688
C	1.294895	3.349568	-0.27345
C	-1.47037	3.232395	-0.58455
C	-0.83428	4.440828	-0.56345
H	-1.40532	5.357056	-0.6686
C	0.573596	4.502437	-0.40137
H	2.373549	3.3981	-0.15694
H	1.074225	5.464385	-0.38333
H	-2.5492	3.194699	-0.70259
H	7.457428	-0.14627	1.105581
H	-7.33372	-0.0987	1.252796

Table S114. Coordinates for the optimized geometry of *syn*-E-**4b** (S_1).

Atom	*x*	*y*	*z*
C	0.72238	1.954451	-0.14073
C	-0.70078	1.959033	-0.36857
C	-1.52287	0.82832	-0.40865
C	1.529387	0.823145	-0.02852
C	-1.29771	-0.54915	-0.31332
C	1.266252	-0.56379	-0.14911
C	-0.0179	-1.19384	-0.33557
H	-2.57097	1.073928	-0.55248
H	2.575909	1.055332	0.141613
C	-2.4907	-1.44087	-0.31336
O	-2.37634	-2.63801	-0.62208
O	0.035594	-2.47429	-0.5087
C	2.388326	-1.43237	-0.08054
O	2.477872	-2.66813	-0.15671
H	-0.92685	-2.81999	-0.64623
C	3.816408	-1.00641	0.127411
C	4.633193	-0.77355	-0.99517
C	4.333621	-0.94687	1.435224
C	5.932769	-0.34423	-0.79517
H	4.232634	-0.90589	-1.99411
C	5.635392	-0.51609	1.617184
H	3.706264	-1.2111	2.279291
C	6.431386	-0.21511	0.506116
H	6.568635	-0.12537	-1.64554
H	6.043161	-0.42914	2.617963
C	-3.83304	-0.9417	0.071364
C	-4.0217	-0.04392	1.130668
C	-4.94324	-1.47425	-0.59461
C	-5.3072	0.327269	1.504635
H	-3.16977	0.336214	1.687279

C	-6.22402	-1.08119	-0.23174
H	-4.7902	-2.19073	-1.39475
C	-6.40649	-0.18163	0.816945
H	-5.45165	1.007468	2.337497
H	-7.08199	-1.48346	-0.75995
C	1.379478	3.222566	-0.04002
C	-1.36385	3.217877	-0.51942
C	0.702823	4.40001	-0.18241
H	1.236299	5.341711	-0.10608
C	-0.69363	4.403567	-0.43326
H	-1.22177	5.343296	-0.54861
H	2.449105	3.234193	0.144634
H	-2.43515	3.214076	-0.69801
H	7.459009	0.099304	0.655202
H	-7.40946	0.11597	1.105823

Table S115. Coordinates for the optimized geometry of *anti*-E-**4b** (S_1).

Atom	*x*	*y*	*z*
C	0.279907	2.770477	-0.085718
C	-1.064988	2.313789	-0.060521
C	-1.535010	0.954130	-0.328756
C	1.425041	1.968523	-0.469702
C	-0.922495	-0.206605	-0.682901
C	1.451443	0.627274	-0.769042
C	0.494483	-0.361493	-1.005979
H	-2.610319	0.869123	-0.210527
H	2.371572	2.496767	-0.495265
C	-1.786224	-1.428201	-0.859722
O	-1.405450	-2.335459	-1.599332
O	0.977720	-1.516670	-1.469561
H	0.202405	-2.092246	-1.711792
C	-3.091270	-1.558110	-0.163801
C	-4.113063	-2.247304	-0.828303
C	-3.295582	-1.107951	1.147209
C	-5.335993	-2.447392	-0.203632
H	-3.937376	-2.615835	-1.833420
C	-4.514385	-1.331915	1.776057
H	-2.494567	-0.613652	1.688708
C	-5.537077	-1.990562	1.097805
H	-6.131305	-2.967001	-0.727545
H	-4.664170	-0.998021	2.797544
C	2.884261	0.085380	-0.967573
O	3.335636	0.445055	-2.030556
C	3.580098	-0.709194	0.038340

C	2.983708	-0.934667	1.284091
C	4.849140	-1.224838	-0.268585
C	3.668917	-1.678443	2.231785
H	2.002652	-0.529708	1.505886
C	5.519310	-1.970636	0.687514
H	5.292261	-1.045173	-1.242558
C	4.930047	-2.194699	1.931794
H	3.221980	-1.860297	3.202632
H	6.499743	-2.377186	0.465751
H	-6.491107	-2.156605	1.588025
H	5.459581	-2.779315	2.677134
C	0.539763	4.106935	0.236550
C	-2.069841	3.225879	0.272976
C	-1.793418	4.559378	0.581950
H	-2.602619	5.235562	0.834609
C	-0.481265	4.999201	0.564777
H	-0.239708	6.028852	0.805665
H	1.566237	4.461052	0.220429
H	-3.101546	2.886546	0.290637

Table S116. Coordinates for the optimized geometry of *syn*-K-**4b** (S_1).

Atom	*x*	*y*	*z*
C	0.5807	2.238224	0.154096
C	-0.80122	2.112167	-0.25187
C	-1.55271	0.910735	-0.40867
C	1.447284	1.154553	0.412785
C	-1.23143	-0.45267	-0.34823
C	1.304571	-0.19797	0.167597
C	0.156413	-0.94382	-0.39137
H	-2.59258	1.10256	-0.65307
H	2.415671	1.433807	0.825523
C	-2.27048	-1.42626	-0.40742
O	-2.0456	-2.67254	-0.7628
O	0.421146	-2.03958	-0.89266
C	2.395642	-1.09804	0.697352
O	1.974141	-1.87278	1.54083
C	3.795723	-0.9742	0.289781
C	4.142104	-0.27332	-0.87235
C	4.78411	-1.5977	1.064227
C	5.474754	-0.18742	-1.25174
H	3.371664	0.180192	-1.49019
C	6.113154	-1.50201	0.682489
H	4.495541	-2.14358	1.956594
C	6.456461	-0.79774	-0.47247

H	5.749017	0.34459	-2.15632
H	6.88443	-1.97618	1.279846
C	-3.6698	-1.15455	-0.07796
C	-4.03173	-0.25907	0.943188
C	-4.67268	-1.86737	-0.75569
C	-5.36852	-0.06605	1.259342
H	-3.26426	0.251517	1.51635
C	-6.0073	-1.65684	-0.44401
H	-4.39555	-2.57355	-1.53045
C	-6.35776	-0.75634	0.561108
H	-5.64012	0.612272	2.061333
H	-6.77767	-2.19925	-0.98169
C	1.143093	3.536072	0.336675
C	-1.50514	3.311122	-0.462
C	0.421152	4.683371	0.110413
H	0.871635	5.659663	0.244544
C	-0.91846	4.560883	-0.29931
H	-1.5096	5.451617	-0.48611
H	2.178549	3.60455	0.657043
H	-2.5482	3.258387	-0.75767
H	7.497862	-0.72941	-0.77073
H	-7.403	-0.60084	0.808264
H	-1.09846	-2.77176	-1.02202

Table S117. Coordinates for the optimized geometry of *anti*-K-**4b** (S_1).

Atom	*x*	*y*	*z*
C	-0.07573	2.926334	0.082842
C	-1.36259	2.288942	-0.05661
C	-1.62048	0.927387	-0.35306
C	1.164313	2.289994	-0.10839
C	-0.81809	-0.18939	-0.63392
C	1.478855	0.983269	-0.44531
C	0.62969	-0.09994	-0.9422
H	-2.68314	0.711831	-0.40396
H	2.03248	2.927269	0.038288
C	-1.43269	-1.46487	-0.76881
O	-0.85791	-2.47135	-1.38875
O	1.180128	-0.9916	-1.59907
C	-2.74466	-1.80666	-0.20659
C	-3.55197	-2.72056	-0.9011
C	-3.18079	-1.30274	1.029097
C	-4.78727	-3.08763	-0.3873
H	-3.20922	-3.12668	-1.84631
C	-4.41097	-1.68711	1.543943

H	-2.54098	-0.64048	1.604296
C	-5.21925	-2.57196	0.833242
H	-5.4139	-3.78126	-0.93783
H	-4.73455	-1.30644	2.507016
C	2.957104	0.67359	-0.53665
O	3.557913	1.502157	-1.2008
C	3.571824	-0.48142	0.121935
C	2.871991	-1.20891	1.092163
C	4.892024	-0.81707	-0.20429
C	3.496237	-2.26434	1.741047
H	1.851928	-0.94292	1.355012
C	5.504751	-1.88091	0.440116
H	5.420353	-0.24323	-0.95851
C	4.807578	-2.60242	1.409664
H	2.96287	-2.82627	2.499896
H	6.52674	-2.14715	0.193226
H	-6.18243	-2.86813	1.23607
H	5.29131	-3.43343	1.912972
C	-0.01677	4.309842	0.421351
C	-2.50299	3.104845	0.129191
C	-2.4084	4.44627	0.447151
H	-3.31436	5.02806	0.58185
C	-1.15053	5.062668	0.59934
H	-1.08085	6.114786	0.85023
H	0.96017	4.7724	0.525255
H	-3.48571	2.656337	0.021426
H	0.011118	-2.17174	-1.75131

Table S118. Coordinates for the optimized geometry of *syn*-E-**4c** (S_0).

Atom	*x*	*y*	*z*
C	0.452829	2.438763	0.257828
C	-0.85932	2.233606	-0.26139
C	-1.50943	0.972197	-0.40711
C	1.344429	1.399945	0.682008
C	-1.09964	-0.33174	-0.26347
C	1.279293	0.041683	0.606754
C	0.243374	-0.75906	0.031474
H	-2.5425	1.070188	-0.73111
H	2.269998	1.775644	1.11703
C	-2.1067	-1.41939	-0.55393
O	-1.71092	-2.50882	-0.96507
O	0.594052	-1.98763	-0.18029
C	2.411114	-0.7492	1.275668
O	2.141709	-1.30855	2.313887

H	-0.1755	-2.48908	-0.5952
C	3.760192	-0.74546	0.663029
C	3.9945	-0.22625	-0.61429
C	4.812707	-1.30785	1.395092
C	5.2702	-0.26074	-1.15754
H	3.18259	0.196314	-1.19827
C	6.090357	-1.33981	0.862149
H	4.611813	-1.7156	2.379914
C	6.31933	-0.81396	-0.41588
H	5.460981	0.133508	-2.14934
H	6.912827	-1.76912	1.423289
C	-3.55509	-1.19325	-0.34057
C	-4.04061	-0.40858	0.714506
C	-4.44905	-1.8736	-1.17683
C	-5.40669	-0.29011	0.920021
H	-3.35786	0.07852	1.403995
C	-5.815	-1.73928	-0.98914
H	-4.0644	-2.50162	-1.97305
C	-6.29445	-0.94497	0.05938
H	-5.79077	0.29905	1.745129
H	-6.51365	-2.24999	-1.64222
C	0.928838	3.759115	0.384647
C	-1.62555	3.363015	-0.63279
C	0.161394	4.84114	0.002029
H	0.555693	5.846428	0.105251
C	-1.12651	4.642236	-0.51338
H	-1.7315	5.491602	-0.81125
H	1.922511	3.922876	0.789751
H	-2.62798	3.212761	-1.02195
C	7.643249	-0.84096	-0.97456
C	-7.7103	-0.80183	0.258986
N	8.708811	-0.85657	-1.4282
N	-8.8505	-0.67709	0.418711

Table S119. Coordinates for the optimized geometry of *anti*-E-**4c** (S_0).

Atom	*x*	*y*	*z*
C	-0.11236	3.344258	0.368107
C	-1.38272	2.736996	0.142575
C	-1.59467	1.410037	-0.33643
C	1.154993	2.72332	0.131087
C	-0.76107	0.435717	-0.82968
C	1.519495	1.534118	-0.43163
C	0.657918	0.575272	-1.04936
H	-2.6454	1.13146	-0.34623

H	2.006053	3.339647	0.415311
C	-1.39396	-0.85081	-1.30348
O	-0.85131	-1.49252	-2.20198
O	1.269096	-0.25031	-1.83832
H	0.599938	-0.89026	-2.23905
C	-2.64804	-1.35393	-0.69567
C	-3.51119	-2.09659	-1.51117
C	-2.93944	-1.18941	0.665549
C	-4.67363	-2.6353	-0.98407
H	-3.26511	-2.24237	-2.55735
C	-4.09032	-1.74583	1.202129
H	-2.25345	-0.66089	1.320453
C	-4.96456	-2.45833	0.37387
H	-5.35439	-3.19791	-1.61296
H	-4.31437	-1.63864	2.257497
C	3.03302	1.318848	-0.56221
O	3.69911	2.237669	-0.98483
C	3.629887	0.039909	-0.09791
C	2.951394	-0.82789	0.762977
C	4.933479	-0.25862	-0.50515
C	3.561893	-1.98969	1.208351
H	1.950812	-0.59088	1.114761
C	5.54659	-1.42521	-0.07616
H	5.453657	0.430267	-1.16168
C	4.859548	-2.29245	0.780992
H	3.047474	-2.66268	1.885152
H	6.553355	-1.66936	-0.39622
C	-0.07334	4.658219	0.876286
C	-2.55021	3.476496	0.438025
C	-2.48083	4.766712	0.920514
H	-3.3913	5.316805	1.131938
C	-1.23189	5.361794	1.141259
H	-1.17297	6.374953	1.524231
H	0.890808	5.12368	1.054735
H	-3.5201	3.01635	0.275327
C	5.487767	-3.50387	1.231712
C	-6.16831	-3.01497	0.926977
N	5.986633	-4.48344	1.5965
N	-7.1405	-3.45537	1.376375

Table S120. Coordinates for the optimized geometry of *syn*-K-**4c** (S_0).

Atom	*x*	*y*	*z*
C	0.557673	2.320366	0.19303
C	-0.79989	2.239256	-0.22904

C	-1.55496	1.036572	-0.39333
C	1.412687	1.19407	0.511108
C	-1.19805	-0.29068	-0.40901
C	1.274864	-0.13337	0.306272
C	0.180151	-0.82099	-0.37544
H	-2.60562	1.224699	-0.59879
H	2.35444	1.48814	0.972679
C	-2.24931	-1.27695	-0.65457
O	-1.94583	-2.40876	-1.16219
O	0.413053	-1.9245	-0.89281
C	2.320401	-1.07857	0.890483
O	1.92983	-1.90227	1.688173
C	3.750097	-0.92147	0.517972
C	4.151551	-0.19799	-0.60952
C	4.70599	-1.56344	1.313526
C	5.496424	-0.10634	-0.93726
H	3.415926	0.279501	-1.24914
C	6.051462	-1.46725	0.999473
H	4.377264	-2.13418	2.175309
C	6.447324	-0.73556	-0.12728
H	5.816012	0.443868	-1.81523
H	6.799131	-1.95485	1.615081
C	-3.65984	-1.07474	-0.34008
C	-4.06078	-0.32392	0.778127
C	-4.62125	-1.71672	-1.13841
C	-5.40653	-0.19714	1.07917
H	-3.32335	0.115144	1.44184
C	-5.96679	-1.57184	-0.85071
H	-4.30793	-2.31098	-1.98962
C	-6.35994	-0.80986	0.257403
H	-5.72497	0.36241	1.951311
H	-6.71679	-2.0475	-1.47236
C	1.128272	3.591833	0.35173
C	-1.51082	3.436093	-0.47522
C	0.414609	4.749434	0.083241
H	0.891923	5.715819	0.207142
C	-0.91574	4.673739	-0.33529
H	-1.47954	5.57703	-0.54001
H	2.157162	3.667684	0.689474
H	-2.54949	3.375552	-0.78694
C	7.841929	-0.62864	-0.4582
C	-7.75607	-0.66021	0.560225
N	8.964822	-0.5352	-0.72648
N	-8.88093	-0.53153	0.803337

H	-0.90868	-2.44517	-1.2545

Table S121. Coordinates for the optimized geometry of *anti*-K-**4c** (S_0).

Atom	*x*	*y*	*z*
C	0.142597	3.434682	-0.33755
C	1.396533	2.795711	-0.11569
C	1.582972	1.441012	0.296173
C	-1.15757	2.822155	-0.17976
C	0.711981	0.475768	0.745628
C	-1.55449	1.641024	0.347351
C	-0.72351	0.665502	1.038967
H	2.628676	1.144915	0.304267
H	-1.98254	3.451565	-0.50916
C	1.273884	-0.81443	1.145573
O	0.649063	-1.53682	1.992287
O	-1.26746	-0.07709	1.876256
C	2.519466	-1.37169	0.626097
C	3.274327	-2.21029	1.463027
C	2.927884	-1.14955	-0.70004
C	4.444615	-2.78199	0.995673
H	2.94756	-2.39447	2.480474
C	4.087331	-1.73914	-1.17495
H	2.317034	-0.55955	-1.37528
C	4.852623	-2.54494	-0.3234
H	5.043516	-3.41484	1.640653
H	4.400031	-1.58992	-2.20215
C	-3.06233	1.399952	0.387296
O	-3.79161	2.326062	0.666534
C	-3.59066	0.058773	0.012897
C	-2.85791	-0.83094	-0.77749
C	-4.88353	-0.27616	0.422703
C	-3.40248	-2.05037	-1.14821
H	-1.86668	-0.56441	-1.13559
C	-5.43057	-1.50071	0.071585
H	-5.44834	0.430732	1.02062
C	-4.68855	-2.38997	-0.71379
H	-2.84642	-2.74035	-1.77318
H	-6.4285	-1.77283	0.396869
C	0.144074	4.768308	-0.77309
C	2.584726	3.527287	-0.34106
C	2.555566	4.845688	-0.75009
H	3.48091	5.38885	-0.9066
C	1.324482	5.46901	-0.96672
H	1.288137	6.50287	-1.29355

H	-0.80523	5.26208	-0.95491
H	3.54169	3.039048	-0.18136
C	-5.24591	-3.66224	-1.08271
C	6.065014	-3.1395	-0.81298
N	-5.68478	-4.69198	-1.38056
N	7.045029	-3.61156	-1.21006
H	-0.2549	-1.05379	2.210009

Table S122. Coordinates for the optimized geometry of *syn*-E-**4c** (T_1).

Atom	*x*	*y*	*z*
C	0.711964	1.991877	-0.50433
C	-0.72894	1.976024	-0.5959
C	-1.53861	0.832277	-0.57178
C	1.543143	0.877715	-0.40264
C	-1.28493	-0.54103	-0.52451
C	1.288571	-0.52337	-0.40592
C	0.004818	-1.16028	-0.60488
H	-2.5975	1.06574	-0.63407
H	2.599702	1.122106	-0.34675
C	-2.4541	-1.46151	-0.50607
O	-2.32148	-2.64837	-0.84271
O	0.077055	-2.43114	-0.82724
H	-0.89009	-2.80097	-0.93494
C	-3.79954	-1.00983	-0.0686
C	-4.91299	-1.53778	-0.73162
C	-3.97753	-0.16555	1.034734
C	-6.19243	-1.19204	-0.32629
H	-4.76845	-2.21554	-1.5659
C	-5.25587	0.164383	1.45893
H	-3.1213	0.207777	1.588393
C	-6.36438	-0.3385	0.769418
H	-7.05936	-1.58416	-0.84609
H	-5.40203	0.80304	2.322826
C	2.384266	-1.39737	-0.2673
O	2.355941	-2.67078	-0.35876
C	3.789072	-1.00861	0.067892
C	4.066196	-0.35992	1.279149
C	4.819307	-1.37233	-0.80735
C	5.372707	-0.01905	1.582881
H	3.263878	-0.1225	1.97023
C	6.127654	-1.02591	-0.50197
H	4.594499	-1.90712	-1.72358
C	6.403541	-0.34824	0.6898
H	5.605876	0.493793	2.509317

H	6.935609	-1.28379	-1.17725
C	-1.41844	3.220077	-0.70515
C	1.360494	3.266809	-0.52311
C	0.657262	4.435371	-0.62932
H	1.184654	5.383406	-0.64302
C	-0.75534	4.417173	-0.7232
H	-2.50221	3.204534	-0.77423
H	-1.30609	5.347292	-0.80775
H	2.443588	3.294873	-0.45418
C	-7.68846	0.022813	1.195482
C	7.756519	0.01354	1.008398
N	-8.75329	0.322614	1.538265
N	8.845193	0.313567	1.265457

Table S123. Coordinates for the optimized geometry of *syn*-K-**4c** (T_1).

Atom	*x*	*y*	*z*
C	0.664949	2.040245	-0.45103
C	-0.77201	1.967768	-0.60722
C	-1.55766	0.815502	-0.60081
C	1.524743	0.949362	-0.33718
C	-1.28168	-0.57506	-0.49635
C	1.300851	-0.43516	-0.38343
C	0.059769	-1.15467	-0.60439
H	-2.61462	1.025897	-0.72717
H	2.573206	1.219243	-0.24435
C	-2.3818	-1.46204	-0.37274
O	-2.29607	-2.75407	-0.5565
O	0.170395	-2.38076	-0.8452
C	2.406302	-1.34528	-0.31742
O	2.23834	-2.56919	-0.54774
H	-1.37647	-3.00378	-0.8129
C	3.805927	-0.97194	0.023566
C	4.844289	-1.4398	-0.78772
C	4.079674	-0.22811	1.177208
C	6.156985	-1.12362	-0.47113
H	4.624043	-2.03545	-1.66692
C	5.3933	0.07392	1.501782
H	3.274728	0.09141	1.831642
C	6.431553	-0.36561	0.672213
H	6.970575	-1.46604	-1.10057
H	5.621021	0.642311	2.396563
C	-3.74464	-1.0429	-0.00075
C	-3.9702	-0.11522	1.025967
C	-4.83229	-1.65327	-0.63939

C	-5.26449	0.217418	1.39252
H	-3.13443	0.320834	1.56391
C	-6.1278	-1.30881	-0.28862
H	-4.65942	-2.38888	-1.41701
C	-6.34548	-0.3709	0.726896
H	-5.44447	0.921953	2.196708
H	-6.97267	-1.7652	-0.79194
C	1.28033	3.330437	-0.43508
C	-1.4829	3.201229	-0.76383
C	-0.84974	4.412342	-0.74981
H	-1.423	5.325565	-0.86803
C	0.555849	4.480441	-0.57862
H	2.357993	3.384519	-0.31233
H	1.053281	5.444041	-0.56626
H	-2.56067	3.160698	-0.88965
C	7.790735	-0.03632	1.002485
C	-7.68703	-0.01145	1.094891
N	8.884229	0.236924	1.268352
N	-8.76647	0.286474	1.390455

Table S124. Coordinates for the optimized geometry of *syn*-E-**4c** (S_1).

Atom	*x*	*y*	*z*
C	0.722518	1.901832	-0.26777
C	-0.69866	1.901064	-0.51222
C	-1.51836	0.770027	-0.54525
C	1.529126	0.773003	-0.12861
C	-1.29254	-0.60737	-0.42426
C	1.268615	-0.61522	-0.22552
C	-0.01457	-1.251	-0.41175
H	-2.564	1.013263	-0.70922
H	2.573638	1.009507	0.049141
C	-2.48277	-1.5003	-0.42685
O	-2.36888	-2.7047	-0.69584
O	0.046917	-2.53623	-0.54861
H	-0.90561	-2.89489	-0.69093
C	-3.8385	-0.99162	-0.08453
C	-4.92929	-1.51989	-0.78313
C	-4.05169	-0.09316	0.968035
C	-6.21855	-1.12297	-0.46453
H	-4.75931	-2.23948	-1.57659
C	-5.34105	0.289336	1.306541
H	-3.21561	0.285587	1.548123
C	-6.42498	-0.21587	0.580899
H	-7.06706	-1.51602	-1.01325

H	-5.51474	0.971542	2.131174
C	2.386918	-1.4845	-0.13002
O	2.466521	-2.7246	-0.18072
C	3.816316	-1.05392	0.076113
C	4.32921	-0.98103	1.382226
C	4.632258	-0.83035	-1.04588
C	5.629337	-0.55091	1.568951
H	3.702973	-1.23565	2.229939
C	5.931728	-0.40085	-0.85458
H	4.236152	-0.9691	-2.04564
C	6.428057	-0.25505	0.451806
H	6.040633	-0.45235	2.566996
H	6.573332	-0.18762	-1.70183
C	-1.36033	3.157334	-0.69132
C	1.378486	3.170632	-0.1795
C	0.702776	4.345612	-0.34827
H	1.234718	5.288722	-0.28054
C	-0.69046	4.343984	-0.61569
H	-2.42919	3.151651	-0.88354
H	-1.21725	5.281599	-0.75238
H	2.445831	3.186458	0.017333
C	-7.7593	0.198809	0.917324
C	7.777825	0.18598	0.647454
N	-8.83208	0.541255	1.187865
N	8.865831	0.551506	0.805623

Table S125. Coordinates for the optimized geometry of *anti*-E-**4c** (S_1).

Atom	*x*	*y*	*z*
C	-0.243778	3.130684	-0.309124
C	1.082891	2.625262	-0.246748
C	1.506441	1.340872	0.305876
C	-1.410585	2.482045	0.251347
C	0.855606	0.315531	0.923992
C	-1.484408	1.242128	0.842739
C	-0.552480	0.301407	1.299768
H	2.574095	1.184786	0.190764
H	-2.337438	3.037865	0.162414
C	1.674326	-0.872706	1.351930
O	1.284205	-1.581535	2.276670
O	-1.071793	-0.690244	2.027203
H	-0.322313	-1.237309	2.381711
C	2.959013	-1.211272	0.678598
C	3.977291	-1.760074	1.466007
C	3.134902	-1.092242	-0.705182

C	5.174980	-2.147375	0.886327
H	3.822656	-1.876267	2.533166
C	4.322762	-1.499694	-1.294329
H	2.335254	-0.709696	-1.331522
C	5.348751	-2.016004	-0.496259
H	5.973941	-2.557228	1.493987
H	4.459911	-1.425218	-2.367325
C	-2.929385	0.815166	1.165423
O	-3.360975	1.446367	2.101662
C	-3.659670	-0.193017	0.396346
C	-3.090367	-0.732275	-0.760966
C	-4.929787	-0.590056	0.837266
C	-3.797614	-1.673650	-1.488924
H	-2.107562	-0.415370	-1.091859
C	-5.632970	-1.535866	0.111019
H	-5.354874	-0.166593	1.740981
C	-5.066419	-2.075113	-1.050679
H	-3.377740	-2.103051	-2.391092
H	-6.615601	-1.859512	0.434583
C	-0.459339	4.374352	-0.914342
C	2.114508	3.403227	-0.779408
C	1.882404	4.650530	-1.368854
H	2.712404	5.221926	-1.769308
C	0.589315	5.135130	-1.437434
H	0.381154	6.095933	-1.895519
H	-1.471505	4.763972	-0.968630
H	3.133272	3.029482	-0.732052
C	-5.795534	-3.055227	-1.807340
C	6.587196	-2.417346	-1.105132
N	-6.380214	-3.844589	-2.420292
N	7.585148	-2.733544	-1.600574

Table S126. Coordinates for the optimized geometry of *syn*-K-**4c** (S_1).

Atom	*x*	*y*	*z*
C	-0.81305	2.16182	-0.28303
C	-1.54018	0.947556	-0.47473
C	1.410061	1.24061	0.510519
C	-1.20923	-0.4099	-0.37417
C	1.292288	-0.11936	0.29066
C	0.183135	-0.88973	-0.31184
H	-2.56383	1.126645	-0.78739
H	2.358373	1.539728	0.955086
C	-2.23209	-1.39564	-0.48744
O	-1.98168	-2.64658	-0.80375

O	0.483622	-2.0023	-0.7512
C	2.370938	-0.9991	0.881519
O	1.9445	-1.75645	1.73376
C	3.78627	-0.87237	0.503614
C	4.159804	-0.20506	-0.66816
C	4.755757	-1.46302	1.323813
C	5.498512	-0.1153	-1.01678
H	3.406823	0.224404	-1.32294
C	6.094692	-1.37003	0.984017
H	4.447404	-1.98646	2.222632
C	6.465286	-0.6934	-0.18579
H	5.801982	0.390056	-1.9266
H	6.856997	-1.8161	1.612789
C	-3.65545	-1.13084	-0.25807
C	-4.09295	-0.23195	0.728897
C	-4.60255	-1.85616	-0.99868
C	-5.44588	-0.0399	0.950889
H	-3.37331	0.287806	1.352664
C	-5.95634	-1.65462	-0.79057
H	-4.2694	-2.56857	-1.74468
C	-6.38187	-0.74233	0.18259
H	-5.78538	0.640337	1.723919
H	-6.68879	-2.20088	-1.37421
C	1.080796	3.616567	0.399294
C	-1.51475	3.347121	-0.54705
C	0.360487	4.753185	0.120821
H	0.790862	5.736198	0.27024
C	-0.95109	4.608928	-0.36358
H	-1.54165	5.489636	-0.5946
H	2.095654	3.700243	0.77668
H	-2.53773	3.282249	-0.90386
C	7.854033	-0.59054	-0.54062
C	-7.78475	-0.52752	0.400799
N	8.972392	-0.50007	-0.82763
N	-8.91513	-0.34466	0.576045
H	-1.01807	-2.75261	-0.98756
C	-0.81305	2.16182	-0.28303

Table S127. Coordinates for the optimized geometry of *anti*-K-**4c** (S_1).

Atom	*x*	*y*	*z*
C	0.081789	3.34836	-0.32326
C	1.354219	2.701527	-0.10733
C	1.58216	1.383294	0.373959
C	-1.17281	2.776281	-0.04584

C	0.754131	0.345287	0.823125
C	-1.5174	1.537976	0.471779
C	-0.68521	0.524741	1.128116
H	2.639792	1.147893	0.438906
H	-2.02779	3.406003	-0.27946
C	1.32769	-0.91802	1.134977
O	0.737241	-1.80427	1.906037
O	-1.25329	-0.23397	1.920583
C	2.616332	-1.38627	0.606986
C	3.404985	-2.22284	1.411267
C	3.042286	-1.08005	-0.69491
C	4.615063	-2.70625	0.941711
H	3.069852	-2.48094	2.409314
C	4.244326	-1.57404	-1.17357
H	2.416007	-0.48291	-1.3497
C	5.039672	-2.38024	-0.35145
H	5.23389	-3.33772	1.569244
H	4.56732	-1.35068	-2.1842
C	-3.0036	1.275459	0.602126
O	-3.59988	2.198448	1.126138
C	-3.63295	0.035275	0.123842
C	-2.94578	-0.83576	-0.72778
C	-4.95287	-0.23453	0.502092
C	-3.57393	-1.97391	-1.20598
H	-1.92626	-0.62201	-1.03646
C	-5.58164	-1.37727	0.03618
H	-5.47404	0.45178	1.160888
C	-4.89104	-2.24693	-0.81747
H	-3.05663	-2.65347	-1.87348
H	-6.60279	-1.6013	0.323152
C	0.051451	4.674644	-0.8494
C	2.507209	3.451686	-0.41298
C	2.441596	4.744267	-0.91269
H	3.361609	5.276077	-1.13239
C	1.201045	5.368512	-1.13837
H	1.154977	6.377738	-1.53043
H	-0.91565	5.14207	-1.00842
H	3.481657	3.00067	-0.25388
C	-5.53929	-3.43295	-1.30488
C	6.293711	-2.87857	-0.84325
N	-6.0569	-4.39076	-1.6997
N	7.306851	-3.2726	-1.24319
H	-0.11121	-1.42945	2.242963

Table S128. Coordinates for the optimized geometry of *syn*-E-**4d** (S_0).

Atom	*x*	*y*	*z*
C	0.419145	2.601165	1.001651
C	-0.82791	2.536942	0.316428
C	-1.45929	1.345584	-0.15454
C	1.262898	1.47822	1.290077
C	-1.06373	0.034721	-0.24343
C	1.214233	0.169538	0.918537
C	0.249648	-0.46478	0.073476
H	-2.4541	1.527023	-0.55373
H	2.134318	1.73411	1.891695
C	-2.03323	-0.95574	-0.85098
O	-1.57476	-1.91009	-1.48675
O	0.626264	-1.61419	-0.38499
C	2.269639	-0.7696	1.518286
O	1.876766	-1.54508	2.363793
C	3.670301	-0.64263	1.086506
C	4.054432	0.1543	0.000877
C	4.646684	-1.37361	1.778098
C	5.38125	0.218884	-0.38996
H	3.311774	0.704962	-0.56942
C	5.975066	-1.2968	1.405431
H	4.344391	-1.9848	2.622041
C	6.355377	-0.502	0.31335
H	5.671887	0.800844	-1.25745
H	6.734552	-1.83054	1.965875
C	-3.48169	-0.79022	-0.68509
C	-4.05693	-0.18592	0.445018
C	-4.32353	-1.35199	-1.65859
C	-5.43123	-0.11789	0.582851
H	-3.43183	0.211867	1.23891
C	-5.69631	-1.27546	-1.52976
H	-3.88326	-1.85929	-2.51043
C	-6.26811	-0.65256	-0.40857
H	-5.86948	0.365868	1.448221
H	-6.34091	-1.72928	-2.27392
C	0.876785	3.856595	1.452054
C	-1.5512	3.735024	0.11115
C	0.154372	5.010254	1.226488
H	0.533642	5.963307	1.57944
C	-1.07043	4.9502	0.547655
H	-1.64146	5.855426	0.372321
H	1.821119	3.909713	1.984797
H	-2.50608	3.688869	-0.40364

H	-0.10567	-1.98442	-0.98006
C	8.564588	-1.52415	-0.22971
C	8.405651	0.742042	-0.37016
C	8.30665	-2.88828	-0.10766
C	9.825729	-1.04563	-0.62806
C	7.99392	2.071715	-0.30095
C	9.726039	0.398041	-0.71329
C	9.35451	-3.76577	-0.35749
H	7.324503	-3.26424	0.157539
C	10.86283	-1.94437	-0.87476
C	8.925187	3.052793	-0.61912
H	6.989198	2.342838	0.004837
C	10.64414	1.39868	-1.02839
C	10.62276	-3.3033	-0.73016
H	9.180851	-4.83333	-0.26553
H	11.84024	-1.58443	-1.1816
C	10.23568	2.724383	-0.98674
H	8.628676	4.096271	-0.57515
H	11.66509	1.142503	-1.29487
H	11.41965	-4.01583	-0.91608
H	10.93834	3.514429	-1.23052
C	-8.55176	-0.18613	-1.28887
C	-8.38822	-0.83744	0.890623
C	-8.29865	0.233464	-2.59318
C	-9.85491	-0.20139	-0.7611
C	-7.95443	-1.31683	2.124932
C	-9.75136	-0.62068	0.623371
C	-9.38777	0.6003	-3.37492
H	-7.2925	0.288993	-2.99414
C	-10.9329	0.171653	-1.5621
C	-8.91538	-1.5336	3.105354
H	-6.91113	-1.53881	2.320929
C	-10.6989	-0.84554	1.62076
C	-10.6934	0.564145	-2.87161
H	-9.21755	0.92461	-4.39673
H	-11.9428	0.160632	-1.16351
C	-10.2731	-1.29349	2.86337
H	-8.60299	-1.90549	4.076068
H	-11.7537	-0.68118	1.423261
H	-11.522	0.854127	-3.50912
H	-10.9978	-1.47285	3.650598
N	-7.65199	-0.57192	-0.2784
N	7.696292	-0.43154	-0.07306

Table S129. Coordinates for the optimized geometry of *syn*-K-**4d** (S_0).

Atom	*x*	*y*	*z*
C	0.525994	2.562218	0.345768
C	-0.7815	2.591742	-0.21104
C	-1.54381	1.446482	-0.62129
C	1.323495	1.370937	0.567529
C	-1.20105	0.1371	-0.82354
C	1.194834	0.106412	0.115064
C	0.176648	-0.40074	-0.80704
H	-2.57444	1.690461	-0.86689
H	2.206163	1.549763	1.179889
C	-2.24483	-0.78673	-1.28794
O	-1.91012	-1.7713	-2.04833
O	0.463355	-1.34936	-1.54703
C	2.144487	-0.96993	0.629793
O	1.635466	-1.92233	1.185595
C	3.603833	-0.78994	0.489012
C	4.170197	0.136342	-0.3948
C	4.446271	-1.61756	1.243262
C	5.546234	0.235147	-0.5243
H	3.53585	0.75815	-1.01935
C	5.820331	-1.50507	1.137601
H	4.001895	-2.33446	1.925669
C	6.38312	-0.5789	0.248139
H	5.983938	0.920001	-1.24225
H	6.471277	-2.1165	1.752871
C	-3.63361	-0.67938	-0.94109
C	-4.06897	-0.05427	0.248562
C	-4.59613	-1.2918	-1.77539
C	-5.40686	-0.01396	0.572242
H	-3.34944	0.368767	0.941284
C	-5.93419	-1.23171	-1.46495
H	-4.27317	-1.81348	-2.6695
C	-6.36518	-0.58892	-0.28559
H	-5.73044	0.486231	1.476928
H	-6.66131	-1.72373	-2.09977
C	1.102143	3.775828	0.753876
C	-1.44059	3.834249	-0.33674
C	0.443515	4.983972	0.598749
H	0.92407	5.903915	0.914759
C	-0.83911	5.016187	0.046922
H	-1.36309	5.958292	-0.07073
H	2.093179	3.761672	1.196991
H	-2.44447	3.8557	-0.75098

H	-0.8917	-1.78018	-2.12743
C	8.669733	-1.53981	-0.02089
C	8.50854	0.722282	0.143214
C	8.411466	-2.90388	-0.145
C	9.977012	-1.0266	-0.10403
C	8.071112	2.033412	0.323235
C	9.874983	0.415076	0.004656
C	9.498752	-3.75075	-0.3197
H	7.401597	-3.29891	-0.12148
C	11.05347	-1.89537	-0.28053
C	9.030455	3.038458	0.328231
H	7.022303	2.268574	0.469228
C	10.82046	1.439759	0.012879
C	10.80831	-3.25731	-0.38054
H	9.325126	-4.81802	-0.41582
H	12.06626	-1.50897	-0.3448
C	10.39157	2.750394	0.168237
H	8.715467	4.068545	0.464449
H	11.87688	1.212433	-0.09411
H	11.63502	-3.94692	-0.51613
H	11.11532	3.558836	0.175333
C	-8.76126	-0.27275	-0.87703
C	-8.2699	-0.72109	1.313293
C	-8.7113	0.055388	-2.22967
C	-9.97444	-0.28432	-0.16942
C	-7.65722	-1.09277	2.50765
C	-9.66354	-0.57569	1.21799
C	-9.91494	0.320289	-2.8737
H	-7.77671	0.125157	-2.77453
C	-11.1695	-0.01373	-0.83192
C	-8.46959	-1.26043	3.623655
H	-6.59117	-1.27593	2.579456
C	-10.4606	-0.74973	2.347071
C	-11.1338	0.277798	-2.18882
H	-9.90269	0.57076	-3.92958
H	-12.1111	-0.02244	-0.29191
C	-9.85494	-1.08106	3.55153
H	-8.01525	-1.54646	4.566796
H	-11.5385	-0.63928	2.281106
H	-12.0556	0.487525	-2.72096
H	-10.4606	-1.21941	4.440852
N	-7.70883	-0.53581	0.029226
N	7.773778	-0.47105	0.129251

Table S130. Coordinates for the optimized geometry of *anti*-E-**4d** (S_0).

Atom	*x*	*y*	*z*
C	-0.03027	5.177562	1.007095
C	-1.28167	4.613769	0.625732
C	-1.45519	3.452914	-0.18844
C	1.255317	4.678949	0.61952
C	-0.59448	2.687598	-0.93301
C	1.651978	3.710529	-0.25496
C	0.809388	2.955659	-1.12998
H	-2.49386	3.140429	-0.26306
H	2.089763	5.208901	1.07552
C	-1.1773	1.544324	-1.73343
O	-0.65031	1.254297	-2.81254
O	1.418138	2.44038	-2.14788
H	0.746632	1.920212	-2.70231
C	-2.33475	0.791223	-1.23808
C	-3.14841	0.142132	-2.18107
C	-2.60555	0.614483	0.12906
C	-4.22178	-0.62533	-1.77436
H	-2.91762	0.24082	-3.23643
C	-3.67286	-0.16254	0.540366
H	-1.9675	1.065599	0.883167
C	-4.50092	-0.78433	-0.40705
H	-4.83301	-1.14045	-2.50664
H	-3.88875	-0.2779	1.596294
C	3.171459	3.562839	-0.41918
O	3.822989	4.577971	-0.55282
C	3.770735	2.217073	-0.32878
C	3.076858	1.114448	0.182996
C	5.101816	2.056954	-0.73228
C	3.693418	-0.11984	0.289898
H	2.057913	1.22125	0.547413
C	5.715712	0.819678	-0.65437
H	5.636144	2.912696	-1.13113
C	5.017548	-0.28086	-0.13932
H	3.170272	-0.96195	0.729161
H	6.731095	0.687601	-1.01136
C	6.914179	-1.78106	0.474692
C	5.081368	-2.75225	-0.45984
C	7.823495	-0.89885	1.055194
C	7.172478	-3.16183	0.398741
C	3.864975	-3.00789	-1.09046
C	6.006354	-3.78044	-0.20041
C	9.018555	-1.42627	1.528367

H	7.611507	0.160499	1.151254
C	8.378451	-3.66942	0.880794
C	3.576791	-4.32527	-1.42548
H	3.169353	-2.21129	-1.33192
C	5.696295	-5.09522	-0.54535
C	9.300552	-2.79517	1.43814
H	9.745895	-0.75977	1.981245
H	8.587969	-4.73343	0.825183
C	4.476567	-5.36257	-1.15109
H	2.635655	-4.55106	-1.91749
H	6.403681	-5.89536	-0.34926
H	10.24479	-3.17372	1.815468
H	4.220985	-6.38083	-1.42527
C	-5.57425	-2.50498	1.043252
C	-6.88202	-1.50622	-0.53649
C	-4.51317	-2.95779	1.824614
C	-6.86209	-3.05638	1.161004
C	-7.40021	-0.68122	-1.53251
C	-7.69489	-2.41744	0.160226
C	-4.78024	-3.94867	2.761996
H	-3.50551	-2.57592	1.702091
C	-7.10806	-4.05012	2.106973
C	-8.7473	-0.81816	-1.84634
H	-6.79073	0.055378	-2.0444
C	-9.04379	-2.53799	-0.16956
C	-6.06422	-4.48572	2.911251
H	-3.97102	-4.3172	3.384516
H	-8.09944	-4.48179	2.204891
C	-9.56244	-1.74066	-1.1802
H	-9.17363	-0.1913	-2.62316
H	-9.67836	-3.24059	0.361908
H	-6.23938	-5.25795	3.652923
H	-10.6096	-1.82457	-1.4513
N	5.635887	-1.53179	-0.0484
N	-5.58366	-1.55578	0.004558
C	-0.02879	6.307986	1.849978
C	-2.46933	5.209098	1.107784
C	-2.43717	6.324753	1.917056
H	-3.36289	6.768655	2.266824
C	-1.20562	6.8787	2.290933
H	0.922399	6.737239	2.148528
H	-1.17534	7.753842	2.931135
H	-3.42487	4.776516	0.827104

Table S131. Coordinates for the optimized geometry of *anti*-K-**4d** (S_0).

Atom	*x*	*y*	*z*
C	-0.04722	-5.52196	0.786741
C	1.177542	-4.94082	0.355427
C	1.314849	-3.68152	-0.31869
C	-1.36321	-4.94247	0.61527
C	0.399048	-2.86124	-0.92108
C	-1.80754	-3.92256	-0.15092
C	-1.02666	-3.18894	-1.1429
H	2.349335	-3.36094	-0.41605
H	-2.1511	-5.4528	1.167024
C	0.882457	-1.64119	-1.57869
O	0.258027	-1.20842	-2.62042
O	-1.59126	-2.76812	-2.16105
C	-3.30518	-3.63231	-0.10144
O	-4.07807	-4.56354	-0.01075
H	-0.56423	-1.79716	-2.7657
C	-3.75261	-2.21862	-0.09808
C	-5.08445	-1.94221	-0.42244
C	-2.9082	-1.16441	0.265148
C	-5.55281	-0.63896	-0.42214
H	-5.73797	-2.76264	-0.69942
C	-3.37755	0.137833	0.299172
H	-1.88388	-1.35876	0.575202
C	-4.70242	0.412202	-0.05971
H	-6.57284	-0.42116	-0.71961
H	-2.73587	0.949799	0.623776
C	2.004723	-0.86873	-1.1292
C	2.435497	-0.8733	0.216491
C	2.653652	-0.00791	-2.04385
C	3.483277	-0.07782	0.622223
H	1.921807	-1.47856	0.955644
C	3.715029	0.769436	-1.64528
H	2.308022	0.037127	-3.07074
C	4.151124	0.7491	-0.30337
H	3.818184	-0.10696	1.651898
H	4.189371	1.442122	-2.34967
C	2.404321	-6.82734	1.293736
C	1.20071	-7.396	1.716477
C	0.002692	-6.74363	1.477094
C	2.386783	-5.61303	0.636504
H	3.346078	-7.32952	1.485979
H	1.201238	-8.34575	2.240885
H	-0.92602	-7.18715	1.822195

H	3.323379	-5.16376	0.319
C	-6.35442	2.181896	0.550542
C	-4.5269	2.831664	-0.63282
C	-7.29701	1.471351	1.29168
C	-6.46547	3.570598	0.352208
C	-3.35345	2.8773	-1.384
C	-5.3015	3.985026	-0.40548
C	-8.37609	2.179084	1.805709
H	-7.19455	0.40728	1.475703
C	-7.55745	4.260611	0.878276
C	-2.95203	4.112182	-1.87815
H	-2.77863	1.981548	-1.59449
C	-4.87878	5.213821	-0.9111
C	-8.51255	3.557937	1.598572
H	-9.12684	1.649129	2.38357
H	-7.6539	5.331966	0.729528
C	-3.69974	5.272505	-1.64092
H	-2.04222	4.175132	-2.46757
H	-5.46916	6.109293	-0.7415
H	-9.3694	4.07948	2.012298
H	-3.35856	6.221764	-2.04094
C	5.287658	2.268016	1.308702
C	6.405503	1.75343	-0.6225
C	4.329044	2.452296	2.302135
C	6.522533	2.935527	1.34863
C	6.835867	1.19049	-1.82147
C	7.234017	2.603486	0.127873
C	4.658808	3.278345	3.371058
H	3.345969	1.998517	2.249745
C	6.833769	3.760357	2.427006
C	8.099754	1.543117	-2.28198
H	6.231146	0.485983	-2.38106
C	8.497939	2.942792	-0.34889
C	5.900393	3.918281	3.442565
H	3.929925	3.434394	4.159786
H	7.786341	4.279549	2.464721
C	8.921048	2.416909	-1.56212
H	8.454713	1.123558	-3.21775
H	9.143477	3.600315	0.22497
H	6.127036	4.556046	4.290323
H	9.901225	2.67336	-1.94983
N	-5.17008	1.733403	-0.04995
N	5.208567	1.538836	0.099628

Table S132. Coordinates for the optimized geometry of *syn*-E-**4d** (T_1).

Atom	*x*	*y*	*z*
C	0.499342	2.296181	1.257783
C	-0.80583	2.28355	0.665204
C	-1.4706	1.168475	0.126282
C	1.355789	1.149345	1.369384
C	-1.11078	-0.21494	-0.04155
C	1.248369	-0.14336	0.965098
C	0.169826	-0.76525	0.221537
H	-2.436	1.4171	-0.30407
H	2.291922	1.368418	1.883347
C	-2.09189	-1.13479	-0.56862
O	-1.81159	-2.25973	-1.04747
O	0.472208	-1.96494	-0.18867
C	2.34106	-1.08988	1.442172
O	2.046963	-1.98205	2.210841
C	3.740778	-0.852	0.999646
C	4.062225	0.002879	-0.05923
C	4.767454	-1.54284	1.654043
C	5.380518	0.163269	-0.46034
H	3.277344	0.526099	-0.59645
C	6.087156	-1.36701	1.276185
H	4.509381	-2.20519	2.473685
C	6.402112	-0.5139	0.211374
H	5.626319	0.795518	-1.30681
H	6.886095	-1.87298	1.807786
C	-3.56527	-0.82956	-0.51956
C	-4.17606	-0.25241	0.599417
C	-4.35884	-1.27832	-1.58093
C	-5.55451	-0.11741	0.659554
H	-3.56887	0.088183	1.431599
C	-5.73506	-1.12603	-1.54998
H	-3.88204	-1.77294	-2.41978
C	-6.32739	-0.54941	-0.42239
H	-6.02693	0.348724	1.518138
H	-6.35201	-1.49802	-2.36162
C	1.01281	3.504618	1.77136
C	-1.50274	3.529178	0.617432
C	0.306633	4.688127	1.712914
H	0.730146	5.602208	2.114055
C	-0.96903	4.689995	1.121424
H	-1.5366	5.613662	1.062118
H	2.000694	3.494144	2.224029
H	-2.49026	3.546385	0.164157

H	-0.3325	-2.34436	-0.67188
C	8.657874	-1.3645	-0.43701
C	8.381698	0.886914	-0.37849
C	8.47144	-2.7461	-0.42038
C	9.891464	-0.79066	-0.79926
C	7.894669	2.181342	-0.20209
C	9.716278	0.646986	-0.75873
C	9.559394	-3.54545	-0.74621
H	7.511835	-3.18785	-0.17406
C	10.96998	-1.61374	-1.12314
C	8.766067	3.235803	-0.44269
H	6.875807	2.364762	0.122074
C	10.57308	1.721794	-0.99479
C	10.7995	-2.99019	-1.08897
H	9.441618	-4.62472	-0.73811
H	11.92615	-1.18118	-1.40272
C	10.09096	3.013795	-0.84078
H	8.410896	4.253845	-0.3143
H	11.60386	1.547326	-1.28899
H	11.62948	-3.64409	-1.33645
H	10.74542	3.86029	-1.022
C	-8.52278	0.186426	-1.34516
C	-8.54519	-0.86302	0.665253
C	-8.11689	0.818079	-2.52277
C	-9.87471	0.130203	-0.93684
C	-8.17901	-1.5777	1.809381
C	-9.88971	-0.54701	0.361068
C	-9.11647	1.365434	-3.32204
H	-7.07112	0.890425	-2.79893
C	-10.8496	0.685814	-1.7371
C	-9.20074	-1.93499	2.683814
H	-7.14908	-1.85636	2.001405
C	-10.8884	-0.91725	1.235075
C	-10.4579	1.297129	-2.94092
H	-8.84634	1.85948	-4.24847
H	-11.8955	0.662714	-1.44974
C	-10.5296	-1.60698	2.40694
H	-8.96074	-2.48619	3.585948
H	-11.9298	-0.69432	1.028359
H	-11.2155	1.73605	-3.58142
H	-11.3063	-1.9033	3.103904
N	-7.73596	-0.41354	-0.36892
N	7.739863	-0.3399	-0.18204

Table S133. Coordinates for the optimized geometry of *syn*-K-**4d** (T_1).

Atom	*x*	*y*	*z*
C	0.574206	2.551546	0.457915
C	-0.73109	2.616284	-0.1017
C	-1.50847	1.511615	-0.57943
C	1.351096	1.342131	0.612079
C	-1.211	0.176401	-0.83222
C	1.193429	0.101388	0.103202
C	0.171741	-0.36461	-0.84436
H	-2.51992	1.802841	-0.85104
H	2.239378	1.473934	1.22925
C	-2.24004	-0.73147	-1.22992
O	-1.97295	-1.83949	-1.92229
O	0.470583	-1.28317	-1.61223
C	2.129495	-0.99816	0.572574
O	1.641446	-1.98239	1.091512
C	3.598303	-0.81668	0.437483
C	4.164682	0.125075	-0.4273
C	4.438887	-1.65727	1.176352
C	5.542399	0.22659	-0.55306
H	3.527555	0.760155	-1.03486
C	5.814484	-1.54111	1.077429
H	3.991625	-2.3891	1.840818
C	6.375904	-0.59881	0.20707
H	5.98214	0.928007	-1.25402
H	6.466043	-2.16454	1.680429
C	-3.65841	-0.59697	-0.90921
C	-4.13113	0.105898	0.21674
C	-4.59463	-1.28417	-1.70934
C	-5.48036	0.140737	0.52092
H	-3.4318	0.603522	0.878829
C	-5.94604	-1.24675	-1.42015
H	-4.23944	-1.85814	-2.5565
C	-6.38713	-0.53151	-0.30347
H	-5.83448	0.697211	1.383066
H	-6.65514	-1.79717	-2.03072
C	1.16994	3.73535	0.933265
C	-1.35958	3.884666	-0.16301
C	0.537547	4.960138	0.846678
H	1.029513	5.855295	1.212019
C	-0.7439	5.032266	0.288608
H	-1.25517	5.986704	0.215283
H	2.161023	3.675409	1.374227
H	-2.35799	3.947261	-0.58732

H	-0.99274	-1.85773	-2.08514
C	8.666734	-1.54016	-0.10817
C	8.503586	0.704881	0.178424
C	8.40803	-2.89665	-0.30023
C	9.975762	-1.02438	-0.15744
C	8.060596	2.004491	0.420602
C	9.872134	0.408701	0.028806
C	9.49505	-3.73432	-0.51337
H	7.396809	-3.28912	-0.29688
C	11.05195	-1.88504	-0.37341
C	9.0167	3.010134	0.481817
H	7.009133	2.227111	0.567667
C	10.81416	1.435028	0.094127
C	10.80577	-3.23961	-0.54456
H	9.321216	-4.79544	-0.66348
H	12.06572	-1.49741	-0.41224
C	10.38021	2.734361	0.315148
H	8.697685	4.031386	0.667154
H	11.87185	1.21704	-0.02095
H	11.63243	-3.92247	-0.71193
H	11.10125	3.543762	0.367129
C	-8.77995	-0.09287	-0.85006
C	-8.32556	-0.88899	1.219606
C	-8.66077	0.410043	-2.1478
C	-10.0231	-0.2094	-0.18546
C	-7.67797	-1.41711	2.340064
C	-9.72919	-0.72232	1.154535
C	-9.83963	0.769002	-2.79485
H	-7.69409	0.523577	-2.62543
C	-11.1783	0.158368	-0.84003
C	-8.47562	-1.74503	3.432122
H	-6.60565	-1.577	2.352162
C	-10.5023	-1.05987	2.243455
C	-11.0752	0.642621	-2.15671
H	-9.79538	1.159056	-3.80542
H	-12.1476	0.084786	-0.35852
C	-9.86001	-1.56605	3.388479
H	-8.01559	-2.15496	4.324104
H	-11.5814	-0.94905	2.225741
H	-11.9776	0.933277	-2.68403
H	-10.4578	-1.83497	4.252898
N	-7.77258	-0.50359	0.009522
N	7.771883	-0.484	0.095952

Table S134. Coordinates for the optimized geometry of *anti*-E-**4d** (T_1).

Atom	*x*	*y*	*z*
C	0.013252	4.999561	0.966542
C	-1.24298	4.341198	0.747005
C	-1.46122	3.190862	-0.02639
C	1.262685	4.574308	0.420522
C	-0.60905	2.371951	-0.84526
C	1.628496	3.558024	-0.41381
C	0.762268	2.642788	-1.11967
H	-2.50771	2.906263	-0.07471
H	2.106004	5.190626	0.727978
C	-1.16369	1.218453	-1.50853
O	-0.60129	0.619011	-2.46082
O	1.375442	1.999194	-2.07247
H	0.714656	1.342583	-2.4854
C	-2.46085	0.596365	-1.06851
C	-3.29966	0.076241	-2.06071
C	-2.78755	0.399957	0.278006
C	-4.46385	-0.5942	-1.7246
H	-3.0123	0.185308	-3.10049
C	-3.93524	-0.29234	0.633056
H	-2.13742	0.788729	1.054832
C	-4.77381	-0.77875	-0.3739
H	-5.10125	-1.01737	-2.49435
H	-4.19978	-0.42526	1.677131
C	3.120897	3.472471	-0.68734
O	3.737201	4.489056	-0.94228
C	3.819749	2.166275	-0.52623
C	3.259773	1.098979	0.180519
C	5.107521	2.034439	-1.05209
C	3.968953	-0.0786	0.358307
H	2.274118	1.195123	0.62774
C	5.810406	0.848917	-0.90597
H	5.539241	2.869319	-1.59372
C	5.244801	-0.21424	-0.19607
H	3.551579	-0.89487	0.93846
H	6.793529	0.731288	-1.34955
C	7.263228	-1.53968	0.444147
C	5.469359	-2.69562	-0.31994
C	8.136324	-0.55015	0.894073
C	7.615123	-2.90293	0.468331
C	4.240897	-3.07553	-0.85943
C	6.469793	-3.64195	-0.02258
C	9.385485	-0.95372	1.3466

H	7.852873	0.496985	0.90049
C	8.876085	-3.28418	0.927722
C	4.02291	-4.43067	-1.07161
H	3.486323	-2.34062	-1.11977
C	6.22701	-4.9975	-0.24361
C	9.758194	-2.30489	1.360524
H	10.08617	-0.20332	1.699693
H	9.159207	-4.33251	0.948967
C	5.000258	-5.3864	-0.76296
H	3.075789	-4.75331	-1.49396
H	6.991424	-5.73553	-0.01877
H	10.74365	-2.58493	1.718499
H	4.798237	-6.43768	-0.94189
C	-6.01165	-2.56575	0.842978
C	-7.22847	-1.19009	-0.48724
C	-4.95985	-3.22619	1.483658
C	-7.3558	-2.99282	0.94547
C	-7.6316	-0.15019	-1.32853
C	-8.14118	-2.10479	0.086305
C	-5.29263	-4.31587	2.282659
H	-3.92927	-2.91616	1.353328
C	-7.66449	-4.07945	1.73449
C	-8.98894	-0.06878	-1.62511
H	-6.92328	0.568489	-1.72483
C	-9.48268	-2.00271	-0.21215
C	-6.6192	-4.73249	2.41164
H	-4.50811	-4.85462	2.801846
H	-8.6849	-4.43508	1.830148
C	-9.89785	-0.97948	-1.08261
H	-9.3446	0.719134	-2.27935
H	-10.2088	-2.68639	0.214678
H	-6.8489	-5.58862	3.036977
H	-10.9507	-0.88956	-1.3278
N	5.95553	-1.41829	-0.03462
N	-5.95267	-1.47999	-0.02029
C	0.055822	6.15141	1.783939
C	-2.39043	4.91587	1.376556
C	-2.31157	6.039471	2.159814
H	-3.21089	6.441835	2.616034
C	-1.07303	6.674638	2.374232
H	1.01595	6.634887	1.941114
H	-1.00969	7.563146	2.992502
H	-3.35392	4.438488	1.219526

Table S135. Coordinates for the optimized geometry of *anti*-K-**4d** (T_1).

Atom	*x*	*y*	*z*
C	2.552379	5.366961	0.626633
C	1.223711	5.132273	0.148945
C	0.70553	3.923202	-0.40355
C	3.598364	4.379825	0.655149
C	1.337177	2.765005	-0.89545
C	3.688026	3.184735	0.024044
C	2.800334	2.662275	-1.02378
H	-0.37083	3.94102	-0.5523
H	4.454941	4.626939	1.281938
C	0.605539	1.639593	-1.33047
O	1.164056	0.695073	-2.10721
O	3.322595	1.990214	-1.92306
C	-0.76928	1.305941	-0.95097
C	-1.56307	0.574397	-1.84977
C	-1.2966	1.603517	0.314376
C	-2.83585	0.156104	-1.4971
H	-1.16695	0.334583	-2.83098
C	-2.57385	1.194409	0.666582
H	-0.6984	2.152493	1.035057
C	-3.35234	0.459451	-0.23243
H	-3.43999	-0.4145	-2.1956
H	-2.97947	1.444655	1.641209
C	4.818211	2.261469	0.36558
O	5.929698	2.618242	0.685743
C	4.426759	0.79936	0.365249
C	3.207323	0.423743	0.934825
C	5.233625	-0.15701	-0.25055
C	2.751228	-0.87688	0.822947
H	2.59621	1.160424	1.446914
C	4.785896	-1.46356	-0.38309
H	6.183627	0.143826	-0.67883
C	3.530828	-1.80667	0.130843
H	1.807195	-1.17486	1.266358
H	5.366763	-2.19284	-0.93835
C	3.650311	-4.30492	0.229262
C	1.735314	-3.36775	-0.55142
C	4.914159	-4.49854	0.784723
C	2.769963	-5.37131	-0.05518
C	0.779315	-2.4611	-1.02974
C	1.530473	-4.76924	-0.55053
C	5.30848	-5.81537	1.011082
H	5.558903	-3.6661	1.042871

C	3.174036	-6.66812	0.184896
C	-0.43599	-2.99594	-1.44412
H	0.976162	-1.39562	-1.11334
C	0.325656	-5.27648	-0.98224
C	4.458369	-6.88083	0.712029
H	6.286823	-6.01163	1.435057
H	2.518758	-7.50852	-0.01831
C	-0.66447	-4.37321	-1.41467
H	-1.21011	-2.32486	-1.79925
H	0.134763	-6.34435	-0.99431
H	4.790271	-7.89577	0.90308
H	-1.62156	-4.76077	-1.74756
C	-4.95098	-0.69627	1.292968
C	-5.802	0.17228	-0.62385
C	-4.11737	-1.1311	2.322335
C	-6.32609	-0.99999	1.279282
C	-5.99302	0.846389	-1.82898
C	-6.86966	-0.44249	0.056872
C	-4.69552	-1.85564	3.357224
H	-3.05337	-0.91855	2.319661
C	-6.88426	-1.72864	2.329237
C	-7.27811	0.868614	-2.35551
H	-5.17651	1.349905	-2.33585
C	-8.15214	-0.40588	-0.49065
C	-6.06524	-2.14934	3.367685
H	-4.06988	-2.20019	4.175105
H	-7.94406	-1.96547	2.330371
C	-8.34847	0.245849	-1.69967
H	-7.45525	1.386166	-3.29323
H	-8.98389	-0.87543	0.026082
H	-6.48639	-2.7134	4.193509
H	-9.34001	0.282868	-2.1388
N	3.006395	-3.10467	-0.07099
N	-4.63474	0.018513	0.133027
C	2.867272	6.619508	1.192892
C	0.292041	6.197191	0.277888
C	0.63822	7.41871	0.810134
H	-0.10409	8.207639	0.876282
C	1.943075	7.640165	1.273343
H	3.87463	6.777422	1.567873
H	2.220719	8.597971	1.699787
H	-0.72477	6.036095	-0.06848
H	2.059394	1.027477	-2.37383

Table S136. Coordinates for the optimized geometry of *syn*-E-**4d** (S_1).

Atom	*x*	*y*	*z*
C	-0.41602	1.908678	-1.92399
C	0.963574	1.788348	-1.5429
C	1.594124	0.648094	-1.031
C	-1.36233	0.837829	-1.88999
C	1.12814	-0.66139	-0.67038
C	-1.29679	-0.46532	-1.49707
C	-0.1949	-1.15222	-0.86991
H	2.645832	0.808197	-0.83019
H	-2.34059	1.124538	-2.27592
C	2.055568	-1.5761	-0.06424
O	1.782823	-2.72991	0.340877
O	-0.51585	-2.34232	-0.44919
C	-2.50616	-1.32754	-1.83071
O	-2.37592	-2.23728	-2.62262
C	-3.82128	-0.9872	-1.22312
C	-3.94788	-0.11887	-0.13482
C	-4.96811	-1.59242	-1.74964
C	-5.19294	0.138007	0.421047
H	-3.06695	0.339373	0.303919
C	-6.21616	-1.31983	-1.21682
H	-4.8611	-2.26588	-2.59348
C	-6.33565	-0.45453	-0.12278
H	-5.28782	0.781449	1.289212
H	-7.10897	-1.75935	-1.64868
C	3.500369	-1.18728	0.111231
C	4.412094	-1.39807	-0.92892
C	3.948722	-0.69102	1.339794
C	5.751234	-1.10596	-0.75578
H	4.059148	-1.76824	-1.88542
C	5.28245	-0.38045	1.525577
H	3.24641	-0.5655	2.156705
C	6.186475	-0.58885	0.473632
H	6.452176	-1.21826	-1.57585
H	5.641388	-0.03716	2.489841
C	-0.89408	3.144608	-2.40617
C	1.776193	2.95478	-1.70079
C	-0.08229	4.250936	-2.53052
H	-0.47976	5.187098	-2.90451
C	1.274705	4.140338	-2.17311
H	1.930153	4.999497	-2.2735
H	-1.93976	3.215079	-2.6924
H	2.825435	2.886513	-1.43013

H	0.303196	-2.77156	-0.03629
C	-8.55214	-1.13912	0.799597
C	-8.12025	1.085886	0.696837
C	-8.47153	-2.53052	0.76488
C	-9.68601	-0.47766	1.308475
C	-7.56397	2.341368	0.455906
C	-9.41177	0.943355	1.239887
C	-9.56699	-3.24931	1.225269
H	-7.58493	-3.03993	0.402636
C	-10.7742	-1.22091	1.765848
C	-8.31863	3.456445	0.79684
H	-6.5821	2.449842	0.00755
C	-10.1509	2.077736	1.574545
C	-10.711	-2.60594	1.716267
H	-9.53131	-4.33425	1.206291
H	-11.6544	-0.72071	2.158921
C	-9.59718	3.331167	1.356054
H	-7.90788	4.446011	0.62055
H	-11.1477	1.97889	1.994294
H	-11.5502	-3.1983	2.066261
H	-10.1601	4.22293	1.612132
C	8.036578	0.899164	1.207765
C	8.612247	-1.10067	0.294513
C	7.336159	2.031156	1.63252
C	9.447601	0.842809	1.207298
C	8.586337	-2.40201	-0.2107
C	9.816748	-0.44246	0.620694
C	8.086005	3.096299	2.121178
H	6.256775	2.091755	1.572155
C	10.17463	1.914515	1.683583
C	9.813123	-3.01504	-0.44729
H	7.657761	-2.9294	-0.39188
C	11.0241	-1.07105	0.390259
C	9.48001	3.039852	2.154124
H	7.577344	3.987751	2.469021
H	11.25915	1.898484	1.688028
C	11.0123	-2.36104	-0.16105
H	9.833571	-4.02264	-0.84601
H	11.96361	-0.5891	0.638294
H	10.03925	3.887347	2.534298
H	11.95305	-2.86621	-0.34912
N	7.541065	-0.27822	0.651153
N	-7.59979	-0.18378	0.428716

Table S137. Coordinates for the optimized geometry of *syn*-K-**4d** (S_1).

Atom	*x*	*y*	*z*
C	0.566489	2.641747	0.508421
C	-0.70207	2.807027	-0.1034
C	-1.49907	1.764154	-0.70165
C	1.293956	1.392752	0.595077
C	-1.22343	0.473564	-1.06255
C	1.127453	0.215726	-0.04323
C	0.144057	-0.10205	-1.08749
H	-2.50297	2.098175	-0.95123
H	2.149799	1.434989	1.267666
C	-2.28587	-0.37545	-1.59584
O	-1.99662	-1.28189	-2.52473
O	0.450314	-0.92446	-1.95296
C	2.018493	-0.95557	0.331889
O	1.485045	-1.98206	0.702691
C	3.494999	-0.79835	0.283198
C	4.122855	0.221105	-0.4395
C	4.280621	-1.74374	0.9528
C	5.506923	0.295788	-0.49426
H	3.530818	0.94094	-0.99596
C	5.661556	-1.65713	0.926579
H	3.786328	-2.53512	1.506386
C	6.28488	-0.63724	0.197201
H	5.994807	1.060518	-1.08909
H	6.269639	-2.36562	1.478851
C	-3.64482	-0.34788	-1.17477
C	-4.08933	0.278663	0.02254
C	-4.60808	-1.06483	-1.94219
C	-5.40708	0.215512	0.412759
H	-3.37778	0.770974	0.674901
C	-5.92626	-1.12108	-1.55906
H	-4.28788	-1.56763	-2.84605
C	-6.32922	-0.47605	-0.38272
H	-5.7278	0.676382	1.34128
H	-6.6517	-1.66275	-2.15741
C	1.179147	3.75838	1.107137
C	-1.28486	4.092706	-0.09203
C	0.594772	5.010161	1.087741
H	1.099395	5.853187	1.54741
C	-0.65145	5.178448	0.478108
H	-1.12381	6.154916	0.458131
H	2.143281	3.625477	1.589369
H	-2.2592	4.225449	-0.55382

H	-1.01243	-1.28895	-2.64749
C	8.560956	-1.60663	-0.13589
C	8.445552	0.594153	0.406055
C	8.274553	-2.92258	-0.4964
C	9.883981	-1.12962	-0.0742
C	8.027634	1.870055	0.781722
C	9.810742	0.274685	0.275118
C	9.346958	-3.76304	-0.76548
H	7.254105	-3.2813	-0.57806
C	10.94508	-1.9922	-0.34914
C	9.006757	2.831553	0.99661
H	6.977304	2.106953	0.913901
C	10.77617	1.256554	0.496107
C	10.67042	-3.30915	-0.68819
H	9.151449	-4.79337	-1.04639
H	11.96947	-1.63443	-0.30348
C	10.36814	2.534489	0.850952
H	8.707598	3.833913	1.287856
H	11.83154	1.020444	0.396708
H	11.48494	-3.99312	-0.90355
H	11.1077	3.309455	1.024637
C	-8.69903	0.305952	-0.33106
C	-8.20289	-1.5387	0.860094
C	-8.60619	1.424215	-1.16115
C	-9.91019	-0.1074	0.274947
C	-7.52068	-2.60911	1.44531
C	-9.588	-1.3076	1.050816
C	-9.77956	2.143718	-1.37984
H	-7.66429	1.71103	-1.61238
C	-11.0577	0.617527	0.046151
C	-8.2692	-3.46715	2.247715
H	-6.46126	-2.75875	1.276807
C	-10.308	-2.1676	1.847426
C	-10.9795	1.750522	-0.78948
H	-9.75821	3.019073	-2.01838
H	-12.0051	0.333028	0.491278
C	-9.63249	-3.25235	2.44564
H	-7.78507	-4.31232	2.722709
H	-11.3698	-2.02641	2.018424
H	-11.8782	2.327719	-0.97684
H	-10.192	-3.93583	3.074743
N	-7.69229	-0.56328	0.034082
N	7.686599	-0.55382	0.156974

Table S138. Coordinates for the optimized geometry of *anti*-E-**4d** (S_1).

Atom	*x*	*y*	*z*
C	0.14923	4.43258	-0.69115
C	1.29269	3.56987	-0.82369
C	1.45865	2.31251	-0.23860
C	-1.05088	4.08577	-0.01553
C	0.66089	1.53062	0.66105
C	-1.47089	2.96643	0.65595
C	-0.67965	1.83976	1.05555
H	2.40939	1.85762	-0.48814
H	-1.81417	4.86102	-0.05456
C	1.22475	0.33314	1.21375
O	0.63936	-0.45754	1.99350
O	-1.29582	1.05089	1.89213
H	-0.68004	0.27703	2.12329
C	2.63911	-0.06512	0.88304
C	3.68787	0.35437	1.70833
C	2.90092	-0.91675	-0.19538
C	4.98571	-0.04447	1.45087
H	3.47728	0.98665	2.56408
C	4.19093	-1.33648	-0.45930
H	2.08833	-1.23156	-0.84124
C	5.23717	-0.89306	0.36306
H	5.79482	0.24374	2.11329
H	4.40482	-1.95526	-1.32403
C	-2.90919	3.03515	1.14381
O	-3.29066	4.04196	1.70892
C	-3.84784	1.92456	0.82411
C	-3.58044	0.98279	-0.17230
C	-5.06579	1.86900	1.50676
C	-4.51039	0.00276	-0.48374
H	-2.65421	1.03215	-0.73785
C	-5.98658	0.87296	1.22336
H	-5.26954	2.60750	2.27479
C	-5.71465	-0.06403	0.22207
H	-4.32195	-0.70590	-1.28327
H	-6.91541	0.80441	1.77975
C	-8.00635	-0.86974	-0.35763
C	-6.38322	-2.43890	-0.15197
C	-8.72539	0.32170	-0.44303
C	-8.60873	-2.11673	-0.61262
C	-5.19224	-3.12237	0.09022
C	-7.57227	-3.12012	-0.47845
C	-10.07342	0.23636	-0.76514

H	-8.25282	1.28342	-0.27380
C	-9.96480	-2.17718	-0.93473
C	-5.20915	-4.50614	-0.02738
H	-4.28618	-2.59738	0.37407
C	-7.56420	-4.51027	-0.59134
C	-10.69251	-0.99815	-1.00436
H	-10.65758	1.14881	-0.83545
H	-10.44040	-3.13351	-1.13178
C	-6.37899	-5.19730	-0.37004
H	-4.29523	-5.06296	0.15715
H	-8.47513	-5.04559	-0.84262
H	-11.74872	-1.02859	-1.25167
H	-6.35783	-6.27914	-0.45398
C	6.94452	-2.61727	-0.17765
C	7.66787	-0.47601	0.05798
C	6.17773	-3.78367	-0.15779
C	8.33759	-2.63415	-0.39877
C	7.73471	0.91313	0.19623
C	8.80334	-1.25828	-0.24825
C	6.83501	-4.97933	-0.43212
H	5.12148	-3.77169	0.08214
C	8.97474	-3.83129	-0.65764
C	8.98897	1.50360	0.08021
H	6.85054	1.51336	0.37073
C	10.03680	-0.65277	-0.37251
C	8.20708	-5.00516	-0.68602
H	6.27193	-5.90540	-0.43268
H	10.04574	-3.87209	-0.82428
C	10.12281	0.73691	-0.19245
H	9.08221	2.57754	0.19125
H	10.92302	-1.22992	-0.61328
H	8.69376	-5.95241	-0.88961
H	11.08687	1.22449	-0.28385
N	-6.65134	-1.07036	-0.07932
N	6.54954	-1.30520	0.09481
C	0.16853	5.70392	-1.31158
C	2.37623	4.05533	-1.62441
C	2.35270	5.29048	-2.21460
H	3.19840	5.61818	-2.81061
C	1.23859	6.14166	-2.05503
H	-0.70122	6.34400	-1.19497
H	1.22358	7.12052	-2.51967
H	3.24241	3.41376	-1.75497

Table S139. Coordinates for the optimized geometry of *anti*-K-**4d** (S_1).

Atom	*x*	*y*	*z*
C	2.303134	5.42687	0.647563
C	0.991812	5.111775	0.176177
C	0.545658	3.870956	-0.37685
C	3.407926	4.507901	0.662906
C	1.230037	2.759755	-0.86239
C	3.563206	3.322364	0.025858
C	2.696808	2.747524	-1.00693
H	-0.53155	3.825966	-0.5116
H	4.25548	4.807149	1.278658
C	0.551703	1.588829	-1.30874
O	1.154087	0.706423	-2.11834
O	3.241078	2.112754	-1.91986
C	-0.80382	1.1972	-0.93661
C	-1.57017	0.447229	-1.84533
C	-1.35481	1.476493	0.324771
C	-2.83823	0.000673	-1.50781
H	-1.16052	0.224313	-2.82458
C	-2.62712	1.039864	0.658926
H	-0.77852	2.036104	1.055
C	-3.37883	0.292372	-0.25193
H	-3.42323	-0.57703	-2.21657
H	-3.04979	1.276254	1.629778
C	4.754277	2.470449	0.350494
O	5.845532	2.894705	0.652675
C	4.444287	0.98833	0.360216
C	3.289115	0.548427	1.007829
C	5.244822	0.081754	-0.33533
C	2.881073	-0.76855	0.897273
H	2.685248	1.247219	1.577192
C	4.843843	-1.23867	-0.46562
H	6.144867	0.432895	-0.82791
C	3.644283	-1.64686	0.12639
H	1.987914	-1.11967	1.402803
H	5.413891	-1.93144	-1.07583
C	3.899021	-4.14029	0.176452
C	1.892066	-3.28987	-0.4432
C	5.206242	-4.27373	0.630819
C	3.057423	-5.24539	-0.05167
C	0.848484	-2.43188	-0.83965
C	1.757941	-4.70537	-0.44335
C	5.681664	-5.57159	0.812013
H	5.828687	-3.41419	0.849469

C	3.542311	-6.52545	0.142358
C	-0.36575	-3.02537	-1.16271
H	0.990595	-1.3628	-0.93105
C	0.551421	-5.26627	-0.78496
C	4.868695	-6.67814	0.567399
H	6.69829	-5.71912	1.158077
H	2.913965	-7.39445	-0.02099
C	-0.51924	-4.41076	-1.12987
H	-1.19704	-2.39613	-1.45389
H	0.413015	-6.34198	-0.79482
H	5.265375	-7.67542	0.721383
H	-1.47637	-4.84752	-1.39138
C	-4.98461	-0.90622	1.233997
C	-5.82608	0.008313	-0.66373
C	-4.15595	-1.35927	2.25951
C	-6.36071	-1.20537	1.209753
C	-6.00906	0.706782	-1.85613
C	-6.89837	-0.61878	-0.00132
C	-4.73935	-2.10121	3.278792
H	-3.09215	-1.14476	2.265524
C	-6.92395	-1.95231	2.24455
C	-7.29163	0.744562	-2.38754
H	-5.18732	1.215156	-2.34975
C	-8.17835	-0.56653	-0.55431
C	-6.10969	-2.39355	3.277868
H	-4.11814	-2.46044	4.093727
H	-7.98445	-2.18596	2.238284
C	-8.36723	0.111989	-1.74942
H	-7.46287	1.281398	-3.31551
H	-9.01371	-1.04552	-0.05224
H	-6.53497	-2.97174	4.091697
H	-9.35659	0.161492	-2.19224
N	3.168305	-2.96422	-0.06345
N	-4.6628	-0.16783	0.092294
C	2.541294	6.695726	1.215977
C	-0.00506	6.113037	0.309689
C	0.264941	7.35301	0.842701
H	-0.5251	8.093415	0.913145
C	1.554879	7.654618	1.302054
H	3.538168	6.916355	1.586518
H	1.772816	8.627075	1.729454
H	-1.01088	5.888072	-0.03279
H	2.028547	1.09632	-2.37896

Table S140. Coordinates for the optimized geometry of **7d** (S_0).

Atom	*x*	*y*	*z*
C	6.750425	-0.624411	0.739759
C	6.871148	0.618573	0.070143
C	5.788957	1.310574	-0.603919
C	5.534678	-1.405110	0.875917
C	4.579907	0.866025	-0.996701
C	4.352444	-1.378218	0.217371
C	4.111132	-0.537811	-0.971056
H	6.006966	2.342931	-0.873962
H	5.593130	-2.191801	1.626362
C	3.677189	1.835381	-1.723245
O	4.133182	2.650060	-2.501087
O	3.513108	-0.966239	-1.946223
C	3.310466	-2.354161	0.742812
O	3.676339	-3.259427	1.471580
C	1.831959	-2.123928	0.591603
C	1.138717	-1.912549	-0.603062
C	1.098569	-2.219643	1.782735
C	-0.243284	-1.767666	-0.598598
H	1.676115	-1.855749	-1.539139
C	-0.278816	-2.077879	1.789945
H	1.622184	-2.431556	2.709083
C	-0.963026	-1.843390	0.593162
H	-0.768246	-1.571057	-1.526304
H	-0.833890	-2.179731	2.716234
C	2.218496	1.787491	-1.410056
C	1.753957	1.400919	-0.151620
C	1.305255	2.195184	-2.384404
C	0.395120	1.411315	0.130766
H	2.452657	1.082032	0.617231
C	-0.053443	2.194042	-2.113059
H	1.677094	2.516079	-3.351783
C	-0.513103	1.800161	-0.852974
H	0.028388	1.082805	1.097371
H	-0.769468	2.510894	-2.864490
C	9.221797	0.725455	0.706395
C	9.105081	-0.499291	1.366798
C	7.884374	-1.146646	1.392435
C	8.115735	1.274773	0.085441
H	10.171621	1.250184	0.687222
H	9.962791	-0.937300	1.866964
H	7.788743	-2.090577	1.921247
H	8.202057	2.234780	-0.415441

C	-3.257651	-2.369902	-0.245135
C	-3.104951	-0.958515	1.519309
C	-3.003742	-3.260850	-1.287791
C	-4.573624	-2.048968	0.138667
C	-2.667020	-0.097459	2.525956
C	-4.476265	-1.149859	1.266949
C	-4.094185	-3.791724	-1.963352
H	-1.993411	-3.542902	-1.561383
C	-5.653437	-2.596029	-0.552589
C	-3.628079	0.539772	3.296968
H	-1.611946	0.082414	2.700365
C	-5.425801	-0.496900	2.052257
C	-5.408973	-3.460580	-1.608825
H	-3.919765	-4.484954	-2.780977
H	-6.670597	-2.344967	-0.265450
C	-4.995979	0.338658	3.071828
H	-3.309591	1.227693	4.073454
H	-6.485767	-0.639958	1.861519
H	-6.238775	-3.892552	-2.159620
H	-5.721455	0.859663	3.688534
C	-2.880816	1.194151	-1.358048
C	-2.523435	2.546028	0.420700
C	-2.739600	0.344552	-2.454366
C	-4.147070	1.535132	-0.845355
C	-1.957532	3.348118	1.409772
C	-3.918708	2.408648	0.286218
C	-3.896633	-0.164687	-3.028086
H	-1.760663	0.088956	-2.847016
C	-5.295252	1.015398	-1.441768
C	-2.822059	4.003936	2.276472
H	-0.882325	3.465824	1.495356
C	-4.765619	3.078782	1.166923
C	-5.163850	0.167124	-2.531265
H	-3.814665	-0.844230	-3.870359
H	-6.276517	1.261414	-1.046028
C	-4.212718	3.873172	2.161098
H	-2.408130	4.637543	3.055540
H	-5.842778	2.973931	1.076589
H	-6.047110	-0.260091	-2.994473
H	-4.860143	4.399822	2.855396
N	-2.364630	-1.702986	0.597626
N	-1.897075	1.801869	-0.578334

9. References

S1. Dolomanov, O. V.; Bourhis, L. J.; Gildea, R. J.; Howard, J. A. K.; Puschmann, H. *OLEX2*: a complete structure solution, refinement and analysis program. *J. Appl. Cryst.* **2009**, *42*, 339–341. DOI: 10.1107/S0021889808042726

S2. Sheldrick, G. M. *SHELXT* – Integrated space-group and crystal structure determination. *Acta Crystallogr. Sect. A* **2015**, *71*, 3–8. DOI: 10.1107/S2053273314026370

S3. Sheldrick, G. M. Crystal structure refinement with *SHELXL*. *Acta Crystallogr. Sect. C* **2015**, *71*, 3–8. DOI: 10.1107/S2053229614024218

S4. Bourhis, L. J.; Dolomanov, O. V.; Gildea, R. J.; Howard, J. A. K.; Puschmann, H. The anatomy of a comprehensive constrained, restrained refinement program for the modern computing environment – *Olex2* dissected. *Acta Crystallogr. Sect. A* **2015**, *71*, 59–75. DOI: 10.1107/S2053273314022207

S5. Liégault, B.; Lee, D.; Huestis, M. P.; Stuart, D.; Fagnou, K. Intramolecular Pd(II)-catalyzed oxidative biaryl synthesis under air: reaction development and scope. *J. Org. Chem.* **2008**, *73*, 5022–5028. DOI: 10.1021/jo800596m

S6. Xiao, Y.; Hu, F.; Luo, X.; Zhao, M.; Sun, Z.; Qian, X.; Yang, Y. Advances in small-molecule fluorescent pH probes for monitoring mitophagy. *ACS Appl. Bio Mater.* **2021**, *4*, 2097–2103. DOI: 10.1021/acsabm.0c01262

S7. Miranda-Salinas, H.; Hung, Y.; Chen, Y.; Luo, D.; Kao, H.; Chang, C.; Wong, K.; Monkman, A. *J. Mater. Chem. C* **2021**, *9*, 8819–8833. DOI: 10.1021/cbmi.3c00070

S8. Frisch, M. J.; Trucks, G. W.; Schlegel, H. B.; Scuseria, G. E.; Robb, M. A.; Cheeseman, J. R.; Scalmani, G.; Barone, V.; Petersson, G. A.; Nakatsuji, H.; et al. *Gaussian 16, Revision C.02*; Gaussian, Inc.: Wallingford, CT, 2019.

S9. Chai, J.-D.; Head-Gordon, M. Systematic optimization of long-range corrected hybrid density functionals. *J. Chem. Phys.* **2008**, *128*, 084106. DOI: 10.1063/1.2834918

S10. Ditchfield, R.; Hehre, W. J.; Pople, J. A. Self-consistent molecular-orbital methods. IX. An extended Gaussian-type basis for molecular-orbital studies of organic molecules. *J. Chem. Phys.* **1971**, *54*, 724–728. DOI: 10.1063/1.1674902

S11. Bauernschmitt, R.; Ahlrichs, R. Treatment of electronic excitations within the adiabatic approximation of time dependent density functional theory. *Chem. Phys. Lett.* **1996**, *256*, 454–464. DOI: 10.1016/0009-2614(96)00440-X

S12. Wolinski, K.; Hinton, J. F.; Pulay, P. Efficient implementation of the gauge-independent atomic orbital method for NMR chemical shift calculations. *J. Am. Chem. Soc.* **1990**, *112,* 8251–8260. DOI: 10.1021/JA00179A005

S13. (a) Geuenich, D.; Hess, K.; Köhler, F.; Herges, R. Anisotropy of the induced current density (ACID), a general method to quantify and visualize electronic delocalization. *Chem. Rev.* **2005**, *105*, 3758–3772. DOI: 10.1021/cr0300901 (b) Keith, T. A.; Bader, R. F. W. Calculation of magnetic response properties using a continuous set of gauge transformations. *Chem. Phys. Lett.* **1993**, *210*, 223–231. DOI: 10.1016/0009-2614(93)89127-4

S14. Roos, B. O.; Taylor, P. R.; Siegbahn, P. E. M. A complete active space SCF method (CASSCF) using a density matrix formulated super-CI approach. *Chem. Phys.* **1980**, *48*, 157–173. DOI: 10.1016/0301-0104(80)80045-0

S15. Aidas, K.; Angeli, C.; Bak, K. L.; Bakken, V.; Bast, R.; Boman, L.; et al. The Dalton quantum chemistry program system. *Wiley Interdiscip. Rev. Comput. Mol. Sci.* **2014**, *4*, 269–284. DOI: 10.1002/wcms.1172

S16. Szczepanik, D. W.; Andrzejak, M.; Dyduch, K.; Żak, E.; Makowski, M.; Mazur, G.; Mrozek, J. A uniform approach to the description of multicenter bonding. *Phys. Chem. Chem. Phys.* **2014**, *16*, 20514–20523. DOI: 10.1039/C4CP02932A

S17. Szczepanik, D. W.; Andrzejak, M.; Dominikowska, J.; Pawełek, B.; Krygowski, T. M.; Szatylowicz, H.; Solà, M. The electron density of delocalized bonds (EDDB) applied for quantifying aromaticity. *Phys. Chem. Chem. Phys.* **2017**, *19*, 28970–28981. DOI: 10.1039/C7CP06114E

S18. J.-D. Chai, M. Head-Gordon, *J. Chem. Phys.*, **2008**, *128*, 084106. DOI: 10.1063/1.2834918

S19. J.-D. Chai, M. Head-Gordon, *Phys. Chem. Chem. Phys.*, **2008**, *10*, 6615–6620. DOI: 10.1039/B810189B

S20. L. M. da Costa, S. R. Stoyanov, S. Gusarov, X. Tan, M. R. Gray, J. M. Stryker, R. Tykwinski, J. W. de M. Carneiro, P. R. Seidl, A. Kovalenko, *Energy Fuels,* **2012**, *26*, 2727–2735.
